# A vending machine for drug-like molecules – automated synthesis of virtual screening hits†

**DOI:** 10.1039/d2sc05182f

**Published:** 2022-10-28

**Authors:** Angus E. McMillan, Wilson W. X. Wu, Paula L. Nichols, Benedikt M. Wanner, Jeffrey W. Bode

**Affiliations:** Laboratory for Organic Chemistry, Department of Chemistry and Applied Biosciences, ETH Zürich Zürich 8093 Switzerland bode@org.chem.ethz.ch; Synple Chem AG Kemptpark 18 Kemptthal 8310 Switzerland wanner@synplechem.com

## Abstract

As a result of high false positive rates in virtual screening campaigns, prospective hits must be synthesised for validation. When done manually, this is a time consuming and laborious process. Large “on-demand” virtual libraries (>7 × 10^12^ members), suitable for preparation using capsule-based automated synthesis and commercial building blocks, were evaluated to determine their structural novelty. One sub-library, constructed from iSnAP capsules, aldehydes and amines, contains unique scaffolds with drug-like physicochemical properties. Virtual screening hits from this iSnAP library were prepared in an automated fashion for evaluation against *Aedes aegypti* and *Phytophthora infestans*. In comparison to manual workflows, this approach provided a 10-fold improvement in user efficiency. A streamlined method of relative stereochemical assignment was also devised to augment the rapid synthesis. User efficiency was further improved to 100-fold by downscaling and parallelising capsule-based chemistry on 96-well plates equipped with filter bases. This work demonstrates that automated synthesis consoles can enable the rapid and reliable preparation of attractive virtual screening hits from large virtual libraries.

## Abstract 2

A compact and operationally simple automation technology can prepare virtual screening hits from a large on-demand library of drug-like molecules.

## Graphical abstract

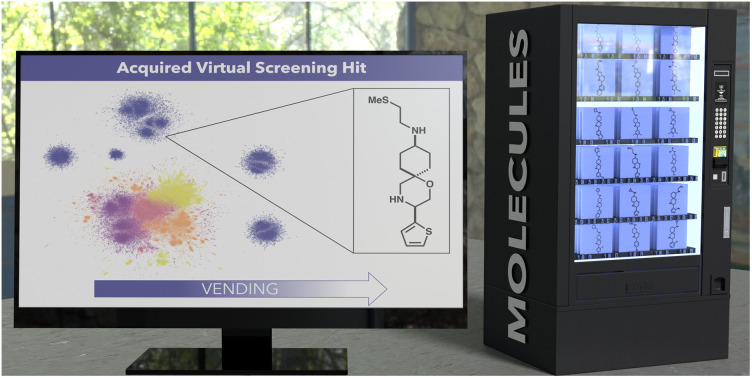

## Introduction

As part of drug discovery efforts, including those seeking to exploit novel targets and new IP space, virtual screening provides an efficient method of evaluating a vast chemical space and rationalizing structure activity relationships.^1,2^ Contrary to conventional high throughput assays, virtual screening occurs in an entirely digital environment.^3^ The near absence of physical constraints allows ultra-high throughput screening and the evaluation of molecules which do not yet physically exist.^4^ The enrichment of true positives in samples selected by virtual screening reduces the number of molecules synthetic chemists must prepare but this is limited by a high rate of false positives.^5–7^ A recent review of 53 structure based virtual screening campaigns provided a median false positive rate of 83%.^8,9^ This necessitates the synthesis of tens to hundreds of putative hit compounds in order to verify one true positive, a task which currently demands a considerable investment in time and resources.^10^ Automation of the synthesis steps can increase labour efficiencies, thereby diminishing the barrier to hit validation and enabling the digitalisation of drug discovery.^11^

Automation is particularly well suited to preparing hits from virtual or “on-demand libraries”.^12^ Such libraries are derived from a bank of building blocks and a defined set of high-fidelity reactions.^13^ They contain molecules which have a high likelihood of successfully being prepared according to a pre-determined route, ensuring synthetic accessibility, and negating retrosynthetic analysis. For example, Enamine Ltd has performed foundational work in this area by providing diverse building blocks and establishing manual on-demand synthesis of molecules from a library of 2.9 × 10^10^ members.^14,15^ Significant steps towards the fully automated synthesis of virtual screening hits have been achieved by Eli Lilly and Strateos with Idea2Data.^16^ This infrastructure unites the Proximal Lilly Collection virtual library with their automated synthesis laboratory.^17,18^

**Fig. 1:**
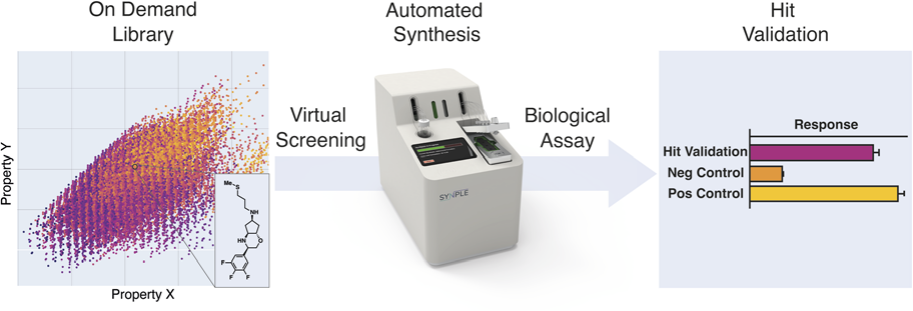
The concept of using console based automated synthesis to validate virtual screening hits.

Although such synthesis platforms offer the potential to accelerate discovery, they require large capital investments, physical infrastructure and technical staff, rendering them beyond the reach of all but the largest drug-discovery organizations. With the development of smaller, lower-cost, multi-functional automated synthesis consoles, it is now possible that the role of these costly, highly specialized automated synthesis labs may be substituted by more widely available platforms (Fig. 1).^19–21^

As part of our efforts to provide inexpensive, flexible, and predictable synthesis of drug-like compounds, we have engineered compact, capsule-based instruments for fully automated chemical synthesis.^19^ By employing fully automated assembly reactions, combined with bifunctional building blocks and commercial reagents, we can access a broad scope of attractive small molecules, with minimal time and manual operation. Limiting the range of assembly reactions does not preclude complexity or novelty, thanks to the vast scope of available building blocks.^22^ Analogous to a vending machine, this system cannot prepare any molecule imaginable, but if a virtual screening hit is identified from the virtual “on-demand” library, then it can be rapidly obtained as a physical sample with minimal effort.

## Results and discussion

**Fig. 2:**
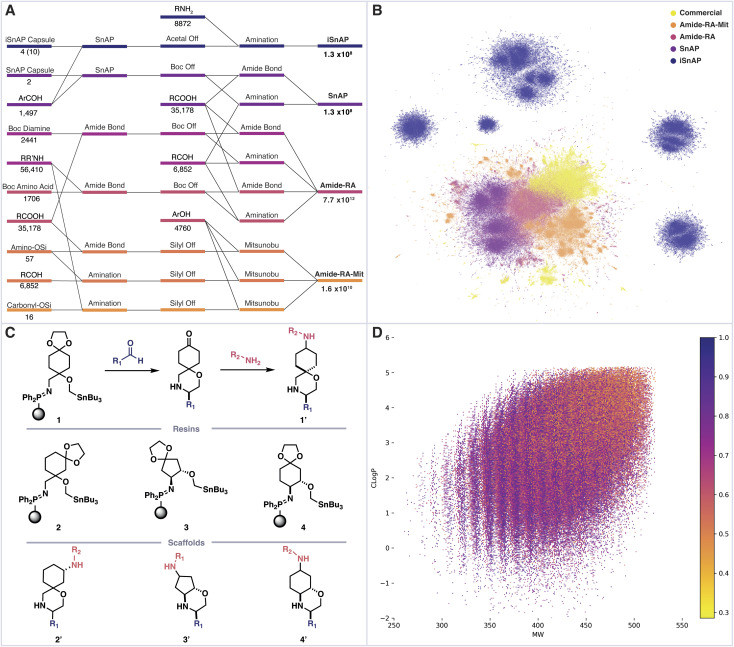
(A) An enumeration workflow, including number of initial building blocks and final library members. (B) UMAP dimensionality reduction of Morgan fingerprints from 1 × 10^5^ randomly selected members from virtual libraries and Zinc now, n_neighbours = 10, min_distance = 0.25. (C) iSnAP library is prepared from iSnAP resins, commercial aldehydes and amines. (D) iSnAP library properties cLogP, molecular weight and fraction of sp^3^ carbons.

We first sought to determine the structure and number of products which could be assembled from commercial building blocks using a linear sequence of three automated synthetic steps. To construct a virtual library of the possible products, we selected commercial building blocks, sourced from “in stock” catalogues, which had been filtered to remove problematic or incompatible functional groups. These materials were then assembled using various combinations of chemical reactions, all of which would be amenable to complete automation (setup, execution, workup, purification) using Synple's reaction capsules and instruments. The selected assembly reactions included reductive amination, amide bond formation, Mitsunobu and Sn amine protocol (SnAP) chemistry, along with appropriate automated deprotections.^23–26^ The resulting full library of decorated bifunctional scaffolds exceeds 7 trillion (7 × 10^12^) members (Fig. 2A).

A sample of 1 × 10^5^ molecules was randomly taken from each sub-library, along with ZINC now as a source of commercially available compounds.^27^ Morgan fingerprints (radius 6, bits 2048) were generated for each entry and the dimensionality reduction method UMAP was used to produce a 2D plot, in which structural similarity trends could be observed (Fig. 2B).^28,29^ Each library displayed varying degrees of structural similarity to the ZINC commercial compounds, with the exception of the iSnAP library which was largely isolated.

This particular library focused on the use of iSnAP resins to build unique spirocyclic or fused morpholines containing a ketal-protected ketone (Fig. 2C). We previously reported a capsule for the preparation of ketones, derived from resin 1, which uses the SCX-2 resin, typically used for automated purification in SnAP capsules, to affect the ketal deprotection.^19^ The resulting ketone can be reacted with amines, using a reductive amination capsule, to provide derivatives of scaffold 1′. Resins 1, 3 and 4 gave the final compounds as a mixture of two diastereomers, with the reductive amination being identified as the divergent step; whereas resin 2 provided access to all four diastereomers. The 10 accessible diastereomers of scaffolds 1′–4′ were enumerated in full using commercial aldehydes and amines to afford an iSnAP library of 1.3 × 10^8^ compounds, from which the randomly selected 1 × 10^5^ members were drawn for structural comparison.

Further analysis revealed that the structural novelty of the iSnAP virtual library likely stemmed from the use of unique scaffolds; substructure analysis against Zinc now returned no results for the iSnAP scaffolds represented as Murcko frameworks with alpha substitution.^30^ Analysis of the physicochemical properties showed good coverage of drug-like chemical space and application of Lipinski's rule of five filters proceeded with retention of 98.6% of the library (Fig. 2D).^31^ Furthermore, the average fraction of sp^3^ carbons was found to be 0.70. Such a high value is desirable in prospective drug libraries as saturation has been shown to improve physicochemical properties, such as solubility, and tends to provide less promiscuous drugs by providing more specific vectors for interactions.^32,33^

**Scheme 1:**
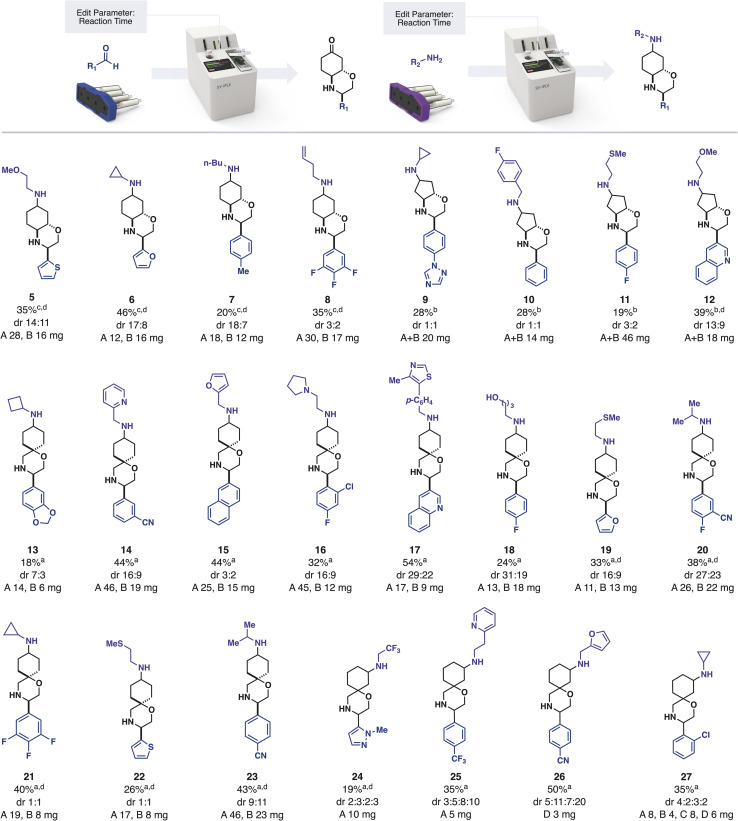
Preparation of library members; crude yield prior to resolution as determined by ^1^H NMR using 1,3,5-trimethoxybenzene as an internal standard; dr determined by HPLC; isolated mass after resolution in mg; ^a^ deprotection 5 h, reductive amination 3 h ^b^ deprotection 24 h, reductive amination 12 h ^c^ deprotection 12 h, reductive amination 12 h ^d^ pre-selected for biological activity.

We provided this iSnAP sub-library to collaboration partners with the interest and infrastructure for virtual screening. In one example, the BASF Open Innovation Platform used the library to identify compounds of interest against 12 target organisms. As part of our collaboration, we prepared these 20 compounds, along with 21 other examples, using fully automated, capsule-based synthesis (Scheme 1).

In a typical automated synthetic workflow, the starting aldehyde was added to the reaction vial and the appropriate reaction program was loaded by scanning the iSnAP capsule using the RFID scanner on the console. Upon pressing “START” the full program of operations required for the reaction and product isolation were automatically initiated, with no further user input required. The system first dissolved the aldehyde in trifluorotoluene and then circulated the solution through the first cartridge of the capsule, which contained one equivalent of the resin-bound iSnAP iminophosphorane at 80 °C. Although the rate of imine formation can be substrate dependent, we used 5 hours of circulation and found that it provided a suitable quantity of output material in all cases. After washing the resin and cooling of the reaction media, the system added one equivalent of Cu(OTf)_2_ and lutidine·HOTf by dissolving them out of the second cartridge in HFIP. Upon reaction completion, the automated program removed copper salts by passing the reaction media through a cartridge containing silica. After washing the silica with MeOH, the resulting solution was automatically loaded onto SCX-2 resin. Tributyltin byproducts and other non-basic impurities were washed from the SCX-2 before a solution of aqueous acetone was circulated over the SCX-2 to affect the ketal deprotection while the product was immobilized on the resin.

The products of resins 3 and 4 (fused morpholines) required longer deprotection times compared to resins 1 and 2. Prior to starting the program the reaction time could be edited by selecting “Edit Parameter” on the touch screen interface. Using this feature, it was found that ketones derived from resin 2 were unstable under prolonged deprotection conditions, with no ketone being detected by ^13^C NMR when the reaction time was increased to 24 hours. This demonstrates a further advantage of automation, in that experimental variables, such as reaction time, can be controlled and tuned without requiring manual actions such as removing a heat source. Upon completion of deprotection, the ketone was eluted from the SCX-2 back into the starting vial using triethylamine in acetonitrile. In the sole manual-intervention step, the solution was concentrated on a rotary evaporator and the selected amine added to the vial. This vial – containing the two reaction partners – was subjected to reductive amination using the appropriate capsule and reaction program on the console. In this reaction, the console circulated the reaction mixture over silica-supported cyanoborohydride in CH_2_Cl_2_/HFIP (4 : 1). Any unreacted amine was scavenged by automatically passing the reaction mixture through pre-swelled polymer-supported benzaldehyde and non-basic impurities were removed using SCX-2 catch and release. After elution of the product, the user concentrated the reaction mixture and added the internal standard 1,3,5-trimethyoxybenzene to determine the crude yield of the reaction *via* quantitative ^1^H NMR. In addition to crude yield, these spectra also informed us that the automated work-up was extremely effective at removing tributyltin residues. Isolation of the final products, including separation of the diastereomers formed, was achieved using standard preparative HPLC methods.

The iSnAP library products could be produced as separable diastereomeric mixtures, further adding to the library diversity. While it is often favourable in early discovery to prepare molecules as racemic mixtures, an assignment of the relatively stereochemistry for diastereoisomers is essential in order to establish a link between ligand structure and activity. The gold standard for stereochemical assignment is X-ray crystallography but growing suitable crystals is a time- and material-consuming process. To avoid negating the time-saving benefits of automation, we sought to establish a method of assignment using data from an analytical technique routinely used for characterization.

**Fig. 3:**
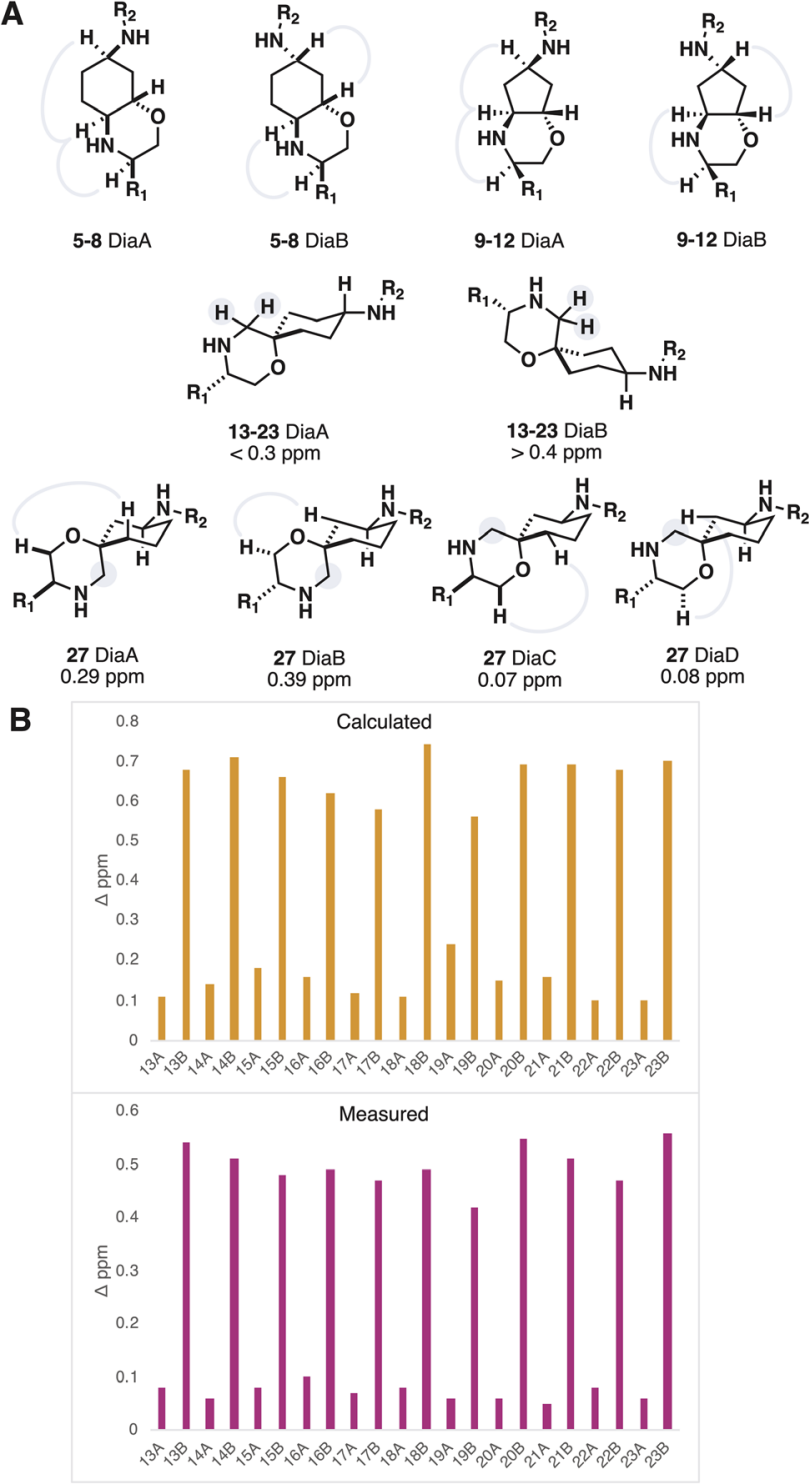
(A) Relative stereochemistry determination of accessible stereoisomers, NOE interactions and splitting of characteristic diastereotopic protons highlighted. (B) Calculated and measured diastereotopic splitting values for 13–23 using B3LYP 6-311G**.

In the case of the products derived from resins 3 and 4 (fused morpholines) the assignment of relative stereochemistry could be achieved using NOE NMR experiments (Fig. 3A). Products of resins 1 and 2 (spirocyclic morpholines) were more difficult to assign, on account of their remote and tertiary stereocentres. The NMR shielding parameters from DFT optimized conformers of compound 15 were Boltzmann weighted to provide predicted ^13^C chemical shifts for 15 DiaA and 15 DiaB. Although the predictions provided the correct assignment with low corrected mean average errors (AA′ 1.39 *vs.* AB′ 1.73, BA′ 1.90 *vs.* BB′ 1.66 ppm, B3LYP-D3 6-31G**) the low deviation between experimental results reduced the confidence in this method of assignment.

To assuredly differentiate between the two very similar diastereomeric products, we sought to identify the spectroscopic signal with maximal divergence, and make that signal the target of our predictions. We reasoned that the preference for the scaffold substituents to adopt an axial conformation would shepherd the morpholine methylene into an axial conformation for one of the diastereomers, resulting in a large diastereotopic splitting (Fig. 3A). The lowest energy conformer for both diastereomers were optimized with DFT (B3LYP 6-311G**). The difference in the ^1^H NMR shielding parameters showed good correlation, when compared to the measured diastereotopic splitting values, and consistently differentiated the two diastereomers (Fig. 3B). Furthermore, a browser-based tool with negatable calculation time also provided useable predictions of the diastereotopic splitting, albeit with less accuracy.^34,35^ This approach became the default method of assigning the relative stereochemistry of spirocyclic morpholines, augmented in examples with three stereocenters by NOE interactions. The method was validated using X-ray crystallography and can be run in parallel to the automated synthesis so as to not adversely influence hit preparation time.^36,37^ Upon compound isolation, only the acquisition of a ^1^H NMR spectrum is needed to make the assignment.

With the assignments in hand, the synthesized hit compounds were screened *in vivo* against the 12 target organisms selected by the BASF Open Innovation Platform (Scheme 1).^38^ Compound 10 DiaB demonstrated average activity against *Aedes aegypti* in the open innovation lead finder assay, where insects were sprayed with 2500 ppm in the entrance screen. Compounds 21 DiaA, 22 DiaB and 23 DiaA also showed average activity in a hit finder assay against *Phytophthora infestans* at 31 ppm. The identification of hit molecules with even modest activity was encouraging and demonstrated that it is indeed possible to use a limited range of robust, automated reaction classes to assemble commercial building blocks into desirable molecules.

We calculated the efficiency, for both manual and automated hit preparation, as user time per molecule prepared.^39^ We estimate that manual preparation can be conducted at a rate of 2.75–5.50 user hours per molecule, not including solvent evaporation and lost time from mismatches in operation scheduling and working hours. In the automated process, preparation of the same molecules takes place at a rate of 0.28–0.55 user hours per molecule. This approximates to a 10-fold improvement in efficiency. An important distinction is that, thanks to their compact nature, the automation rate can be scaled linearly by assembling banks of synthesis consoles.

**Fig. 4:**
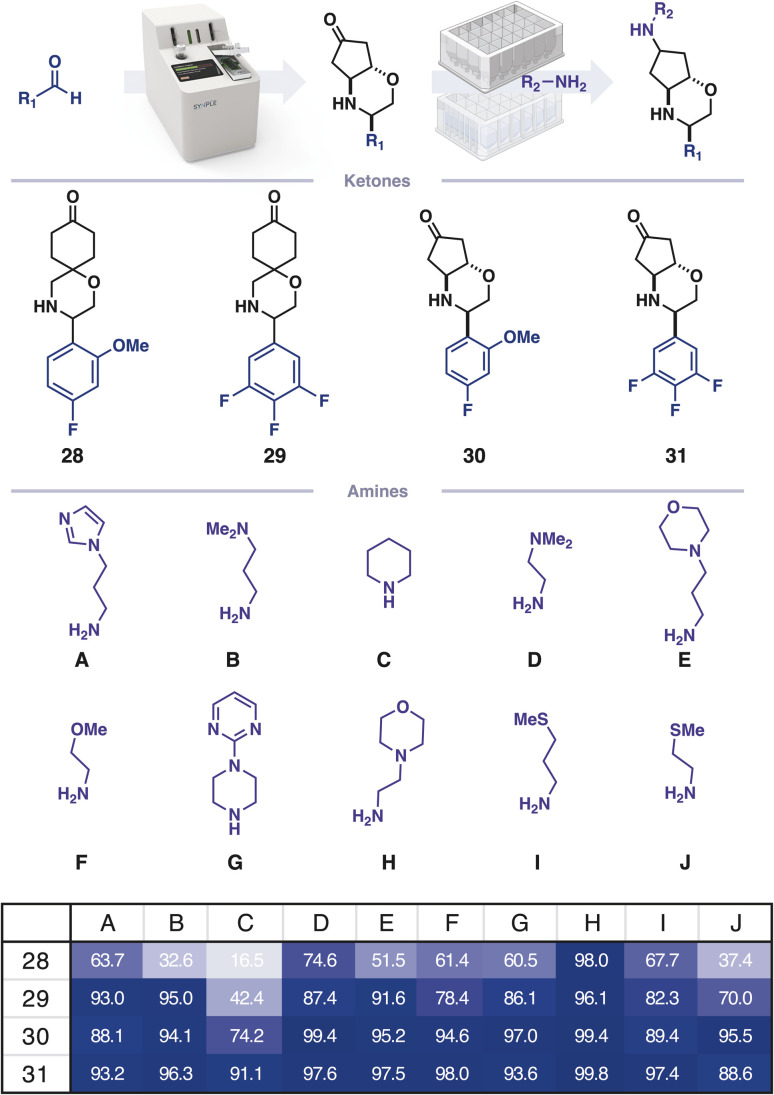
Rapid diversification using filter plate-based reductive amination; product area percentage as calculated from mass ion extracted chromatograms of ketone, alcohol byproduct and desired product.

For many screening campaigns, and in the pursuit of large datasets for machine learning, a further increase in throughput would be beneficial. Capsule based chemistry can be miniaturised to a 4 μmol scale (100-fold decrease) and performed in parallel using 96-well-plates equipped with filter floors in place of a capsule (Fig. 4). To demonstrate this, four ketones were prepared on the console and isolated. Each ketone was combined with 10 different amines in CH_2_Cl_2_/HFIP (3 : 1) and transferred to a 96-well filter plate containing silica supported cyanoborohydride. The reactions were agitated for 12 h and the crude material was subsequently obtained by centrifuging the reaction solutions through the filter into a collection plate, which was concentrated prior to analysis by high throughput LCMS.

Automated extraction of mass ion chromatograms was used for the qualitative detection of remaining starting material and an alcohol byproduct. This analysis informs subsequent screening assays which negative controls should be included, thereby allowing the method to prepare hits at a rate exceeding 0.04–0.07 user hours per molecule, giving a final 100-fold efficacy increase. This rate can be developed further by including more amines in the parallel diversification step. As the plate-based diversification has no resolution of diastereomers, this increase in throughput comes at the cost of confounding relative stereochemical effects. However, the established trends can be conveniently ratified by preparing key molecules using the initial console-based workflow.

**Scheme 2:**
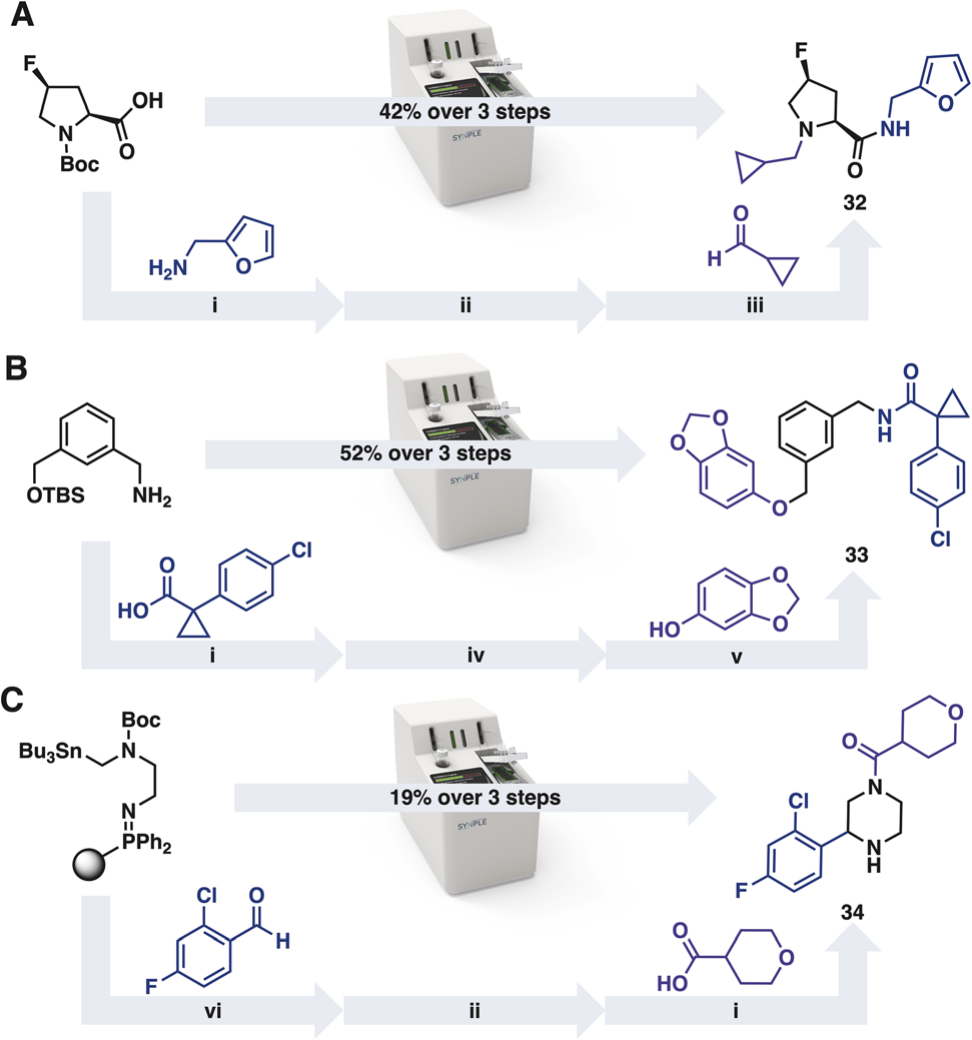
Automated synthesis of members from virtual libraries (A) Amide-RA, (B) Amide-RA-Mit and (C) SnAP libraries; i amide bond forming capsule, ii *tert*-butyloxycarbonyl (Boc) deprotection capsule, iii reductive amination capsule, iv *tert*-butyldimethylsilyl (TBS) ether deprotection capsule, v Mitsunobu capsule, vi SnAP capsule.

In addition to preparing molecules from the iSnAP library, commercial reaction capsules can be applied iteratively to prepare members from the other on-demand libraries (Scheme 2).^40^ The default reaction programs were used without optimisation. After completion of each transformation, the console performed an automated purification of the product. The resulting material was concentrated and used directly in the subsequent automated reaction. In the examples tested, ample material for screening was obtained from each of the three step reaction sequences.

## Conclusions

Synthesis consoles can use iterative automated reactions to rapidly assemble molecules from virtual libraries exceeding 7 × 10^12^ members. Structural comparison indicates that this library covers a similar space to a source of commercial compounds in addition to containing sub-libraries with unique structural elements. One structurally unique sub-library derived from commercial aldehydes, amines and iSnAP capsules was found to contain novel scaffolds with drug-like physiochemical properties. The automated preparation of screening hits from this library was demonstrated and a streamlined method of relative stereochemical assignment was devised to augment the rapid synthesis. Comparison to the manual preparation of the same molecules, the automated workflow provided a 10-fold improvement in user efficiency, which was further increased to >100-fold by using a parallel diversification strategy. Overall, the integration of console based automated synthesis and on-demand virtual libraries provides a widely deployable platform for the rapid validation of virtual screening hits.

## Data availability

Experimental data is provided in the ESI.†

## Author contributions

A. E. M. and W. W. X. W. prepared and analysed the virtual libraries. A. E. M. performed the experiments and diastereomer assignment. The manuscript was written by A. E. M., P. L. N. and J. W. B.

## Conflicts of interest

B. M. W., P. L. N. and J. W. B. are listed as inventors of a patent (WO Pat., WO2017121724A1, 2016) related to this technology and are co-founders of Synple Chem AG.

## Supplementary Material

SC-013-D2SC05182F-s001

SC-013-D2SC05182F-s002
