# Supplementary material for: A vending machine for drug-like molecules – automated synthesis of virtual screening hits
Source: Chem Sci. 2022 Oct 28;13(48):14292–9. doi: 10.1039/d2sc05182f (PMC9749103; doi:10.1039/d2sc05182f)
Supplement: SC-013-D2SC05182F-s001 [file SC-013-D2SC05182F-s001.pdf]

Electronic Supplementary Material (ESI) for Chemical Science.
This journal is © The Royal Society of Chemistry 2022

Supporting Information

A Vending Machine for Drug-like Molecules – Automated Synthesis of Virtual Screening Hits

Angus E. McMillan[1], Wilson W. X. Wu[1], Paula L. Nichols[1,2],
Benedikt M. Wanner[2]*, and Jeffrey W. Bode[1]*

bode@org.chem.ethz.ch or wanner@synplechem.com

[1]Laboratory for Organic Chemistry, Department of Chemistry and Applied Biosciences, ETH Zürich, 8093 Zürich, Switzerland.

[2]Synple Chem AG, Kemptpark 18, 8310 Kempttahl, Switzerland

Table of Contents

Virtual Library **3**
Building Blocks 3
Enumeration 4
iSnAP nomenclature 4
iSnAP Cores 5
Filtering and analysis 5

Synthesis **6**
General Remarks 6
Synthesis of iSnAP Resins 7
Resin 1 7
Resin 2 9
Resin 3 10
Resin 4 12
Loading of iSnAP resins 13
Synthesis of Library Members 13
Capsule Contents 13
General Procedure 13
Preparative HPLC 14
Plate Diversification 14
Synthesis of Compounds 5-34 17
Relative Stereochemical Assignments 44
X-Ray Crystallography 44
NOE NMR Spectra 46
Computational Procedures 56
Compound 15 ^{13}C NMR Prediction 57
Compounds 13-23 ^{1}H NMR Prediction 58

Workflow comparisons **59**

NMR Spectra Error! Bookmark not defined.

Virtual Library

Building Blocks

Functional Group	Source	Source 2	Filters Applied	Final number of members
Aromatic aldehydes	Enamine Heteroarmoatic aldehydes 20220317 (https://enamine.net/building-blocks/functional-classes/aldehydes, accessed 25/04/22)	Manually curated aromatic aldehydes from Scifinder	Acid Chloride = 0<br>Carboxylic Acid = 0<br>Sulfonyl Chloride = 0<br>Amine = 0<br>Boronic Acid = 0<br>Isocyanate = 0<br>Alcohol = 0<br>Aromatic Aldehyde = 1<br>Aliphatic Aldehyde = 0<br>Duplicates removed<br>Molecular weight < 200<br>LogP < 2	1497
Aldehydes	Enamine stock aldehydes (https://enamine.net/building-blocks/functional-classes/aldehydes, accessed 25/04/22)	None	Acid Chloride = 0<br>Carboxylic Acid = 0<br>Sulfonyl Chloride = 0<br>Amine = 0<br>Boronic Acid = 0<br>Isocyanate = 0<br>Alcohol = 0<br>Aldehyde = 1	6852
Carboxylic acids	Enamine stock carboxylic acids (https://enamine.net/building-blocks/functional-classes/acids, accessed 25/04/22)	None	Acid Chloride = 0<br>Aromatic Carboxylic Acid <= 1<br>Aliphatic Carboxylic Acid <= 1<br>Sulfonyl Chloride = 0<br>Amine = 0<br>Boronic Acid = 0<br>Isocyanate = 0<br>Alcohol = 0<br>Aldehyde = 0	35178
Phenols	Emolecules (https://www.emolecules.com, accessed 25/04/22)	None	Acid Chloride = 0<br>Carboxylic Acid = 0<br>Amine = 0<br>Boronic Acid = 0<br>Isocyanate = 0<br>Aromatic Alcohol = 1<br>Aliphatic Alcohol = 0<br>Aldehyde = 0	4760
Amines	Enamine stock primary amines (https://enamine.net/building-blocks/functional-classes/primary-amines, accessed 25/04/22)	Enamine stock secondary amines (https://enamine.net/building-blocks/functional-classes/secondary-amines, accessed 25/04/22)	Acid Chloride = 0<br>Carboxylic Acid = 0<br>Sulfonyl Chloride = 0<br>Amine = 1<br>Boronic Acid = 0<br>Isocyanate = 0<br>Alcohol = 0<br>Aldehyde = 0	56410
Primary Amines	Enamine stock primary amines (https://enamine.net/building-blocks/functional-classes/primary-amines, accessed 25/04/22)	Manually curated primary amines from Scifinder	Primary Amine = 1<br>Secondary Amine =0<br>Aromatic amine = 0<br>Duplicates removed<br>Molecular weight < 200<br>LogP < 2	8872
Boc-diamines	Manually sourced* boc-diamines from Scifinder(listed below)	Emolecules (https://www.emolecules.com, accessed 25/04/22)	Amine = 1<br>Boc-amine = 1	2441
Boc-amino acids	Manually sourced* Boc-amino acids from Scifinder	Emolecules (https://www.emolecules.com, accessed 25/04/22)	Carboxylic Acid = 1<br>Boc-amine = 1	1706
Carbonyl-OSi	Manually sourced* Carbonyl-OSi from Scifinder	Emolecules (https://www.emolecules.com, accessed 25/04/22)	Duplicates removed<br>Manual inspection	16
Amine-OSi	Manually sourced* Amine-OSi from Scifinder	Emolecules (https://www.emolecules.com, accessed 25/04/22)	Duplicates removed<br>Manual inspection	57

*Manually sourced from the Scifinder database using sub-structure search function.[1]

[1] CAS Scifinder^{n}, https://scifinder-n.cas.org, (accessed August 2020).

Enumeration

Enumerations were carried out using RDKit 1 and 2 component reaction nodes in KNIME.[2,3] Input materials were used in SMILES format and transformations were defined using SMARTS.[4]

iSnAP nomenclature

An in-house nomenclature was used for the naming of computational jobs. The one-line representation can hold chemical information about the X heteroatom used, scaffold motif, ring size(s), bridge head position(s) (if any), position of reductive amination, and stereochemistry. Consisting only of letters, numbers and underscores, the nomenclature is suitable for filename and command line usage. The heteroatom and ring size formed in the SnAP reaction are written first (e.g. O6 meaning morpholine), followed by the stereochemical assignment of the substituted carbon derived from the imine (e.g. O6_R). Next, numbers are used to denote the position of substitution on the newly formed ring, with the nature of the substitution being denoted as “spi” or “fus”, meaning spirocyclic or fused (e.g. O6_R_5_spi). The ring size is then combined with any “spi” or “fus” descriptors, and finally, the shortest number of carbons to the site of functionalization is included with stereochemistry (e.g. O6_R_5_spi6_3R_ami).

[2] M. R. Berthold, N. Cebron, F. Dill, T. R. Gabriel, T. Kötter, Thorsten Meinl, Peter Ohl, Christoph Sieb, Kilian Thiel, Bernd Wiswedel, *Data analysis, machine learning and applications: proceedings of the 31st Annual Conference of the Gesellschaft für Klassifikation e.V., Albert-Ludwigs-Universität Freiburg, March 7-9, 2007*, Springer, Berlin, 2008, ch. 4, pp 319.

[3] Open-source cheminformatics; http://www.rdkit.org

[4] Daylight Chemical Information Systems; https://www.daylight.com

iSnAP Cores

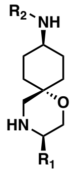
O6_R_5r_spi6_4R_ami

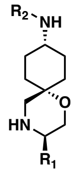
O6_R_5s_spi6_4S_ami

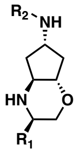
O6_R_5S_6S_fus5_3S_ami

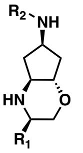
O6_R_5S_6S_fus5_3R_ami

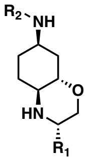
O6_S_5S_6S_fus6_3R_ami

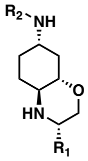
O6_S_5S_6S_fus6_3S_ami

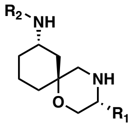
O6_R_5S_spi6_3S_ami

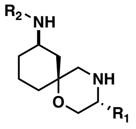
O6_R_5S_spi6_3R_ami

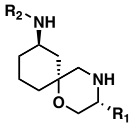
O6_R_5R_spi6_3R_ami

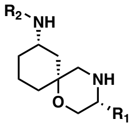
O6_R_5R_spi6_3S_ami

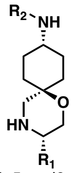
O6_S_5r_spi6_4S_ami

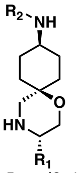
O6_S_5s_spi6_4R_ami

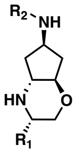
O6_S_5R_6R_fus5_3R_ami

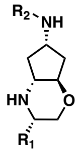
O6_S_5R_6R_fus5_3S_ami

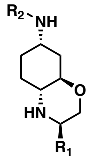
O6_R_5R_6R_fus6_3S_ami

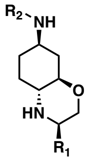
O6_R_5R_6R_fus6_3R_ami

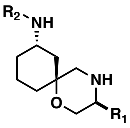
O6_S_5S_spi6_3S_ami

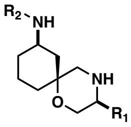
O6_S_5S_spi6_3R_ami

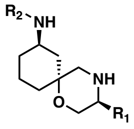
O6_S_5R_spi6_3R_ami

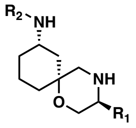
O6_S_5R_spi6_3S_ami

Filtering and analysis

Properties were generated using RDKit property generator in KNIME. Lipinski's rule of 5 was defined as MW ≤ 500, -5 ≤ LogP ≥ 5, HBD ≤ 5, HBA ≤ 10. These property ranges were applied to the iSnAP library using a rule-based filter, 98.6% passed the filter. Row filter node was used to randomly select sub-populations from libraries.

MW vs LogP scatter plot was generated using seaborn and matplotlib python libraries.[5]

Morgan fingerprint descriptors (radius = 6, nBits = 2048) were generated from SMILES using RDkit and reduced in dimension using UMAP (metric = jaccard, n_neighbours = 10, min_distance = 0.25) from sklearn.[6] The resulting plot was generated using seaborn.

---

[5] M. Waskom, *JOSS*, 2021, **6**, 3021.

[6] F. Pedregosa, G. Varoquaux, A. Gramfort, V. Michel, B. Thirion, O. Grisel, M. Blondel, P. Prettenhofer, R. Weiss, V. Dubourg, J. Vanderplas, A. Passos and D. Cournapeau, *Journal of Machine Learning Research*, 2011, **12**, 2825.

Synthesis

General Remarks

Unless otherwise noted, all reactions were performed under N_2 with anhydrous conditions. All reagents were used as received from commercial suppliers.

Reactions were monitored by thin layer chromatography (TLC) on Merck precoated aluminum-backed silica gel 60 F254 plates with UV at 254 nm, or by subjecting to NMR analysis. Flash chromatography purification was performed on Silicycle Silica Flash F60 (230–400 Mesh) silica gel using a forced flow of eluent at 0.2-0.3 bar.

NMR spectra were recorded on Bruker Avance III at 400 or 500 MHz (H) and at 100, 125 MHz (C), respectively, using $CDCl_3$ as the solvent unless indicated otherwise. Chemical shifts (δ) are reported in ppm, using the residual solvent peak in $CDCl_3$ (H: δ = 7.26 ppm and C: δ = 77.16 ppm) as reference. All ^{13}C spectra were measured with proton decoupling. NMR coupling constants (*J*) are reported in Hertz (Hz), and splitting patterns are indicated as follows: br, broad; s, singlet; d, doublet; dd, doublet of doublet; ddd, doublet of doublet of doublet; dt, doublet of triplet; t, triplet; q, quartet; quint; quintet; sext, sextet; m, multiplet.

High resolution mass spectra were measured by the Mass Spectrometry Service Facility of Molecular and Biomolecular Analysis Service (MoBiAS), Department of Chemistry and Applied Biosciences at ETH Zurich on a Bruker Daltonics maXis for ESI-Q-TOF spectrometer (ESI-MS).

Synthesis of iSnAP Resins

Resin 1

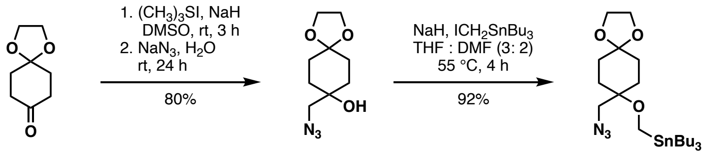

SM 1-1 1-2

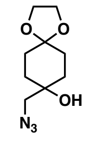

8-(azidomethyl)-1,4-dioxaspiro[4.5]decan-8-ol (1-1)

Trimethylsulfonium iodide (50.7 g, 230 mmol, 1.20 equiv) was dissolved in anhydrous DMSO (600 mL) and cooled with an ice bath. NaH (60 wt%, 14.7 g, 231 mmol, 1.20 equiv) was added portionwise then 1,4-dioxaspiro[4.5]decan-8-one (30.0 g, 192 mmol, 1.00 equiv) in DMSO (300 mL) was added dropwise. The reaction was allowed to return to rt over 3 h. The reaction was diluted with H_2O (600 mL) and the product was extracted in MTBE (5 x 500 mL). The organic phase was washed with H_2O (300 mL) and concentrated to afford a clear oil. H_2O (600 mL) and NaN_3 (62.4 g, 960 mmol, 5.00 equiv) were added. After stirring at rt for 24 h the product was extracted in EtOAc (5 x 200 mL). The organic phase was dried over anhydrous Na_2SO_4 then concentrated to dryness to afford a crystalline solid. The product was recrystallized in 400 mL of a 1:1 Et_2O : *n*-hexane solution to afford the title compound as a colorless crystalline solid (32.9 g, 154 mmol, 80% yield); ^1H NMR ($CDCl_3$, 300 MHz): δ 4.03–3.91 (m, 4H), 3.32 (s, 2H), 2.00–1.84 (m, 2H), 1.81–1.57 (m, 7H); ^{13}C NMR ($CDCl_3$, 100 MHz): δ 108.4, 70.6, 64.4, 64.2, 61.7, 32.5 (2C), 30.0 (2C); HRMS (ESI) *m/z* for $C_9H_{15}N_3O_3$ $[M+Na]^+$ calcd. 236.1006, found 236.1011; IR (ν/cm^{-1}, thin film) 3458, 2932, 2102, 1373, 1282, 1105, 1036, 923; m.p. 45 °C.

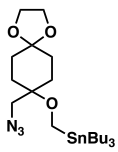

(((8-(azidomethyl)-1,4-dioxaspiro[4.5]decan-8-yl)oxy)methyl)tributylstannane (1-2)

NaH (60 wt%, 3.38 g, 84.4 mmol, 1.20 equiv) and anhydrous THF (110 mL) were added to a dry 500 mL r.b. flask. 8-(Azidomethyl)-1,4-dioxaspiro[4.5]decan-8-ol (15.0 g, 70.3 mmol, 1.00 equiv) was dissolved in DMF (75 mL) and added dropwise to the reaction. After stirring at 55 °C for 30 mins, the slurry became a light orange solution. Tributyl(iodomethyl)stannane (36.4 g, 84.4 mmol, 1.20 equiv) was added dropwise and a white precipitate began to form. After 3 h at 55 °C the reaction was cooled to rt and diluted with H_2O (200 mL). The product was extracted in MTBE (4 x 100 mL), dried over anhydrous Na_2SO_4 and concentrated under reduced pressure. The product was purified by flash silica chromatography (5% EtOAc in *n*-hexane) to afford the title compound as a clear oil (33.4 g, 64.7 mmol, 92% yield); ^1H NMR ($CDCl_3$, 400 MHz): δ 4.02–3.89 (m, 4H), 3.44 (t, J = 12.0 Hz, 2H), 3.23 (s, 2H), 1.97–1.87 (m, 2H), 1.81–1.70 (m, 2H), 1.58–1.48 (m, 10H), 1.39–1.25 (m, 6H), 0.97–0.88 (m, 15H); ^{13}C NMR

($CDCl_3$, 100 MHz): δ 108.7, 75.6, 64.3, 64.2, 54.8, 49.9, 29.9 (2C), 29.2 (3C), 28.9 (2C), 27.4 (3C), 13.8 (3C), 9.0 (3C); HRMS (ESI) *m/z* for $C_{22}H_{43}N_3O_3Sn$ $[M+Na]^+$ calcd. 540.2223, found 540.2224; IR (ν/cm^{-1}, thin film) 3446, 2956, 2360, 2097, 1635, 730, 688 cm.

Resin 2

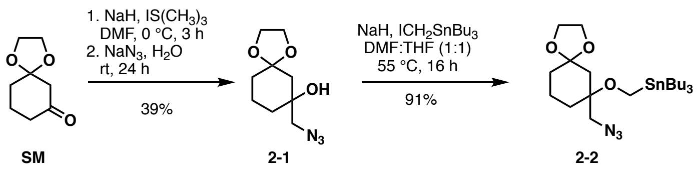

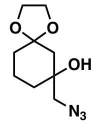

7-(azidomethyl)-1,4-dioxaspiro[4.5]decan-7-ol (2-1)

NaH (60 wt%, 0.66 g, 16.5 mmol, 1.60 equiv) was added to a solution of trimethylsulfonium iodide (2.95 g, 14.4 mmol, 1.40 equiv) in anhydrous DMF (52 mL) at 0 °C. After 15 mins, 1,4-dioxaspiro[4.5]decan-7-one (1.61 g, 10.3 mmol, 1.00 equiv) was added and the reaction was allowed to return to rt over 3 h. The reaction was quenched with H_2O (100 mL) and the product was extracted in EtOAc (5 x 50 mL). The extractions were combined and concentrated to dryness. NaN_3 (3.35 g, 51.5 mmol, 5.00 equiv) in H_2O (100 mL) was added to the resulting oil and the mixture was stirred at rt for 24 h. The product was extracted in EtOAc (5 x 50 mL), dried over anhydrous Na_2SO_4, concentrated to dryness and purified by flash silica chromatography (0-30% EtOAc in *n*-hexane) to afford the title compound as a clear oil (0.86 g, 4.06 mmol, 39% yield); ^1H NMR (400 MHz, $CDCl_3$) δ 4.07 – 3.89 (m, 4H), 3.27 (d, J = 12.4 Hz, 1H), 3.11 (d, J = 12.4 Hz, 1H), 1.92 – 1.58 (m, 6H), 1.54 – 1.43 (m, 1H), 1.36 – 1.25 (m, 1H), exchangeable 1 x OH not observed; ^{13}C NMR (100 MHz, $CDCl_3$) δ 109.2, 72.4, 64.6, 64.2, 60.7, 41.4, 34.5, 34.1, 18.6; HRMS (ESI) *m/z* calcd. for $C_9H_{15}N_3O_3$ $[M+Na]^+$ 236.1006 found 236.1012; IR (ν/cm^{-1}, thin film) 3506, 2948, 2886, 2102, 1449, 1277, 1168, 1077, 947, 834.

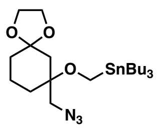

(((7-(azidomethyl)-1,4-dioxaspiro[4.5]decan-7-yl)oxy)methyl)tributylstannane (2-2)

NaH (60 wt%, 226 mg, 5.65 mmol, 1.40 equiv) was added to a solution of 7-(azidomethyl)-1,4-dioxaspiro[4.5]decan-7-ol (0.86 g, 4.03 mmol, 1.00 equiv) in THF:DMF (1:1 40 mL) at 0 °C. After 0.4 h, tributyl(iodomethyl)stannane (2.09 g, 4.84 mmol, 1.20 equiv) was added and the reaction was stirred for 3 h. The reaction was then heated to 55 °C for 15 h. After returning to rt, the reaction was quenched with H_2O (150 mL) and the product was extracted in EtOAc (4 x 80 mL). The organic phase was dried over anhydrous Na_2SO_4, concentrated and the product was purified by flash silica chromatography (0-10% EtOAc in *n*-hexane) to afford the title compound as a clear oil (1.89 g, 3.66 mmol, 91% yield); ^1H NMR (400 MHz, $CDCl_3$) δ 4.03 – 3.88 (m, 4H), 3.56 – 3.33 (m, 4H), 1.87 – 1.79 (m, 2H), 1.79 – 1.62 (m, 3H), 1.60 – 1.42 (m, 9H), 1.38 – 1.25 (m, 6H), 1.03 – 0.85 (m, 15H); ^{13}C NMR (100 MHz, $CDCl_3$) δ 109.1, 78.6, 64.4, 64.1, 53.9, 49.4, 38.9, 34.7, 30.7, 29.1 (3C), 27.3 (3C), 19.6, 13.8 (3C), 9.0 (3C); HRMS (ESI) *m/z* calcd. for $C_{22}H_{43}N_3O_3Sn$ $[M+Na]^+$ 540.2223, found 540.2229; IR (ν/cm^{-1}, thin film) 2954, 2925, 2098, 1455, 1340, 1311, 1067, 952, 864.

Resin 3

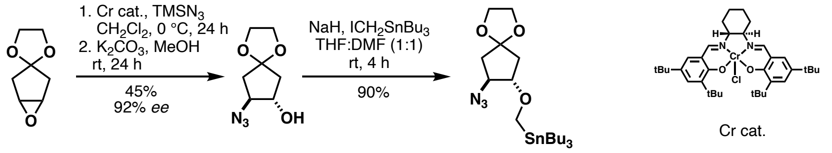

SM	3-1	3-2

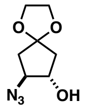

(7*S*,8*S*)-8-azido-1,4-dioxaspiro[4.4]nonan-7-ol (3-1)

6-oxaspiro[bicyclo[3.1.0]hexane-3,2'-[1,3]dioxolane (3.07 g, 21.6 mmol, 1.00 equiv), (*R*,*R*)-*N*,*N'*-*bis*(3,5-di-tert-butylsalicylidene)-1,2-cyclohexanediaminochromium(III) chloride (0.63 g, 0.43 mmol, 0.02 equiv) and trimethylsilyl azide (2.97 g, 43.1 mmol, 2.00 equiv) were combined and stirred at rt for 24 h. The reaction was cooled to 0 °C and added dropwise to a cold solution of aq NaOH (2 M, 200 mL). The product was extracted in EtOAc (4 x 50 mL). The organic phase was washed with H_2O, dried over anhydrous Na_2SO_4 and concentrated under reduced pressure to dryness. Potassium carbonate (5.96 g, 43.2 mmol, 2.00 equiv) and MeOH (100 mL) were added to the resulting oil and the slurry was stirred at rt for 24 h. The slurry was passed through a filter and the filtrate was concentrated and purified by flash silica chromatography (25% EtOAc in *n*-hexane) to afford the title compound as a clear oil (1.78 g, 9.60 mmol, 45% yield, 92% *ee*); ^{1}H NMR (400 MHz, $CDCl_3$) δ 4.19 – 4.08 (m 1H), 3.99 – 3.90 (m, 4H), 3.85 (ddd, J = 7.4, 7.4, 4.9 Hz, 1H), 2.40 (ddd, J = 14.3, 7.8, 1.3 Hz, 1H), 2.34 – 2.26 (m, 2H), 1.97 – 1.86 (m, 2H); ^{13}C NMR (100 MHz, $CDCl_3$) δ 113.9, 75.4, 66.4, 65.0, 64.4, 42.9, 40.4; HRMS (ESI) *m/z* for $C_7H_{11}N_3O_3$ $[M+Na]^+$ calcd. 208.0693, found 208.0693, IR (ν/cm^{-1}, thin film) 3431, 2889, 2102, 1320, 1262, 1086, 1058, 1011 cm^{-1}; determination of enantiomeric excess was achieved using a copper catalyzed azide alkyne click reaction with phenyl acetylene and analyzed using normal phase chiral HPLC according to a previous report.[7] NP-HPLC, column: Daicel Chiralpak ADH (4.6 x 250 mm); eluent: EtOH : *n*-hexane 1:1; flow rate: 0.5 mL/min; detection: 254 nm; retention time: 28.6 min (major), 40.2 min (minor).

Racemic

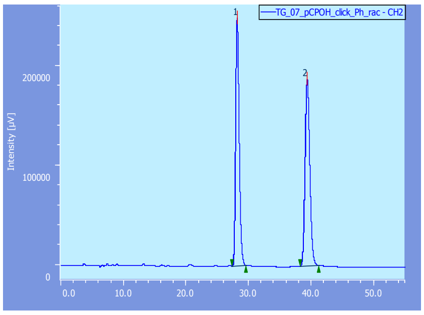

Enantioenriched

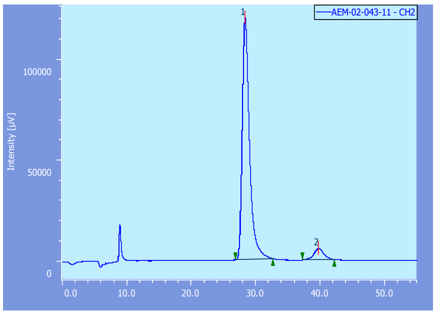

[7] F. Saito, N. Trapp and J. Bode, *J. Am. Chem. Soc.*, 2019, **141**, 5544.

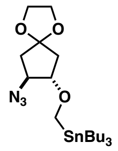

((((7*S*,8*S*)-8-azido-1,4-dioxaspiro[4.4]nonan-7-yl)oxy)methyl)tributylstannane (3-2)
NaH (60 wt%, 0.42 g, 10.4 mmol, 1.20 equiv) was added to a solution of (7*S*,8*S*)-8-azido-1,4-dioxaspiro[4.4]nonan-7-ol (1.60 g, 8.65 mmol, 1.00 equiv) in THF (43 mL) at 0 °C. After 30 mins, tributyl(iodomethyl)stannane (4.47 g, 10.4 mmol, 1.20 equiv) was added. The reaction was allowed to warm to rt over 4 h. The reaction was quenched with H_2O (200 mL) and the product was extracted in MTBE (4 x 100 mL). The organic phases were combined and dried over anhydrous Na_2SO_4, concentrated to dryness and purified by flash silica chromatography (0-15% EtOAc in *n*-hexane) to afford the title compound as a clear oil (3.79 g, 7.77 mmol, 90 % yield); ^{1}H NMR (400 MHz, $CDCl_3$) δ 3.96 – 3.89 (m, 4H), 3.83 (td, *J* = 7.7, 5.5 Hz, 1H), 3.78 – 3.67 (m, 2H), 3.66 (td, *J* = 7.1, 5.5 Hz, 1H), 2.34 (ddd, *J* = 13.9, 7.5, 1.4 Hz, 1H), 2.27 (ddd, *J* = 14.0, 8.2, 1.1 Hz, 1H), 1.90 – 1.80 (m, 2H), 1.65 – 1.41 (m, 6H), 1.39 – 1.25 (m, 6H), 1.03 – 0.82 (m, 15H); ^{13}C NMR (100 MHz, $CDCl_3$) δ 113.8, 86.5, 64.4, 64.4, 64.2, 60.5, 40.9, 40.1, 29.1(3C), 27.3(3C), 13.7(3C), 8.9(3C); HRMS (ESI) *m/z* for $C_{20}H_{39}N_3O_3Sn$ $[M+Na]^+$ calcd. 512.1909, found 512.1918; IR (ν/cm^{-1}, thin film) 2954, 2922, 2360, 2102, 1463, 1259, 874, 684; $[\alpha]^{25}$ +28° (c 0.4, CH_2Cl_2).

Resin 4

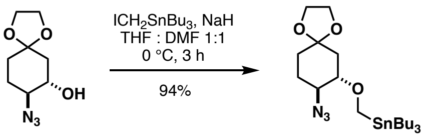

SM 4-1

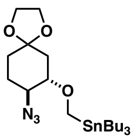

((((7*S*,8*S*)-8-azido-1,4-dioxaspiro[4.5]decan-7-yl)oxy)methyl)tributylstannane (4-1)
(7*S*,8*S*)-8-azido-1,4-dioxaspiro[4.5]decan-7-ol (1.24 g, 6.23 mmol, 1.00 equiv) was dissolved in a mixture of DMF and THF (60 mL, 1:1) and cooled to 0 °C. NaH (0.30 g, 7.48 mmol, 1.20 equiv) was added and the reaction was stirred for 20 mins. Tributyl(iodomethyl)stannane (3.22 g, 7.47 mmol, 1.20 equiv) was added and the reaction was stirred at 0 °C for 3 h. The reaction was quenched with H_2O (100 mL) and the product was extracted in EtOAc (4 x 100 mL). The organic phase was dried over anhydrous $MgSO_4$, concentrated and the product was purified by flash silica chromatography (0-10% EtOAc in *n*-hexane) to afford the title compound as a clear oil (2.94 g, 5.86 mmol, 94% yield); ^{1}H NMR (400 MHz, $CDCl_3$) δ 4.07 – 3.81 (m, 5H), 3.73 – 3.58 (m, 1H), 3.36 – 3.13 (m, 2H), 2.29 (ddd, *J* = 12.7, 4.2, 3.0 Hz, 1H), 1.90 – 1.83 (m, 1H), 1.73 (dq, *J* = 12.3, 3.0 Hz, 1H), 1.66 – 1.41 (m, 9H), 1.40 – 1.26 (m, 6H), 1.07 – 0.83 (m, 15H); ^{13}C NMR (101 MHz, $CDCl_3$) δ 108.3, 83.8, 64.5, 64.4, 64.1, 59.6, 38.4, 32.8, 29.1 (3C), 27.3 (3C), 26.6, 13.7 (3C), 8.9 (3C); HRMS (ESI) *m/z* for $C_{21}H_{41}N_3O_3Sn$ $[M+H]^+$ calcd. 526.2066, found 526.2060; IR (ν/cm^{-1}, thin film) 2953, 2923, 2098, 1456, 1262, 1068, 921.

Loading of iSnAP resins

A solution of the iSnAP reagent in CH_2Cl_2 (0.2 mol/L, 1.0 equiv) was added to polystyrene-supported triphenylphosphine (1.5 eq) and stirred at rt for 24 h. The solvent was removed under reduced pressure to afford the iSnAP resin. Loading was determined by allowing the resin to react with an excess of 4-bromobenzaldehyde in CH_2Cl_2 and determining the conversion after 24 h by ^1H NMR.

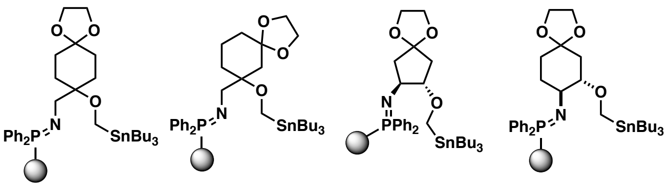

1 0.46 mmol/g | 2 0.60 mmol/g | 3 0.64 mmol/g | 4 0.35 mmol/g

Synthesis of Library Members

Capsule Contents

iSnAP Capsule

Cartridge 1: iSnAP resin (0.5 mmol, 1.0 equiv), 4 Å molecular sieves (100 mg)

Cartridge 2: $Cu(OTf)_2$ (0.5 mmol, 1.0 equiv), lutidinium triflate (0.5 mmol, 1.0 equiv)

Cartridge 3: SCX-2 (2.0 mmol, 4.0 equiv)

Cartridge 4: Silica (5 g)

Reductive Amination Capsule

Cartridge 1: silica supported cyanoborohydride (1.0 mmol, 2.0 equiv)

Cartridge 2: SCX-2 (2.0 mmol, 4.0 equiv)

Cartridge 3: polystyrene supported benzaldehyde (1.0 mmol, 2.0 equiv)

General Procedure

The aldehyde (0.5 mmol) and a stirring bar were added to a vial. The vial was inserted into the vial holder of console and the line cap was attached. The iSnAP capsule was scanned to load the correct program and placed into the capsule holder, which was closed and locked. The ketal deprotection time was set using the "edit recipe" menu [resin **1**, 5 h; resin **2**, 5 h; resin **3**, 24 h; resin **4** 12 h]. After checking the solvents were full and the waste container was not, the program was initiated by pressing "run" on the touch screen interphase. Upon program competition, the solution in the vial was concentrated using a vial adaptor and standard rotary evaporator.

The amine (0.5 mmol) was added to the product containing vial from step 1 and the vial was returned to the console. The reductive amination capsule was scanned to load the program, and placed to the capsule holder, which was closed and locked. The reaction time was set using the "edit recipe" menu [resin **1**, 3 h; resin **2**, 3 h; resin **3**, 12 h; resin **4** 12 h]. The program was initiated by pressing "run" on the touch screen interphase. Upon program completion, the stirring bar was removed, the solution was concentrated as before and trimethoxybenzene (0.1 mmol) was added (as an internal standard). The crude yield was determined by ^1H NMR and the product was purified by high pressure liquid chromatography.

Preparative HPLC

Preparative reverse phase high performance liquid chromatography was carried out on a Jasco preparative instrument with dual pumps, mixer, in-line degasser, Rheodyne 7725i injector with 10 mL injection loop and a variable wavelength UV with detection at 220, 254 and 301 nm. The generic method used a CAPCELL PAK C18 (20 mmI.D x 250 mm, 5 μm, Cat. No. 92539) column and ran the following gradient at a flow rate of 10 mL/min. Time (min), mobile phase (% of CH_3CN in H_2O with 0.1% TFA).

Time 00 Mobile Phase 5,
Time 10 Mobile Phase 70,
Time 30 Mobile Phase 95,
Time 35 Mobile Phase 95,
Time 36 Mobile Phase 10.

Compound elution occurred between 10 and 20 min and the internal standard (1,3,5-trimethoxybenzene) eluted at 31 min.

Plate Diversification

Silica supported cyanoborohydride (10 mg, 1.0 mmol/g, 10 μmol, 2.5 equiv) was added to each well of a 96-well filtration plate. The filter base of the plate was sealed from below using pre-cut sealing tape for 96-well plates. A solution of each ketone (30 μL, 130 mM in CH_2Cl_2, 4.0 μmol, 1.0 equiv) was distributed in each row of the plate. A solution of each amine (10 μL, 600 mM in HFIP, 6.0 μmol, 1.5 equiv) was distributed to each column of the plate. The plate was agitated for 12 h at rt. CH_3CN (200 μL) was added to each well of the plate. The sealing tape was removed from the based, the filtration plate was stacked onto a collection plate and centrifuged to transfer the solution. The crude material was obtained by concentrating the solutions with N_2 using a blow down apparatus. Each product was dissolved in CH_3CN (1 mL) and diluted 100-fold prior to analysis.

Plate layout with the structures of expected products.

(<5 umol/ well)	1	2	3	4	5	6	7	8	9	10
A	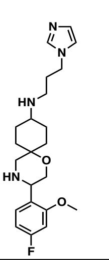	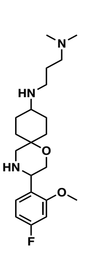	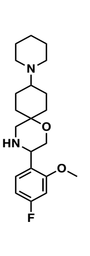	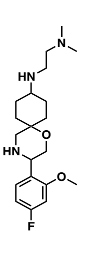	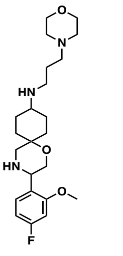	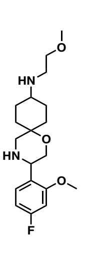	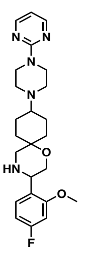	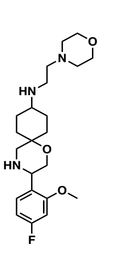	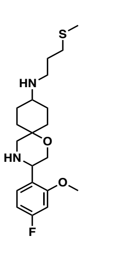	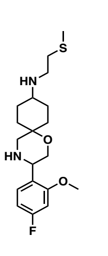
B	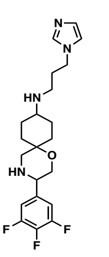	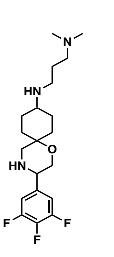	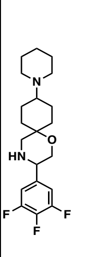	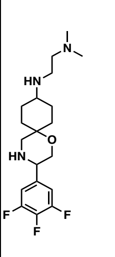	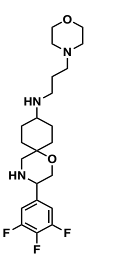	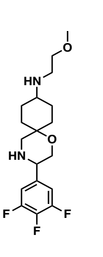	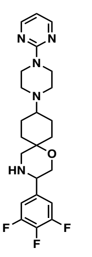	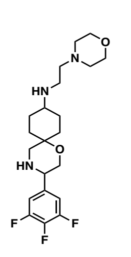	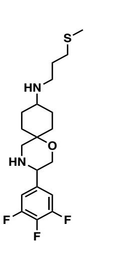	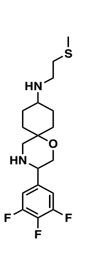
C	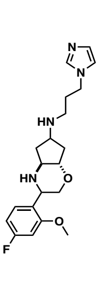	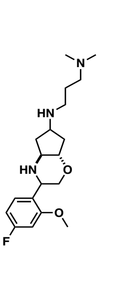	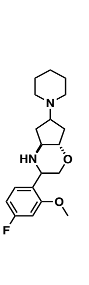	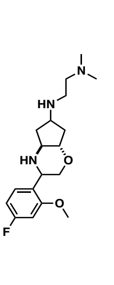	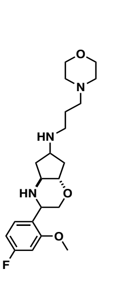	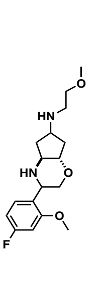	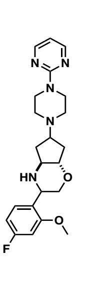	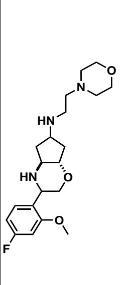	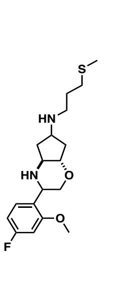	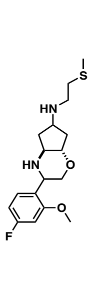
D	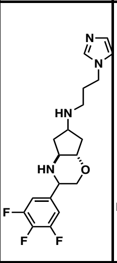	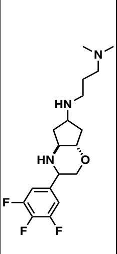	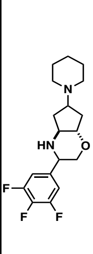	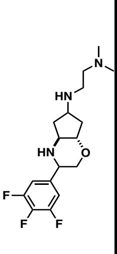	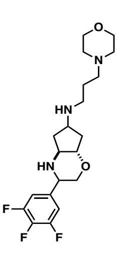	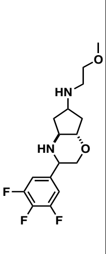	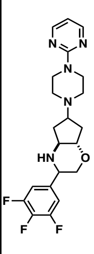	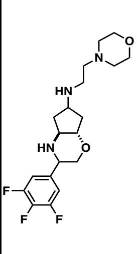	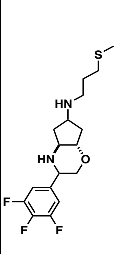	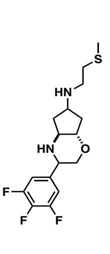

Processed results with sum formula, extracted ion chromatogram area and area %

Plate Well	Ketone Formula	Alcohol Formula	Product Formula	Ketone Area	Alcohol Area	Product Area	Ketone Area %	Alcohol Area %	Product Area %
A1	C16H20FNO3	C16H22FNO3	C22H31FN4O2	0	159205	278799	0.0	36.3	63.7
A2	C16H20FNO3	C16H22FNO3	C21H34FN3O2	25220	14523738	7023891	0.1	67.3	32.6
A3	C16H20FNO3	C16H22FNO3	C21H31FN2O2	101639	43634719	8663670	0.2	83.3	16.5
A4	C16H20FNO3	C16H22FNO3	C20H32FN3O2	0	3460857	10150292	0.0	25.4	74.6
A5	C16H20FNO3	C16H22FNO3	C23H36FN3O3	0	3313868	3524346	0.0	48.5	51.5
A6	C16H20FNO3	C16H22FNO3	C19H29FN2O3	42729	9594204	15337054	0.2	38.4	61.4
A7	C16H20FNO3	C16H22FNO3	C24H32FN5O2	169425	47608084	73102188	0.1	39.4	60.5
A8	C16H20FNO3	C16H22FNO3	C22H34FN3O3	0	2720522	130425360	0.0	2.0	98.0
A9	C16H20FNO3	C16H22FNO3	C20H31FN2O2S	42755	18105242	37994303	0.1	32.2	67.7
A10	C16H20FNO3	C16H22FNO3	C19H29FN2O2S	13538	9159878	5477447	0.1	62.5	37.4
B1	C15H16F3NO2	C15H18F3NO2	C21H27F3N4O	0	3692833	48745380	0.0	7.0	93.0
B2	C15H16F3NO2	C15H18F3NO2	C20H30F3N3O	0	915915	17226837	0.0	5.0	95.0
B3	C15H16F3NO2	C15H18F3NO2	C20H27F3N2O	6768	8941057	6599151	0.0	57.5	42.4
B4	C15H16F3NO2	C15H18F3NO2	C19H28F3N3O	0	3913288	27176190	0.0	12.6	87.4
B5	C15H16F3NO2	C15H18F3NO2	C22H32F3N3O2	0	1918345	20972093	0.0	8.4	91.6
B6	C15H16F3NO2	C15H18F3NO2	C18H25F3N2O2	35563	8450674	30859625	0.1	21.5	78.4
B7	C15H16F3NO2	C15H18F3NO2	C23H28F3N5O	13012	7694328	47643524	0.0	13.9	86.1
B8	C15H16F3NO2	C15H18F3NO2	C21H30F3N3O2	0	2776532	69016722	0.0	3.9	96.1
B9	C15H16F3NO2	C15H18F3NO2	C19H27F3N2OS	32528	8468340	39467353	0.1	17.7	82.3
B10	C15H16F3NO2	C15H18F3NO2	C18H25F3N2OS	65757	16887279	39645378	0.1	29.8	70.0
C1	C14H16FNO3	C14H18FNO3	C20H27FN4O2	14518	2814773	20915534	0.1	11.9	88.1
C2	C14H16FNO3	C14H18FNO3	C19H30FN3O2	11911	1509804	24488142	0.0	5.8	94.1
C3	C14H16FNO3	C14H18FNO3	C19H27FN2O2	1553192	4272740	16744440	6.9	18.9	74.2
C4	C14H16FNO3	C14H18FNO3	C18H28FN3O2	30865	153442	30327122	0.1	0.5	99.4
C5	C14H16FNO3	C14H18FNO3	C21H32FN3O3	10047	1201219	23979274	0.0	4.8	95.2
C6	C14H16FNO3	C14H18FNO3	C17H25FN2O3	111384	357812	8275297	1.3	4.1	94.6
C7	C14H16FNO3	C14H18FNO3	C22H28FN5O2	102575	771051	28686968	0.3	2.6	97.0
C8	C14H16FNO3	C14H18FNO3	C20H30FN3O3	37002	85470	20139430	0.2	0.4	99.4
C9	C14H16FNO3	C14H18FNO3	C18H27FN2O2S	4341282	3712608	67592223	5.7	4.9	89.4
C10	C14H16FNO3	C14H18FNO3	C17H25FN2O2S	61914	672925	15692000	0.4	4.1	95.5
D1	C13H12F3NO2	C13H14F3NO2	C19H23F3N4O	0	1089035	14824338	0.0	6.8	93.2
D2	C13H12F3NO2	C13H14F3NO2	C18H26F3N3O	0	1134972	29681904	0.0	3.7	96.3
D3	C13H12F3NO2	C13H14F3NO2	C18H23F3N2O	336393	3443601	38644024	0.8	8.1	91.1
D4	C13H12F3NO2	C13H14F3NO2	C17H24F3N3O	165107	458887	25265318	0.6	1.8	97.6
D5	C13H12F3NO2	C13H14F3NO2	C20H28F3N3O2	0	840060	32619832	0.0	2.5	97.5
D6	C13H12F3NO2	C13H14F3NO2	C16H21F3N2O2	57824	247782	14849718	0.4	1.6	98.0
D7	C13H12F3NO2	C13H14F3NO2	C21H24F3N5O	0	348007	5131707	0.0	6.4	93.6
D8	C13H12F3NO2	C13H14F3NO2	C19H26F3N3O2	0	43900	19149445	0.0	0.2	99.8
D9	C13H12F3NO2	C13H14F3NO2	C17H23F3N2OS	149232	512095	24427744	0.6	2.0	97.4
D10	C13H12F3NO2	C13H14F3NO2	C16H21F3N2OS	327935	567438	6926796	4.2	7.3	88.6

Processed results of desired product, alcohol and ketone area % plotted as a heat map

Desired Product area %

	A	B	C	D	E	F	G	H	I	J
28	63.7	32.6	16.5	74.6	51.5	61.4	60.5	98.0	67.7	37.4
29	93.0	95.0	42.4	87.4	91.6	78.4	86.1	96.1	82.3	70.0
30	88.1	94.1	74.2	99.4	95.2	94.6	97.0	99.4	89.4	95.5
31	93.2	96.3	91.1	97.6	97.5	98.0	93.6	99.8	97.4	88.6

Alcohol area %

	A	B	C	D	E	F	G	H	I	J
1	36.3	67.3	83.3	25.4	48.5	38.4	39.4	2.0	32.2	62.5
2	7.0	5.0	57.5	12.6	8.4	21.5	13.9	3.9	17.7	29.8
3	11.9	5.8	18.9	0.5	4.8	4.1	2.6	0.4	4.9	4.1
4	6.8	3.7	8.1	1.8	2.5	1.6	6.4	0.2	2.0	7.3

Ketone area %

	A	B	C	D	E	F	G	H	I	J
1	0.0	0.1	0.2	0.0	0.0	0.2	0.1	0.0	0.1	0.1
2	0.0	0.0	0.0	0.0	0.0	0.1	0.0	0.0	0.1	0.1
3	0.1	0.0	6.9	0.1	0.0	1.3	0.3	0.2	5.7	0.4
4	0.0	0.0	0.8	0.6	0.0	0.4	0.0	0.0	0.6	4.2

Synthesis of Compounds 5-34

(3*S*,4a*S*,8a*S*)-*N*-(2-methoxyethyl)-3-(thiophen-2-yl)octahydro-2*H*-benzo[*b*][1,4]oxazin-7-amine 5

Thiophene-2-carbaldehyde (56 mg, 0.50 mmol, 1.0 equiv), 2-methoxyethan-1-amine (38 mg, 0.50 mmol, 1.0 equiv) and iSnAP resin **4** were reacted according to the General Procedure to afford the title compound (0.18 mmol, 35% NMR yield, *dr* 56:44).

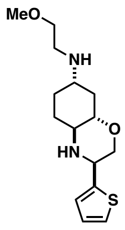

(3*S*,4a*S*,7*S*,8a*S*)-*N*-(2-methoxyethyl)-3-(thiophen-2-yl)octahydro-2*H*-benzo[*b*][1,4]oxazin-7-amine (major, 28 mg, 95 μmol, 19% isolated yield); ^{1}H NMR (500 MHz, $CDCl_3$) δ 7.23 (dd, *J* = 4.9, 1.3 Hz, 1H), 7.03 – 6.94 (m, 2H), 4.35 (ddd, *J* = 10.3, 3.4, 0.7 Hz, 1H), 3.94 (dd, *J* = 11.0, 3.3 Hz, 1H), 3.54 – 3.47 (m, 3H), 3.37 (s, 3H), 3.18 (ddd, *J* = 11.6, 8.8, 4.1 Hz, 1H), 2.86 – 2.81 (m, 2H), 2.64 (m, 2H), 2.20 – 2.14 (m, 1H), 2.05 – 1.86 (m, 3H), 1.77 – 1.68 (m, 1H), 1.47 – 1.35 (m, 1H), 1.35 – 1.18 (m, 2H); ^{13}C NMR (101 MHz, $CDCl_3$) δ 144.4, 126.6, 124.3, 124.1, 78.6, 74.0, 72.1, 60.3, 58.8, 56.3, 55.4, 46.7, 37.2, 31.3, 28.9; HRMS (ESI) *m/z* for $C_{15}H_{24}N_2O_2S$ $[M+H]^+$ calcd. 297.1631, found 297.1632; IR (ν/cm^{-1}, thin film) 2924, 2849, 1681, 1098, 1051, 704.

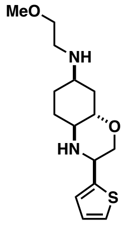

(3*S*,4a*S*,7*R*,8a*S*)-*N*-(2-methoxyethyl)-3-(thiophen-2-yl)octahydro-2*H*-benzo[*b*][1,4]oxazin-7-amine (minor, 16 mg, 54 μmol, 11% isolated yield); ^{1}H NMR (500 MHz, $CDCl_3$) δ 7.23 (dd, *J* = 4.9, 1.4 Hz, 1H), 7.01 – 6.95 (m, 2H), 4.35 (dd, *J* = 10.4, 3.4 Hz, 1H), 3.96 (dd, *J* = 11.0, 3.4 Hz, 1H), 3.58 – 3.49 (m, 4H), 3.38 (s, 3H), 3.09 (m, 1H), 2.83 – 2.73 (m, 2H), 2.65 – 2.59 (m, 1H), 2.08 – 1.67 (m, 5H), 1.62 – 1.46 (m, 3H); ^{13}C NMR (101 MHz, $CDCl_3$) δ 143.8, 126.6, 124.3, 124.0, 76.1, 74.2, 72.1, 61.5, 58.8, 56.4, 53.2, 47.5, 34.6, 28.6, 25.9; HRMS (ESI) *m/z* for $C_{15}H_{24}N_2O_2S$ $[M+H]^+$ calcd. 297.1631, found 297.1633; IR (ν/cm^{-1}, thin film) 2924, 2850, 1443, 1113, 700.

(3*S*,4a*S*,8a*S*)-*N*-cyclopropyl-3-(furan-2-yl)octahydro-2*H*-benzo[*b*][1,4]oxazin-7-amine 6
Furan-2-carbaldehyde (48 mg, 0.50 mmol, 1.0 equiv), cyclopropanamine (29 mg, 0.50 mmol, 1.0 equiv) and iSnAP resin **4** were reacted according to the General Procedure to afford the title compound (0.23 mmol, 46% NMR yield, *dr* 68:32).

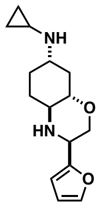

(3*S*,4a*S*,7*S*,8a*S*)-*N*-cyclopropyl-3-(furan-2-yl)octahydro-2*H*-benzo[*b*][1,4]oxazin-7-amine (major, 12 mg, 46 μmol, 9% isolated yield); ^{1}H NMR (500 MHz, $CDCl_3$) δ 7.37 (dd, *J* = 1.8, 0.8 Hz, 1H), 6.33 (dd, *J* = 3.3, 1.8 Hz, 1H), 6.22 (m, 1H), 4.16 (d, *J* = 3.3 Hz, 1H), 4.04 (dd, *J* = 11.0, 3.3 Hz, 1H), 3.66 (t, *J* = 10.8 Hz, 1H), 3.18 (ddd, *J* = 11.5, 8.8, 4.1 Hz, 1H), 2.80 (tt, *J* = 11.4, 3.9 Hz, 1H), 2.59 (ddd, *J* = 11.5, 8.8, 4.0 Hz, 1H), 2.29 (dtd, *J* = 12.0, 4.0, 2.2 Hz, 1H), 2.16 (tt, *J* = 6.7, 3.7 Hz, 1H), 2.07 – 1.98 (m, 1H), 1.89 (br. s, 2H), 1.76 (m, 1H), 1.47 – 1.34 (m, 1H), 1.34 – 1.16 (m, 2H), 0.55 – 0.42 (m, 2H), 0.43 – 0.32 (m, 2H); ^{13}C NMR (101 MHz, $CDCl_3$) δ 153.3, 142.0, 110.1, 105.9, 78.9, 70.8, 59.9, 55.9, 54.1, 37.6, 31.8, 28.9, 28.4, 6.6, 6.3; HRMS (ESI) *m/z* for $C_{15}H_{22}N_2O_2$ $[M+H]^+$ calcd. 263.1754, found 263.1752; IR (ν/cm^{-1}, thin film) 2924, 2856, 1448, 1358, 1097, 737.

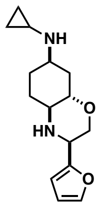

(3*S*,4a*S*,7*R*,8a*S*)-*N*-cyclopropyl-3-(furan-2-yl)octahydro-2*H*-benzo[*b*][1,4]oxazin-7-amine (minor, isolated 16 mg, 61 μmol, 12% isolated yield); ^{1}H NMR (400 MHz, $CDCl_3$) δ 7.36 (dd, *J* = 1.8, 0.8 Hz, 1H), 6.32 (dd, *J* = 3.2, 1.8 Hz, 1H), 6.24 – 6.18 (m, 1H), 4.15 (dd, *J* = 10.6, 3.5 Hz, 1H), 4.03 (dd, *J* = 11.0, 3.3 Hz, 1H), 3.64 (t, *J* = 10.8 Hz, 1H), 3.45 (ddd, *J* = 11.8, 8.9, 4.2 Hz, 1H), 3.23 (m, 1H), 2.63 – 2.54 (m, 1H), 2.13 – 2.03 (m, 2H), 1.93 – 1.83 (m, 1H), 1.73 (s, 1H), 1.69 – 1.43 (m, 5H), 0.51 – 0.41 (m, 2H), 0.39 – 0.29 (m, 2H); ^{13}C NMR (101 MHz, $CDCl_3$) δ 153.4, 141.9, 110.1, 105.8, 76.6, 71.2, 60.5, 54.2, 53.7, 35.0, 29.1, 28.9, 26.0, 6.3, 6.1; HRMS (ESI) *m/z* for $C_{15}H_{22}N_2O_2$ $[M+H]^+$ calcd. 263.1754, found 263.1755; IR (ν/cm^{-1}, thin film) 2924, 2856, 1673, 1340, 1097, 1010, 736.

(3*R*,4a*S*,8a*S*)-*N*-butyl-3-(*p*-tolyl)octahydro-2*H*-benzo[*b*][1,4]oxazin-7-amine 7
4-Methylbenzaldehyde (60 mg, 0.50 mmol, 1.0 equiv), butan-1-amine (37 mg, 0.50 mmol, 1.0 equiv) and iSnAP resin **4** were reacted according to the General Procedure to afford the title compound (0.10 mmol, 20% NMR yield, *dr* 72:28).

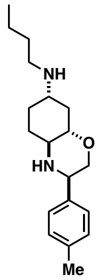

(3*R*,4a*S*,7*S*,8a*S*)-*N*-butyl-3-(*p*-tolyl)octahydro-2*H*-benzo[*b*][1,4]oxazin-7-amine (major, 18 mg, 60 μmol, 12% isolated yield); ^{1}H NMR (400 MHz, $CDCl_3$) δ 7.29 (d, *J* = 5.8 Hz, 2H), 7.15 (d, *J* = 7.8 Hz, 2H), 4.01 (dd, *J* = 10.3, 3.2 Hz, 1H), 3.86 (dd, *J* = 11.0, 3.3 Hz, 1H), 3.45 (t, *J* = 10.7 Hz, 1H), 3.18 (ddd, *J* = 11.6, 8.7, 4.1 Hz, 1H), 2.74 – 2.65 (m, 3H), 2.62 (ddd, *J* = 11.4, 8.8, 4.1 Hz, 1H), 2.35 (s, 3H), 2.24 – 2.16 (m, 1H), 2.04 – 1.95 (m, 1H), 1.79 – 1.68 (m, 1H), 1.57 – 1.45 (m, 2H), 1.45 – 1.25 (m, 5H), 0.94 (t, *J* = 7.3 Hz, 3H), exchangeable 2 x NH not observed; ^{13}C NMR (101 MHz, $CDCl_3$) δ 137.4, 137.3, 129.1 (2C), 127.1 (2C), 78.5, 73.7, 60.7, 60.4, 55.5, 46.8, 37.1, 32.1, 31.1, 29.0, 21.1, 20.5, 14.0; HRMS (ESI) *m/z* for $C_{19}H_{30}N_2O$ $[M+H]^+$ calcd. 303.2431, found 303.2429, IR (ν/cm^{-1}, thin film) 2923, 2854, 2361, 1663, 1451, 1131, 764, 750.

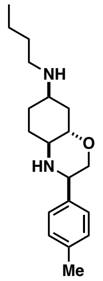

(3*R*,4a*S*,7*R*,8a*S*)-*N*-butyl-3-(*p*-tolyl)octahydro-2*H*-benzo[*b*][1,4]oxazin-7-amine (minor, 12 mg, 40 μmol, 8% isolated yield); ^{1}H NMR (400 MHz, $CDCl_3$) δ 7.29 (d, *J* = 8.1 Hz, 2H), 7.15 (d, *J* = 7.9 Hz, 2H), 4.01 (dd, *J* = 10.4, 3.3 Hz, 1H), 3.87 (dd, *J* = 11.1, 3.3 Hz, 1H), 3.55 – 3.44 (m, 2H), 3.17 – 3.09 (m, 1H), 2.72 – 2.57 (m, 3H), 2.35 (s, 3H), 2.08 – 1.99 (m, 1H), 1.88 – 1.78 (m, 1H), 1.77 – 1.67 (m, 1H), 1.64 – 1.45 (m, 6H), 1.44 – 1.31 (m, 2H), 0.95 (t, *J* = 7.3 Hz, 3H), exchangeable 1 x NH not observed; ^{13}C NMR (101 MHz, $CDCl_3$) δ 137.4, 137.3, 129.1 (2C), 127.1 (2C), 76.0, 74.0, 61.1, 60.7, 53.4, 47.3, 34.4, 32.0, 28.5, 26.0, 21.1, 20.5, 14.0; HRMS (ESI) *m/z* for $C_{19}H_{30}N_2O$ $[M+H]^+$ calcd. 303.2431, found 303.2431; IR (ν/cm^{-1}, thin film) 2923, 2854, 1681, 1455, 1201, 1094.

(3*R*,4a*S*,8a*S*)-*N*-(but-3-en-1-yl)-3-(3,4,5-trifluorophenyl)octahydro-2*H*-benzo[*b*][1,4]oxazin-7-amine 8

3,4,5-Trifluorobenzaldehyde (80 mg, 0.50 mmol, 1.0 equiv), but-3-en-1-amine (36 mg, 0.50 mmol, 1.0 equiv) and iSnAP resin **4** were reacted according to the General Procedure to afford the title compound (0.18 mmol, 35% NMR yield, *dr* 60:40).

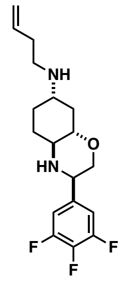

(3*R*,4a*S*,7*S*,8a*S*)-*N*-(but-3-en-1-yl)-3-(3,4,5-trifluorophenyl)octahydro-2*H*-benzo[*b*][1,4]oxazin-7-amine (major, 30 mg, 88 μmol, 18% isolated yield); ^{1}H NMR (500 MHz, $CDCl_3$) δ 7.12 – 7.00 (m, 2H), 5.79 (ddt, *J* = 17.1, 10.2, 6.8 Hz, 1H), 5.16 – 5.03 (m, 2H), 3.98 (dd, *J* = 10.2, 3.3 Hz, 1H), 3.81 (dd, *J* = 11.1, 3.3 Hz, 1H), 3.31 (dd, *J* = 11.1, 10.2 Hz, 1H), 3.14 (ddd, *J* = 11.5, 8.8, 4.1 Hz, 1H), 2.74 (m, 2H), 2.67 (tt, *J* = 11.3, 3.9 Hz, 1H), 2.60 (ddd, *J* = 11.4, 8.8, 4.1 Hz, 1H), 2.31 – 2.24 (m, 2H), 2.16 (dtd, *J* = 11.9, 4.0, 2.2 Hz, 1H), 2.00 – 1.92 (m, 1H), 1.87 – 1.52 (m, 3H), 1.45 – 1.35 (m, 1H), 1.32 – 1.18 (m, 2H); ^{13}C NMR (126 MHz, $CDCl_3$) δ 151.2 (ddd, *J* = 249.9, 9.9, 3.9 Hz, 2C), 139.0 (dt, *J* = 251.2, 15.4 Hz), 136.8 (td, *J* = 7.0, 6.9, 4.4 Hz), 136.2, 116.6, 111.1 (dd, *J* = 16.5, 5.1 Hz, 2C), 78.6, 73.4, 60.0, 59.7, 55.3, 46.1, 37.2, 34.3, 31.3, 29.0; HRMS (ESI) *m/z* for $C_{18}H_{23}F_3N_2O$ $[M+H]^+$ calcd. 341.1834, found 342.1835; IR (ν/cm^{-1}, thin film) 2928, 2853, 1527, 1445, 1349, 1095, 1041, 711.

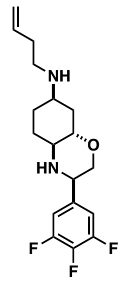

(3*R*,4a*S*,7*R*,8a*S*)-*N*-(but-3-en-1-yl)-3-(3,4,5-trifluorophenyl)octahydro-2*H*-benzo[*b*][1,4]oxazin-7-amine (minor, 17 mg, 50 μmol, 10% isolated yield); ^{1}H NMR (500 MHz, $CDCl_3$) δ 7.12 – 7.03 (m, 2H), 5.82 (ddt, *J* = 17.1, 10.2, 6.8 Hz, 1H), 5.16 – 5.03 (m, 2H), 3.98 (dd, *J* = 10.3, 3.3 Hz, 1H), 3.82 (dd, *J* = 11.0, 3.3 Hz, 1H), 3.50 (ddd, *J* = 11.8, 8.8, 4.2 Hz, 1H), 3.35 (dd, *J* = 11.0, 10.3 Hz, 1H), 3.11 (m, 1H), 2.69 (td, *J* = 6.9, 2.2 Hz, 2H), 2.61 (ddd, *J* = 11.4, 8.9, 4.1 Hz, 1H), 2.28 (qt, *J* = 6.9, 1.4 Hz, 2H), 1.99 (ddt, *J* = 13.2, 4.5, 2.5 Hz, 1H), 1.83 – 1.69 (m, 3H), 1.62 – 1.46 (m, 4H), ^{13}C NMR (126 MHz, $CDCl_3$) δ 151.2 (ddd, *J* = 249.9, 9.9, 3.8 Hz, 2C), 139.1 (dt, *J* = 251.1, 15.6 Hz), 136.9 (td, *J* = 6.7, 4.4 Hz), 136.4, 116.4, 111.1 (dd, *J* = 16.6, 5.0 Hz, 2C), 76.1, 73.6, 60.7, 59.8, 53.0, 46.5, 34.6, 34.2, 28.5, 26.0; HRMS (ESI) *m/z* for $C_{18}H_{23}F_3N_2O$ $[M+H]^+$ calcd. 341.1834, found 342.1837; IR (ν/cm^{-1}, thin film) 2928, 2854, 2360, 1620, 1527, 1443, 1350, 1094, 1034, 706.

(3*R*,4a*S*,7a*S*)-3-(4-(1*H*-1,2,4-triazol-1-yl)phenyl)-*N*-cyclopropyloctahydrocyclopenta[*b*][1,4]oxazin-6-amine 9

4-(1*H*-1,2,4-triazol-1-yl)benzaldehyde (87 mg, 0.50 mmol, 1.0 equiv), cyclopropanamine (29 mg, 0.50 mmol, 1.0 equiv) and iSnAP resin **3** were reacted according to the General Procedure to afford the title compound (0.14 mmol, 28% NMR yield, *dr* 50:50).

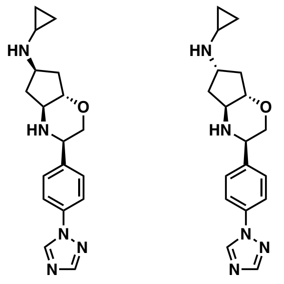

A B

(3*R*,4a*S*,7a*S*)-3-(4-(1*H*-1,2,4-triazol-1-yl)phenyl)-*N*-cyclopropyloctahydrocyclopenta[*b*][1,4]oxazin-6-amine (mixture, 20 mg, 62 *μ*mol, 12% isolated yield); diastereomer A ^{1}H NMR (500 MHz, $CDCl_3$) δ 8.53 (s, 1H), 8.08 (s, 1H), 7.68 – 7.60 (m, 2H), 7.60 – 7.52 (m, 2H), 4.12 – 4.01 (m, 1H), 3.92 – 3.83 (m, 1H), 3.57 – 3.48 (m, 1H), 3.49 – 3.41 (m, 2H), 2.80 (ddd, *J* = 12.0, 9.1, 6.4 Hz, 1H), 2.29 (ddd, *J* = 12.1, 7.6, 6.4 Hz, 1H), 2.25 (br. s, 2H), 2.13 (tt, *J* = 6.7, 3.2 Hz, 1H), 1.99 – 1.83 (m, 2H), 1.37 (td, *J* = 12.1, 7.8 Hz, 1H), 0.53 – 0.43 (m, 2H), 0.46 – 0.36 (m, 2H); ^{13}C NMR (126 MHz, $CDCl_3$) δ 152.7, 140,9m 140.4, 136.6, 128.9 (2C), 120.2 (2C), 81.3, 70.25, 60.8, 60.7, 53.7, 36.2, 34.2, 29.1, 6.3 (2C); diastereomer B ^{1}H NMR (500 MHz, $CDCl_3$) δ 8.53 (s, 1H), 8.08 (s, 1H), 7.68 – 7.60 (m, 2H), 7.60 – 7.52 (m, 2H), 4.12 – 4.01 (m, 1H), 3.92 – 3.83 (m, 1H), 3.49 – 3.41 (m, 2H), 3.32 (ddd, *J* = 11.4, 9.2, 6.7 Hz, 1H), 3.07 – 2.97 (m, 1H), 2.40 (ddd, *J* = 12.1, 7.6, 6.7 Hz, 1H), 2.25 (br. s, 2H), 2.13 (tt, *J* = 6.7, 3.2 Hz, 1H), 1.86 – 1.70 (m, 2H), 1.51 (td, *J* = 11.7, 7.2 Hz, 1H), 0.53 – 0.43 (m, 2H), 0.46 – 0.36 (m, 2H); ^{13}C NMR (126 MHz, $CDCl_3$) δ 152.7, 140,9, 140.4, 136.6, 128.9 (2C), 120.2 (2C), 81.1, 70.25, 60.7, 60.1, 53.7, 36.0, 34.2, 29.1, 6.3 (2C); HRMS (ESI) *m/z* for $C_{18}H_{23}N_5O$ $[M+H]^+$ calcd. 326.1975, found 326.1977; IR (ν/cm^{-1}, thin film) 2957, 2850, 1338, 1124, 982, 836, 729, 546 cm; [α]^{24} -27.8° (c 1.00, CH_2Cl_2).

(3*R*,4a*S*,7a*S*)-*N*-(4-fluorobenzyl)-3-phenyloctahydrocyclopenta[*b*][1,4]oxazin-6-amine 10

Benzaldehyde (53 mg, 0.50 mmol, 1.0 equiv), (4-fluorophenyl)methanamine (63 mg, 0.50 mmol, 1.0 equiv) and iSnAP resin **3** were reacted according to the General Procedure to afford the title compound (0.14 mmol, 28% NMR yield, *dr* 50:50).

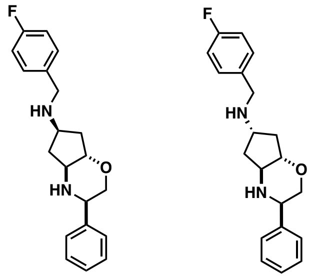

A **B**

(3*R*,4a*S*,7a*S*)-*N*-(4-fluorobenzyl)-3-phenyloctahydrocyclopenta[*b*][1,4]oxazin-6-amine (mixture, 14 mg, 43 *µ*mol, 9% isolated yield); diastereomer A ^{1}H NMR (400 MHz, $CDCl_3$) δ 7.37 – 7.30 (m, 2H), 7.29 – 7.17 (m, 5H), 6.97 – 6.89 (m, 2H), 3.93 (ddd, *J* = 11.8, 10.5, 3.4 Hz, 1H), 3.82 (ddd, *J* = 11.8, 10.5, 3.4 Hz, 1H), 3.66 (d, *J* = 1.6 Hz, 2H), 3.53 – 3.46 (m, 1H), 3.46 – 3.37 (m, 1H), 3.34 – 3.20 (m, 1H), 2.72 (ddd, *J* = 11.9, 9.1, 6.5 Hz, 1H), 2.17 (ddd, *J* = 12.0, 7.6, 6.5 Hz, 1H), 1.90 (br s, 2H), 1.83 – 1.73 (m, 2H), 1.32 (td, *J* = 12.0, 7.5 Hz, 1H); ^{13}C NMR (101 MHz, $CDCl_3$) δ 162.0 (d, *J* = 244.9 Hz), 139.0, 129.8 (d, *J* = 8.0 Hz, 2C), 128.5 (2C), 127.4 (2C), 127.4 (2C), 115.3 (d, *J* = 21.3 Hz, 2C), 81.1, 74.3, 61.2, 61.1, 52.7, 51.3, 36.0, 34.3; diastereomer B ^{1}H NMR (400 MHz, $CDCl_3$) δ 7.37 – 7.30 (m, 2H), 7.29 – 7.17 (m, 5H), 6.97 – 6.89 (m, 2H), 3.82 (ddd, *J* = 11.8, 10.5, 3.4 Hz, 1H), 3.9 (ddd, *J* = 11.8, 10.5, 3.4 Hz, 1H), 3.66 (d, *J* = 1.6 Hz, 2H), 3.53 – 3.46 (m, 1H), 3.34 – 3.20 (m, 2H), 2.99 (ddd, *J* = 11.3, 9.3, 8.2 Hz, 1H), 2.29 (ddd, *J* = 12.0, 7.6, 6.8 Hz, 1H), 1.9 (br s, 2H), 1.71 – 1.64 (m, 2H), 1.43 (td, *J* = 11.6, 7.0 Hz, 1H); ^{13}C NMR (101 MHz, $CDCl_3$) δ 162.0 (d, *J* = 244.9 Hz), 139.0, 129.8 (dd, *J* = 8.0 Hz, 2C), 128.5 (2C), 127.4 (2C), 127.4 (2C), 115.3 (d, *J* = 21.3 Hz, 2C), 81.1, 74.3, 61.2, 60.4, 52.7, 51.3, 36.0, 34.3; HRMS (ESI) *m/z* for $C_{20}H_{23}FN_2O$ $[M+Na]^+$ calcd. 349.1687, found 349.1684; IR (ν/cm^{-1}, thin film) 2923, 2854, 1664, 1450, 1131, 1098, 813; $[\alpha]^{25}$ -33.2° (c 0.85 CH_2Cl_2).

(3*R*,4a*S*,7a*S*)-3-(4-fluorophenyl)-*N*-(2-(methylthio)ethyl)octahydrocyclopenta[*b*][1,4]oxazin-6-amine 11

4-Fluorobenzaldehyde (62 mg, 0.50 mmol, 1.0 equiv), 2-(methylthio)ethan-1-amine (46 mg, 0.50 mmol, 1.0 equiv) and iSnAP resin 3 were reacted according to the General Procedure to afford the title compound (0.11 mmol, 23% NMR yield, *dr* 60:40).

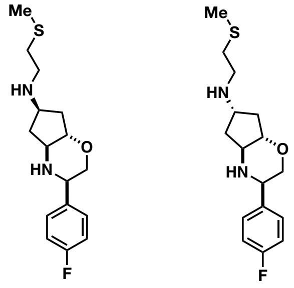

(3*R*,4a*S*,7a*S*)-3-(4-fluorophenyl)-*N*-(2-(methylthio)ethyl)octahydrocyclopenta[*b*][1,4]oxazin-6-amine (mixture, 22 mg, 71 *µ*mol, 14% isolated yield); diastereomer A ^{1}H NMR (500 MHz, $CDCl_3$) δ 7.41 – 7.33 (m, 2H), 7.04 – 6.95 (m, 2H), 3.97 (td, *J* = 10.7, 3.4 Hz, 1H), 3.84 (ddd, *J* = 11.4, 3.5, 0.7 Hz, 1H), 3.56 – 3.53 (m, 1H), 3.52 – 3.49 (m, 2H), 3.41 (dt, *J* = 11.4, 10.3 Hz, 1H), 3.36 (s, 3H), 3.34 – 3.26 (m, 1H), 2.82 – 2.70 (m, 3H), 2.34 (ddd, *J* = 11.9, 7.6, 6.7 Hz, 1H), 1.98 (br s, 2H), 1.88 – 1.79 (m, 2H), 1.34 (td, *J* = 12.0, 7.6 Hz, 1H); ^{13}C NMR (126 MHz, $CDCl_3$) δ 162.3 (d, *J* = 245.8 Hz), 135.8 (d, *J* = 3.2 Hz), 129.0 (d, *J* = 8.0 Hz, 2C), 115.3 (d, *J* = 21.2 Hz, 2C), 81.1, 74.4, 74.2, 71.9, 60.9, 60.3, 58.8, 53.5, 47.6, 35.9; diastereomer B ^{1}H NMR (500 MHz, $CDCl_3$) δ 7.41 – 7.33 (m, 2H), 7.04 – 6.95 (m, 2H), 3.97 (td, *J* = 10.7, 3.4 Hz, 1H), 3.84 (ddd, *J* = 11.4, 3.5, 0.7 Hz, 1H), 3.52 – 3.49 (m, 2H), 3.41 (dt, *J* = 11.4, 10.3 Hz, 1H), 3.36 (s, 3H), 3.34 – 3.26 (m, 2H), 3.03 (ddd, *J* = 11.3, 9.3, 8.2 Hz, 1H), 2.82 – 2.70 (m, 2H), 2.22 (ddd, *J* = 12.0, 7.5, 6.4 Hz, 1H), 1.98 (br s, 2H), 1.76 – 1.67 (m, 2H), 1.47 (td, *J* = 11.6, 7.1 Hz, 1H); ^{13}C NMR (126 MHz, $CDCl_3$) δ 162.3 (d, *J* = 245.8 Hz), 135.8 (d, *J* = 3.2 Hz), 129.0 (d, *J* = 8.0 Hz, 2C), 115.3 (d, *J* = 21.2 Hz, 2C), 81.0, 74.4, 74.2 71.9, 60.5, 60.3, 58.8, 53.5, 47.6, 34.3; HRMS (ESI) *m/z* for $C_{16}H_{23}FN_2OS$ $[M+H]^+$ 311.1558, calcd. found 311.1586; IR (ν/cm^{-1}, thin film) 2924, 2850, 1677, 1509, 1220, 1124, 835; $[\alpha]^{25}$ -25.9° (c 0.85 CH_2Cl_2).

(3*R*,4a*S*,7a*S*)-*N*-(2-methoxyethyl)-3-(quinolin-3-yl)octahydrocyclopenta[*b*][1,4]oxazin-6-amine 12

Quinoline-3-carbaldehyde (79 mg, 0.50 mmol, 1.0 equiv), 2-methoxyethan-1-amine (75 mg, 0.50 mmol, 1.0 equiv) and iSnAP resin **3** were reacted according to the General Procedure to afford the title compound (0.20 mmol, 39% NMR yield, *dr* 59:41).

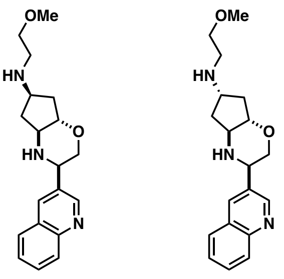

A B

(3*R*,4a*S*,7a*S*)-*N*-(2-methoxyethyl)-3-(quinolin-3-yl)octahydrocyclopenta[*b*][1,4]oxazin-6-amine (mixture, 18 mg, 55 μmol, 11% isolated yield); diastereomer A ^{1}H NMR (500 MHz, $CDCl_3$) δ 8.93 (t, *J* = 2.5 Hz, 1H), 8.93 (t, *J* = 2.5 Hz, 1H), 8.11 (dt, *J* = 8.5, 1.0 Hz, 1H), 7.85 – 7.81 (m, 1H), 7.71 (ddd, *J* = 8.4, 6.9, 1.4 Hz, 1H), 7.56 (m, 1H), 4.24 (td, *J* = 10.3, 3.4 Hz, 1H), 3.99 (ddd, *J* = 11.5, 3.5, 0.7 Hz, 1H), 3.65 – 3.59 (m, 1H), 3.59 – 3.51 (m, 3H), 3.44 – 3.33 (m, 4H), 2.88 (ddd, *J* = 11.9, 9.1, 6.5 Hz, 1H), 2.83 – 2.74 (m, 2H), 2.30 (ddd, *J* = 12.0, 7.5, 6.5 Hz, 1H), 2.02 (br s, 2H), 1.94 – 1.87 (m, 2H), 1.43 (td, *J* = 12.0, 7.6 Hz, 1H); ^{13}C NMR (126 MHz, $CDCl_3$) δ 150.5, 148.0, 134.2, 132.8, 129.4, 129.3, 128.0, 127.7, 126.8, 81.1, 74.1, 71.9, 60.9, 58.9, 58.8, 53.5, 47.3, 35.9, 34.3; diastereomer B ^{1}H NMR (500 MHz, $CDCl_3$) δ 8.93 (t, *J* = 2.5 Hz, 1H), 8.93 (t, *J* = 2.5 Hz, 1H), 8.11 (dt, *J* = 8.5, 1.0 Hz, 1H), 7.85 – 7.81 (m, 1H), 7.71 (ddd, *J* = 8.4, 6.9, 1.4 Hz, 1H), 7.56 (m, 1H), 4.24 (td, *J* = 10.3, 3.4 Hz, 1H), 3.99 (ddd, *J* = 11.5, 3.5, 0.7 Hz, 1H), 3.59 – 3.51 (m, 3H), 3.44 – 3.33 (m, 5H), 3.13 (q, *J* = 9.5 Hz, 1H), 2.83 – 2.74 (m, 2H), 2.39 (ddd, *J* = 12.0, 7.5, 6.7 Hz, 1H), 2.02 (br s, 2H), 1.82 – 1.78 (m, 2H), 1.53 (td, *J* = 11.7, 7.1 Hz, 1H); ^{13}C NMR (126 MHz, $CDCl_3$) δ 150.5, 148.0, 134.2, 132.8, 129.4, 129.3, 128.0, 127.7, 126.8, 81.1, 74.1, 71.9, 60.3, 58.9, 58.8, 53.5, 47.3, 35.9, 34.3; HRMS (ESI) *m/z* for $C_{19}H_{25}N_3O_2$ $[M+H]^+$ calcd. 328.2020, found 328.2020; IR (ν/cm^{-1}, thin film) 2932, 2843, 1446, 1326, 1123, 788, 754; $[\alpha]^{25}$ -53.8° (c 0.05 CH_2Cl_2).

(3*R*)-3-(benzo[*d*][1,3]dioxol-5-yl)-*N*-cyclobutyl-1-oxa-4-azaspiro[5.5]undecan-9-amine 13

Benzo[*d*][1,3]dioxole-5-carbaldehyde (75 mg, 0.50 mmol, 1.0 equiv), cyclobutanamine (36 mg, 0.50 mmol, 1.0 equiv) and iSnAP resin **1** were reacted according to the General Procedure to afford the title compound (0.09 mmol, 18% NMR yield, *dr* 70:30).

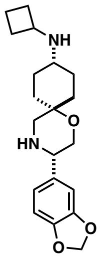

(3*S*,6*s*,9*R*)-3-(benzo[*d*][1,3]dioxol-5-yl)-*N*-cyclobutyl-1-oxa-4-azaspiro[5.5]undecan-9-amine (major, 14 mg, 41 μmol, 8% isolated yield); ^{1}H NMR (400 MHz, $CDCl_3$) δ 6.94 (d, *J* = 1.6 Hz, 1H), 6.85 (dd, *J* = 8.1, 1.7 Hz, 1H), 6.77 (d, *J* = 7.9 Hz, 1H), 5.99 – 5.90 (m, 2H), 3.78 (dd, *J* = 10.3, 3.8 Hz, 1H), 3.59 – 3.41 (m, 3H), 2.92 – 2.72 (m, 4H), 2.61 (tt, *J* = 10.9, 4.0 Hz, 1H), 2.30 – 2.16 (m, 2H), 1.93 – 1.78 (m, 2H), 1.78 – 1.55 (m, 7H), 1.46 – 1.20 (m, 2H), 1.18 – 1.05 (m, 1H); ^{13}C NMR (126 MHz, $CDCl_3$) δ 147.6, 146.9, 134.8, 120.3, 108.2, 107.6, 100.9, 69.9, 67.0, 60.5, 56.0, 55.3, 51.5, 35.1, 31.4, 27.4, 27.1, 26.9 (2C), 15.1; HRMS (ESI) *m/z* for $C_{20}H_{28}N_2O_3$ $[M+H]^+$ calcd. 345.2173, measured 345.2174, IR (ν/cm^{-1} thin film) 2935, 2361, 1672, 1485, 1247, 1038, 931, 729.

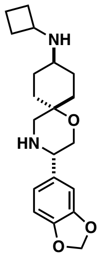

(3*S*,6*r*,9*S*)-3-(benzo[*d*][1,3]dioxol-5-yl)-*N*-cyclobutyl-1-oxa-4-azaspiro[5.5]undecan-9-amine (minor, 6 mg, 17 μmol, 3% isolated yield); ^{1}H NMR (400 MHz, $CDCl_3$) δ 6.95 (d, *J* = 1.7 Hz, 1H), 6.86 (dd, *J* = 8.3, 1.6 Hz, 1H), 6.80 – 6.74 (m, 1H), 5.95 (s, 2H), 3.79 (dd, *J* = 10.2, 3.8 Hz, 1H), 3.66 – 3.59 (m, 2H), 3.41 (p, *J* = 7.6 Hz, 1H), 3.25 (d, *J* = 11.7 Hz, 1H), 2.75 – 2.66 (m, 2H), 2.63 – 2.54 (m, 1H), 2.31 – 2.19 (m, 3H), 1.96 – 1.59 (m, 8H), 1.60 – 1.44 (m, 2H), 1.41 – 1.25 (m, 2H); ^{13}C NMR (126 MHz, $CDCl_3$) δ 147.6, 146.9, 134.8, 120.3, 108.1, 107.6, 101.0, 71.7, 67.6, 60.7, 53.8, 52.3, 51.8, 50.8, 34.8, 31.5, 28.7, 28.3, 26.6, 15.0; HRMS (ESI) *m/z* for $C_{20}H_{28}N_2O_3$ $[M+H]^+$ calcd. 345.2173, measured 345.2175; IR (ν/cm^{-1} thin film) 2935, 1504, 1485, 1439, 1246, 1089, 1038, 808.

3-((3*R*)-9-((pyridin-2-ylmethyl)amino)-1-oxa-4-azaspiro[5.5]undecan-3-yl)benzonitrile 14
3-Formylbenzonitrile (66 mg, 0.50 mmol, 1.0 equiv), pyridin-2-ylmethanamine (54 mg, 0.50 mmol, 1.0 equiv) and iSnAP resin **1** were reacted according to the General Procedure to afford the title compound (0.22 mmol, 44% NMR yield, *dr* 64:36).

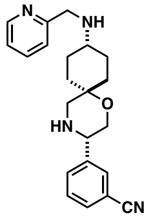

3-((3*S*,6*s*,9*R*)-9-((pyridin-2-ylmethyl)amino)-1-oxa-4-azaspiro[5.5]undecan-3-yl)benzonitrile (major, 46 mg, 127 μmol, 25% isolated yield); ^{1}H NMR (400 MHz, $CDCl_3$) δ 8.55 (ddd, *J* = 4.9, 1.9, 1.0 Hz, 1H), 7.79 – 7.73 (m, 1H), 7.69 – 7.60 (m, 2H), 7.57 (dt, *J* = 7.8, 1.5 Hz, 1H), 7.43 (t, *J* = 7.7 Hz, 1H), 7.34 (d, *J* = 7.8 Hz, 1H), 7.18 (ddd, *J* = 7.5, 4.9, 1.2 Hz, 1H), 4.04 (s, 2H), 3.90 (dd, *J* = 10.2, 3.7 Hz, 1H), 3.59 (dd, *J* = 11.6, 3.7 Hz, 1H), 3.48 (dd, *J* = 11.5, 10.3 Hz, 1H), 2.89 – 2.76 (m, 3H), 2.73 – 2.63 (m, 1H), 1.89 – 1.78 (m, 2H), 1.75 – 1.64 (m, 2H), 1.50 – 1.30 (m, 2H), 1.16 (td, *J* = 14.0, 3.5 Hz, 1H), exchangeable NH x 2 not observed; ^{13}C NMR (101 MHz, $CDCl_3$) δ 158.6, 149.2, 142.4, 136.6, 131.8, 131.3, 130.8, 129.2, 122.4, 122.2, 118.7, 112.6, 70.3, 66.6, 60.0, 56.7, 55.5, 51.7, 34.9, 27.1, 26.9, 26.8; HRMS (ESI) *m/z* for $C_{22}H_{26}N_4O$ [M+Na]$^+$ calcd. 385.1999, found 385.1995; IR (ν/cm^{-1}, thin film) 2934, 2228, 1672, 1591, 1433, 1091, 730.

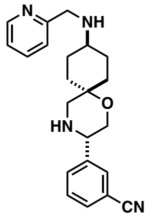

3-((3*S*,6*r*,9*S*)-9-((pyridin-2-ylmethyl)amino)-1-oxa-4-azaspiro[5.5]undecan-3-yl)benzonitrile (minor, 19 mg, 52 μmol, 10% isolated yield); ^{1}H NMR (400 MHz, $CDCl_3$) δ 8.58 (ddd, *J* = 4.9, 1.8, 0.9 Hz, 1H), 7.81 – 7.74 (m, 1H), 7.70 – 7.62 (m, 2H), 7.58 (dt, *J* = 7.7, 1.4 Hz, 1H), 7.44 (t, *J* = 7.7 Hz, 1H), 7.32 (d, *J* = 7.8 Hz, 1H), 7.19 (ddd, *J* = 7.6, 4.9, 1.2 Hz, 1H), 3.97 (s, 2H), 3.92 (dd, *J* = 9.2, 4.8 Hz, 1H), 3.66 – 3.53 (m, 2H), 3.25 (d, *J* = 11.7 Hz, 1H), 2.78 – 2.70 (m, 2H), 2.57 – 2.49 (m, 1H), 1.99 – 1.90 (m, 2H), 1.79 – 1.71 (m, 1H), 1.62 (ddd, *J* = 13.6, 10.7, 3.9 Hz, 1H), 1.53 (ddd, *J* = 13.2, 11.0, 3.9 Hz, 1H), 1.46 – 1.31 (m, 2H), exchangeable NH x 2 not observed; ^{13}C NMR (101 MHz, $CDCl_3$) δ 159.3, 149.3, 142.4, 136.5, 131.8, 131.4, 130.8, 129.2, 122.4, 122.0, 118.8, 112.6, 72.0, 67.2, 60.2, 55.4, 52.6, 52.3, 33.9, 28.4, 28.1, 26.3; HRMS (ESI) *m/z* for $C_{22}H_{26}N_4O$ [M+H]$^+$ calcd. 363.2179, found 363.2178; IR (ν/cm^{-1}, thin film) 2933, 2228, 1671, 1590, 1433, 1087, 730.

(3*R*)-*N*-(furan-2-ylmethyl)-3-(naphthalen-2-yl)-1-oxa-4-azaspiro[5.5]undecan-9-amine 15
2-Naphthaldehyde (78 mg, 0.50 mmol, 1.0 equiv), furan-2-ylmethanamine (48 mg, 0.50 mmol, 1.0 equiv) and iSnAP resin 1 were reacted according to the General Procedure to afford the title compound (0.22 mmol, 44% NMR yield, *dr* 60:40).

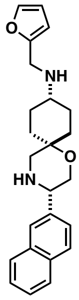

(3*S*,6*s*,9*R*)-*N*-(furan-2-ylmethyl)-3-(naphthalen-2-yl)-1-oxa-4-azaspiro[5.5]undecan-9-amine (major, 25 mg, 66 μmol, 13% isolated yield); ^{1}H NMR (500 MHz, $CDCl_3$) δ 7.91 – 7.87 (m, 1H), 7.86 – 7.81 (m, 3H), 7.52 (dd, *J* = 8.5, 1.8 Hz, 1H), 7.50 – 7.46 (m, 2H), 7.38 (dd, *J* = 1.9, 0.9 Hz, 1H), 6.33 (dd, *J* = 3.2, 1.8 Hz, 1H), 6.23 – 6.18 (m, 1H), 4.05 (dd, *J* = 10.2, 3.9 Hz, 1H), 3.88 (s, 2H), 3.74 – 3.62 (m, 2H), 2.97 – 2.83 (m, 3H), 2.58 (tt, *J* = 10.9, 3.9 Hz, 1H), 1.86 (s, 2H), 1.83 – 1.75 (m, 2H), 1.75 – 1.68 (m, 1H), 1.68 – 1.58 (m, 1H), 1.46 – 1.36 (m, 2H), 1.20 (td, *J* = 13.9, 3.5 Hz, 1H), ^{13}C NMR (101 MHz, $CDCl_3$) δ 154.4, 141.7, 138.3, 133.4, 133.1, 128.0, 127.8, 127.7, 126.1, 125.8, 125.7, 125.5, 110.1, 106.5, 70.3, 66.9, 60.9, 56.1, 55.9, 43.4, 35.2, 27.7, 27.5, 27.0; HRMS (ESI) *m/z* for $C_{24}H_{28}N_2O_2$ [M+H]$^+$ calcd. 377.2224, found 377.2225; IR (ν/cm^{-1}, thin film) 2932, 2855, 1440, 1086, 1065, 731, 479.

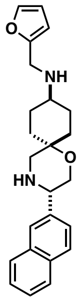

(3*S*,6*r*,9*S*)-*N*-(furan-2-ylmethyl)-3-(naphthalen-2-yl)-1-oxa-4-azaspiro[5.5]undecan-9-amine (minor, 15 mg, 40 μmol, 8% isolated yield); ^{1}H NMR (500 MHz, $CDCl_3$) δ 7.92 – 7.88 (m, 1H), 7.88 – 7.79 (m, 3H), 7.53 (dd, *J* = 8.5, 1.7 Hz, 1H), 7.51 – 7.44 (m, 2H), 7.39 (dd, *J* = 1.9, 0.9 Hz, 1H), 6.35 (dd, *J* = 3.2, 1.9 Hz, 1H), 6.23 – 6.20 (m, 1H), 4.05 (dd, *J* = 10.3, 3.8 Hz, 1H), 3.86 (s, 2H), 3.78 (dd, *J* = 11.5, 10.3 Hz, 1H), 3.70 (dd, *J* = 11.5, 3.8 Hz, 1H), 3.30 (d, *J* = 11.7 Hz, 1H), 2.81 (dd, *J* = 11.8, 1.0 Hz, 1H), 2.71 (tt, *J* = 8.5, 3.9 Hz, 1H), 2.64 – 2.57 (m, 1H), 1.96 – 1.89 (m, 4H), 1.80 – 1.74 (m, 1H), 1.70 – 1.63 (m, 1H), 1.55 (ddd, *J* = 13.2, 11.0, 4.1 Hz, 1H), 1.44 – 1.28 (m, 2H); ^{13}C NMR (101 MHz, $CDCl_3$) δ 154.6, 141.8, 138.3, 133.4, 133.1, 128.0, 127.8, 127.7, 126.1, 125.8, 125.7, 125.5, 110.1, 106.7, 71.9, 67.5, 61.1, 54.6, 52.7, 43.8, 34.7, 28.6, 28.2, 26.0; HRMS (ESI) *m/z* for $C_{24}H_{28}N_2O_2$ [M+H]$^+$ calcd. 377.2224, found 377.2220; IR (ν/cm^{-1}, thin film) 2930, 2859, 1450, 1084, 819, 731, 479.

(3*R*)-3-(2-chloro-4-fluorophenyl)-*N*-(2-(pyrrolidin-1-yl)ethyl)-1-oxa-4-azaspiro[5.5]undecan-9-amine 16

4-Fluorobenzaldehyde (79 mg, 0.50 mmol, 1.0 equiv), 2-(pyrrolidin-1-yl)ethan-1-amine (57 mg, 0.50 mmol, 1.0 equiv) and iSnAP resin **1** were reacted according to the General Procedure to afford the title compound (0.16 mmol, 32% NMR yield, *dr* 64:36).

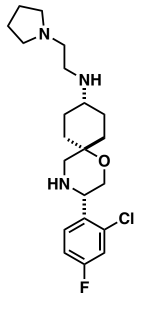

(3*S*,6*s*,9*R*)-3-(2-chloro-4-fluorophenyl)-*N*-(2-(pyrrolidin-1-yl)ethyl)-1-oxa-4-azaspiro[5.5]undecan-9-amine (major, 45 mg, 114 *μ*mol, 23% isolated yield); ^{1}H NMR (400 MHz, $CDCl_3$) δ 7.69 (dd, *J* = 8.7, 6.3 Hz, 1H), 7.09 (dd, *J* = 8.5, 2.6 Hz, 1H), 6.98 (td, *J* = 8.4, 2.6 Hz, 1H), 4.28 (dd, *J* = 10.0, 3.5 Hz, 1H), 3.70 (dd, *J* = 11.5, 3.6 Hz, 1H), 3.40 (dd, *J* = 11.4, 10.0 Hz, 1H), 2.29 (br. s, 2H), 2.90 (d, *J* = 11.4 Hz, 1H), 2.85 – 2.75 (m, 4H), 2.65 – 2.59 (m, 2H), 2.56 – 2.47 (m, 5H), 1.83 – 1.67 (m, 7H), 1.61 – 1.49 (m, 1H), 1.41 – 1.25 (m, 2H), 1.14 (td, *J* = 13.8, 3.4 Hz, 1H); ^{13}C NMR (101 MHz, $CDCl_3$) δ 161.5 (d, *J* = 249.3 Hz), 134.2 (d, *J* = 3.5 Hz), 133.8 (d, *J* = 10.0 Hz), 129.5 (d, *J* = 8.6 Hz), 116.7 (d, *J* = 24.6 Hz), 114.2 (d, *J* = 20.6 Hz), 70.4, 64.9, 57.0, 56.4, 56.2, 55.9, 54.1 (2C), 45.5, 35.0, 27.7, 27.5, 27.2, 23.5 (2C); HRMS (ESI) *m/z* for $C_{21}H_{31}ClFN_3O$ $[M+H]^+$ calcd. 396.2212, found 396.2208; IR (ν/cm^{-1}, thin film) 2930, 2856, 2794, 1487, 1231, 1087, 732, 589.

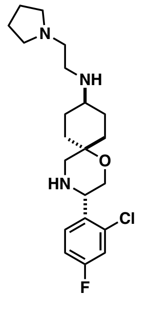

(3*S*,6*r*,9*S*)-3-(2-chloro-4-fluorophenyl)-*N*-(2-(pyrrolidin-1-yl)ethyl)-1-oxa-4-azaspiro[5.5]undecan-9-amine (minor, 12 mg, 30 *μ*mol, 6% isolated yield); ^{1}H NMR (400 MHz, $CDCl_3$) δ 7.76 – 7.66 (m, 1H), 7.10 (dd, *J* = 8.5, 2.6 Hz, 1H), 7.00 (td, *J* = 8.3, 2.6 Hz, 1H), 4.45 – 4.25 (m, 2H), 3.90 (s, 1H), 3.73 (dd, *J* = 11.3, 3.6 Hz, 1H), 3.53 (dd, *J* = 11.3, 10.0 Hz, 1H), 3.25 (d, *J* = 11.6 Hz, 1H), 2.85 – 2.72 (m, 3H), 2.70 – 2.50 (m, 7H), 1.98 – 1.68 (m, 7H), 1.62 – 1.44 (m, 2H), 1.40 – 1.19 (m, 2H), exchangeable NH x 1 not observed; ^{13}C NMR (101 MHz, $CDCl_3$) δ 161.6 (d, *J* = 249.3 Hz), 134.1 (d, *J* = 3.4 Hz), 133.7 (d, *J* = 10.1 Hz), 129.5 (d, *J* = 8.6 Hz), 116.7 (d, *J* = 24.7 Hz), 114.2 (d, *J* = 20.7 Hz), 72.0, 65.5, 56.45, 56.1, 55.6, 54.1 (2C), 52.0, 45.6, 35.3, 28.6, 28.3, 27.0, 23.4 (2C); HRMS (ESI) *m/z* for $C_{21}H_{31}ClFN_3O$ $[M+H]^+$ calcd. 396.2212, found 396.2212; IR (ν/cm^{-1}, thin film) 2932, 2859, 2796, 2360, 1683, 1488, 1230, 1089, 856.

(3*R*)-*N*-(furan-2-ylmethyl)-3-(naphthalen-2-yl)-1-oxa-4-azaspiro[5.5]undecan-9-amine 17
Quinoline-3-carbaldehyde (79 mg, 0.50 mmol, 1.0 equiv), (4-(4-methylthiazol-5-yl)phenyl)methanamine (102 mg, 0.50 mmol, 1.0 equiv) and iSnAP resin **1** were reacted according to the General Procedure to afford the title compound (0.27 mmol, 54% NMR yield, *dr* 57:43).

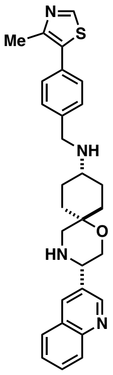

(3*S*,6*s*,9*R*)-*N*-(4-(4-methylthiazol-5-yl)benzyl)-3-(quinolin-3-yl)-1-oxa-4-azaspiro[5.5]undecan-9-amine (major, 17 mg, 61 *μ*mol, 12% isolated yield); ^{1}H NMR (400 MHz, $CDCl_3$) δ 8.95 (d, *J* = 2.2 Hz, 1H), 8.69 (s, 1H), 8.22 (d, *J* = 2.2 Hz, 1H), 8.12 (d, *J* = 8.5 Hz, 1H), 7.84 (dd, *J* = 8.1, 1.4 Hz, 1H), 7.72 (ddd, *J* = 8.4, 6.9, 1.5 Hz, 1H), 7.57 (ddd, *J* = 8.2, 6.9, 1.2 Hz, 1H), 7.42 (s, 3H), 4.12 (dd, *J* = 10.1, 3.8 Hz, 1H), 3.91 (s, 2H), 3.77 – 3.63 (m, 2H), 2.98 – 2.86 (m, 3H), 2.63 (tt, *J* = 10.8, 3.9 Hz, 1H), 2.56 (s, 3H), 1.85 (dt, *J* = 12.3, 3.6 Hz, 2H), 1.79 – 1.58 (m, 4H), 1.49 – 1.37 (m, 2H), 1.24 (td, *J* = 13.8, 3.4 Hz, 1H), exchangeable NH x 1 not observed; ^{13}C NMR (101 MHz, $CDCl_3$) δ 150.4, 150.1, 148.4, 148.0, 140.8, 133.8, 133.6, 131.8, 130.5, 129.3 (3C), 129.3, 128.4 (2C), 128.0, 127.7, 126.8, 70.5, 66.8, 58.6, 56.3, 55.8, 51.1, 35.1, 27.9, 27.7, 27.0, 16.8; HRMS (ESI) *m/z* for $C_{29}H_{32}N_4OS$ [M+H]$^+$ calcd. 485.2370, found 285.2368, IR (ν/cm^{-1}, thin film) 2931, 2856, 1495, 1319, 1124, 909, 728.

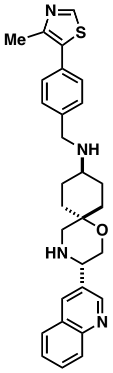

(3*S*,6*r*,9*S*)-*N*-(4-(4-methylthiazol-5-yl)benzyl)-3-(quinolin-3-yl)-1-oxa-4-azaspiro[5.5]undecan-9-amine (minor, 9 mg, 19 *μ*mol, 4% isolated yield); ^{1}H NMR (400 MHz, $CDCl_3$) δ 8.95 (d, *J* = 2.2 Hz, 1H), 8.70 (s, 1H), 8.24 (d, *J* = 2.2 Hz, 1H), 8.12 (d, *J* = 9.2 Hz, 1H), 7.87 – 7.80 (m, 1H), 7.72 (ddd, *J* = 8.4, 6.9, 1.5 Hz, 1H), 7.57 (ddd, *J* = 8.1, 6.9, 1.2 Hz, 1H), 7.44 (t, *J* = 1.2 Hz, 4H), 4.13 (dd, *J* = 9.8, 4.1 Hz, 1H), 3.88 (s, 2H), 3.82 – 3.68 (m, 2H), 3.31 (d, *J* = 11.7 Hz, 1H), 2.87 – 2.75 (m, 2H), 2.57 (s, 3H), 2.03 – 1.91 (m, 2H), 1.86 – 1.68 (m, 2H), 1.66 – 1.51 (m, 4H), 1.49 – 1.33 (m, 2H); ^{13}C NMR (126 MHz, $CDCl_3$) δ 150.5, 150.2, 148.5, 148.0, 140.7, 133.9, 133.6, 131.8, 130.6, 129.4 (2C), 129.3, 129.3, 128.4 (2C), 128.0, 127.7, 126.8, 72.0,

67.3, 58.8, 55.0, 52.7, 51.2, 34.5, 28.6, 28.3, 26.3, 16.1; HRMS (ESI) *m/z* for $C_{29}H_{32}N_4OS$ $[M+H]^+$ calcd. 485.2370, found 485.2366; IR (ν/cm^{-1}, thin film) 2932, 2858, 2359, 1445, 1089, 910, 729.

4-(((3*R*)-3-(4-fluorophenyl)-1-oxa-4-azaspiro[5.5]undecan-9-yl)amino)butan-1-ol 18

4-Fluorobenzaldehyde (62 mg, 0.50 mmol, 1.0 equiv), 4-aminobutan-1-ol (45 mg, 0.50 mmol, 1.0 equiv) and iSnAP resin **1** were reacted according to the General Procedure to afford the title compound (0.12 mmol, 24% NMR yield, *dr* 62:38).

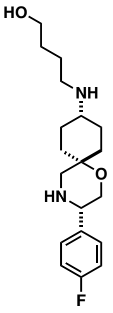

4-(((3*S*,6*s*,9*R*)-3-(4-fluorophenyl)-1-oxa-4-azaspiro[5.5]undecan-9-yl)amino)butan-1-ol (major, 13 mg, 39 μmol, 8% isolated yield); ^{1}H NMR (400 MHz, $CDCl_3$) δ 7.33 – 7.24 (m, 2H), 7.02 – 6.87 (m, 2H), 3.76 (dd, *J* = 10.3, 3.8 Hz, 1H), 3.53 – 3.37 (m, 4H), 2.82 – 2.68 (m, 3H), 2.67 – 2.59 (m, 2H), 2.42 (tt, *J* = 11.1, 4.0 Hz, 1H), 1.78 – 1.67 (m, 2H), 1.65 – 1.39 (m, 6H), 1.33 – 1.20 (m, 2H), 1.19 (d, *J* = 2.5 Hz, 3H), 1.05 (td, *J* = 13.9, 3.5 Hz, 1H); ^{13}C NMR (126 MHz, $CDCl_3$) δ 162.4 (d, *J* = 245.7 Hz), 136.6 (d, *J* = 3.1 Hz), 128.8 (d, *J* = 7.9 Hz, 2C), 115.4 (d, *J* = 21.2 Hz, 2C), 70.0, 67.1, 62.4, 60.2, 56.8, 56.1, 46.2, 35.2, 32.2, 28.2, 27.1, 26.7, 26.4; HRMS (ESI) *m/z* for $C_{19}H_{29}FN_2O_2$ $[M+H]^+$ calcd. 337.2286, found 337.2287; IR (ν/cm^{-1}, thin film) 2925, 2852, 1508, 1221, 1064, 835.

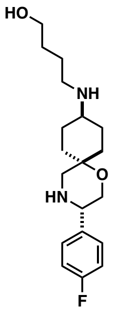

4-(((3*S*,6*r*,9*S*)-3-(4-fluorophenyl)-1-oxa-4-azaspiro[5.5]undecan-9-yl)amino)butan-1-ol (minor, 18 mg, 54 μmol, 11% isolated yield); ^{1}H NMR (500 MHz, $CDCl_3$) δ 7.41 – 7.36 (m, 2H), 7.06 – 7.00 (m, 2H), 3.86 (dd, *J* = 10.1, 4.0 Hz, 1H), 3.65 – 3.54 (m, 4H), 3.22 (d, *J* = 11.7 Hz, 1H), 2.76 – 2.65 (m, 4H), 2.50 – 2.43 (m, 1H), 1.99 – 1.87 (m, 2H), 1.76 – 1.61 (m, 6H), 1.58 – 1.47 (m, 1H), 1.39 – 1.25 (m, 3H), exchangeable NH/OH x 2 not observed; ^{13}C NMR (126 MHz, $CDCl_3$) δ 162.3 (d, *J* = 245.5 Hz), 136.6 (d, *J* = 3.1 Hz), 128.7 (d, *J* = 7.9 Hz, 2C), 115.2 (d, *J* = 21.2 Hz, 2C), 71.6, 67.6, 62.6, 60.2, 55.5, 52.7, 47.3, 34.5, 32.7, 29.5, 28.3, 27.9, 26.3; HRMS (ESI) *m/z* for $C_{19}H_{29}FN_2O_2$ $[M+H]^+$ calcd. 337.2286, found 337.2283; IR (ν/cm^{-1}, thin film) 2925, 2856, 2360, 1509, 1221, 1090, 835.

(3*S*)-3-(furan-2-yl)-*N*-(2-(methylthio)ethyl)-1-oxa-4-azaspiro[5.5]undecan-9-amine 19

Furan-2-carbaldehyde (48 mg, 0.5 mmol, 1.0 equiv), 2-(methylthio)ethan-1-amine (46 mg, 0.5 mmol, 1.0 equiv) and iSnAP resin 1 were reacted according to the General Procedure to afford the title compound (0.17 mmol, 33% NMR yield, *dr* 64:36).

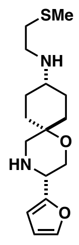

(3*R*,6*s*,9*S*)-3-(furan-2-yl)-*N*-(2-(methylthio)ethyl)-1-oxa-4-azaspiro[5.5]undecan-9-amine (major, 11 mg, 35 μmol, 7% isolated yield); ^{1}H NMR (400 MHz, $CDCl_3$) δ 7.36 (dd, *J* = 1.9, 0.9 Hz, 1H), 6.33 (dd, *J* = 3.3, 1.9 Hz, 1H), 6.22 (d, *J* = 3.3 Hz, 1H), 3.97 (dd, *J* = 9.4, 4.2 Hz, 1H), 3.82 – 3.67 (m, 2H), 2.88 (t, *J* = 6.5 Hz, 2H), 2.79 (q, *J* = 12.1 Hz, 2H), 2.71 – 2.60 (m, 3H), 2.52 (tt, *J* = 11.0, 3.9 Hz, 1H), 2.11 (s, 3H), 1.83 – 1.65 (m, 5H), 1.62 – 1.49 (m, 1H), 1.45 – 1.25 (m, 2H), 1.12 (td, *J* = 13.8, 3.5 Hz, 1H); ^{13}C NMR (101 MHz, $CDCl_3$) δ 153.8, 141.4, 110.1, 105.9, 70.0, 63.6, 56.5, 55.1, 53.4, 44.9, 34.8, 34.5, 27.9, 27.6, 26.6, 15.2; HRMS (ESI) *m/z* for $C_{16}H_{26}N_2O_2S$ $[M+H]^+$ calcd. 311.1788, found 311.1784; IR (thin film, ν/cm^{-1}) 2920, 2856, 1680, 1442, 1092, 737.

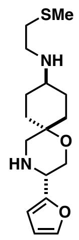

(3*R*,6*r*,9*R*)-3-(furan-2-yl)-*N*-(2-(methylthio)ethyl)-1-oxa-4-azaspiro[5.5]undecan-9-amine (minor, 13 mg, 42 μmol, 8% isolated yield); ^{1}H NMR (400 MHz, $CDCl_3$) δ 7.37 (dd, *J* = 1.9, 0.9 Hz, 1H), 6.34 (dd, *J* = 3.3, 1.8 Hz, 1H), 6.24 (dt, *J* = 3.2, 0.8 Hz, 1H), 3.98 (dd, *J* = 9.5, 3.9 Hz, 1H), 3.90 – 3.76 (m, 2H), 3.15 (d, *J* = 12.2 Hz, 1H), 2.90 (t, *J* = 6.5 Hz, 3H), 2.79 – 2.67 (m, 4H), 2.42 – 2.31 (m, 1H), 2.13 (s, 3H), 1.98 – 1.86 (m, 2H), 1.82 – 1.73 (m, 1H), 1.65 – 1.48 (m, 2H), 1.42 – 1.25 (m, 2H), exchangeable NH x 1 not observed; ^{13}C NMR (101 MHz, $CDCl_3$) δ 153.8, 141.9, 110.1, 106.0, 71.5, 64.2, 55.3, 53.5, 51.6, 45.0, 33.9, 33.6, 28.0, 27.9, 26.8, 15.2; HRMS (ESI) *m/z* for $C_{16}H_{26}N_2O_2S$ $[M+H]^+$ calcd. 311.1788, found 311.1785; IR (ν/cm^{-1}, thin film) 2922, 2855, 2362, 1677, 1455, 1093, 738.

2-fluoro-5-((3*R*)-9-(isopropylamino)-1-oxa-4-azaspiro[5.5]undecan-3-yl)benzonitrile 20
2-Fluoro-5-formylbenzonitrile (75 mg, 0.50 mmol, 1.0 equiv), propan-2-amine (30 mg, 0.50 mmol, 1.0 equiv) and iSnAP resin **1** were reacted according to the General Procedure to afford the title compound (0.19 mmol, 38% NMR yield, *dr* 54:46).

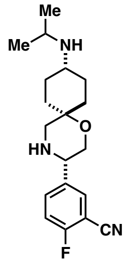

2-Fluoro-5-((3*S*,6*s*,9*R*)-9-(isopropylamino)-1-oxa-4-azaspiro[5.5]undecan-3-yl)benzonitrile (major, 26 mg, 79 *μ*mol, 16% isolated yield); ^{1}H NMR (400 MHz, $CDCl_3$) δ 7.74 (dd, *J* = 6.1, 2.2 Hz, 1H), 7.64 (ddd, *J* = 8.8, 5.1, 2.3 Hz, 1H), 7.18 (t, *J* = 8.7 Hz, 1H), 3.88 (dd, *J* = 10.2, 3.7 Hz, 1H), 3.56 (dd, *J* = 11.6, 3.7 Hz, 1H), 3.45 (dd, *J* = 11.5, 10.3 Hz, 1H), 3.05 (p, *J* = 6.3 Hz, 1H), 2.90 – 2.75 (m, 3H), 2.69 – 2.59 (m, 1H), 1.81 – 1.71 (m, 2H), 1.67 (m, 1H), 1.58 – 1.46 (m, 1H), 1.39 (dd, *J* = 13.2, 3.9 Hz, 1H), 1.35 – 1.23 (m, 1H), 1.16 (td, *J* = 13.9, 3.3 Hz, 1H), 1.08 (d, *J* = 6.3 Hz, 6H), exchangeable NH x 2 not observed; ^{13}C NMR (101 MHz, $CDCl_3$) δ 162.5 (d, *J* = 259.1 Hz), 138.1 (d, *J* = 3.6 Hz), 133.9 (d, *J* = 8.2 Hz), 132.1 (2C), 116.4 (d, *J* = 19.5 Hz), 113.9, 101.5 (d, *J* = 15.5 Hz), 70.4, 66.6, 59.4, 55.6, 53.4, 44.5, 35.2, 27.8, 27.6, 27.1, 22.6; HRMS (ESI) *m/z* for $C_{19}H_{26}FN_3O$ $[M+H]^+$ calcd. 332.2133, found 332.2132; IR (ν/cm^{-1}, thin film) 2929, 2858, 1680, 1442, 1114, 880, 732.

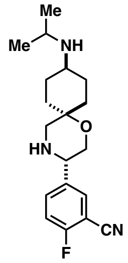

2-fluoro-5-((3*S*,6*r*,9*S*)-9-(isopropylamino)-1-oxa-4-azaspiro[5.5]undecan-3-yl)benzonitrile (minor, 22 mg, 66 *μ*mol, 13% isolated yield); ^{1}H NMR (400 MHz, $CDCl_3$) δ 7.74 (dd, *J* = 6.1, 2.3 Hz, 1H), 7.68 – 7.60 (m, 1H), 7.18 (t, *J* = 8.7 Hz, 1H), 3.89 (dd, *J* = 9.0, 5.0 Hz, 1H), 3.63 – 3.52 (m, 2H), 3.26 (d, *J* = 11.7 Hz, 1H), 2.98 (p, *J* = 6.3 Hz, 1H), 2.79 – 2.67 (m, 2H), 2.63 – 2.53 (m, 1H), 1.96 – 1.82 (m, 2H), 1.75 – 1.60 (m, 2H), 1.60 – 1.44 (m, 2H), 1.34 – 1.16 (m, 2H), 1.10 (d, *J* = 6.2 Hz, 6H), exchangeable NH x 1 not observed; ^{13}C NMR (101 MHz, $CDCl_3$) δ 162.5 (d, *J* = 259.1 Hz), 138.1 (d, *J* = 3.6 Hz), 133.9 (d, *J* = 8.2 Hz), 132.1, 116.4 (d, *J* = 19.5 Hz), 113.9, 101.5 (d, *J* = 15.5 Hz), 72.1, 67.2, 59.5, 52.6, 51.8, 45.4, 35.0, 29.1, 28.8, 26.9, 23.2, 23.1; HRMS (ESI) *m/z* for $C_{19}H_{26}FN_3O$ $[M+H]^+$ calcd. 332.2133, found 332.2133; IR (ν/cm^{-1}, thin film) 2929, 2859, 1679, 1497, 1088, 831, 731.

N-cyclopropyl-3-(3,4,5-trifluorophenyl)-1-oxa-4-azaspiro[5.5]undecan-9-amine 21

3,4,5-Trifluorobenzaldehyde (80 mg, 0.50 mmol, 1.0 equiv), cyclopropanamine (29 mg, 0.50 mmol, 1.0 equiv) and iSnAP resin **1** were reacted according to the General Procedure to afford the title compound (0.20 mmol, 40% NMR yield, *dr* 50:50).

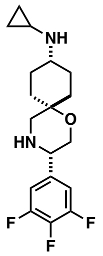

(3*S*,6*s*,9*R*)-*N*-cyclopropyl-3-(3,4,5-trifluorophenyl)-1-oxa-4-azaspiro[5.5]undecan-9-amine (major, 19 mg, 56 μmol, 11% isolated yield); ^{1}H NMR (500 MHz, $CDCl_3$) δ 7.13 – 7.03 (m, 2H), 3.81 (dd, *J* = 10.3, 3.7 Hz, 1H), 3.56 (dd, *J* = 11.6, 3.7 Hz, 1H), 3.44 (dd, *J* = 11.5, 10.3 Hz, 1H), 2.88 – 2.79 (m, 2H), 2.79 – 2.73 (m, 1H), 2.65 (tt, *J* = 11.0, 4.0 Hz, 1H), 2.17 (tt, *J* = 6.7, 3.7 Hz, 1H), 1.86 – 1.79 (m, 2H), 1.71 – 1.63 (m, 2H), 1.60 – 1.48 (m, 1H), 1.44 – 1.26 (m, 2H), 1.17 (td, *J* = 13.9, 3.4 Hz, 1H), 0.49 – 0.43 (m, 2H), 0.39 – 0.34 (m, 2H), exchangeable NH x 1 not observed; ^{13}C NMR (126 MHz, $CDCl_3$) δ 151.2 (ddd, *J* = 249.9, 9.9, 3.9 Hz, 2C), 138.9 (dt, *J* = 251.0, 15.3 Hz), 137.3 (td, *J* = 6.8, 4.4 Hz); 111.0 (dd, *J* = 16.5, 5.0 Hz, 2C), 70.5, 66.6, 59.6, 57.2, 55.6, 35.1, 28.2, 28.0, 27.8, 27.0, 6.4 (2C); HRMS (ESI) *m/z* for $C_{18}H_{23}F_3N_2O$ $[M+H]^+$ calcd. 341.1835, found 341.1837; IR (ν/cm^{-1}, thin film) 2937, 2857, 1618, 1528, 1442, 1350, 1089, 1048, 825, 710.

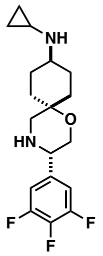

(3*S*,6*r*,9*S*)-*N*-cyclopropyl-3-(3,4,5-trifluorophenyl)-1-oxa-4-azaspiro[5.5]undecan-9-amine (minor, 8 mg, 34 μmol, 5% isolated yield); ^{1}H NMR (500 MHz, $CDCl_3$) δ 7.10 – 7.01 (m, 2H), 3.83 – 3.76 (m, 1H), 3.59 – 3.50 (m, 2H), 3.19 (d, *J* = 11.7 Hz, 1H), 2.78 (tt, *J* = 8.7, 3.9 Hz, 1H), 2.68 (dd, *J* = 11.7, 1.1 Hz, 1H), 2.51 – 2.42 (m, 1H), 2.11 (tt, *J* = 6.6, 3.7 Hz, 1H), 1.97 – 1.89 (m, 2H), 1.75 (s, 1H), 1.71 – 1.64 (m, 1H), 1.59 – 1.47 (m, 2H), 1.34 – 1.21 (m, 2H), 0.50 – 0.45 (m, 2H), 0.38 – 0.33 (m, 2H); exchangeable NH x 1 not observed; ^{13}C NMR (126 MHz, $CDCl_3$) δ151.2 (ddd, *J* = 250.0, 9.9, 3.9 Hz, 2C), 138.9 (dt, *J* = 251.0, 15.3 Hz), 137.3 (td, *J* = 6.8, 4.3 Hz), 111.1 (dd, *J* = 16.5, 4.9 Hz, 2C), 72.1, 67.1, 59.7, 56.2, 52.1, 34.7, 28.8, 28.7, 28.5, 26.5, 6.4, 6.3; HRMS (ESI) *m/z* for $C_{18}H_{23}F_3N_2O$ $[M+H]^+$ calcd. 341.1835, found 341.1835; IR (ν/cm^{-1}, thin film) 2937, 2860, 1618, 1530, 1443, 1351, 1090, 1048, 855, 711.

N-(2-(methylthio)ethyl)-3-(thiophen-2-yl)-1-oxa-4-azaspiro[5.5]undecan-9-amine 22
Thiophene-2-carbaldehyde (56 mg, 0.50 mmol, 1.0 equiv), 2-(methylthio)ethan-1-amine (46 mg, 0.50 mmol, 1.0 equiv) and iSnAP resin **1** were reacted according to the General Procedure to afford the title compound (0.13 mmol, 26% NMR yield, *dr* 50:50).

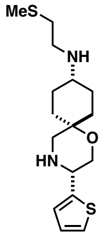

(3*R*,6*s*,9*S*)-*N*-(2-(methylthio)ethyl)-3-(thiophen-2-yl)-1-oxa-4-azaspiro[5.5]undecan-9-amine (major, 17 mg, 52 μmol, 10% isolated yield) ^{1}H NMR (400 MHz, $CDCl_3$) δ 7.25 – 7.21 (m, 1H), 7.00 – 6.96 (m, 2H), 4.18 (dd, *J* = 10.1, 3.9 Hz, 1H), 3.70 (dd, *J* = 11.6, 3.9 Hz, 1H), 3.61 (dd, *J* = 11.5, 10.1 Hz, 1H), 2.90 – 2.84 (m, 3H), 2.82 – 2.73 (m, 2H), 2.67 (td, *J* = 6.5, 0.5 Hz, 2H), 2.52 (tt, *J* = 11.0, 4.0 Hz, 1H), 2.11 (s, 3H), 1.82 – 1.64 (m, 4H), 1.56 (tdd, *J* = 12.5, 10.8, 3.6 Hz, 1H), 1.41 – 1.28 (m, 2H), 1.19 – 1.09 (m, 1H), exchangeable NH x 1 not observed; ^{13}C NMR (101 MHz, $CDCl_3$) δ 144.2, 126.6, 124.3, 123.9, 70.2, 67.0, 56.5, 55.9, 55.7, 44.9, 35.0, 34.8, 27.9, 27.6, 26.9, 15.2; HRMS (ESI) *m/z* for $C_{16}H_{26}N_2OS_2$ [M+H]$^+$ calcd. 327.1559, found 327.1559; IR (ν/cm^{-1}, thin film) 2918, 2856, 1669, 1439, 1312, 1089, 1065, 701.

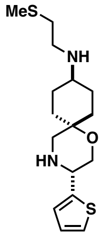

(3*R*,6*r*,9*R*)-*N*-(2-(methylthio)ethyl)-3-(thiophen-2-yl)-1-oxa-4-azaspiro[5.5]undecan-9-amine (minor, 8 mg, 25 μmol, 5% isolated yield) ^{1}H NMR (400 MHz, $CDCl_3$) δ 7.27 – 7.22 (m, 1H), 7.02 – 6.96 (m, 2H), 4.18 (dd, *J* = 9.4, 4.4 Hz, 1H), 3.79 – 3.68 (m, 2H), 3.21 (d, *J* = 12.0 Hz, 1H), 2.91 – 2.84 (m, 2H), 2.77 – 2.65 (m, 5H), 2.53 – 2.44 (m, 1H), 2.12 (d, *J* = 0.3 Hz, 4H), 1.96 – 1.85 (m, 2H), 1.79 – 1.69 (m, 1H), 1.64 – 1.48 (m, 2H), 1.33 (td, *J* = 14.3, 7.0 Hz, 2H); ^{13}C NMR (101 MHz, $CDCl_3$) δ 144.1, 126.6, 124.3, 124.0, 71.7, 67.6, 56.0, 55.3, 52.2, 45.0, 34.4, 34.3, 28.5, 28.2, 26.4, 15.2; HRMS (ESI) *m/z* for $C_{16}H_{26}N_2OS_2$ [M+H]$^+$ calcd. 327.1559, found 327.1557; IR (ν/cm^{-1}, thin film) 2916, 2857, 2806, 1671, 1438, 1199, 1088, 701.

4-(9-(isopropylamino)-1-oxa-4-azaspiro[5.5]undecan-3-yl)benzonitrile 23

4-Formylbenzonitrile (65 mg, 0.50 mmol, 1.0 equiv), propan-2-amine (30 mg, 0.50 mmol, 1.0 equiv) and iSnAP resin **1** were reacted according to the General Procedure to afford the title compound (0.21 mmol, 43% NMR yield, *dr* 45:55).

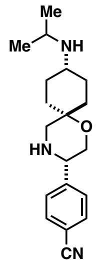

4-((3*S*,6*s*,9*R*)-9-(isopropylamino)-1-oxa-4-azaspiro[5.5]undecan-3-yl)benzonitrile (major, 46 mg, 147 *μ*mol, 29% isolated yield) ^{1}H NMR (400 MHz, $CDCl_3$) δ 7.66 – 7.58 (m, 2H), 7.57 – 7.50 (m, 2H), 3.93 (dd, *J* = 10.3, 3.7 Hz, 1H), 3.59 (dd, *J* = 11.6, 3.7 Hz, 1H), 3.48 (dd, *J* = 11.6, 10.2 Hz, 1H), 3.10 – 3.00 (m, 1H), 2.91 – 2.75 (m, 3H), 2.69 – 2.58 (m, 1H), 1.75 (ddt, *J* = 12.1, 3.8, 2.0 Hz, 2H), 1.67 (dq, *J* = 13.3, 3.3 Hz, 1H), 1.58 – 1.46 (m, 1H), 1.42 – 1.24 (m, 3H), 1.15 (td, *J* = 13.9, 3.3 Hz, 1H), 1.08 (d, *J* = 6.2 Hz, 6H), exchangeable NH x 1 not observed; ^{13}C NMR (101 MHz, $CDCl_3$) δ 146.2, 132.3 (2C), 127.9 (2C), 118.8, 111.5, 70.4, 66.5, 60.5, 55.6, 53.4, 44.9, 35.2, 27.8, 27.6, 27.1, 23.0, 23.0; HRMS (ESI) *m/z* for $C_{19}H_{27}N_3O$ $[M+Na]^+$ calcd. 336.2046, found 336.2049; IR (ν/cm^{-1}, thin film) 2931. 2857, 2226, 1441, 1376, 1168, 1087, 1065, 834, 729.

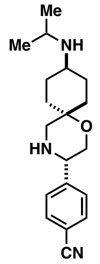

4-((3*S*,6*r*,9*S*)-9-(isopropylamino)-1-oxa-4-azaspiro[5.5]undecan-3-yl)benzonitrile (minor, 23 mg, 73 *μ*mol, 15% isolated yield); ^{1}H NMR (400 MHz, $CDCl_3$) δ 7.66 – 7.58 (m, 2H), 7.57 – 7.50 (m, 2H), 3.93 (dd, *J* = 9.3, 4.6 Hz, 1H), 3.67 – 3.54 (m, 2H), 3.27 (d, *J* = 11.7 Hz, 1H), 3.03 – 2.89 (m, 1H), 2.78 – 2.67 (m, 2H), 2.64 – 2.54 (m, 1H), 1.94 – 1.84 (m, 2H), 1.74 – 1.66 (m, 2H), 1.59 – 1.45 (m, 2H), 1.31 – 1.15 (m, 2H), 1.08 (d, *J* = 6.3 Hz, 6H), exchangeable NH x 1 not observed; ^{13}C NMR (101 MHz, $CDCl_3$) δ 146.3, 132.3 (2C), 128.0 (2C), 118.8, 111.5, 72.1, 67.1, 60.6, 52.6, 51.8, 45.4, 35.1, 29.4, 29.0, 27.0, 23.4, 23.3; HRMS (ESI) *m/z* for $C_{19}H_{27}N_3O$ $[M+H]^+$ calcd. 314.227, found 314.225; IR (ν/cm^{-1}, thin film) 2916, 2857, 2806, 1671, 1438, 1199, 1131, 1088, 701.

3-(1-methyl-1*H*-pyrazol-4-yl)-*N*-(2,2,2-trifluoroethyl)-1-oxa-4-azaspiro[5.5]undecan-8-amine 24

1-Methyl-1*H*-pyrazole-4-carbaldehyde (55 mg, 0.50 mmol, 1.0 equiv), 2,2,2-trifluoroethan-1-amine (50 mg, 0.50 mmol, 1.0 equiv) and iSnAP resin **2** were reacted according to the General Procedure to afford the title compound (0.09 mmol, 19% NMR yield, *dr* 20:30:20:30).

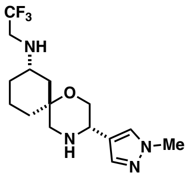

(3*S*,6*R*,8*S*)-3-(1-methyl-1*H*-pyrazol-4-yl)-*N*-(2,2,2-trifluoroethyl)-1-oxa-4-azaspiro[5.5]undecan-8-amine (10 mg, 30 μmol, 6% isolated yield); ^{1}H NMR (400 MHz, $CDCl_3$) δ 7.46 (s, 1H), 7.36 (s, 1H), 3.94 – 3.89 (m, 1H), 3.88 (s, 3H), 3.74 – 3.61 (m, 2H), 3.20 (q, *J* = 9.4 Hz, 2H), 2.99 (d, *J* = 12.1 Hz, 1H), 2.76 (d, *J* = 12.2 Hz, 1H), 2.75 (s, 1H), 2.16 – 2.08 (m, 1H), 1.82 – 1.68 (m, 4H), 1.49 (dd, *J* = 13.1, 8.5 Hz, 1H), 1.43 – 1.31 (m, 2H), exchangeable NH x 2 not observed; ^{13}C NMR (126 MHz, $CDCl_3$) δ 137.6, 128.2, 125.6 (d, *J* = 277.8 Hz), 120.5, 72.3, 66.2, 52.9, 52.6, 51.5, 48.0 (q, *J* = 31.2 Hz), 42.2, 38.9, 32.0, 29.1, 18.9; HRMS (ESI) *m/z* for $C_{15}H_{23}F_3N_4O$ $[M+H]^+$ calcd. 333.1897, found 333.1897; IR (ν/cm^{-1}, thin film) 2933, 2860, 2361, 1671, 1270, 1143, 1090, 856.

***N*-(2-(pyridin-2-yl)ethyl)-3-(4-(trifluoromethyl)phenyl)-1-oxa-4-azaspiro[5.5]undecan-8-amine 25**

4-(trifluoromethyl)benzaldehyde (87 mg, 0.50 mmol, 1.0 equiv), 2-(pyridin-2-yl)ethan-1-amine (61 mg, 0.50 mmol, 1.0 equiv) and iSnAP resin **2** were reacted according to the General Procedure to afford the title compound (0.17 mmol, 35% NMR yield, *dr* 12:19:31:38).

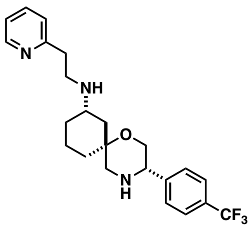

(3*S*,6*R*,8*S*)-*N*-(2-(pyridin-2-yl)ethyl)-3-(4-(trifluoromethyl)phenyl)-1-oxa-4-azaspiro[5.5]undecan-8-amine (5 mg, 12 μmol, 2% isolated yield); 1H NMR (400 MHz, $CDCl_3$) δ 8.54 (dd, *J* = 5.0, 1.9 Hz, 1H), 7.71 – 7.43 (m, 5H), 7.25 – 7.04 (m, 2H), 3.94 (dd, *J* = 10.2, 3.8 Hz, 1H), 3.65 (t, *J* = 10.8 Hz, 1H), 3.56 (dd, *J* = 11.4, 3.8 Hz, 1H), 3.21 – 2.93 (m, 5H), 2.82 – 2.60 (m, 2H), 2.54 – 2.40 (m, 1H), 1.81 (dtt, *J* = 24.2, 13.3, 4.1 Hz, 3H), 1.64 (s, 1H), 1.57 – 1.23 (m, 5H); ^{13}C NMR (126 MHz, $CDCl_3$) δ 160.2, 149.3, 144.9, 136.4, 129.9 (d, *J* = 32.3 Hz), 127.5 (3C), 125.4 (q, *J* = 3.8 Hz, 2C), 123.3, 121.3, 72.9, 67.0, 60.4, 53.4, 52.6, 46.5, 43.5, 38.6, 32.3, 29.2, 20.1; HRMS (ESI) *m/z* for $C_{23}H_{28}F_3N_3O$ $[M+H]^+$ calcd. 420.2257, measured 420.2253; IR (ν/cm^{-1}, thin film) 2932, 2858, 1590, 1474, 1324, 1162, 1122, 1066, 838, 605.

4-(8-((furan-2-ylmethyl)amino)-1-oxa-4-azaspiro[5.5]undecan-3-yl)benzonitrile 26

4-Formylbenzonitrile (66 mg, 0.50 mmol, 1.0 equiv), furan-2-ylmethanamine (48 mg, 0.50 mmol, 1.0 equiv) and iSnAP resin **2** were reacted according to the General Procedure to afford the title compound (0.17 mmol, 33% NMR yield, *dr* 12:25:16:47).

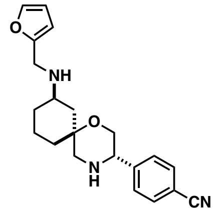

4-((3*S*,6*S*,8*R*)-8-((furan-2-ylmethyl)amino)-1-oxa-4-azaspiro[5.5]undecan-3-yl)benzonitrile (3 mg, 9 μmol, 2% isolated yield); ^{1}H NMR (500 MHz, $CDCl_3$) δ 7.68 – 7.64 (m, 2H), 7.58 – 7.54 (m, 2H), 7.35 (dd, *J* = 1.8, 0.9 Hz, 1H), 6.34 – 6.29 (m, 2H), 4.07 – 3.96 (m, 2H), 3.92 (dd, *J* = 10.1, 3.8 Hz, 1H), 3.60 – 3.47 (m, 2H), 3.21 (d, *J* = 13.8 Hz, 1H), 2.94 – 2.83 (m, 3H), 2.13 – 2.03 (m, 2H), 1.73 (dt, *J* = 13.2, 3.7 Hz, 1H), 1.70 – 1.59 (m, 2H), 1.34 – 1.26 (m, 2H), 1.11 (t, *J* = 12.8 Hz, 1H), exchangeable x 1NH not observed; ^{13}C NMR (126 MHz, $CDCl_3$) δ 145.9, 142.7 (2C), 132.3 (2C), 128.0 (2C), 118.7, 111.7, 110.6 (2C), 71.9, 66.5, 60.5, 55.8, 51.5, 41.9, 35.6, 33.3, 31.4, 19.3; HRMS (ESI) *m/z* for $C_{21}H_{25}N_3O_2$ $[M+H]^+$ calcd. 352.2020, measured 352.2017; IR (ν/cm^{-1}, thin film) 2939, 2359, 2341, 1674, 1200, 1131, 1068, 835, 742.

3-(2-chlorophenyl)-*N*-cyclopropyl-1-oxa-4-azaspiro[5.5]undecan-8-amine 27

2-Chlorobenzaldehyde (70 mg, 0.50 mmol, 1.0 equiv), cyclopropanamine (29 mg, 0.50 mmol, 1.0 equiv) and iSnAP resin **2** were reacted according to the General Procedure to afford the title compound (0.10 mmol, 22% NMR yield, *dr* 36:18:27:18).

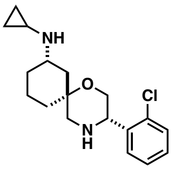

(3*S*,6*R*,8*S*)-3-(2-chlorophenyl)-*N*-cyclopropyl-1-oxa-4-azaspiro[5.5]undecan-8-amine (8 mg, 25 μmol, 5% isolated yield); ^{1}H NMR (500 MHz, $CDCl_3$) δ 7.71 (dd, J = 7.7, 1.8 Hz, 1H), 7.36 (dd, J = 7.9, 1.4 Hz, 1H), 7.31 – 7.26 (m, 1H), 7.25 – 7.18 (m, 1H), 4.37 (dd, J = 10.1, 3.5 Hz, 1H), 3.77 (dd, J = 11.3, 3.5 Hz, 1H), 3.60 (dd, J = 11.3, 10.1 Hz, 1H), 3.16 (d, J = 11.6 Hz, 1H), 2.91 – 2.80 (m, 2H), 2.44 – 2.32 (m, 1H), 2.24 (tt, J = 6.5, 3.9 Hz, 1H), 1.94 – 1.86 (m, 2H), 1.78 (m, 1H), 1.69 – 1.60 (m, 1H), 1.52 (d, J = 11.1 Hz, 1H), 1.43 (q, J = 10.6 Hz, 2H), 0.56 – 0.44 (m, 4H), exchangeable NH x 2 not observed; ^{13}C NMR (126 MHz, CDCl3) δ 138.0, 133.3, 129.5, 128.6, 128.3, 127.1, 73.0, 65.3, 56.9, 54.0, 53.0, 42.8, 32.3, 29.2, 28.1, 19.8, 6.1 (2C); HRMS (ESI) *m/z* for $C_{18}H_{25}ClN_2O$ $[M+H]^+$ calcd. 321.1728, measured 321.1726; IR (ν/cm^{-1}, thin film) 2932, 2860, 1441, 1335, 1085, 1072, 755, 730.

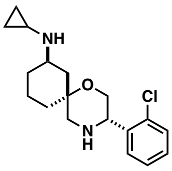

(3*S*,6*R*,8*R*)-3-(2-chlorophenyl)-*N*-cyclopropyl-1-oxa-4-azaspiro[5.5]undecan-8-amine (4 mg, 13 μmol, 3% isolated yield); ^{1}H NMR (500 MHz, $CDCl_3$) δ 7.74 (ddt, *J* = 7.8, 1.8, 0.5 Hz, 1H), 7.37 – 7.33 (m, 1H), 7.31 – 7.26 (m, 1H), 7.22 (ddd, *J* = 7.9, 7.4, 1.8 Hz, 1H), 4.36 (dd, *J* = 9.9, 3.6 Hz, 1H), 3.82 – 3.74 (m, 1H), 3.68 (dd, *J* = 11.4, 9.9 Hz, 1H), 3.19 (d, *J* = 11.8 Hz, 1H), 2.81 (dt, *J* = 11.8, 4.3 Hz, 3H), 2.31 – 2.22 (m, 1H), 2.08 – 1.96 (m, 2H), 1.78 (tt, *J* = 9.8, 5.2 Hz, 1H), 1.67 (d, *J* = 13.6 Hz, 1H), 1.54 (td, *J* = 12.3, 4.2 Hz, 1H), 1.43 – 1.26 (m, 3H), 0.57 – 0.43 (m, 5H); ^{13}C NMR (126 MHz, $CDCl_3$) δ 138.0, 133.2, 129.4, 128.6, 128.5, 127.1, 72.6, 65.3, 57.0, 54.5, 53.1, 36.9, 36.1, 32.3, 28.1, 19.9, 6.7, 5.6; HRMS (ESI) *m/z* for $C_{18}H_{25}ClN_2O$ [M+H]$^+$ calcd. 321.1728, measured 321.1726; IR (ν/cm^{-1}, thin film) 2932, 2860, 1471, 1441, 1085, 1072, 755, 730.

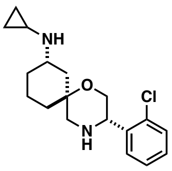

(3*S*,6*S*,8*S*)-3-(2-chlorophenyl)-*N*-cyclopropyl-1-oxa-4-azaspiro[5.5]undecan-8-amine (8 mg, 25 μmol, 5% isolated yield); ^{1}H NMR (500 MHz, $CDCl_3$) δ 7.71 (dd, *J* = 7.7, 1.8 Hz, 1H), 7.36 (dd, *J* = 7.9, 1.3 Hz, 1H), 7.31 – 7.25 (m, 1H), 7.24 – 7.16 (m, 1H), 4.37 (dd, *J* = 10.1, 3.6 Hz, 1H), 3.77 (dd, *J* = 11.4, 3.5 Hz, 1H), 3.45 (dd, *J* = 11.4, 10.1 Hz, 1H), 3.08 (tt, *J* = 11.5, 4.1 Hz, 1H), 2.94 (d, *J* = 11.4 Hz, 1H), 2.87 (d, *J* = 11.5 Hz, 1H), 2.83 (d, *J* = 15.5 Hz, 1H), 2.21 (tt, *J* = 6.7, 3.7 Hz, 1H), 2.13 – 2.08 (m, 1H), 2.04 – 1.97 (m, 1H), 1.62 (dt, *J* = 13.5, 3.6 Hz, 1H), 1.52 (qt, *J* = 13.4, 3.5 Hz, 1H), 1.13 (dd, *J* = 12.8, 11.6 Hz, 1H), 1.10 – 0.98 (m, 2H), 0.54 – 0.46 (m, 2H), 0.45 – 0.37 (m, 2H), exchangeable NH x 2 not observed;^{13}C NMR (126 MHz, $CDCl_3$) δ 138.1, 133.3, 129.5, 128.5, 128.3, 127.1, 72.3, 64.9, 56.9, 56.2, 52.9, 43.2, 33.5, 28.3 (2C), 19.4, 6.3 (2C); HRMS (ESI) *m/z* for $C_{18}H_{25}ClN_2O$ [M+H]$^+$ calcd. 321.1728, measured 321.1729; IR (ν/cm^{-1}, thin film) 2931, 2861, 1674, 1447, 1200, 1085, 755, 721.

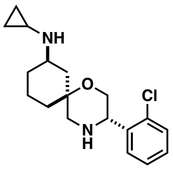

(3*S*,6*S*,8*R*)-3-(2-chlorophenyl)-*N*-cyclopropyl-1-oxa-4-azaspiro[5.5]undecan-8-amine (6 mg, 19 μmol, 4% isolated yield); ^{1}H NMR (500 MHz, $CDCl_3$) δ 7.73 (ddt, *J* = 7.8, 1.8, 0.5 Hz, 1H), 7.38 – 7.34 (m, 1H), 7.31 – 7.26 (m, 1H), 7.22 (ddd, *J* = 7.9, 7.4, 1.8 Hz, 1H), 4.36 (dd, *J* = 10.0, 3.5 Hz, 1H), 3.80 (dd, *J* = 11.5, 3.5 Hz, 1H), 3.54 (dd, *J* = 11.3, 10.0 Hz, 1H), 3.17 (dd, *J* = 13.8, 3.1 Hz, 1H), 2.94 (d, *J* = 11.5 Hz, 1H), 2.91 – 2.82 (m, 2H), 2.24 (tt, *J* = 6.7, 3.8 Hz, 1H), 2.15 – 2.10 (m, 1H), 2.06 – 1.91 (m, 2H), 1.83 – 1.72 (m, 1H), 1.72 – 1.59 (m, 2H), 1.32 – 1.22 (m, 1H), 1.16 – 1.04 (m, 1H), 0.93 (dd, *J* = 13.7, 11.8 Hz, 1H), 0.51 (dddd, *J* = 6.5, 4.1, 2.8, 1.2 Hz, 2H), 0.48 – 0.41 (m, 2H); ^{13}C NMR (126 MHz, $CDCl_3$) δ 138.1, 133.3, 129.5, 128.5,

128.4, 127.1, 72.1, 65.0, 56.9, 56.3, 52.7, 36.0, 35.5, 33.0, 28.2, 19.7, 6.9, 5.2; HRMS (ESI) *m/z* for $C_{18}H_{25}ClN_2O$ $[M+H]^+$ calcd. 321.1728, measured 321.1732; IR (ν/cm^{-1}, thin film) 2934, 2857, 1472, 1442, 1091, 1066, 754.

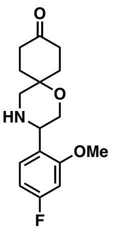

3-(4-fluoro-2-methoxyphenyl)-1-oxa-4-azaspiro[5.5]undecan-9-one 28

4-Fluoro-2-methoxybenzaldehyde (77 mg, 0.50 mmol, 1.0 equiv) and iSnAP resin **1** were reacted according to the General Procedure. The ketone was purified using the generic HPLC method to afford the title compound (39 mg, 0.13 mmol, 27% yield); ^{1}H NMR (400 MHz, $CDCl_3$) δ 7.48 (dd, *J* = 8.5, 6.8 Hz, 1H), 6.72 – 6.58 (m, 2H), 4.28 (dd, *J* = 10.0, 3.5 Hz, 1H), 3.88 – 3.76 (m, 4H), 3.58 (dd, *J* = 11.4, 10.0 Hz, 1H), 3.04 – 2.86 (m, 3H), 2.80 – 2.66 (m, 1H), 2.57 – 2.43 (m, 1H), 2.35 – 2.20 (m, 2H), 2.07 – 1.94 (m, 1H), 1.76 (td, *J* = 13.3, 5.2 Hz, 1H), 1.65 (ddd, *J* = 14.4, 13.4, 4.5 Hz, 1H), exchangeable NH x 1 not observed; ^{13}C NMR (126 MHz, $CDCl_3$) δ 211.9, 163.1 (d, *J* = 245.6 Hz), 158.12 (d, *J* = 9.7 Hz), 128.52 (d, *J* = 9.9 Hz), 123.5, 106.94 (d, *J* = 20.9 Hz), 98.85 (d, *J* = 25.8 Hz), 69.6, 65.4, 55.6, 54.6, 54.0, 36.4, 36.1, 35.8, 28.8; HRMS (ESI) *m/z* for $C_{16}H_{20}FNO_3$ $[M+H]^+$ calcd. 294.1500, measured 294.1497; IR (ν/cm^{-1}, thin film) 2851, 1750, 1607, 1500, 1412, 1117, 952.

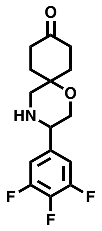

3-(3,4,5-trifluorophenyl)-1-oxa-4-azaspiro[5.5]undecan-9-one 29

3,4,5-trifluorobenzaldehyde (80 mg, 0.50 mmol, 1.0 equiv) and iSnAP resin **1** were reacted according to the General Procedure. The ketone was purified using the generic HPLC method to afford the title compound (36 mg, 0.12 mmol, 24% yield); ^{1}H NMR (400 MHz, $CDCl_3$) δ 7.10 (dd, *J* = 8.4, 6.6 Hz, 2H), 3.89 (dd, *J* = 10.3, 3.6 Hz, 1H), 3.68 (dd, *J* = 11.5, 3.7 Hz, 1H), 3.50 (dd, *J* = 11.5, 10.3 Hz, 1H), 3.06 – 2.84 (m, 3H), 2.78 – 2.65 (m, 1H), 2.54 – 2.40 (m, 1H), 2.32 – 2.23 (m, 2H), 2.02 – 1.93 (m, 1H), 1.89 – 1.60 (m, 3H); ^{13}C NMR (126 MHz, $CDCl_3$) δ 211.7, 151.3 (ddd, *J* = 250.2, 9.9, 4.0 Hz, 2C), 139.1 (dt, *J* = 251.6, 15.4 Hz), 137.06 – 136.41 (m), 111.66 – 110.40 (m, 2C), 70.0, 67.3, 59.5, 54.3, 36.3, 36.1, 35.8, 27.9; HRMS (ESI) *m/z* for $C_{15}H_{16}F_3NO_2$ $[M+H]^+$ calcd. 300.1206, measured 300.1202; IR (ν/cm^{-1}, thin film) 2854, 1751, 1529, 1446, 1116.

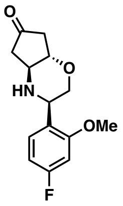

(3*R*,4a*S*,7a*S*)-3-(4-fluoro-2-methoxyphenyl)hexahydrocyclopenta[*b*][1,4]oxazin-6(2*H*)-one 30

4-fluoro-2-methoxybenzaldehyde (77 mg, 0.50 mmol, 1.0 equiv) and iSnAP resin **3** were reacted according to the General Procedure. The ketone was purified using the generic HPLC method to afford the title compound (26 mg, 0.10 mmol, 20% yield); ^{1}H NMR (400 MHz, $CDCl_3$) δ 7.52 (ddd, *J* = 8.5, 6.7, 0.6 Hz, 1H), 6.72 – 6.56 (m, 2H), 4.56 (dd, *J* = 10.3, 3.3 Hz, 1H), 4.07 (dd, *J* = 11.3, 3.4 Hz, 1H), 3.85 (s, 3H), 3.80 – 3.70 (m, 1H), 3.56 (t, *J* = 10.9 Hz, 1H), 3.28 (ddd, *J* = 13.0, 8.9, 7.2 Hz, 1H), 2.67 (ddt, *J* = 17.6, 7.4, 0.9 Hz, 1H), 2.61 – 2.52 (m, 1H), 2.43 – 2.21 (m, 2H), exchangeable NH x 1 not observed; ^{13}C NMR (126 MHz, $CDCl_3$) δ 209.7, 164.4, 161.9. 157.9 (d, *J* = 9.7 Hz), 128.5 (d, *J* = 9.9 Hz), 107.0 (d, *J* = 20.9 Hz), 98.8 (d, *J* = 25.9 Hz), 79.4, 72.5, 60.2, 55.6, 54.1, 43.8, 43.1; HRMS (ESI) *m/z* for $C_{14}H_{16}FNO_3$ $[M+H]^+$ calcd. 266.1187, measured 266.1183; IR (ν/cm^{-1}, thin film) 2940, 2861, 1708, 1606, 1500, 1275, 1146, 1032, 834; $[\alpha]^{25}$ +15.94° (c 2.60 $CDCl_3$).

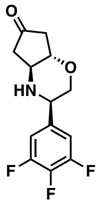

(3*R*,4a*S*,7a*S*)-3-(3,4,5-trifluorophenyl)hexahydrocyclopenta[*b*][1,4]oxazin-6(2*H*)-one 31

3,4,5-trifluorobenzaldehyde (80 mg, 0.50 mmol, 1.0 equiv) and iSnAP resin **3** were reacted according to the General Procedure. The ketone was purified using the generic HPLC method to afford the title compound (36 mg, 0.13 mmol, 27% yield); ^{1}H NMR (400 MHz, $CDCl_3$) δ 7.12 (dd, *J* = 8.3, 6.6 Hz, 2H), 4.13 (dd, *J* = 10.5, 3.5 Hz, 1H), 3.93 (dd, *J* = 11.5, 3.5 Hz, 1H), 3.72 (ddd, *J* = 12.1, 8.9, 7.4 Hz, 1H), 3.50 (dd, *J* = 11.5, 10.5 Hz, 1H), 3.22 (ddd, *J* = 12.9, 8.9, 7.1 Hz, 1H), 2.61 (dddt, *J* = 31.3, 17.4, 7.2, 0.9 Hz, 2H), 2.46 – 2.16 (m, 3H); ^{13}C NMR (126 MHz, $CDCl_3$) δ 209.1, 151.3 (ddd, *J* = 250.5, 10.0, 4.0 Hz, 2C), 139.3 (dt, *J* = 251.9, 15.4 Hz), 135.7 (td, *J* = 6.9, 4.5 Hz), 111.4 (d, *J* = 21.9 Hz, 2C), 79.4, 74.0, 59.9, 59.6, 43.8, 42.9; HRMS (ESI) *m/z* for $C_{13}H_{12}F_3NO_2$ $[M+H]^+$ calcd. 272.0893, measured 272.0891; IR (ν/cm^{-1}, thin film) 2854, 1751, 1620, 1529, 1352, 1116, 1044; $[\alpha]^{25}$ +4.70° (c 4.00 CH_2Cl_2).

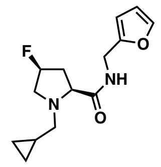

(2*S*,4*S*)-1-(cyclopropylmethyl)-4-fluoro-*N*-(furan-2-ylmethyl)pyrrolidine-2-carboxamide 32

Furan-2-ylmethanamine (48 mg, 0.50 mmol, 1.0 equiv), (2*S*,4*S*)-1-(*tert*-butoxycarbonyl)-4-fluoropyrrolidine-2-carboxylic acid (128 mg, 0.55 mmol, 1.1 equiv), anhydrous EtOH (2.0 mL), anhydrous CH_2Cl_2 (2.0 mL) and a stirring bar were added to a reaction vial. The vial was inserted into the vial holder of the console and the line cap was attached. The “amide formation” capsule was scanned to load the appropriate program and inserted into the capsule holder.[8] After checking the solvents were full and the waste container was not, the program was initiated by pressing “run” on the touch screen interphase. Upon program completion, the solution in the vial was concentrated using a vial adaptor and standard rotary evaporator.

The reaction vial was returned to the vial holder of the console and the line cap was attached. A “Boc deprotection” capsule was scanned and inserted into the capsule holder.[9] The program was initiated by pressing “run” on the touch screen interphase. Upon program completion, the solution in the vial was concentrated using a vial adaptor and standard rotary evaporator.

Cyclopropanecarbaldehyde (35 mg, 0.50 mmol, 1.0 equiv), *i*-Pr_2NEt (174 μL, 1.0 mmol, 2.0 equiv.), anhydrous CH_2Cl_2 (4.0 mL) and anhydrous HFIP (1.0 mL) were added to the reaction vial. The reaction vial was returned to the vial holder of the console and the line cap was attached. A “reductive amination” capsule was scanned and inserted into the capsule holder.[10] Reaction program “aldehyde + secondary amine” was selected and initiated by pressing “run” on the touch screen interphase. Upon program completion, the solution in the vial was concentrated and the product purified using the generic HPLC method to afford the title compound (56 mg, 0.21 mmol, 42% yield); ^{1}H NMR (500 MHz, $CDCl_3$) δ 7.77 (s, 1H), 7.34 (dd, *J* = 1.9, 0.9 Hz, 1H), 6.31 (dd, *J* = 3.2, 1.9 Hz, 1H), 6.21 (dq, *J* = 3.2, 0.8 Hz, 1H), 5.19 – 5.05 (m, 1H), 4.55 (dd, *J* = 15.7, 6.6 Hz, 1H), 4.40 – 4.32 (m, 1H), 3.53 (ddd, *J* = 16.9, 11.6, 2.5 Hz, 1H), 3.18 (dd, *J* = 11.2, 4.1 Hz, 1H), 2.62 – 2.39 (m, 3H), 2.38 – 2.33 (m, 1H), 2.26 – 2.14 (m, 1H), 0.84 – 0.75 (m, 1H), 0.48 – 0.40 (m, 2H), 0.10 – 0.02 (m, 2H); ^{13}C NMR (126 MHz, $CDCl_3$) δ 174.0, 151.8, 142.1, 110.5, 107.1, 92.8 (d, *J* = 176.8 Hz), 65.9, 60.3, 60.2 (d, *J* = 20.5 Hz), 38.0 (d, *J* = 22.3 Hz), 36.2, 10.1, 4.2, 3.8; HRMS (ESI) *m/z* for $C_{14}H_{19}FN_2O_2$ $[M+H]^+$ calcd. 267.1503, measured 267.1505; IR (ν/cm^{-1}, thin film) 2988,1668, 1198, 1131, 800, 720, 598; $[\alpha]^{24}$ -111.60° (c 0.90 $CDCl_3$).

[8] Synple Chem, https://www.synplechem.com/solutions/cartridges#AmideSec, (accessed October 2022).

[9] Synple Chem, https://www.synplechem.com/solutions/cartridges#Bocdeprotsec (accessed October 2022).

[10] Synple Chem, https://www.synplechem.com/solutions/cartridges#redamin (accessed October 2022).

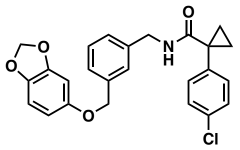

N-(3-((benzo[d][1,3]dioxol-5-yloxy)methyl)benzyl)-1-(4-chlorophenyl)cyclopropane-1-carboxamide 33

(3-(((*tert*-butyldimethylsilyl)oxy)methyl)phenyl)methanamine (126 mg, 0.50 mmol, 1.0 equiv), 1-(4-chlorophenyl)cyclopropane-1-carboxylic acid (98 mg, 0.55 mmol, 1.1 equiv), anhydrous EtOH (2.0 mL), anhydrous CH_2Cl_2 (2.0 mL) and a stirring bar were added to a reaction vial. The vial was inserted into the vial holder of the console and the line cap was attached. The “amide formation” capsule was scanned to load the appropriate program and inserted into the capsule holder.[8] After checking the solvents were full and the waste container was not, the program was initiated by pressing “run” on the touch screen interphase. Upon program completion, the solution in the vial was concentrated using a vial adaptor and standard rotary evaporator.

The reaction vial was returned to the vial holder of the console, MeOH (2.0 mL) was added and the line cap was attached. A “silyl deprotection” capsule was scanned and inserted into the capsule holder.[11] The program was initiated by pressing “run” on the touch screen interphase. Upon program completion, the solution in the vial was concentrated using a vial adaptor and standard rotary evaporator.

Benzo[*d*][1,3]dioxol-5-ol (104 mg, 0.75 mmol, 1.5 equiv) was added to the reaction vial. The reaction vial was returned to the vial holder of the console and the line cap was attached. A “Mitsunobu” capsule was scanned and placed into the capsule holder.[12] Reaction program “Mitsunobu standard” was selected and initiated by pressing “run” on the touch screen interphase. Upon program completion, the solution in the vial was concentrated and the product purified using the generic HPLC method to afford the title compound (113 mg, 0.26 mmol, 52% yield); ^{1}H NMR (500 MHz, $CDCl_3$) δ 7.39 – 7.27 (m, 6H), 7.22 – 7.18 (m, 1H), 7.12 – 7.09 (m, 1H), 6.71 (d, *J* = 8.5 Hz, 1H), 6.54 (d, *J* = 2.5 Hz, 1H), 6.37 (dd, *J* = 8.5, 2.5 Hz, 1H), 5.92 (s, 2H), 5.61 – 5.56 (m, 1H), 4.94 (s, 2H), 4.39 (d, *J* = 5.9 Hz, 2H), 1.67 – 1.63 (m, 2H), 1.06 – 1.03 (m, 2H); ^{13}C NMR (126 MHz, $CDCl_3$) δ 173.5, 154.3, 148.4, 142.0, 139.0, 138.3, 137.6, 134.1, 132.5 (2C), 129.4 (2C), 129.0, 127.0, 126.5, 126.4, 108.1, 106.2, 101.3, 98.5, 70.9, 43.9, 30.0, 15.9 (2C); HRMS (ESI) *m/z* for $C_{25}H_{22}ClNO_4$ $[M+H]^+$ calcd. 436.1310, measured 436.1313; IR (ν/cm^{-1}, thin film) 3490, 1651, 1485, 1241, 1180, 1099, 1034, 927.

[11] Synple Chem, https://www.synplechem.com/solutions/cartridges#SilylSec, (accessed October 2022).

[12] Synple Chem, https://www.synplechem.com/solutions/cartridges#mitsunobusec, (accessed October 2022).

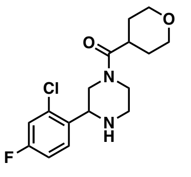

(3-(2-chloro-4-fluorophenyl)piperazin-1-yl)(tetrahydro-2*H*-pyran-4-yl)methanone 34
2-Chloro-4-fluorobenzaldehyde (79 mg, 0.50 mmol, 1.0 equiv) and a stirring bar were added to a reaction vial. The vial was inserted into the vial holder of the console and the line cap was attached to the vial. The "SnAP Piperazine N-Heterocycle formation" capsule was scanned to load the appropriate program and inserted into the capsule holder.[13] After checking the solvents were full and the waste container was not, the program was initiated by pressing "run" on the touch screen interphase. Upon program completion, the solution in the vial was concentrated using a vial adaptor and standard rotary evaporator.

The reaction vial was returned to the vial holder of the console and the line cap was attached. A "Boc deprotection" capsule was scanned and inserted into the capsule holder.[9] The program was initiated by pressing "run" on the touch screen interphase. Upon program completion, the solution in the vial was concentrated using a vial adaptor and standard rotary evaporator.

Tetrahydro-2*H*-pyran-4-carboxylic acid (65 mg, 0.50 mmol, 1.0 equiv), *i*-Pr_2NEt (174 μL, 1.0 mmol, 2.0 equiv.), anhydrous EtOH (2.0 mL), anhydrous CH_2Cl_2 (2.0 mL) and a stirring bar were added to a reaction vial. The vial was inserted into the vial holder of the console and the line cap was attached. An "amide formation" capsule was scanned to load the appropriate program and inserted into the capsule holder.[8] The program was initiated by pressing "run" on the touch screen interphase. Upon program completion, the solution in the vial was concentrated and the product purified using the generic HPLC method to afford the title compound (31 mg, 0.10 mmol, 19% yield). NMR spectroscopy revealed the presence of two partially resolved conformer populations consistent with previous reports on acylated piperazines.[14] This was confirmed using temperature-dependant ^{1}H NMR spectroscopy. NMR characterisation was performed at 328.15 K. ^{1}H NMR (600 MHz, $CDCl_3$, 328.15 K) δ 7.80 – 7.55 (m, 1H), 7.22 – 7.10 (m, 1H), 7.09 – 6.97 (m, 1H), 4.82 – 4.54 (m, 1H), 4.18 – 4.10 (m, 1H), 4.09 – 3.76 (m, 3H), 3.52 – 3.40 (m, 2H), 3.19 (m, 1H), 2.97 – 2.90 (m, 1H), 2.88 – 2.74 (m, 2H), 2.08 – 1.89 (m, 2H), 1.89 – 1.64 (m, 2H), 1.63 – 1.47 (m, 2H); ^{13}C NMR (151 MHz, $CDCl_3$, 328.15 K) δ 172.9, 161.9 (d, *J* = 250.3 Hz), 134.3, 133.3, 129.2, 117.0 (d, *J* = 24.8 Hz), 114.5, 67.2 (2C), 56.8, 51.4, 46.1, 42.0, 37.7, 29.6, 29.0; HRMS (ESI) *m/z* for $C_{16}H_{20}ClFN_2O_2$ $[M+H]^+$ calcd. 327.1270, measured 327.1264; IR (ν/cm^{-1}, thin film) 3298, 2954, 2844, 1634, 1444, 1132, 822.

[13] Synple Chem, https://www.synplechem.com/solutions/cartridges#rclasses, (accessed October 2022).

[14] R. Wodtke, J. Steinberg, M. Köckerling, R. Löser and C. Mamat, *RSC Adv.*, 2018, **8**, 40921.

Relative Stereochemical Assignments

X-Ray Crystallography

Crystallization of 23

Compound **23 DiaA** was crystalized using slow evaporation in EtOAc with 2 equiv of TFA. (CCDC 2064033)

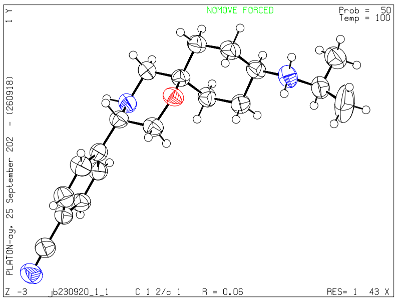

Compound **23 DiaB** was crystalized, as the freebase, using slow evaporation in EtOAc with 2 equiv of TFA. (CCDC 2064034)

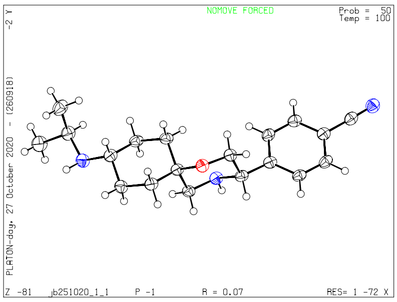

Crystallization of 27

Compound **27 DiaA** was crystalized, as the freebase, using slow evaporation in EtOAc. (CCDC 2169258)

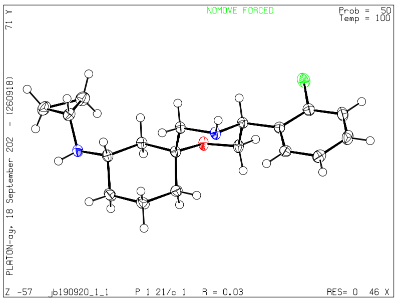

Compound **27 DiaB** was crystalized using slow evaporation in EtOAc with 2 equiv of TFA. (CCDC 2169260)

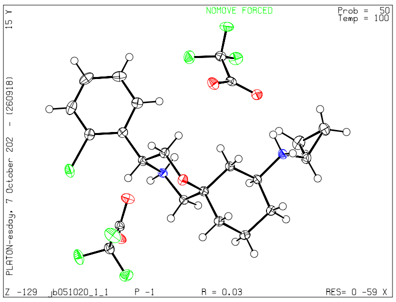

Compound **27 DiaC** formed an oil under slow evaporation in EtOAc, both as the free base and with 2 eq. of TFA.

Compound **27 DiaD** was crystalized using slow evaporation in EtOAc with 2 equiv of TFA. (CCDC 2169267)

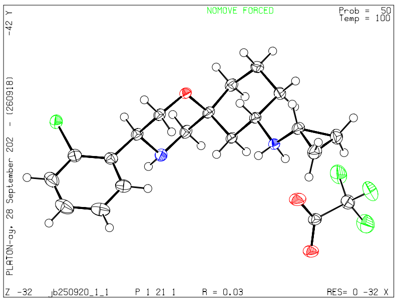

Crystallization of Compound 31

Compound 31 was crystalized using slow evaporation in EtOAc. (CCDC 2169554)

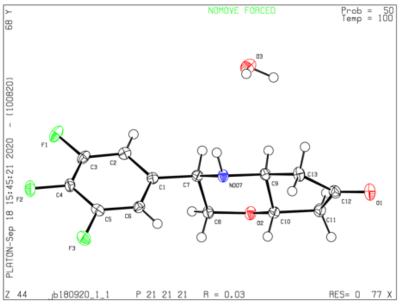

NOE NMR Spectra

NOE Spectrum of 5 DiaA

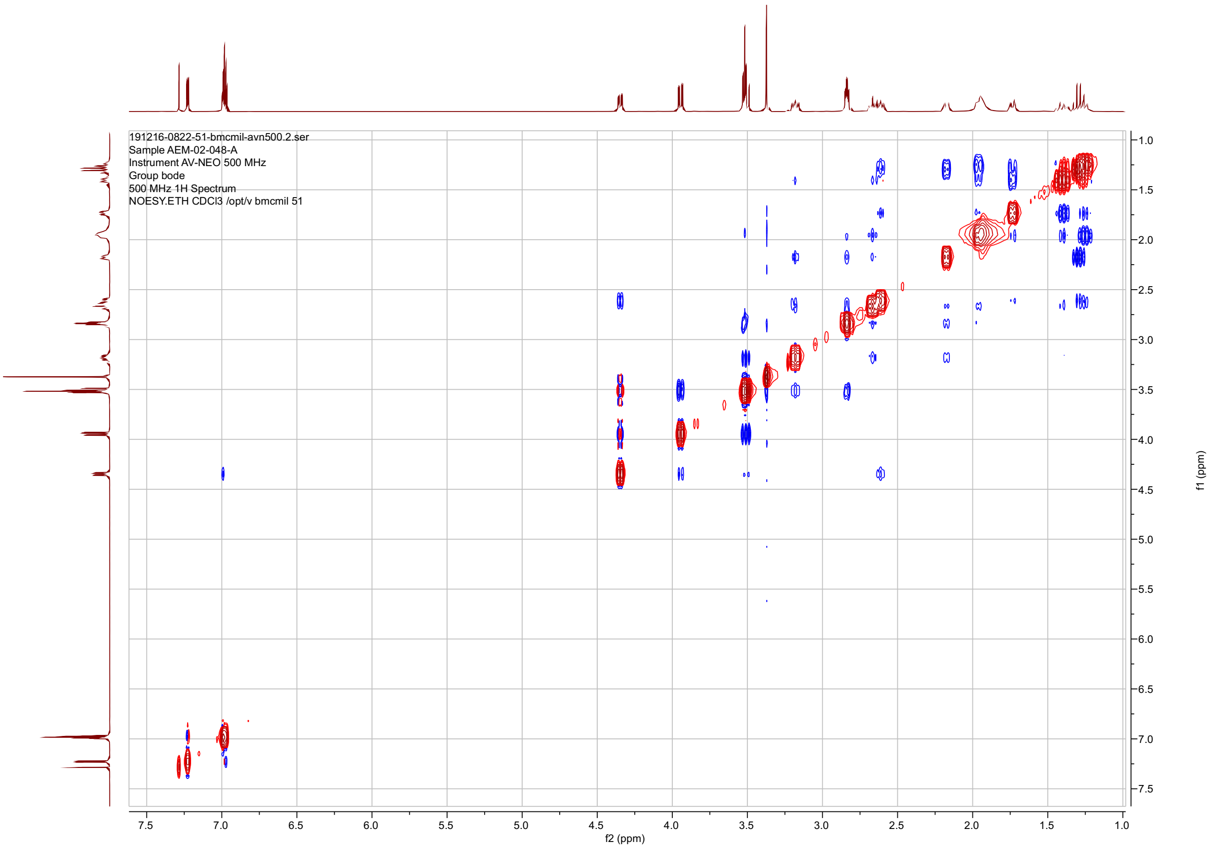

NOE Spectrum of 5 DiaB

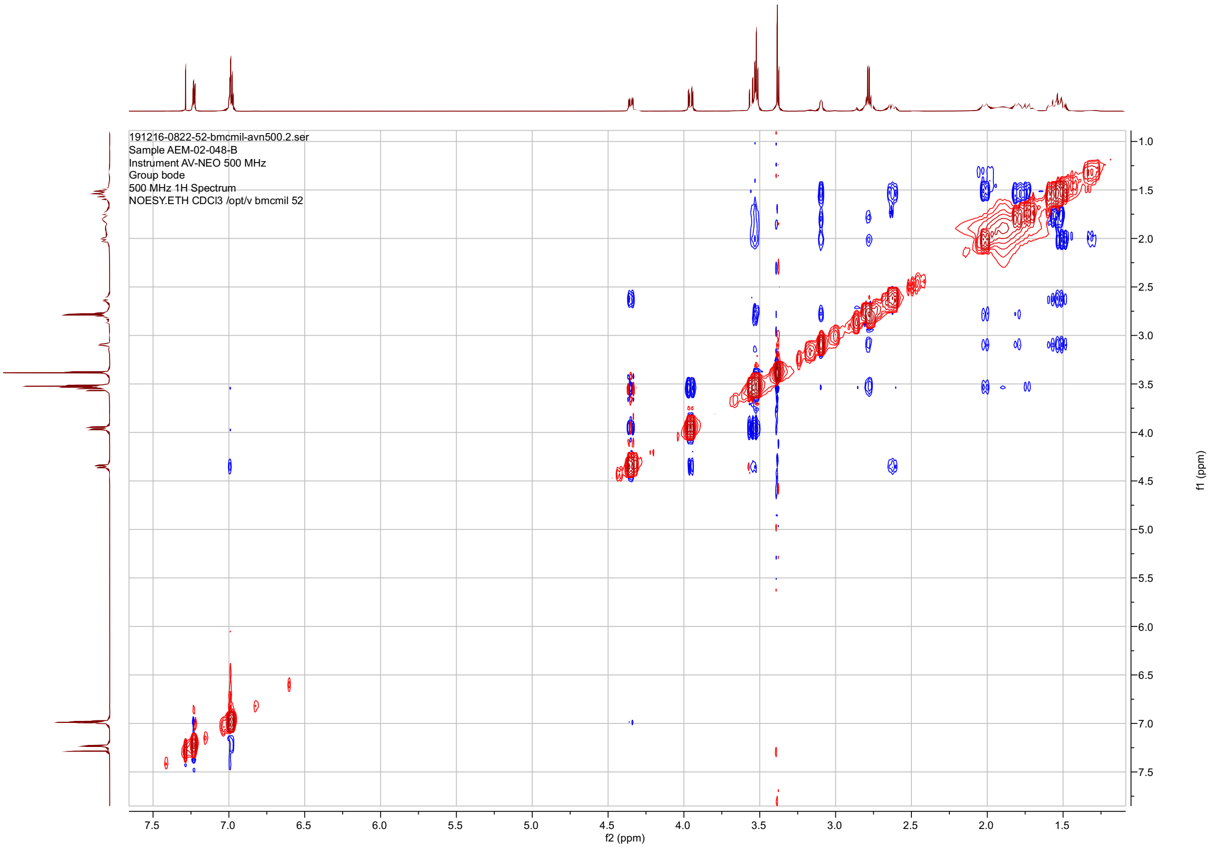

NOE Spectrum of 6 DiaA

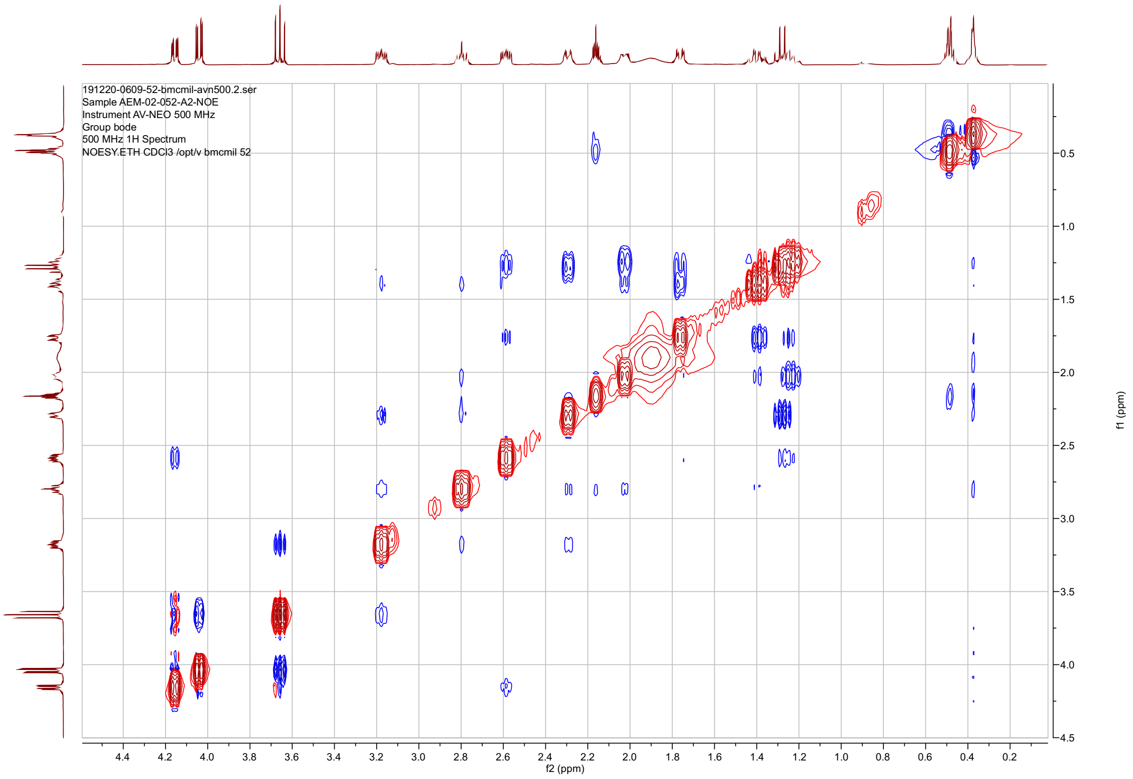

NOE Spectrum of 6 DiaB

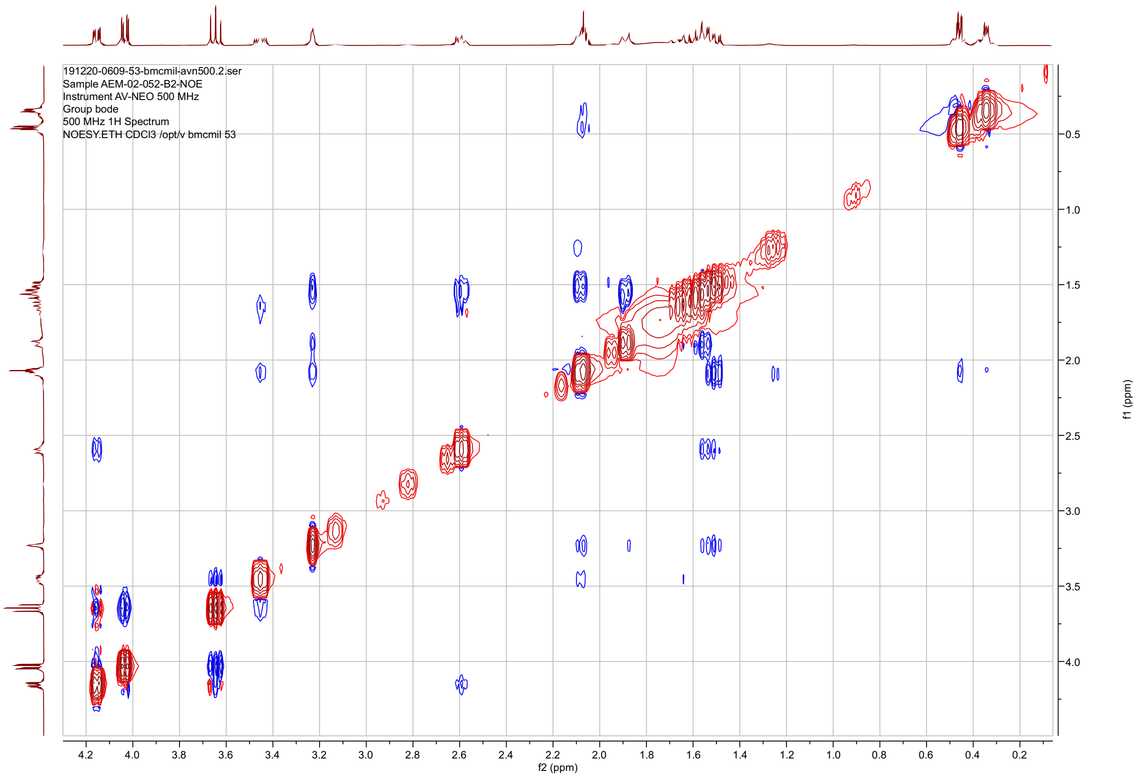

NOE Spectrum of 7 DiaA

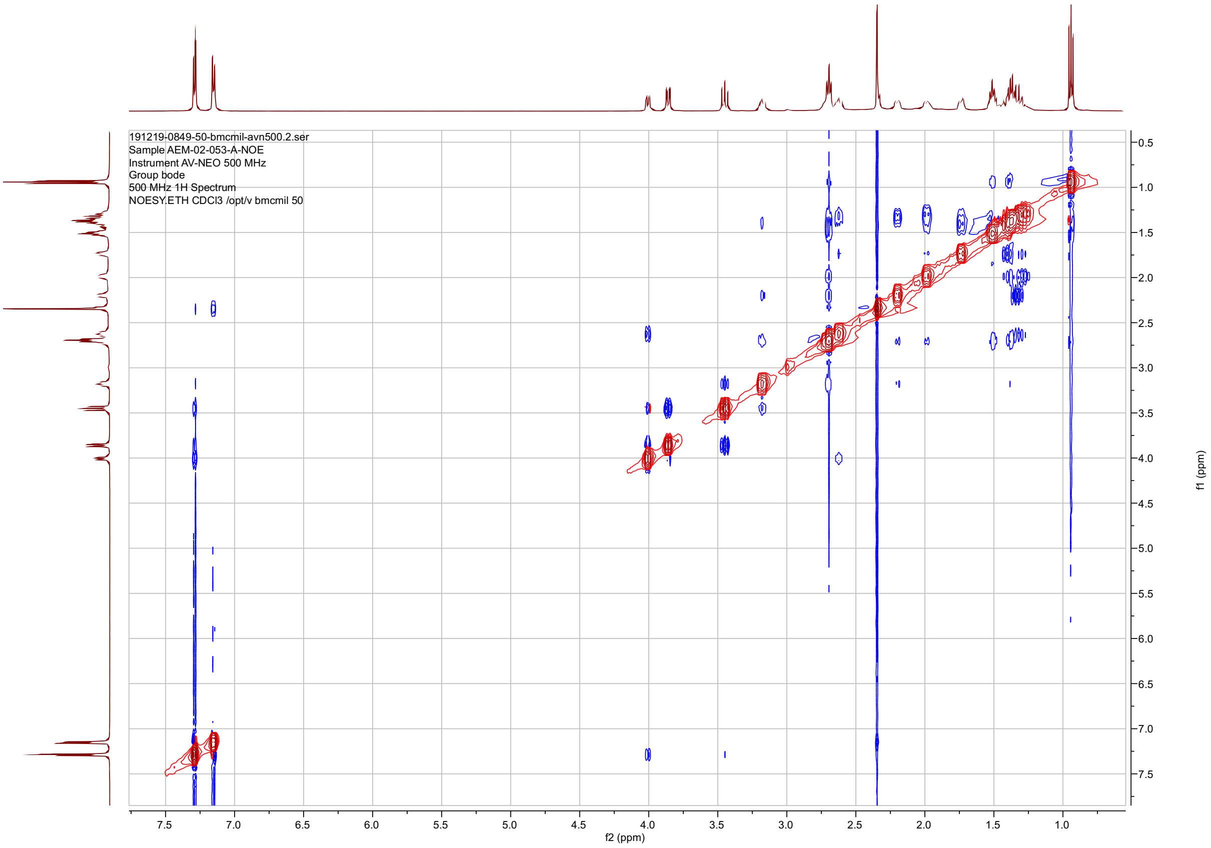

NOE Spectrum of 7 DiaB

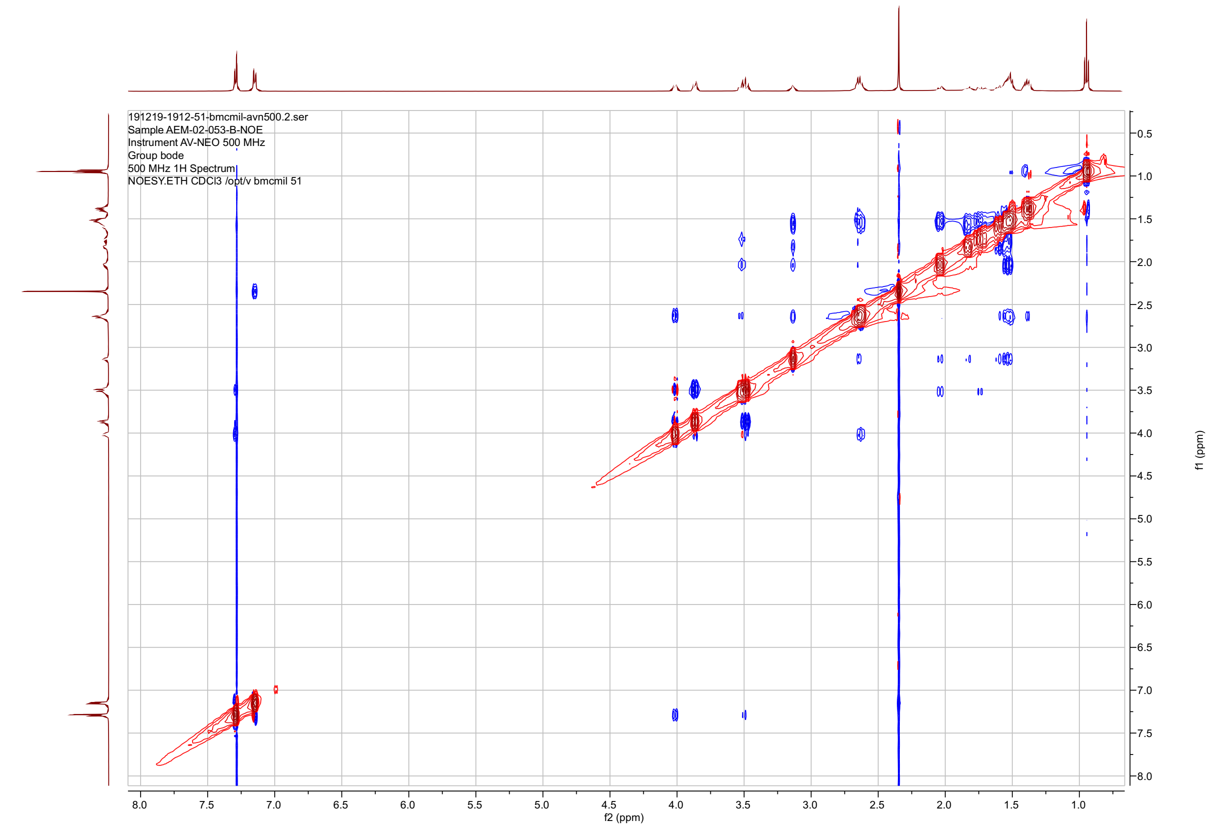

NOE Spectrum of 8 DiaA

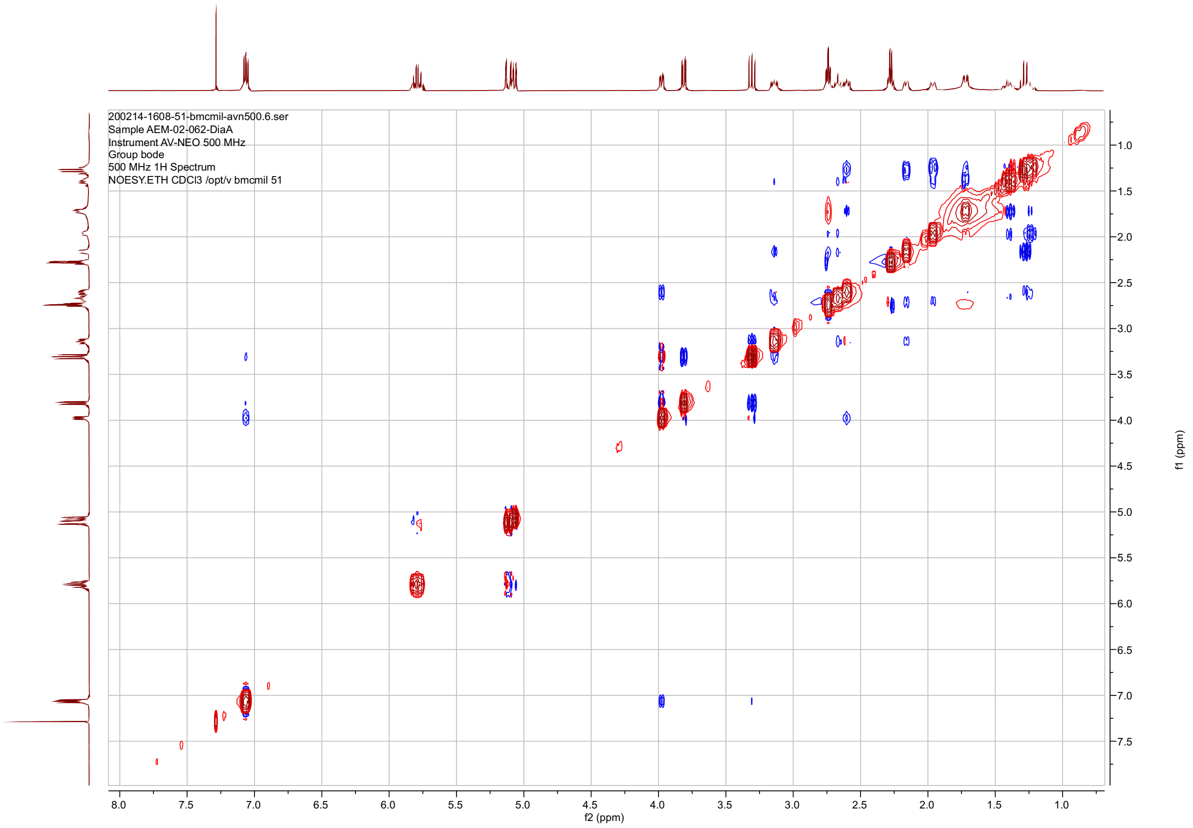

NOE Spectrum of 8 DiaB

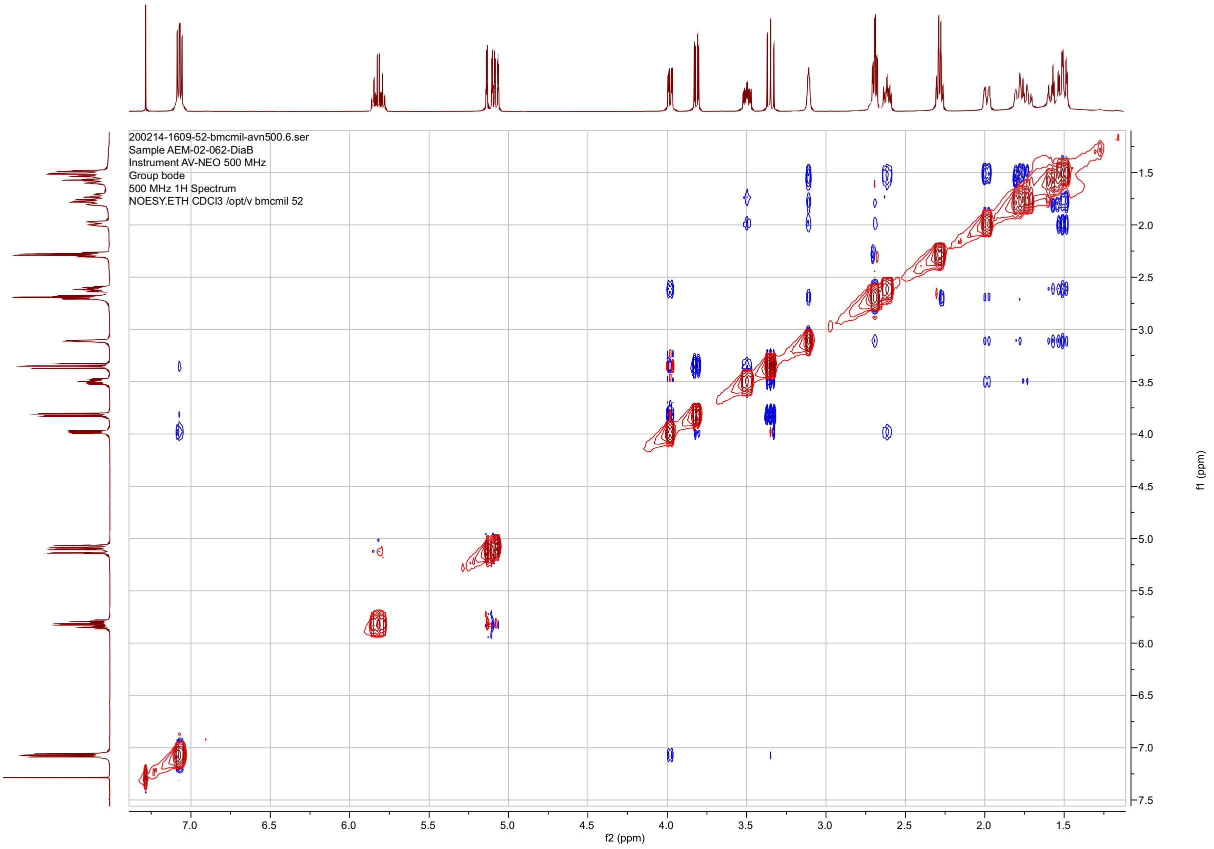

NOE Spectrum of 9

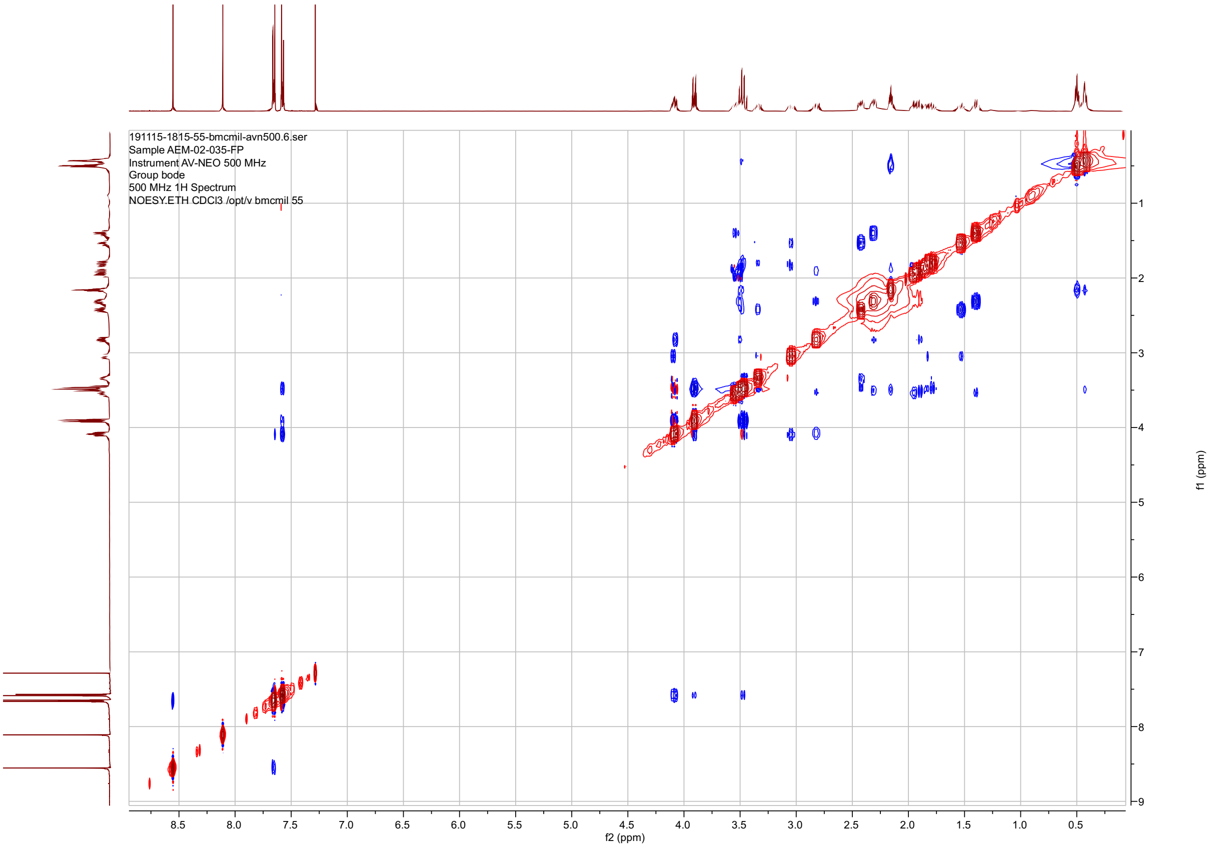

NOE Spectrum of 10

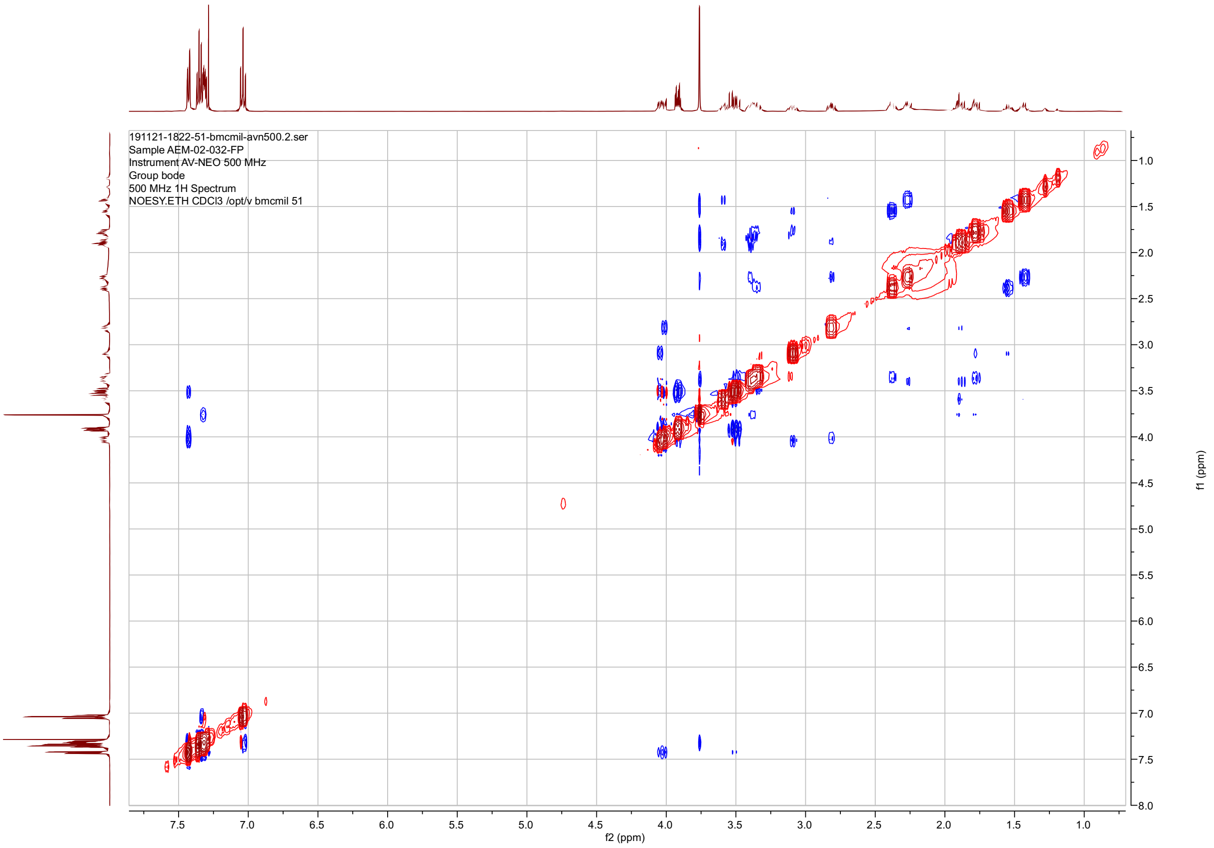

NOE Spectrum of 11

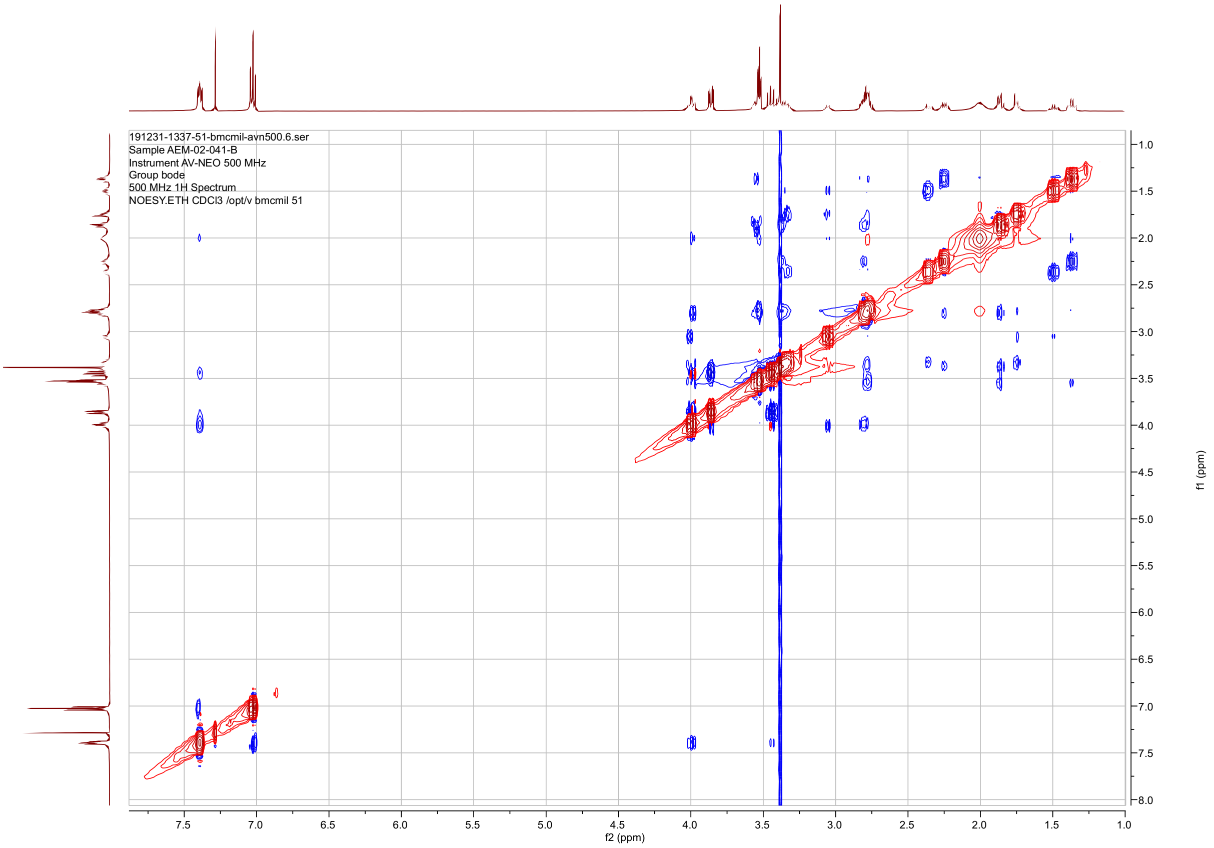

NOE Spectrum of 12

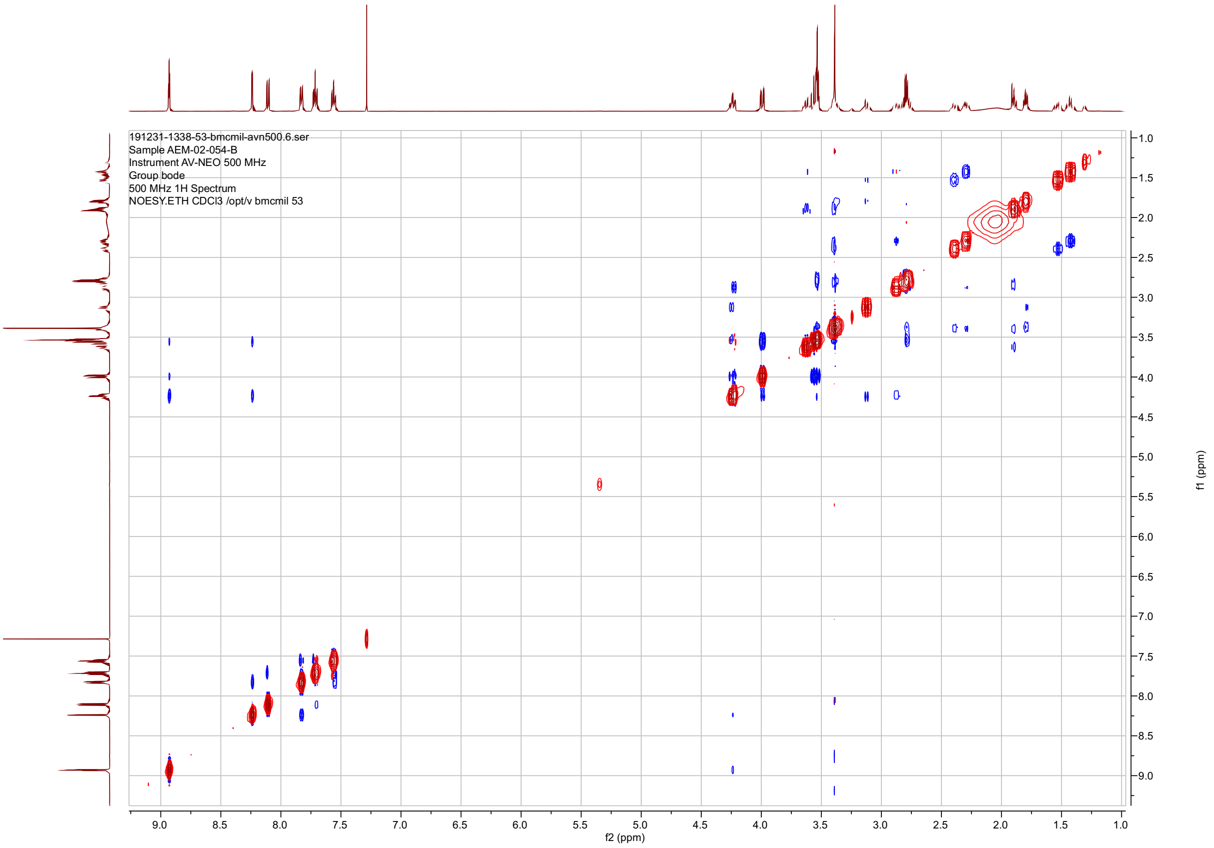

NOE of Spectrum 24

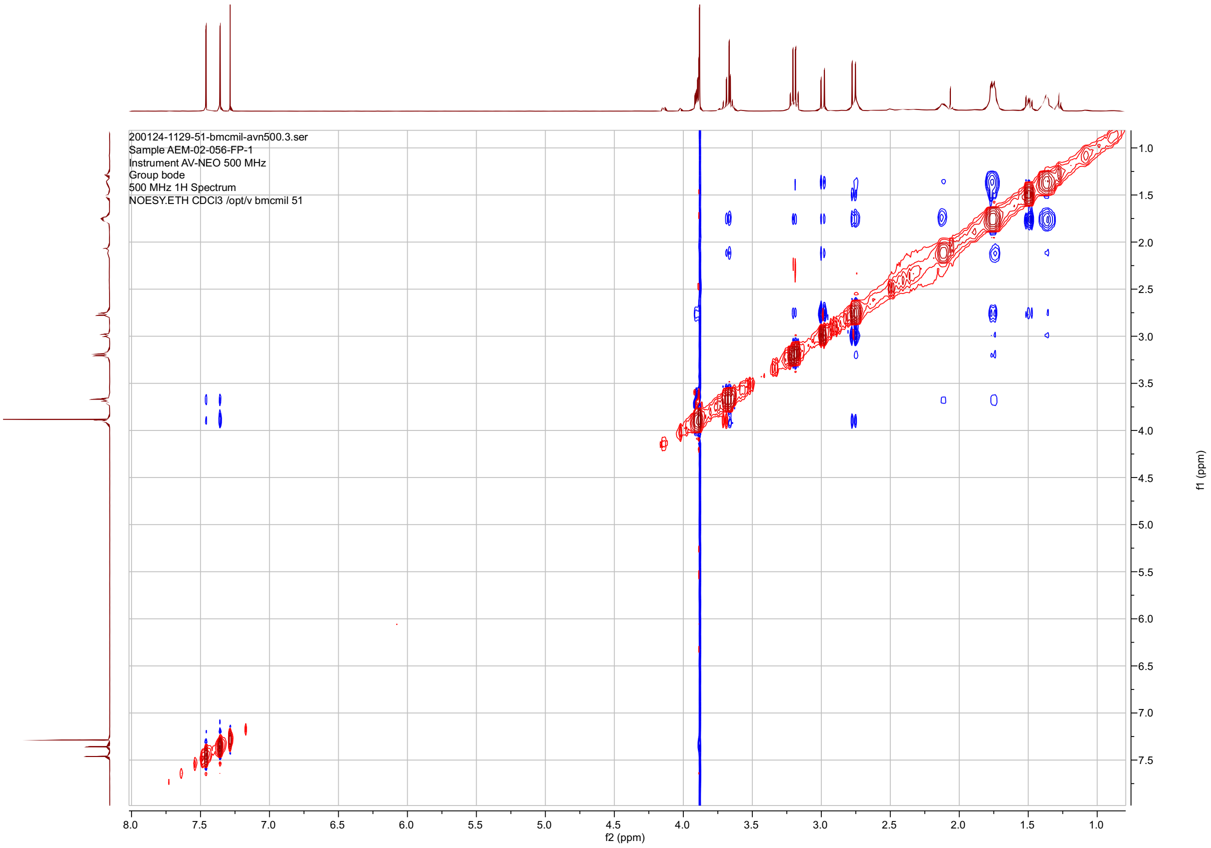

NOE Spectrum of 25

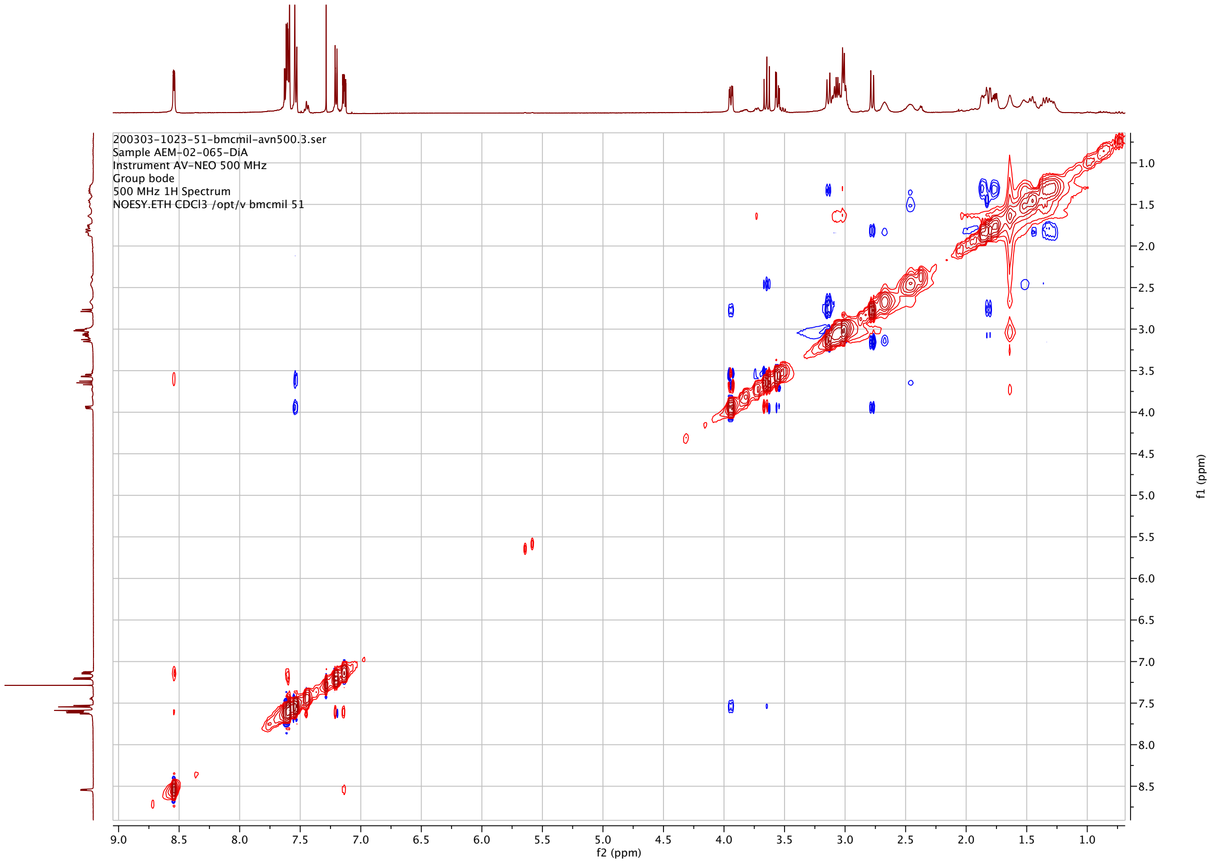

NOE Spectrum of 26

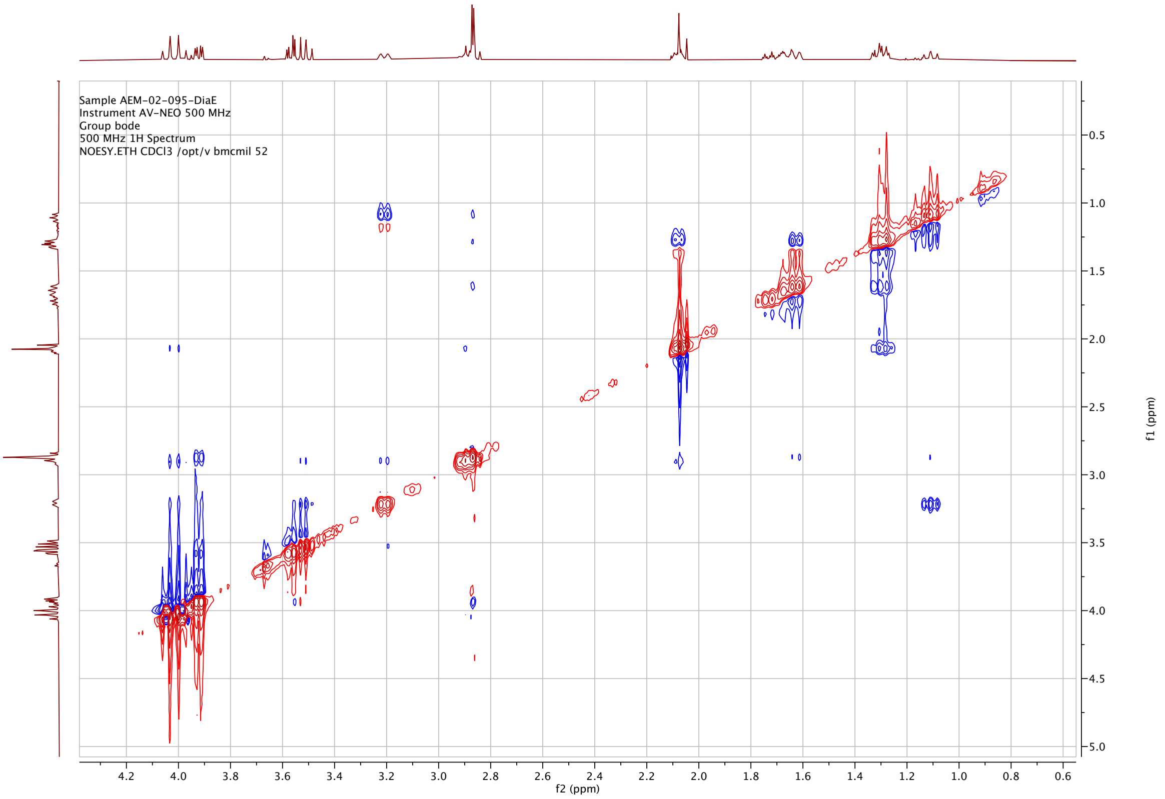

NOE Spectrum of 27 DiaA

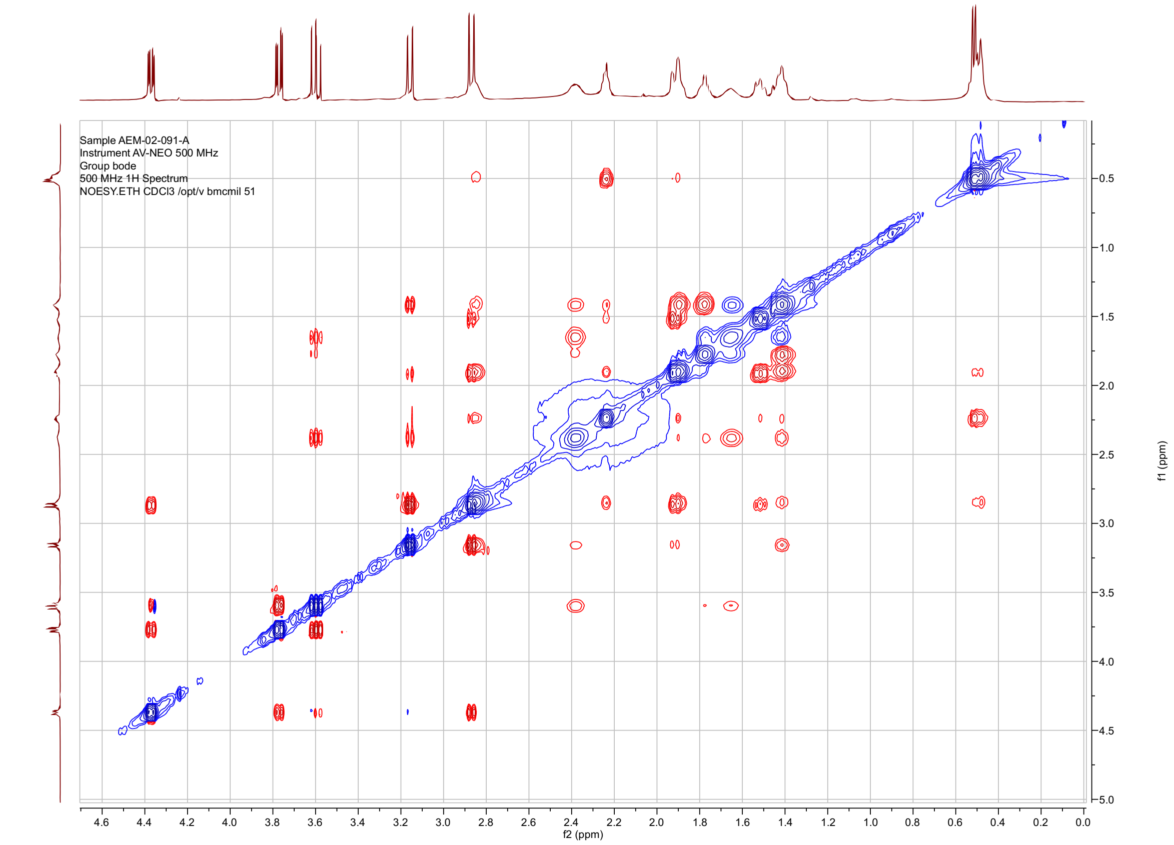

NOE Spectrum of 27 DiaB

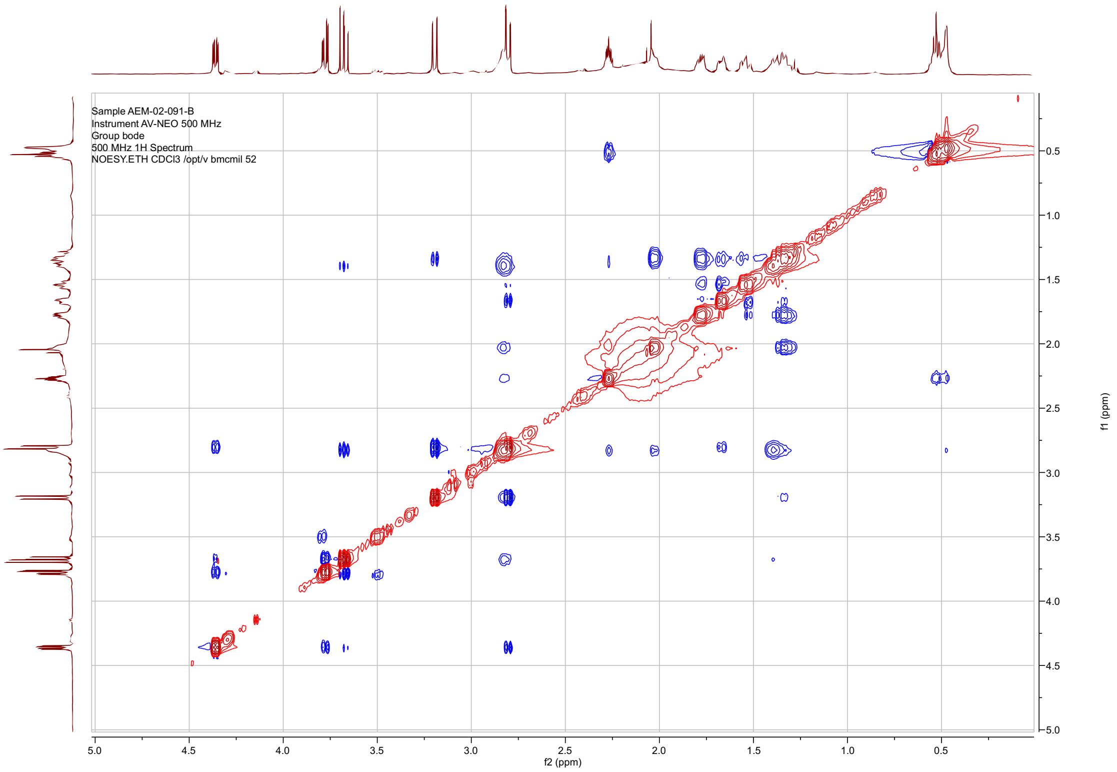

NOE Spectrum of 27 DiaC

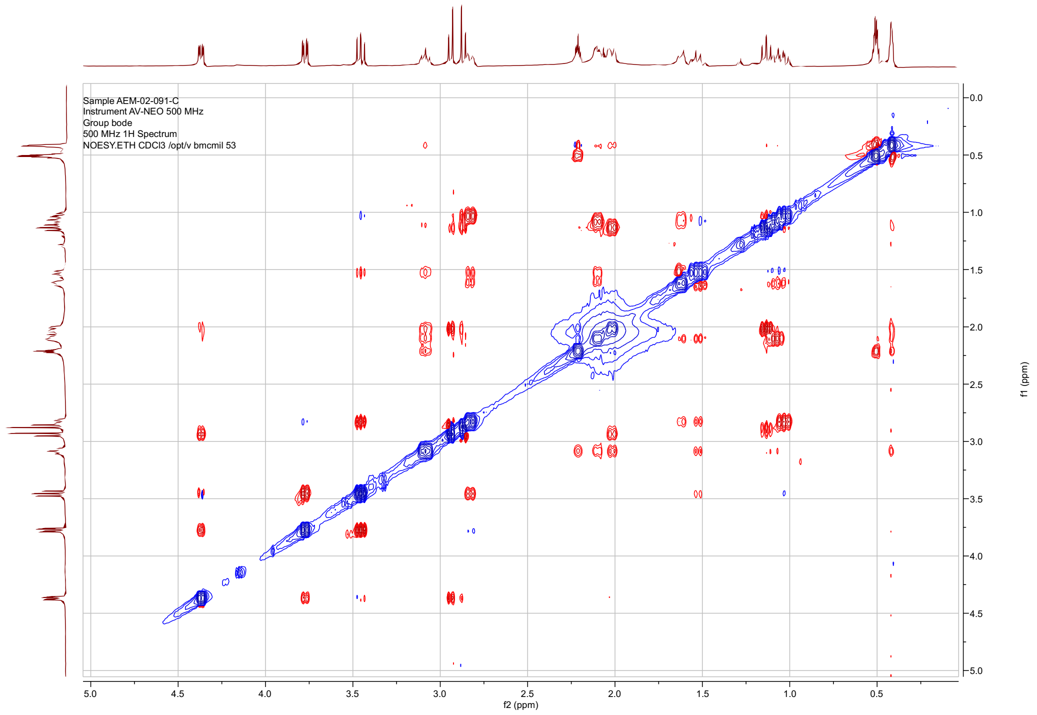

NOE Spectrum of 27 DiaD

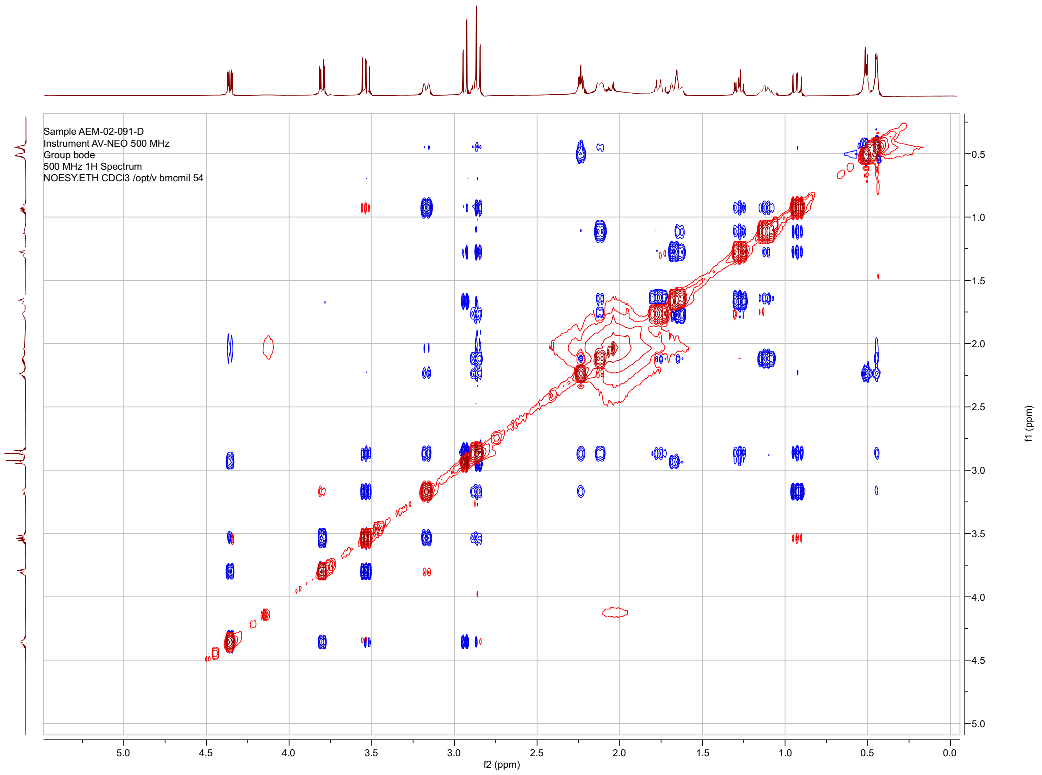

Computational Procedures

Conformational analysis was performed using the MacroModel package.[15] All molecular mechanics calculations were carried out using the OPLS4 force field.[16] Geometry optimizations and NMR predictions were performed using the Jaguar package.[17,18] Conformers were subjected to density functional theory (DFT) geometry optimizations at the B3LYP/6-311G** level of theory with an "ultrafine" integration grid using the polarizable continuum model (PCM) with chloroform as a solvent.

^{13}C NMR Predictions

Conformer searching was performed using torsional sampling.[19] Selected conformers were subject to DFT geometry optimization and NMR prediction as described above. The resulting NMR chemical shifts were Boltzmann weighted and used for comparison to experimental values using corrected mean average error (CMAE).

^{1}H NMR Predictions

The lowest energy conformer was found using force field minimization and subject to DFT geometry optimization and NMR prediction as described above. The predicted chemical shifts were directly compared to experimental values without further manipulation.

---

15 Schrödinger Release 2022-3: MacroModel, Schrödinger, LLC, New York, NY, 2021.

16 C. Lu, C. Wu, D. Ghoreishi, W. Chen, L. Wang, W. Damm, G. A. Ross, M. K. Dahlgren, E. Russell, C. D. Von Bargen, R. Abel, R. A. Friesner, E. D. Harder, *J. Chem. Theory Comput.*, 2021, **17**, 4291.

17 Schrödinger Release 2022-3: Jaguar, Schrödinger, LLC, New York, NY, 2021.

18 A. D. Bochevarov, E. Harder, T. F. Hughes, J. R. Greenwood, D. A. Braden, D. M. Philipp, D. Rinaldo, M. D. Halls, J. Zhang, R. A. Friesner, *Int. J. Quantum Chem.*, 2013, **113**, 2110.

19 P. H. Willoughby, M. J. Jansma, T. R. Hoye, *Nature Protocols*, 2014, **9**, 643.

Compound 15 ^{13}C NMR Prediction

Experimental 13C NMR (ppm)		Predicted 13C NMR (ppm)	
**DiaA**	**DiaB**	**A'**	**B'**
154.36	154.61	156.44	155.25
141.69	141.79	142.05	140.71
138.3	138.33	141.91	136.98
133.41	133.41	134.63	135.29
133.06	133.06	134.51	133.35
128.05	128.04	129.22	129.36
127.83	127.84	128.74	128.61
127.67	127.67	128.47	124.82
126.11	126.11	128.18	124.75
125.8	125.8	126.32	124.51
125.66	125.73	126.27	124.12
125.46	125.47	124.67	120.96
110.11	110.15	110.52	111.43
106.54	106.73	109.75	108.73
70.33	71.9	73.04	71.66
66.94	67.5	65.3	69.45
60.92	61.07	57.15	59.59
56.11	54.61	56.76	53.22
55.86	52.71	53.45	52.96
43.45	43.84	42.88	43.33
35.23	34.69	36.23	39.4
27.71	28.58	30.18	29.92
27.47	28.2	26.27	26.57
26.97	26.04	25.52	26.03

**Assignment**	AA'	AB'	BA'	BB'
**CMAE**	1.39	1.73	1.90	1.66

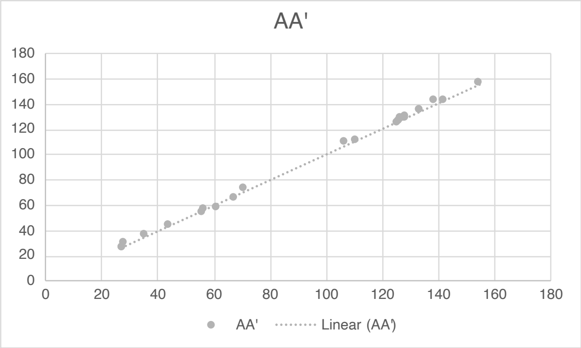

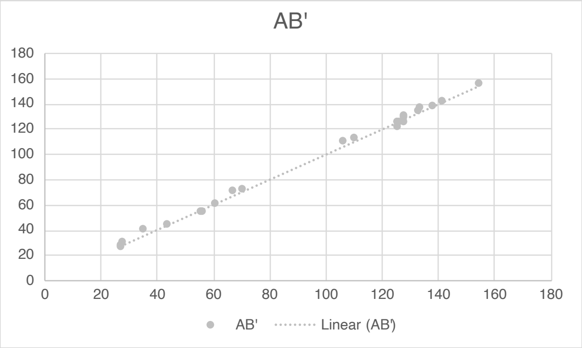

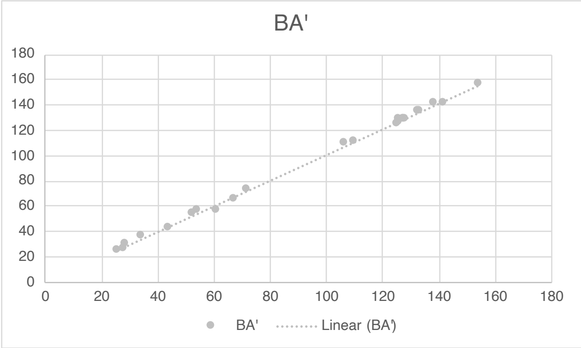

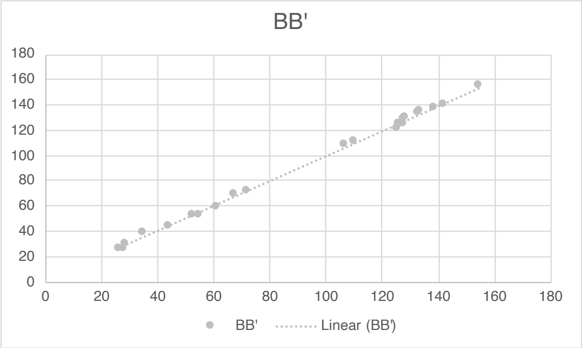

Compounds 13-23 ¹H NMR Prediction

Compound	Experimental Proton 1	Experimental Proton 2	Experimental Δ	Calculated Proton 1	Calculated Proton 2	Calculated Δ
13A	2.85	2.77	0.08	2.93	2.82	0.11
13B	3.25	2.71	0.54	3.41	2.73	0.68
14A	2.86	2.8	0.06	3.03	2.89	0.14
14B	3.25	2.74	0.51	3.33	2.62	0.71
15A	2.95	2.87	0.08	2.91	2.73	0.18
15B	3.29	2.81	0.48	3.51	2.85	0.66
16A	2.9	2.8	0.1	3.03	2.87	0.16
16B	3.26	2.77	0.49	3.48	2.86	0.62
17A	2.96	2.89	0.07	2.93	2.81	0.12
17B	3.31	2.84	0.47	3.58	3	0.58
18A	2.85	2.77	0.08	2.96	2.85	0.11
18B	3.22	2.73	0.49	3.48	2.74	0.74
19A	2.82	2.76	0.06	2.95	2.71	0.24
19B	3.15	2.73	0.42	3.34	2.78	0.56
20A	2.86	2.8	0.06	2.96	2.81	0.15
20B	3.26	2.71	0.55	3.47	2.78	0.69
21A	2.85	2.8	0.05	2.99	2.83	0.16
21B	3.22	2.71	0.51	3.48	2.79	0.69
22A	2.87	2.79	0.08	2.94	2.84	0.1
22B	3.21	2.74	0.47	3.44	2.76	0.68
23A	2.87	2.81	0.06	2.94	2.84	0.1
23B	3.27	2.71	0.56	3.46	2.76	0.7

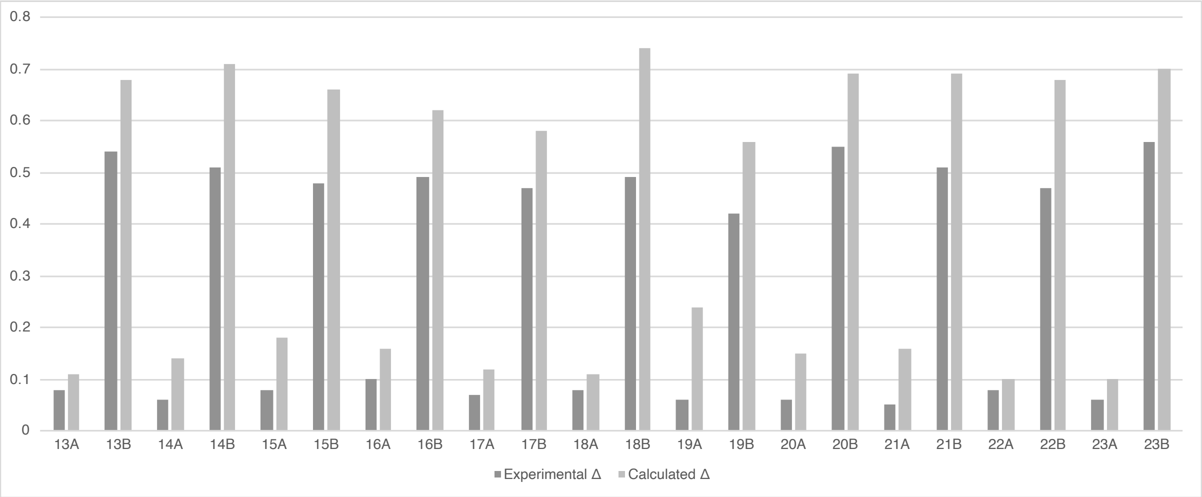

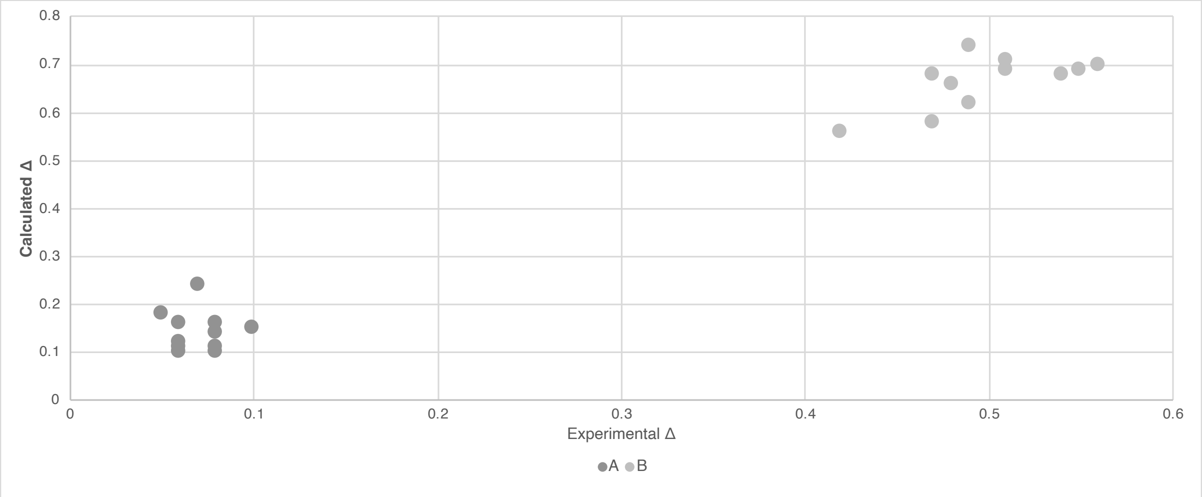

Workflow comparisons

Standard Autoamted Workflow			
Operation	Time min (h)	Time max (h)	User Involvment (yes/no)
Weigh out starting material	0.1		Yes
Insert capsule and start reaction	0.05		Yes
Automated SnAP and Ketone deprotection	15.5	22.5	No
Concentrate ketone	0.3		Yes
Add amine, insert capsule and start reductive amination	0.15		Yes
Automated Reductive Amination	3	12	No
Dry product	0.3		No
Optional add IS and crude NMR	0	0.5	Yes
Preparative HPLC	1		No
Optional freebasing	0	0.5	Yes
Dry Product	1		No
Characterise Product	1		No
Total hours	22.4	38.4	
Total User Hours	0.6	1.6	
Molecules	4	2	
**User Hours per Molecule**	**0.15**	**0.80**	**Average = 0.5**

Automated Workflow with plate Diversification			
Operation	Time min (h)	Time max (h)	User Involvment (yes/no)
Weigh out starting material	0.1		Yes
Insert capsule and start reaction	0.05		Yes
Automated SnAP and Ketone deprotection	15.5	22.5	No
Concentrate ketone	0.3		Yes
Disolve and pipette starting materials	1		Yes
Automated Reductive Amination	3	12	No
Contrafuge and concentrate	1		No
Analysis	1.5		No
Total Hours	22.45	38.45	
Total User Hours	1.45	1.45	
Molecules	40	20	
**User Hours per Molecule**	**0.04**	**0.07**	**Average = 0.06**

Manual Workflow			
Operation	Time min (h)	Time max (h)	User Involvment (yes/no)
Weigh out starting material	0.5		Yes
Set up imine formation	0.5		Yes
Imine formation	5		No
Filter molecular sieves	0.5		Yes
Weigh out and pre-form SnAP catalyst	0.5		Yes
SnAP reaction	5		No
SnAP workup	1		Yes
SnAP column	2		Yes
Dry product	1		No
Set up acetal deprotection	0.5		Yes
Acetal deprotection	5	12	No
Work up acetal deprotection	1		Yes
Optional Column Ketone	2		Yes
Dry ketone	1		No
Set up reductive amination	0.5		Yes
Reductive Amination	3.00	12	No
Work up reductive amination	1		Yes
Dry product	1		No
Optional add IS and crude NMR	0.5		Yes
Preparative HPLC	1		No
Optional freebasing	0	0.5	Yes
Dry product	1		No
Characterise Product	1		No
Total Hours	34.50	50.5	
Total User Hours	10.5	11	
Molecules	4	2	
**User Hours per Molecule**	**2.63**	**5.5**	**Average = 4.1**

NMR Spectra

1-1

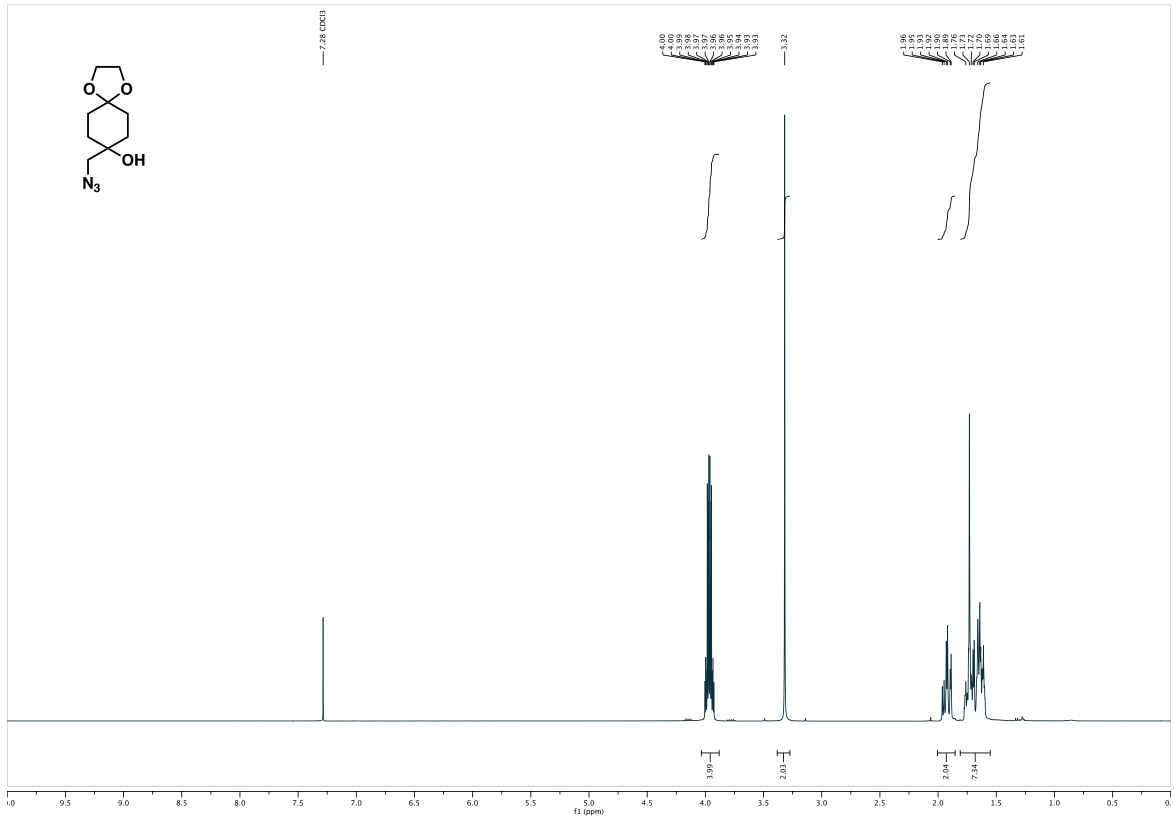

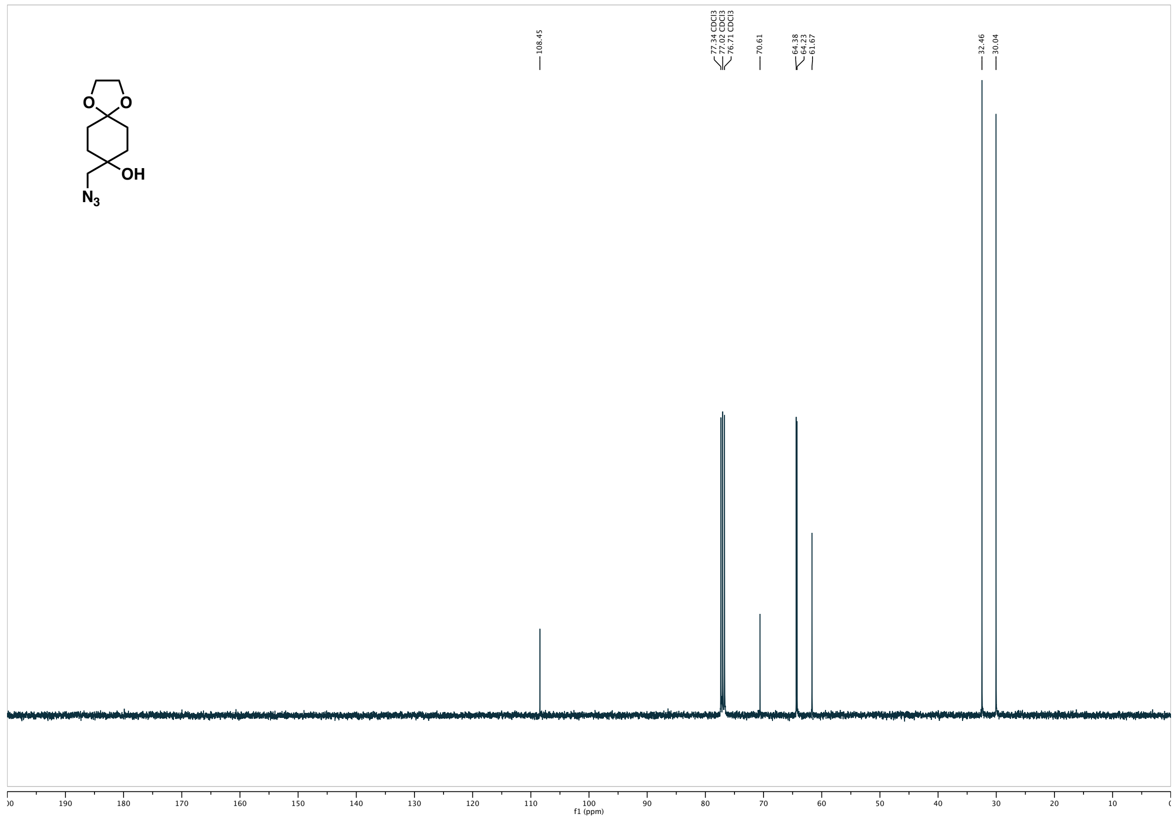

1-2

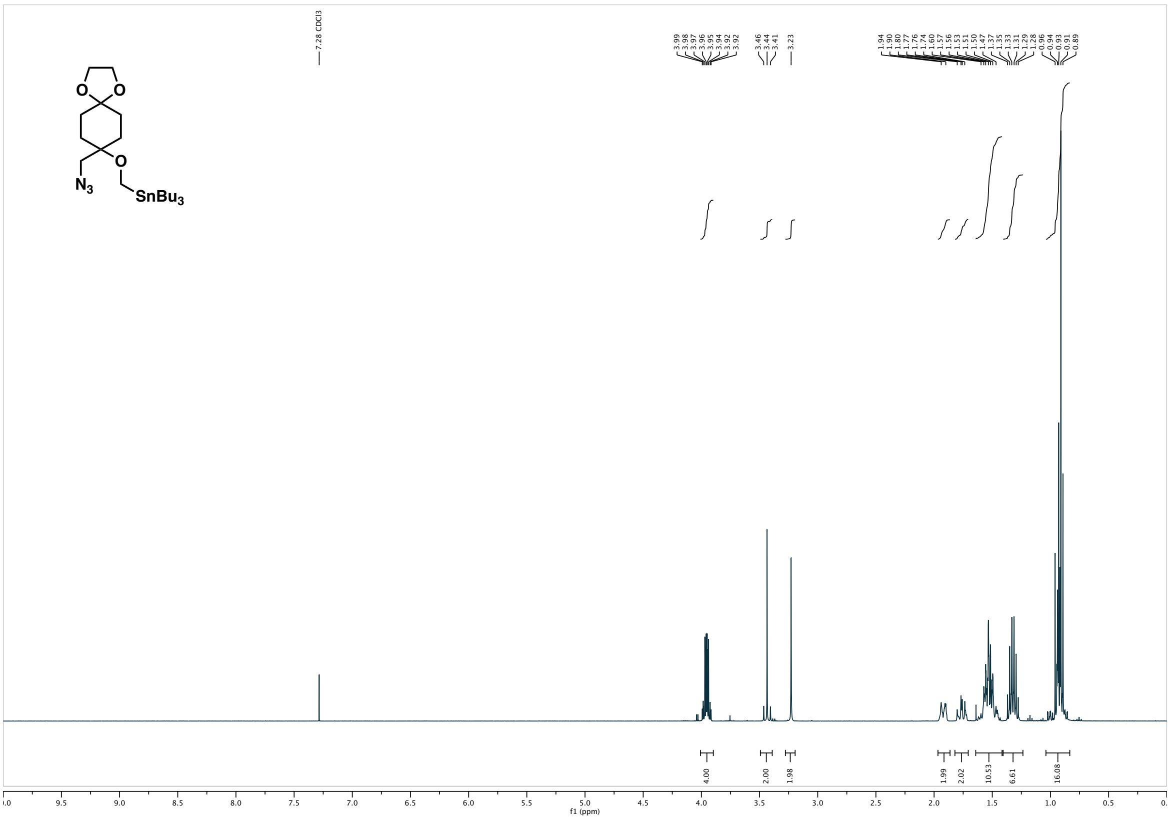
N3
SnBu3
7.28 CDCl3
3.99
3.98
3.97
3.96
3.95
3.94
3.92
3.92
3.46
3.44
3.41
3.23
1.94
1.90
1.80
1.77
1.76
1.74
1.60
1.57
1.56
1.53
1.51
1.50
1.47
1.37
1.35
1.33
1.31
1.29
1.28
0.96
0.94
0.93
0.91
0.89
4.00
2.00
1.98
1.99
2.02
10.53
6.61
16.08
f1 (ppm)

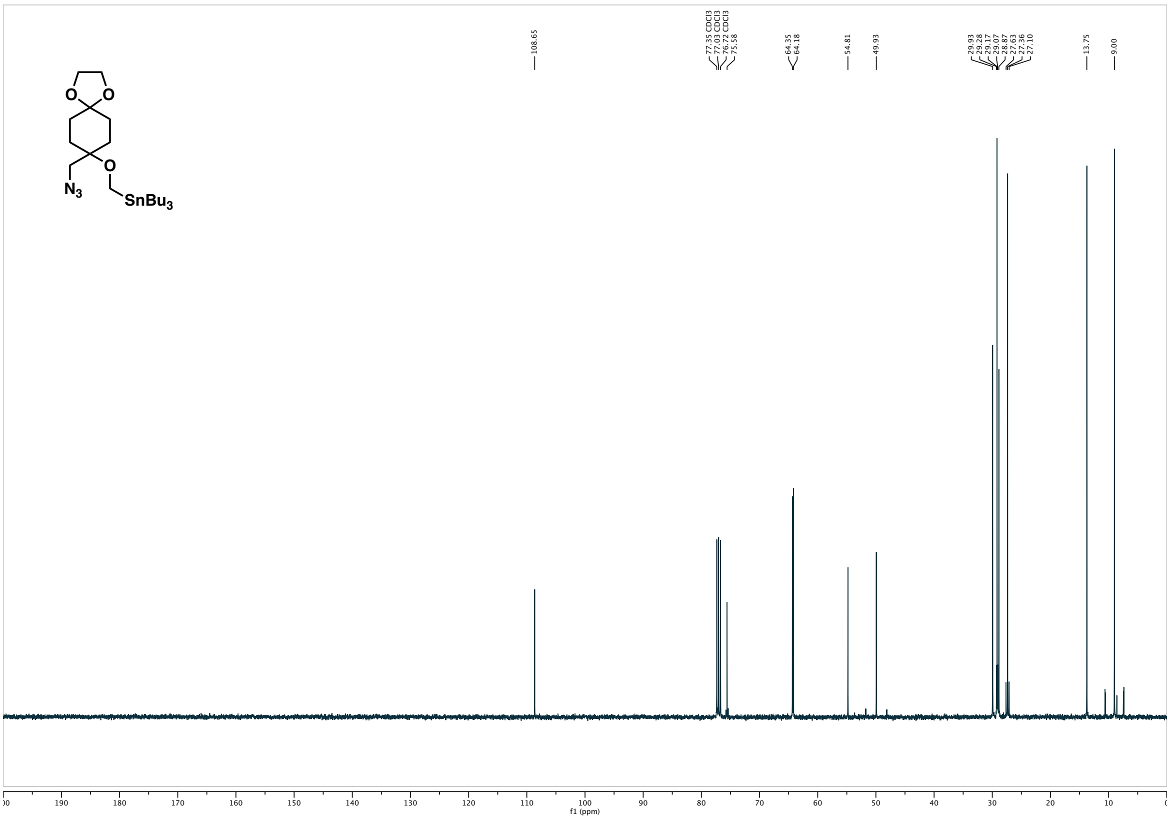
N3
SnBu3
108.65
77.35 CDCl3
77.03 CDCl3
76.72 CDCl3
75.58
64.35
64.18
54.81
49.93
29.93
29.28
29.17
29.07
28.87
27.63
27.36
27.10
13.75
9.00
f1 (ppm)

2-1

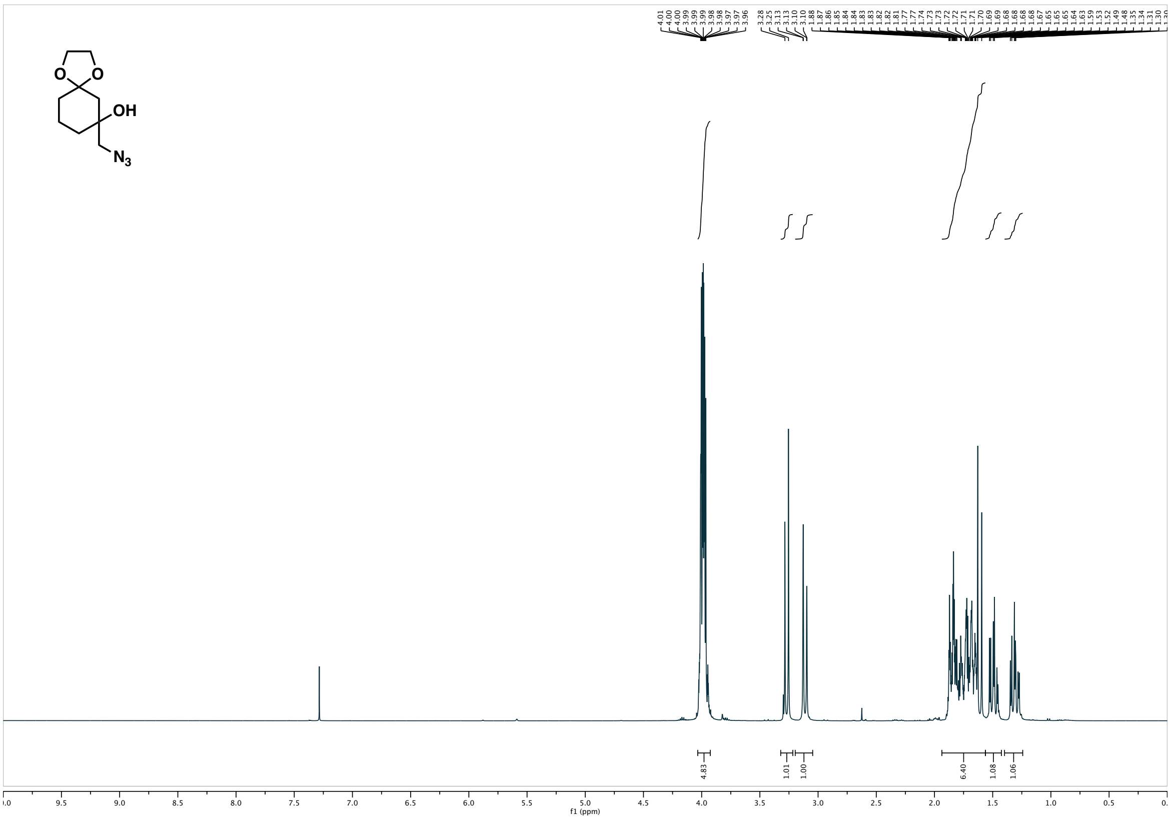
OH
N3
4.83
1.01
1.00
6.40
1.08
1.06
f1 (ppm)

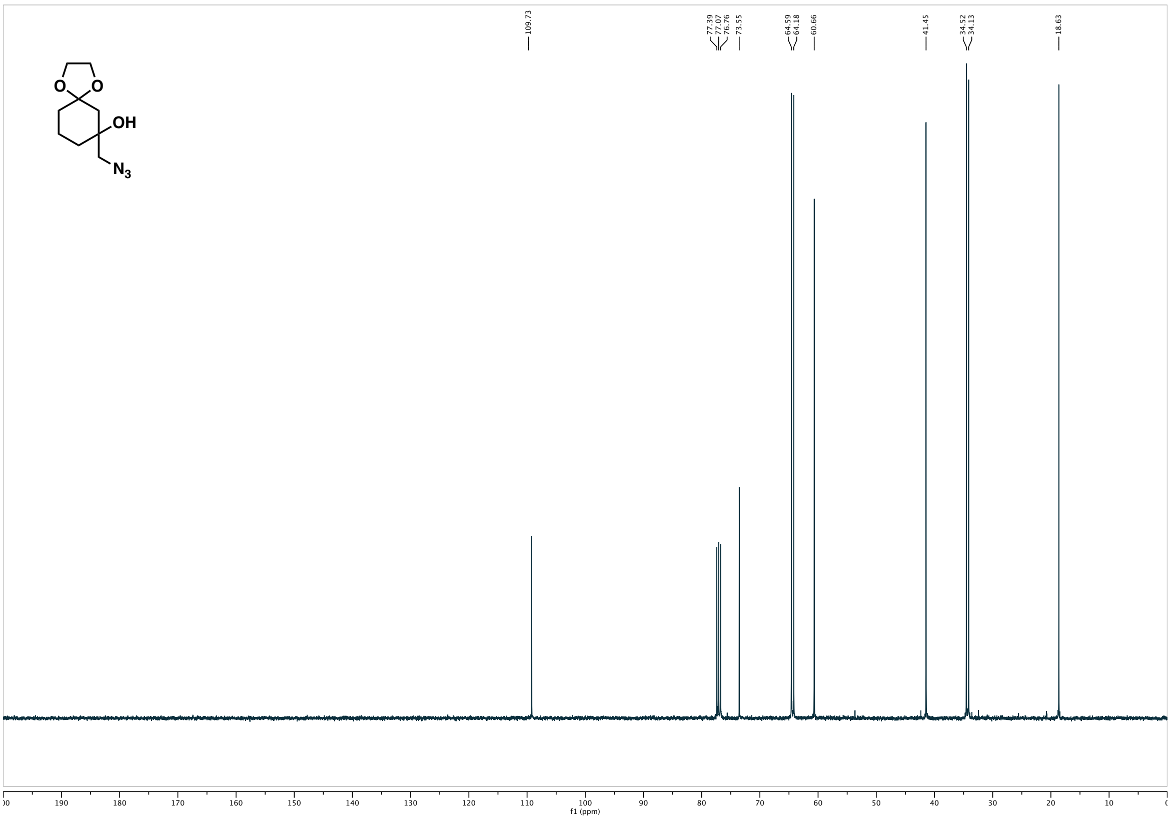
OH
N3
109.73
77.39
77.07
76.76
73.55
64.59
64.18
60.66
41.45
34.52
34.13
18.63
f1 (ppm)

2-2

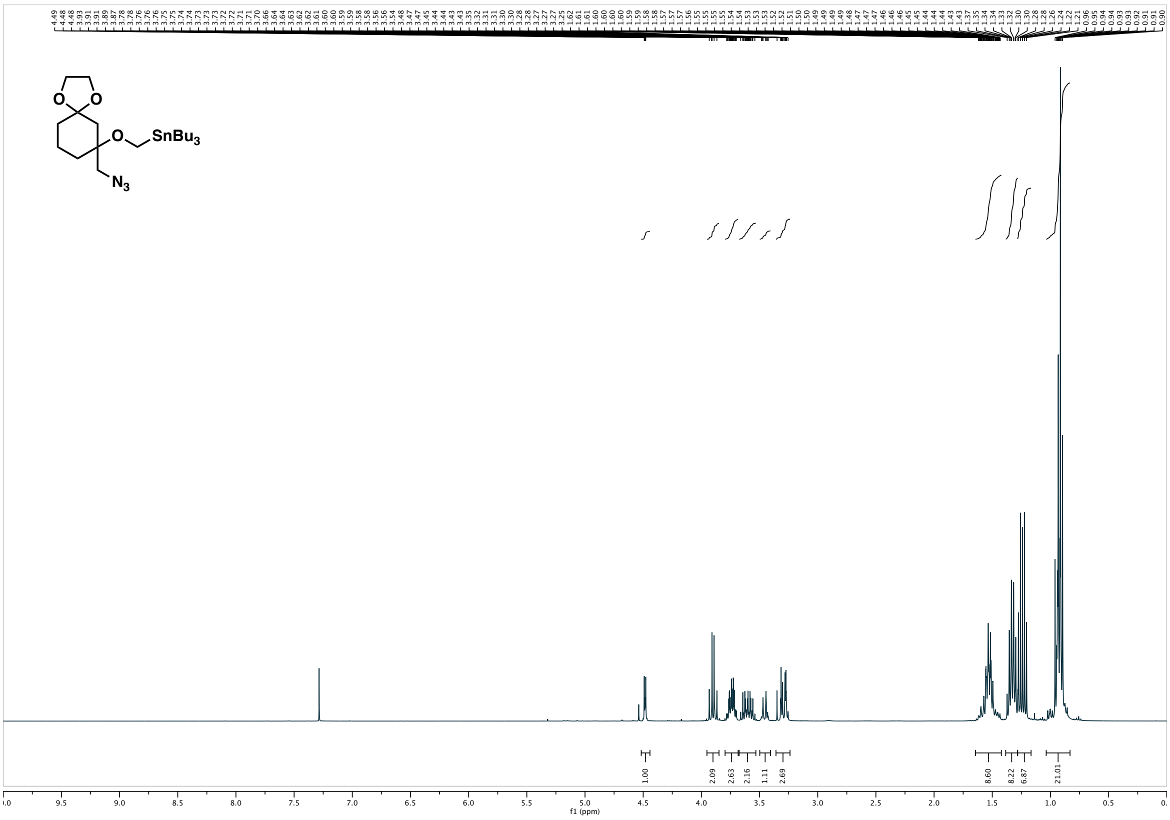

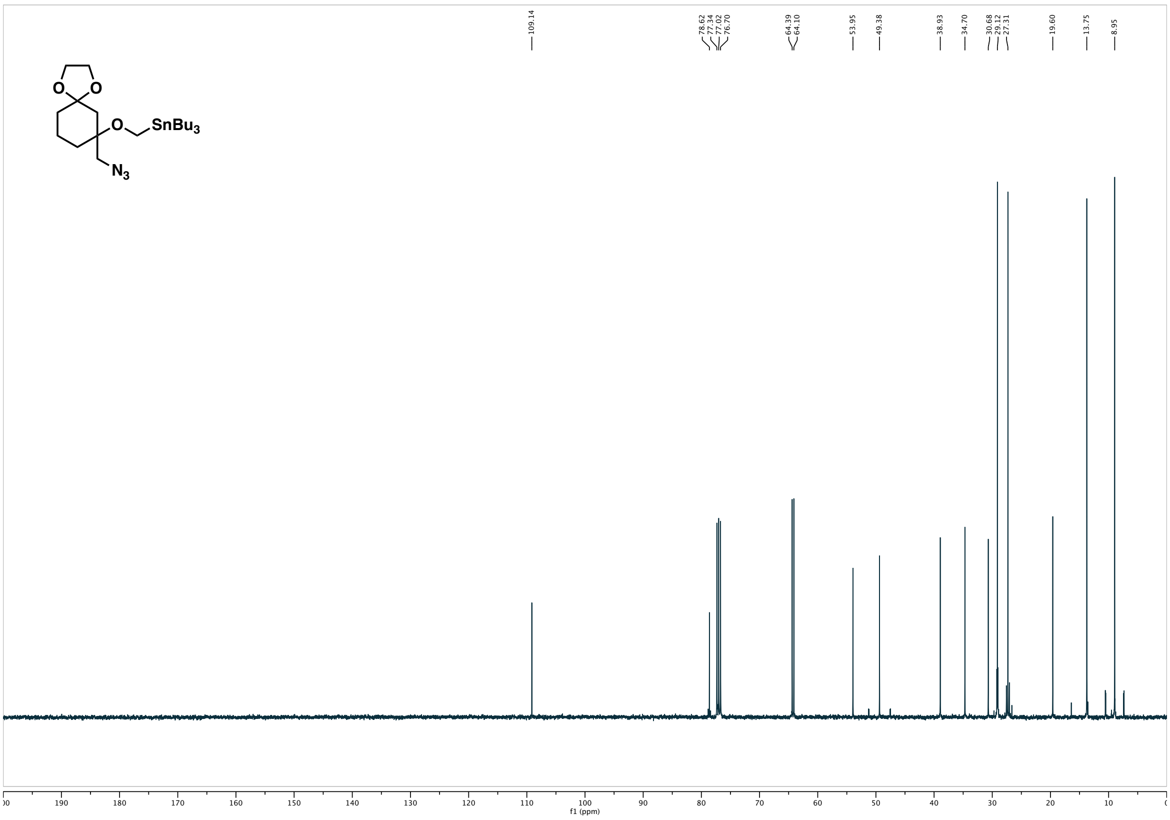

3-1

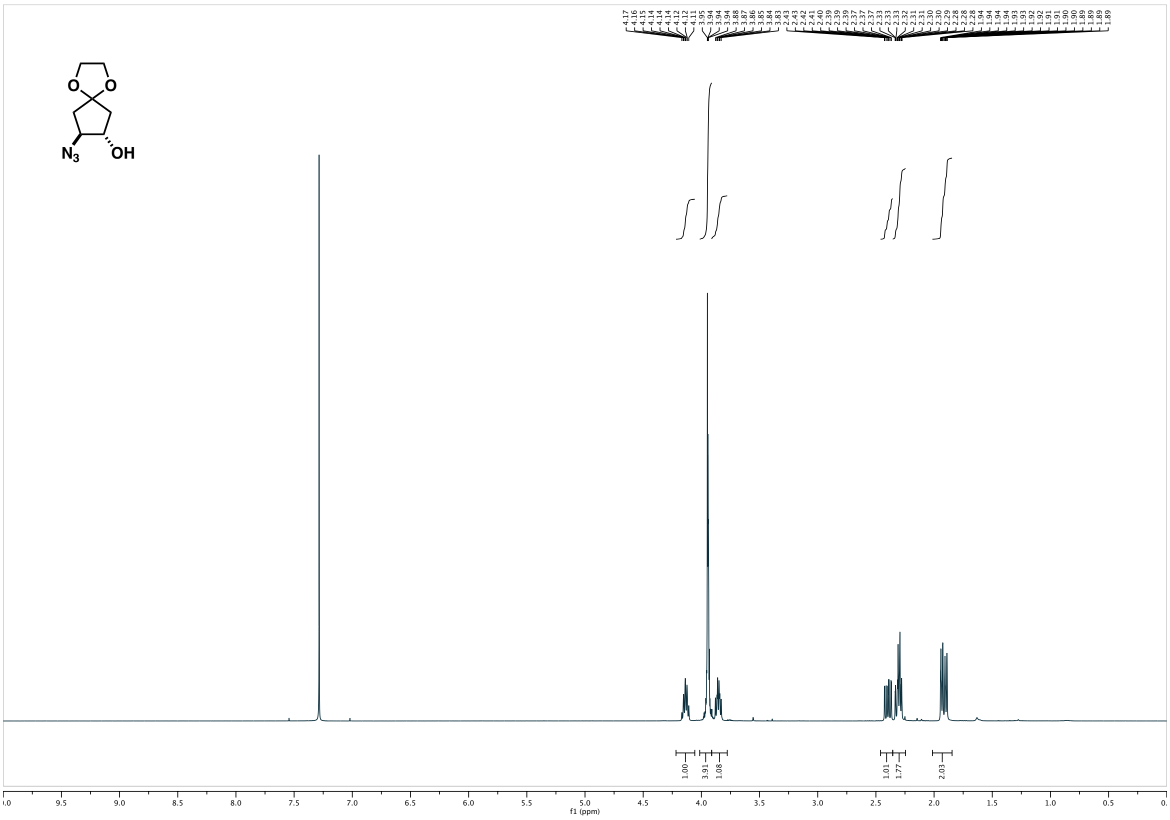
O
O
N3
OH
1.00
3.91
1.08
1.01
1.77
2.03
f1 (ppm)

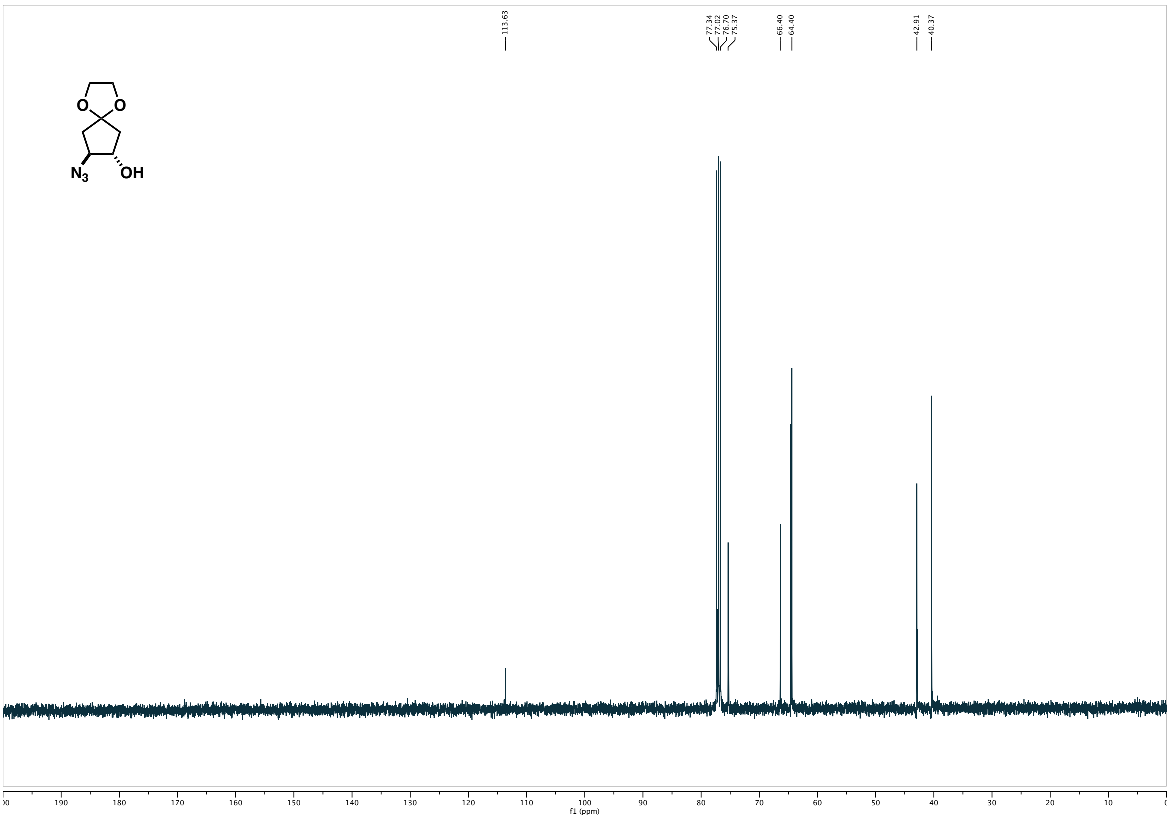
113.63
77.34
77.02
76.70
75.37
66.40
64.40
42.91
40.37
O
O
N3
OH
f1 (ppm)

3-2

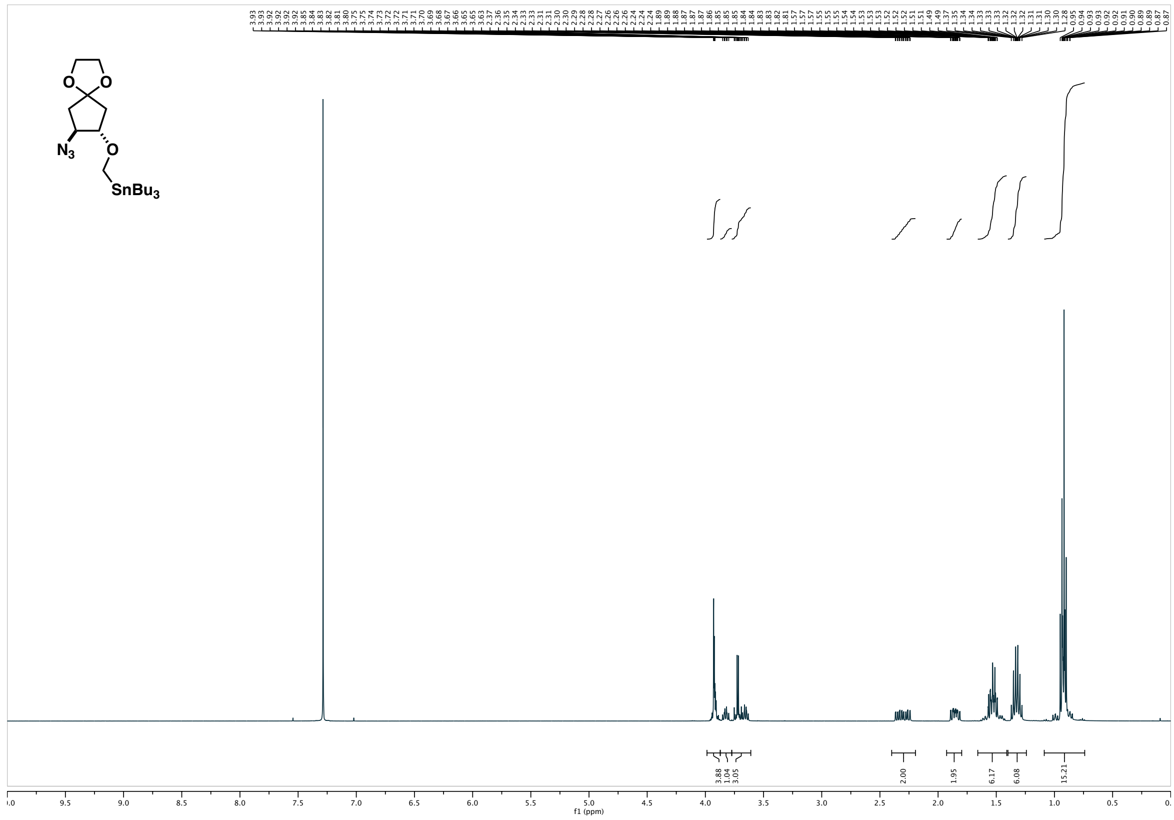
O
O
N3
O
SnBu3
3.88
1.04
3.05
2.00
1.95
6.17
6.08
15.21
f1 (ppm)

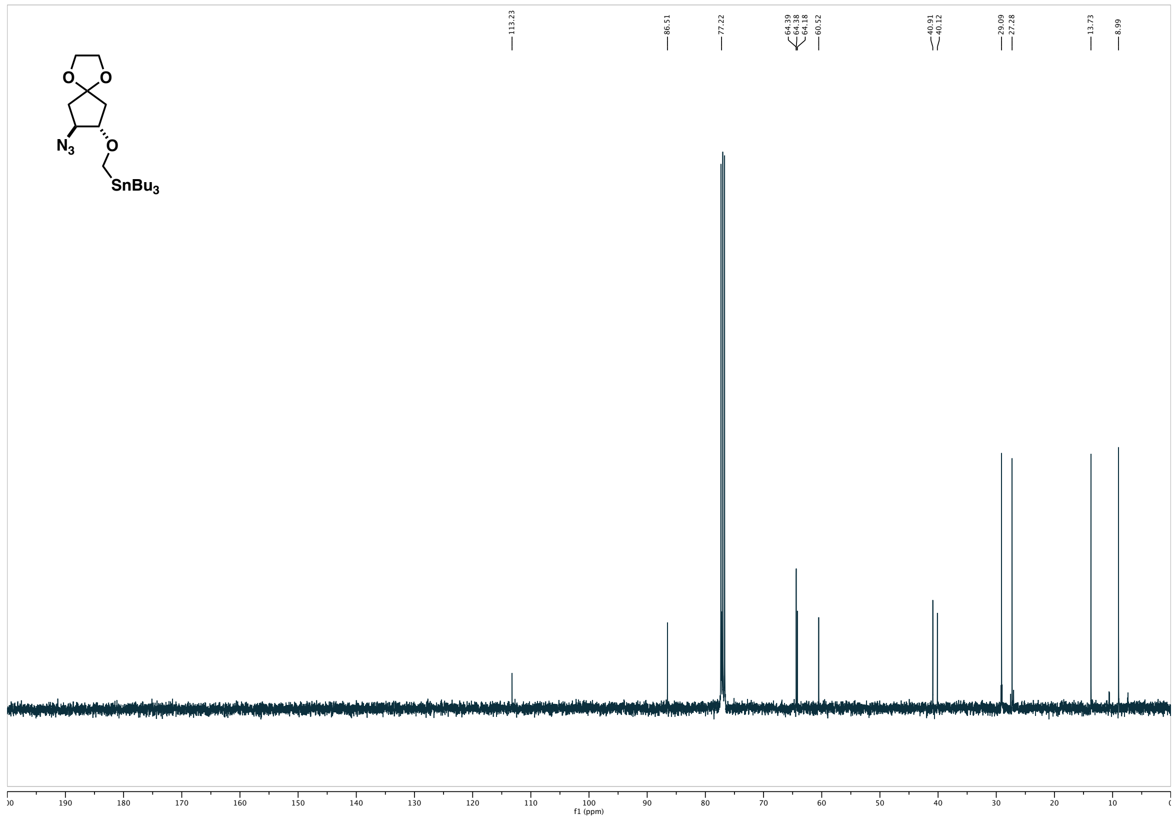
113.23
86.51
77.22
64.39
64.38
64.18
60.52
40.91
40.12
29.09
27.28
13.73
8.99
O
O
N3
O
SnBu3
f1 (ppm)

4-1

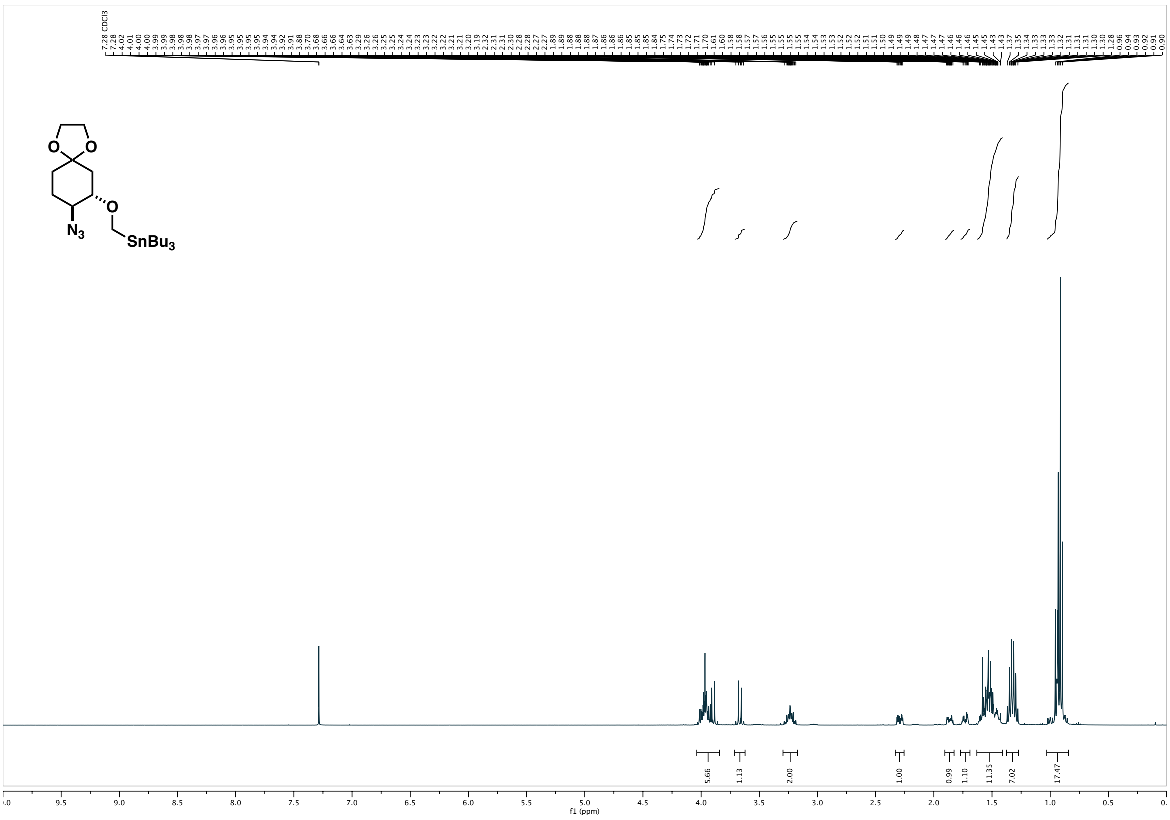
N3
SnBu3
5.66
1.13
2.00
1.00
0.99
1.10
11.35
7.02
17.47
f1 (ppm)

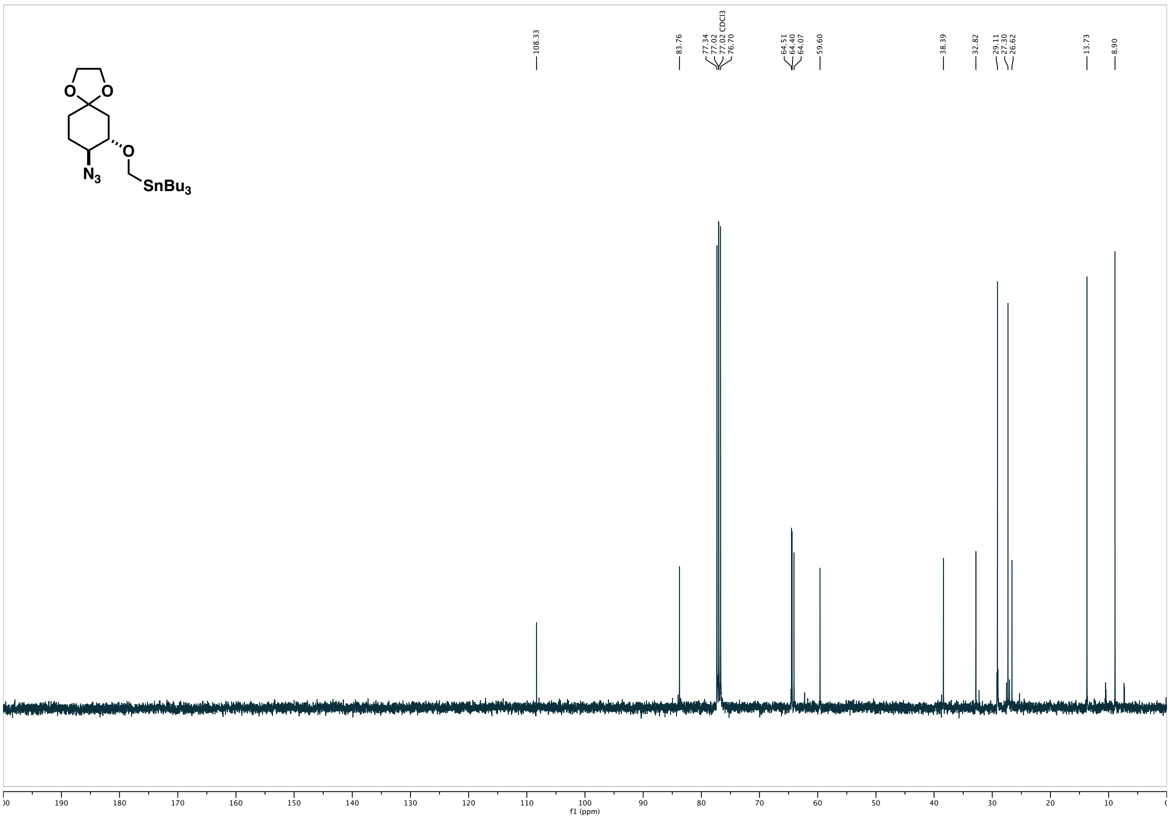
108.33
83.76
77.34
77.02
77.02 CDCl3
76.70
64.51
64.40
64.07
59.60
38.39
32.82
29.11
27.30
26.62
13.73
8.90
N3
SnBu3
f1 (ppm)

5-DiaA

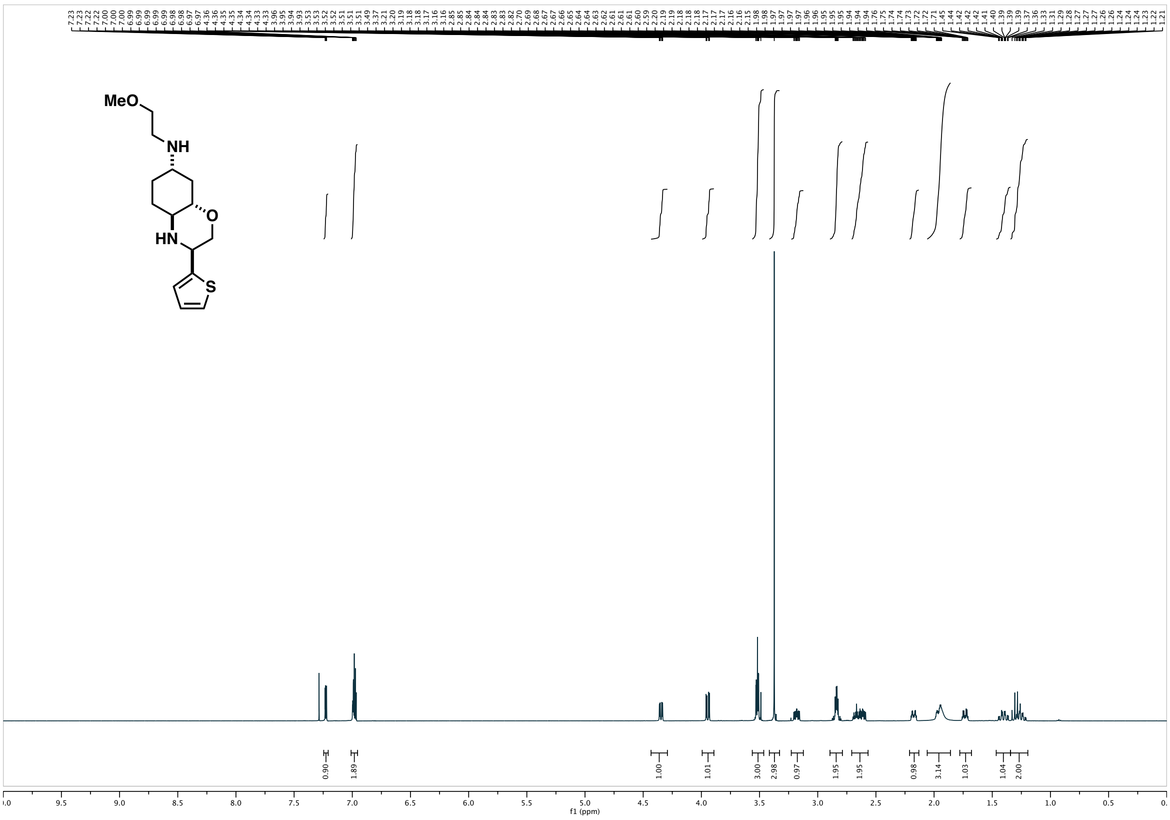
MeO
NH
O
HN
S
f1 (ppm)

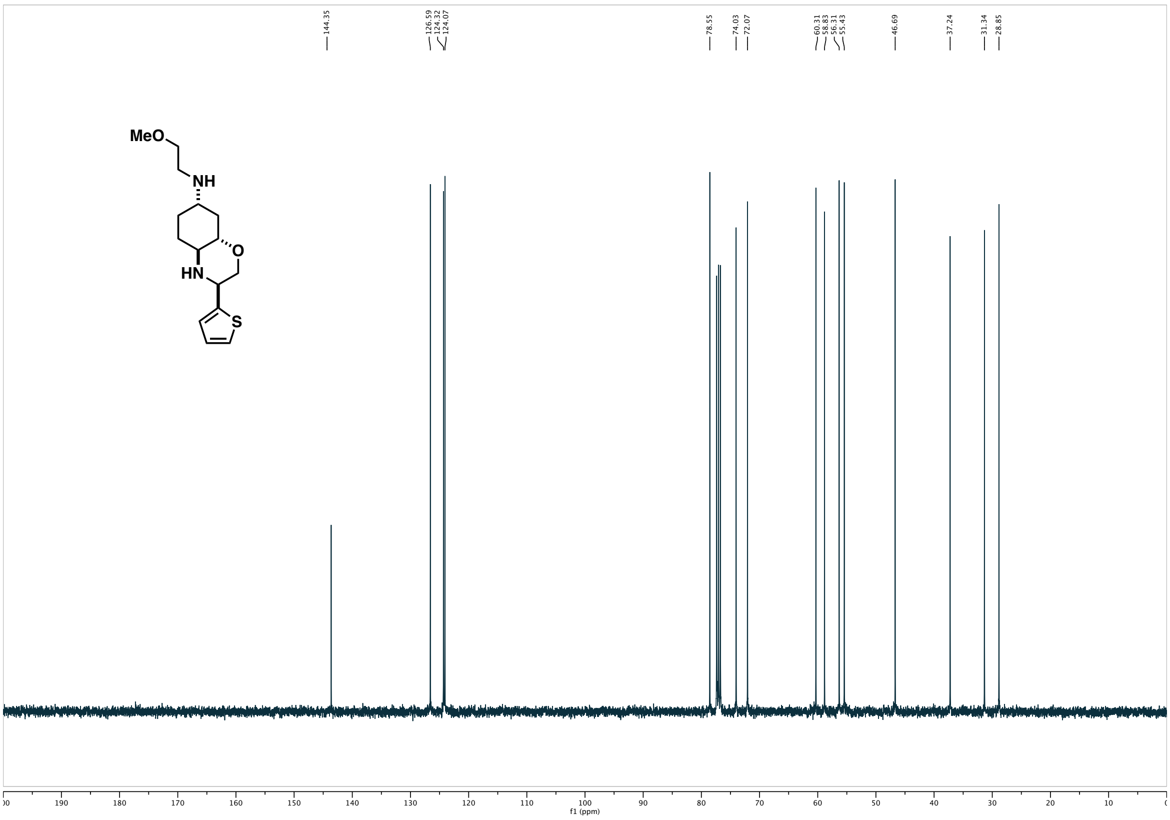
144.35
126.59
124.32
124.07
78.55
74.03
72.07
60.31
58.83
56.31
55.43
46.69
37.24
31.34
28.85
MeO
NH
O
HN
S
f1 (ppm)

5-DiaB

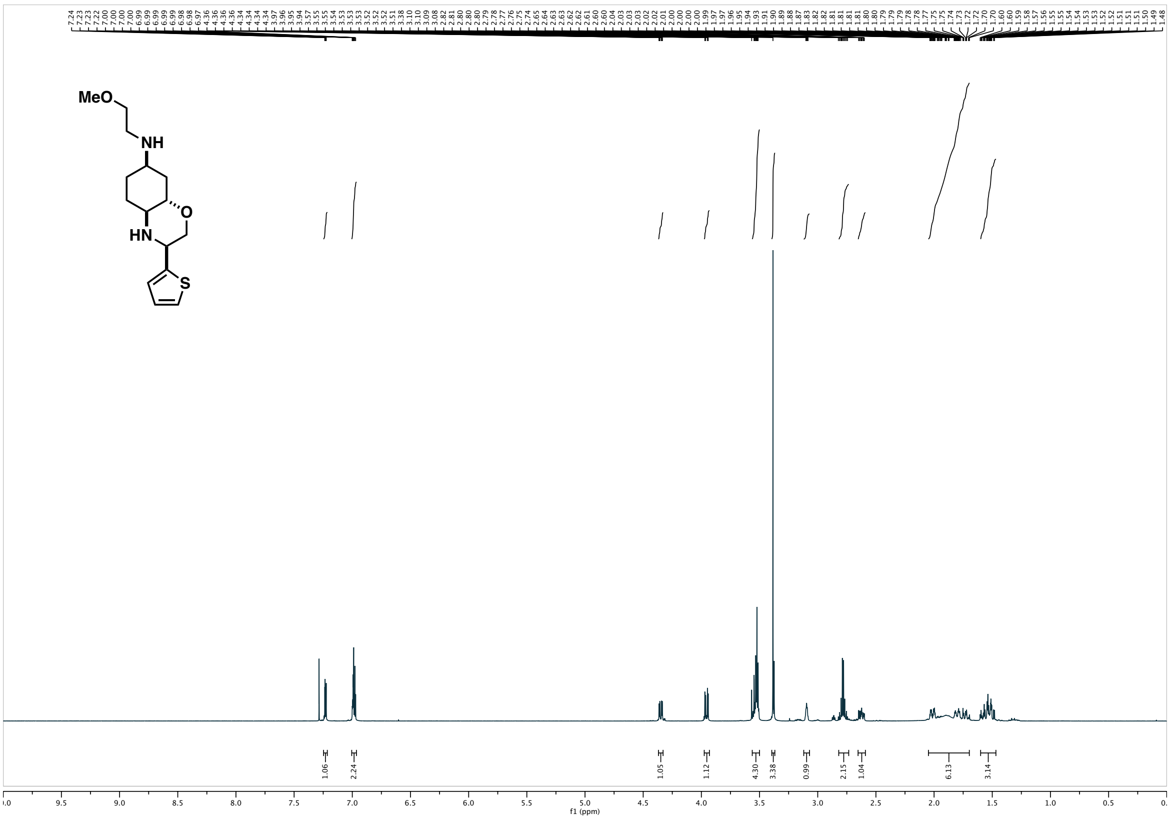

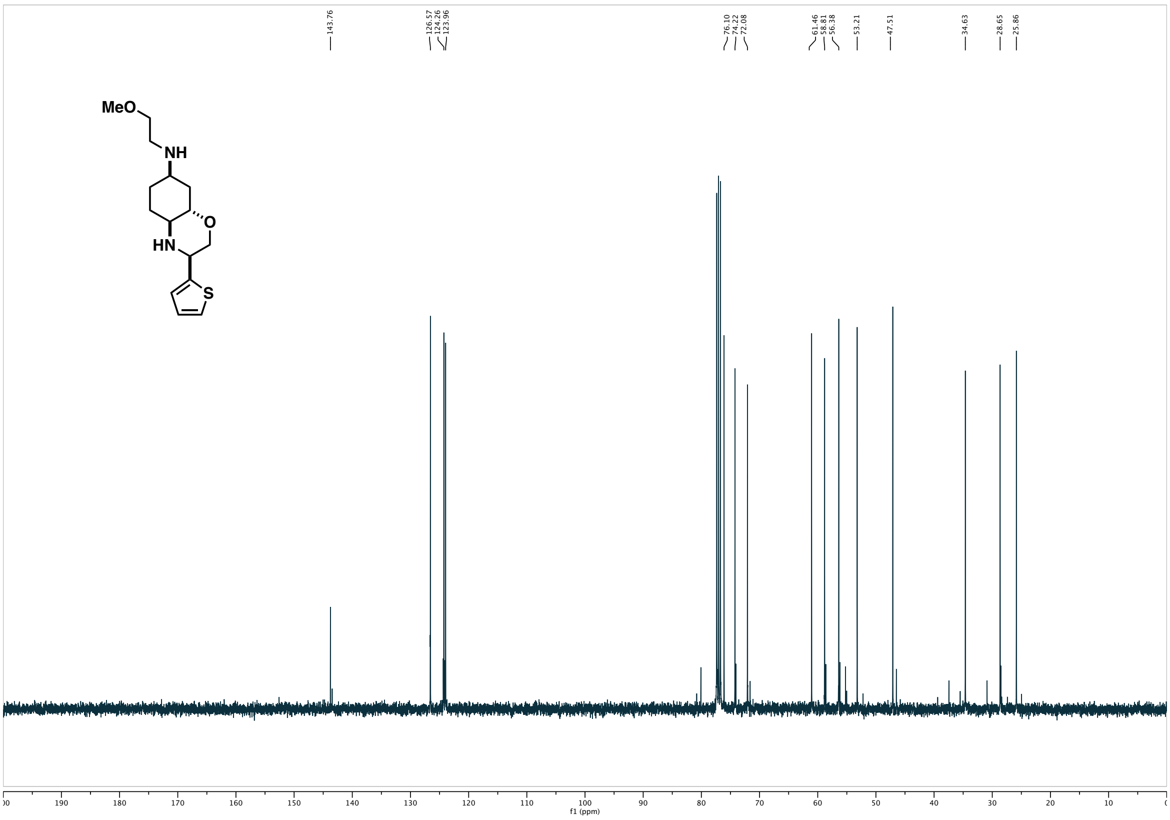

6-DiaA

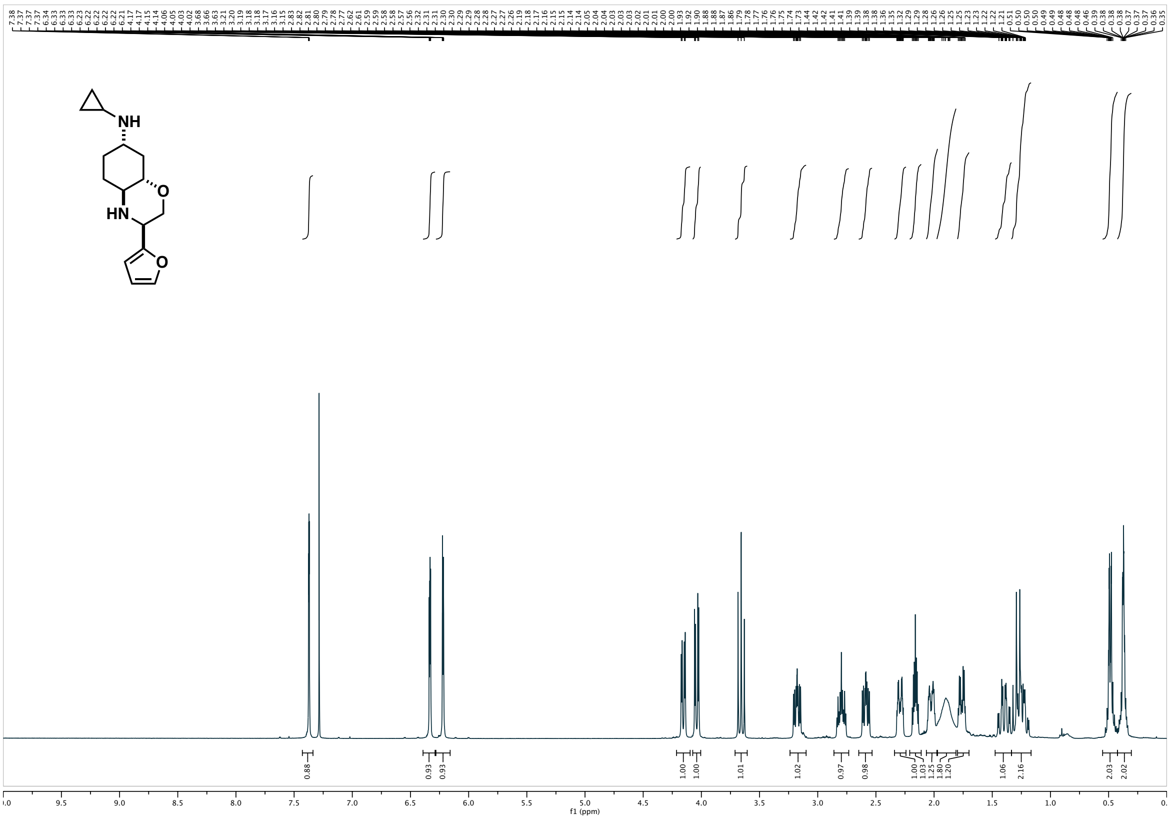

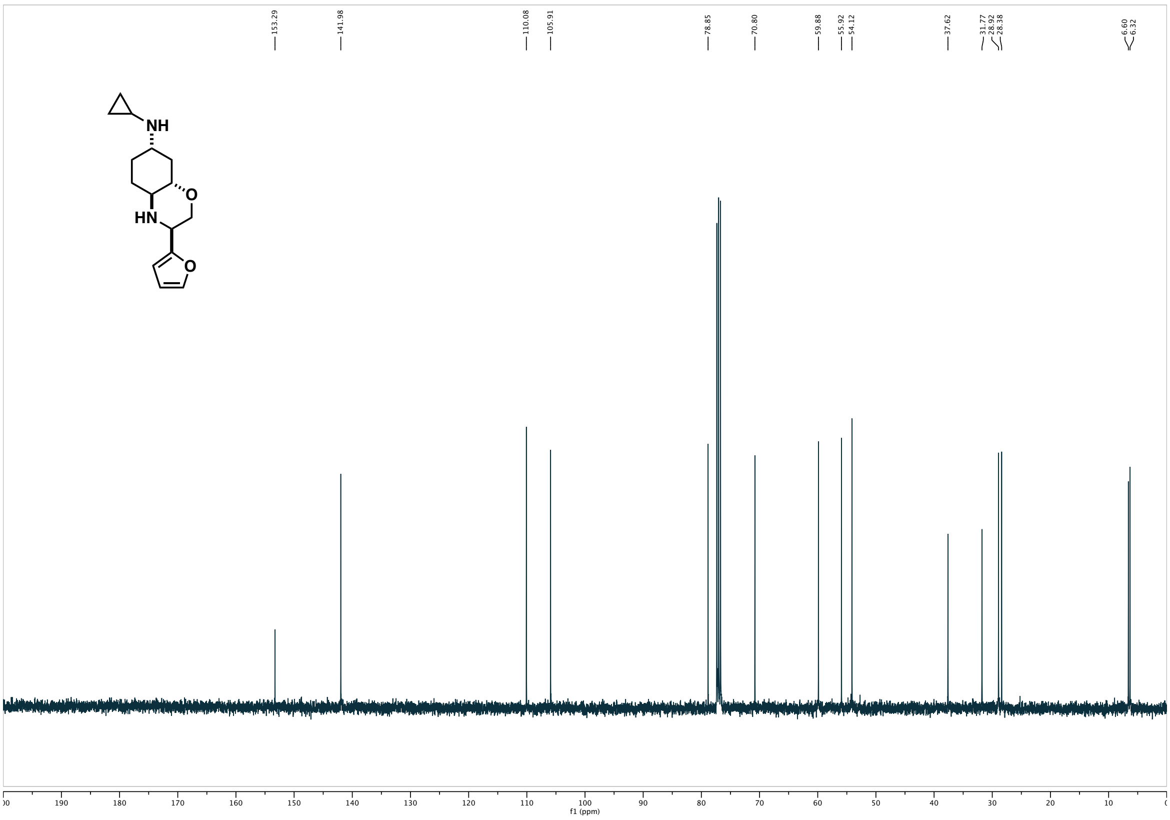
153.29
141.98
110.08
105.91
78.85
70.80
59.88
55.92
54.12
37.62
31.77
28.92
28.38
6.60
6.32
f1 (ppm)

6-DiaB

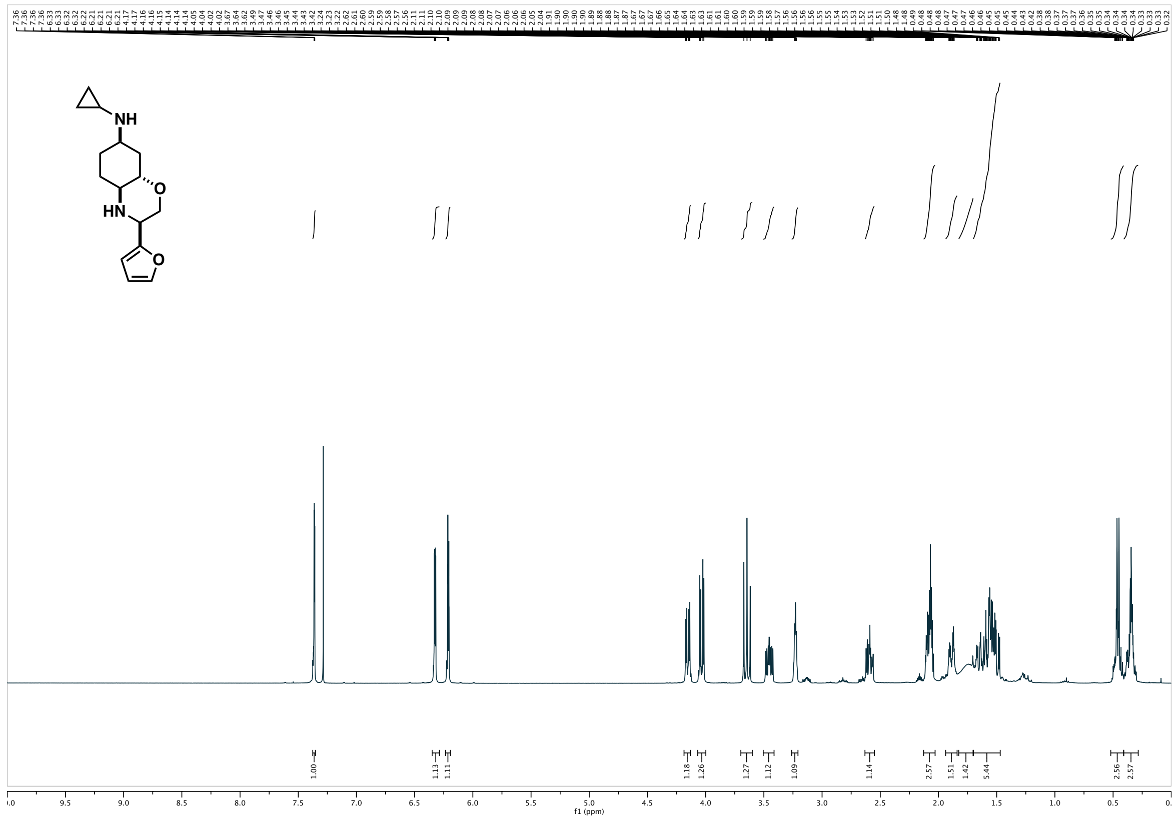

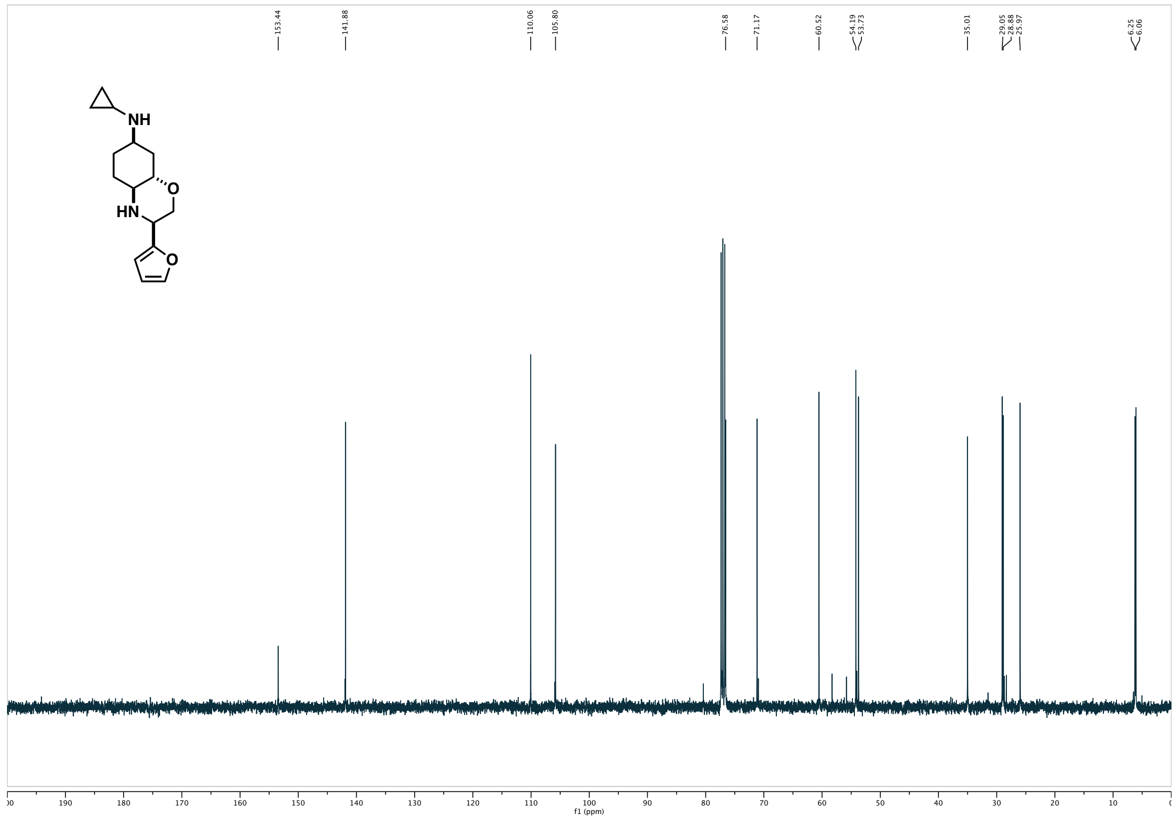

7-DiaA

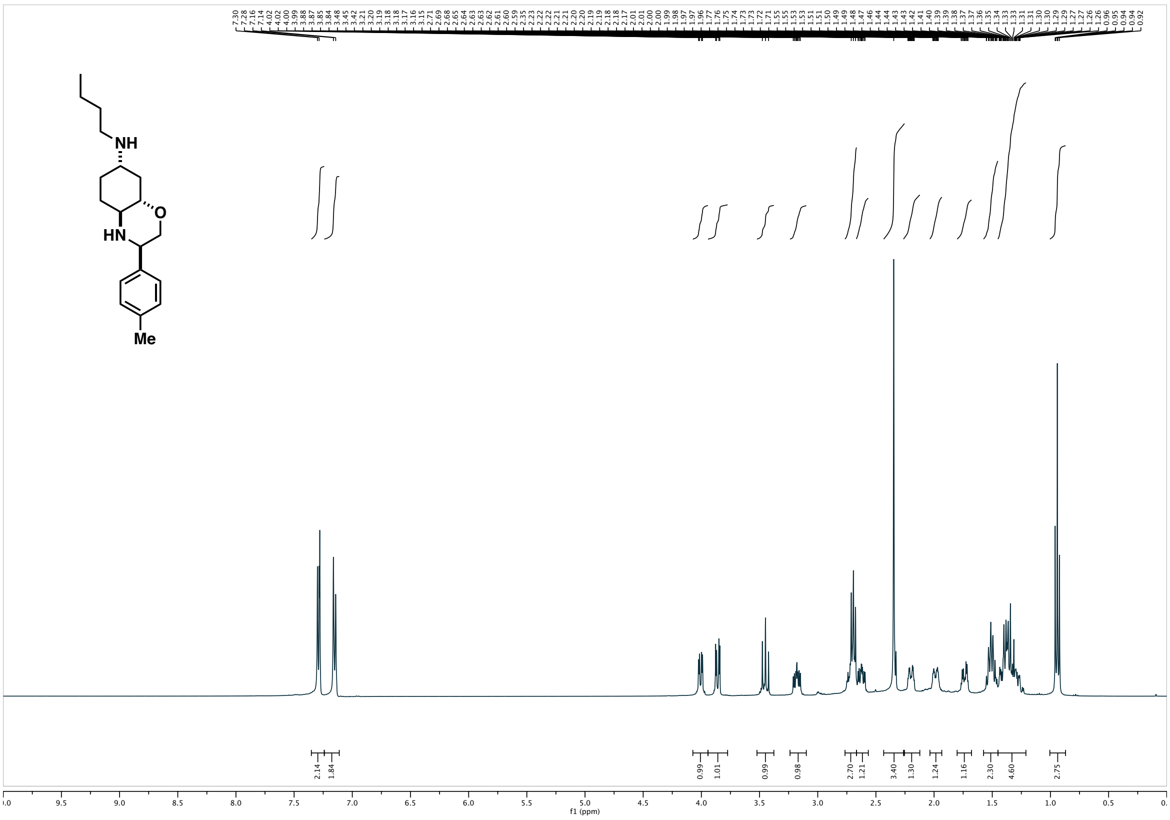

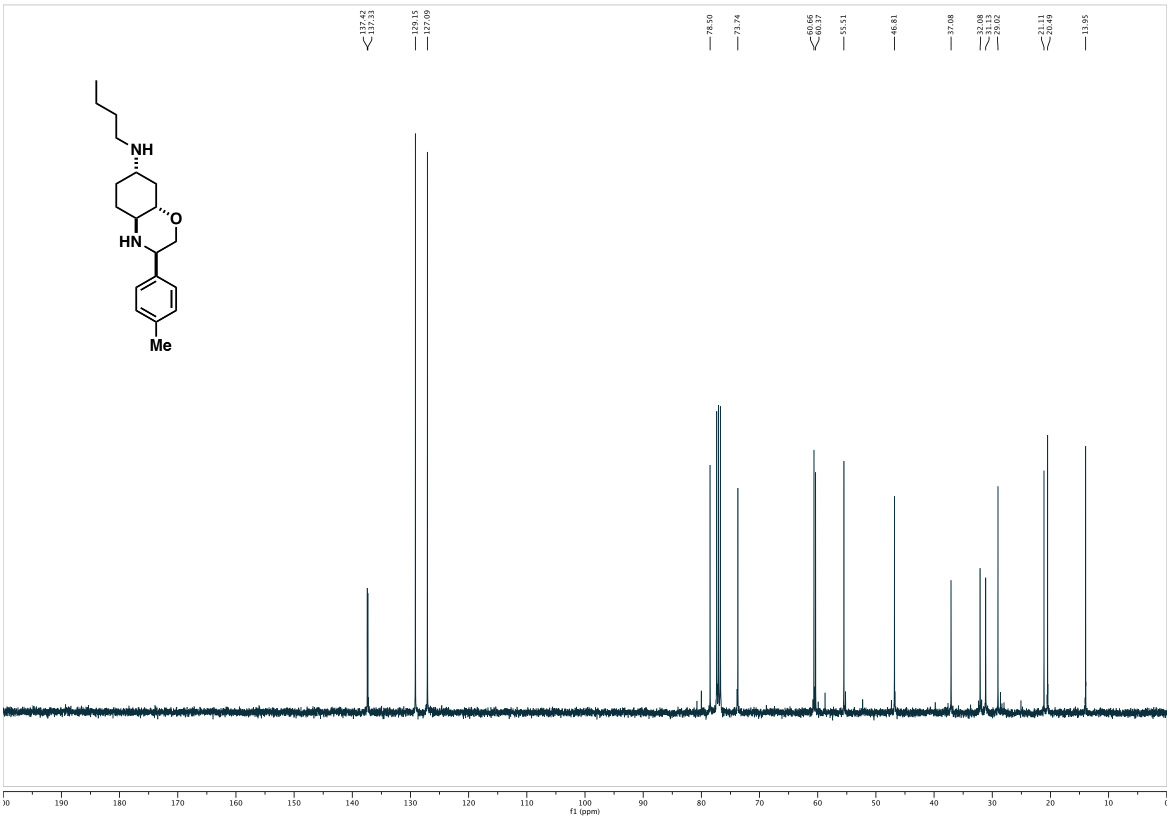

7-DiaB

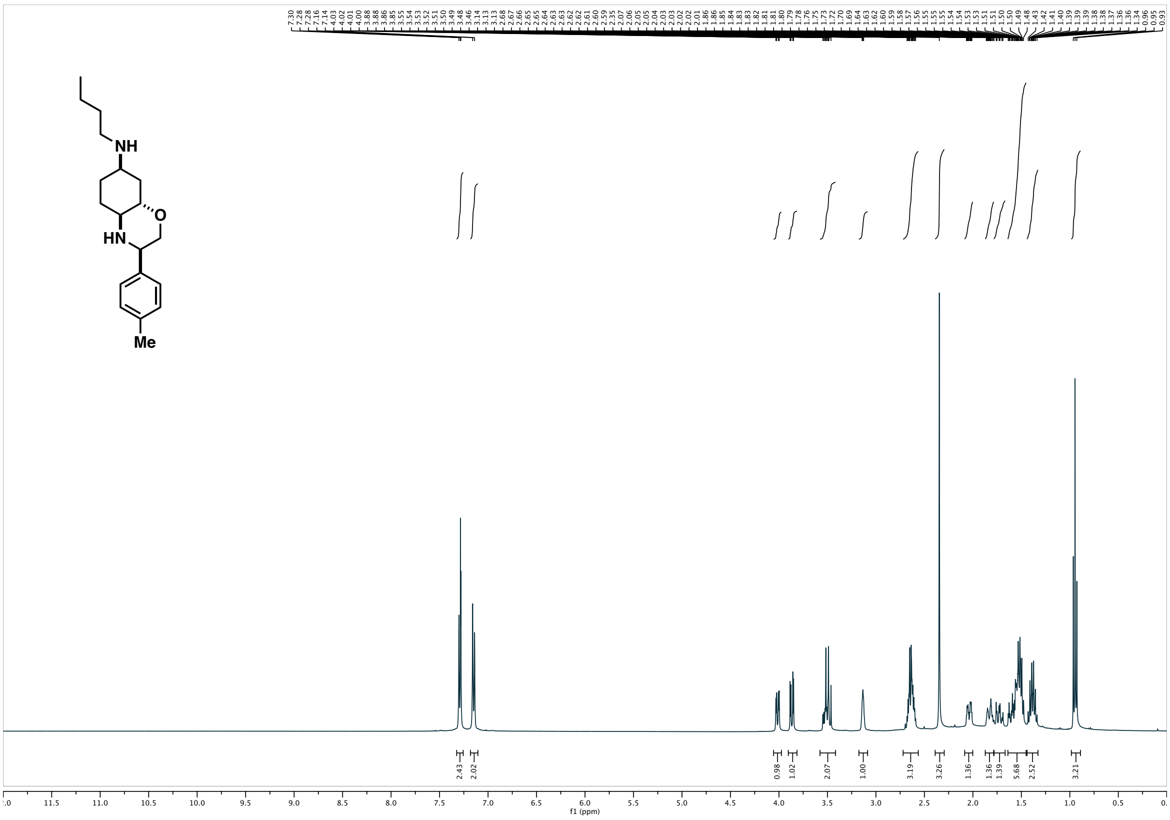

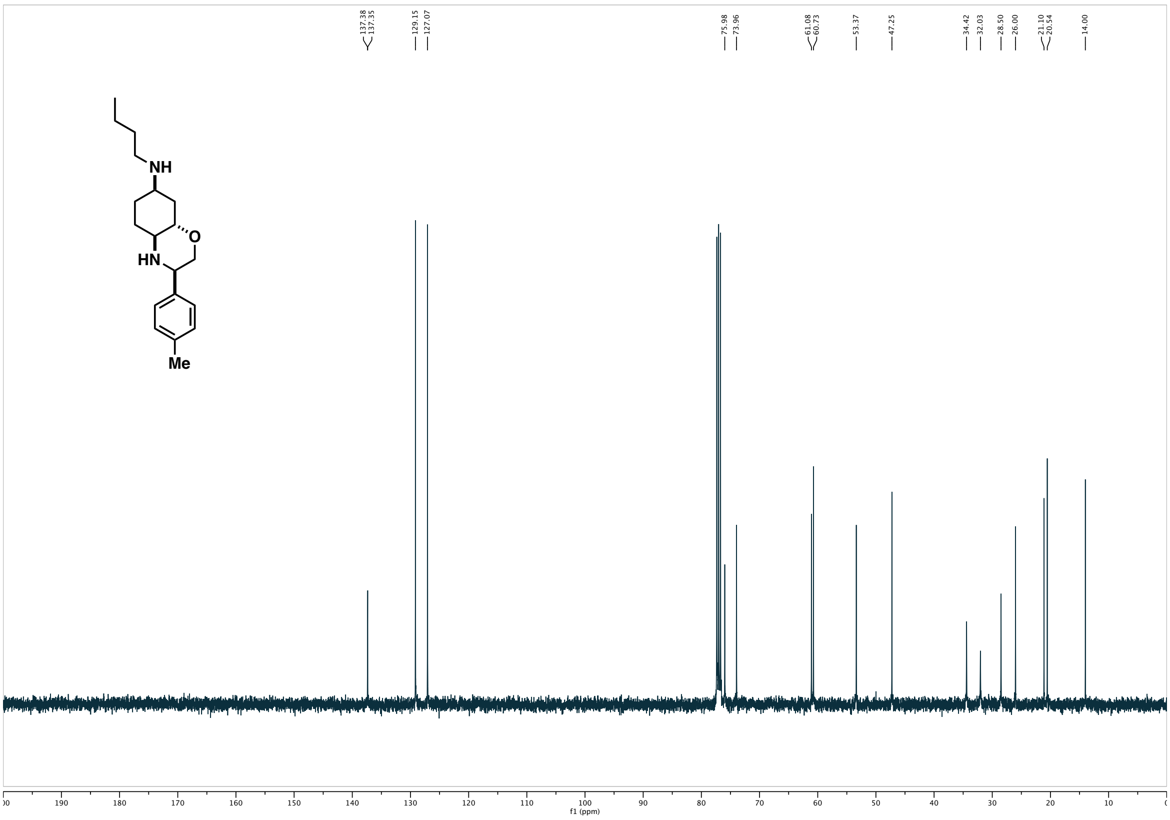

8-DiaA

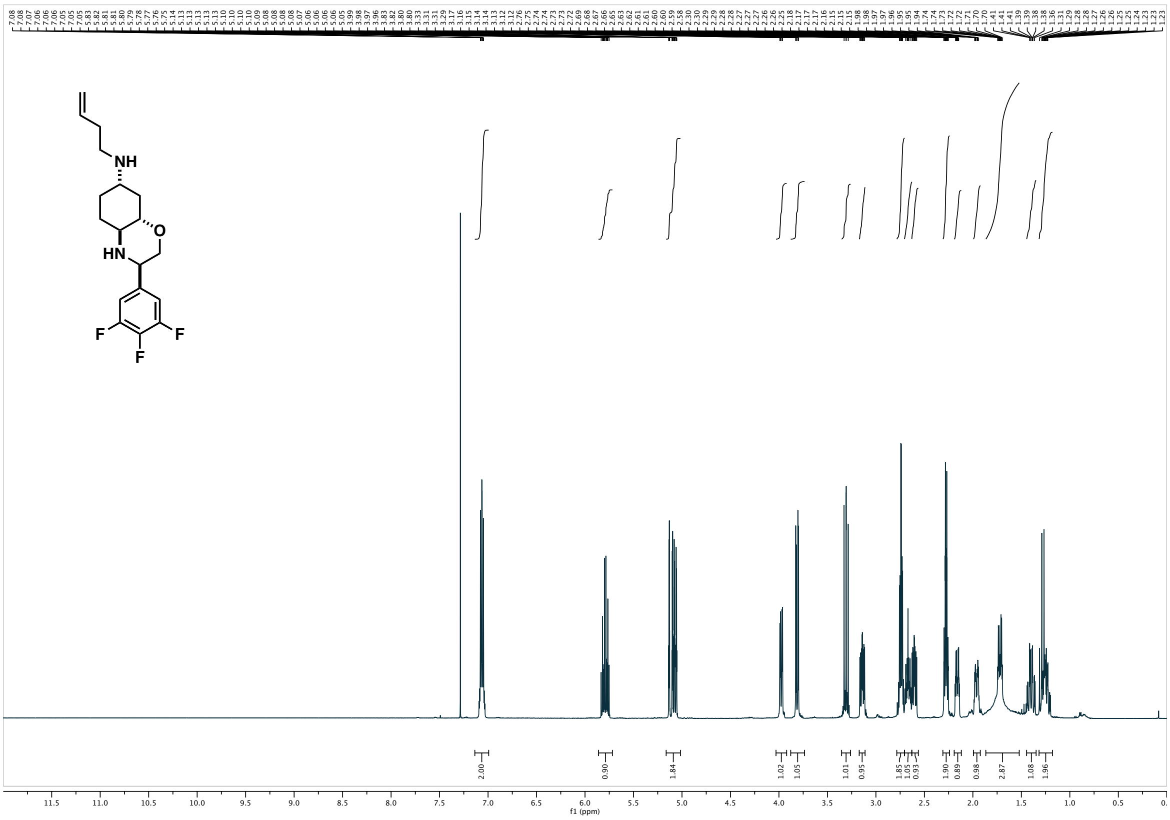
NH
O
HN
F
F
F
2.00
0.90
1.84
1.02
1.05
1.01
0.95
1.85
1.05
0.93
1.90
0.89
0.98
2.87
1.08
1.96
f1 (ppm)

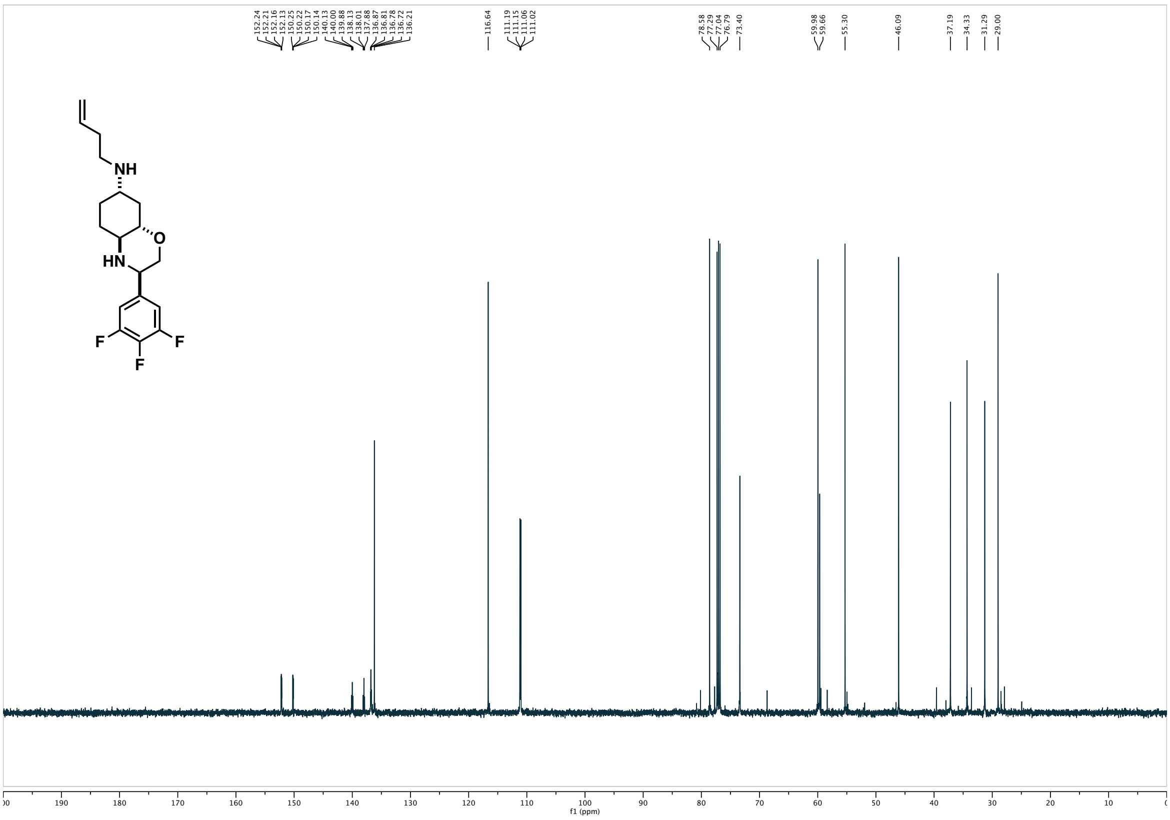
152.24
152.21
152.16
152.13
150.25
150.22
150.17
150.14
140.13
140.00
139.88
138.13
138.01
137.88
136.87
136.81
136.78
136.72
136.21
116.64
111.19
111.15
111.06
111.02
78.58
77.29
77.04
76.79
73.40
59.98
59.66
55.30
46.09
37.19
34.33
31.29
29.00
NH
O
HN
F
F
F
f1 (ppm)

8-DiaB

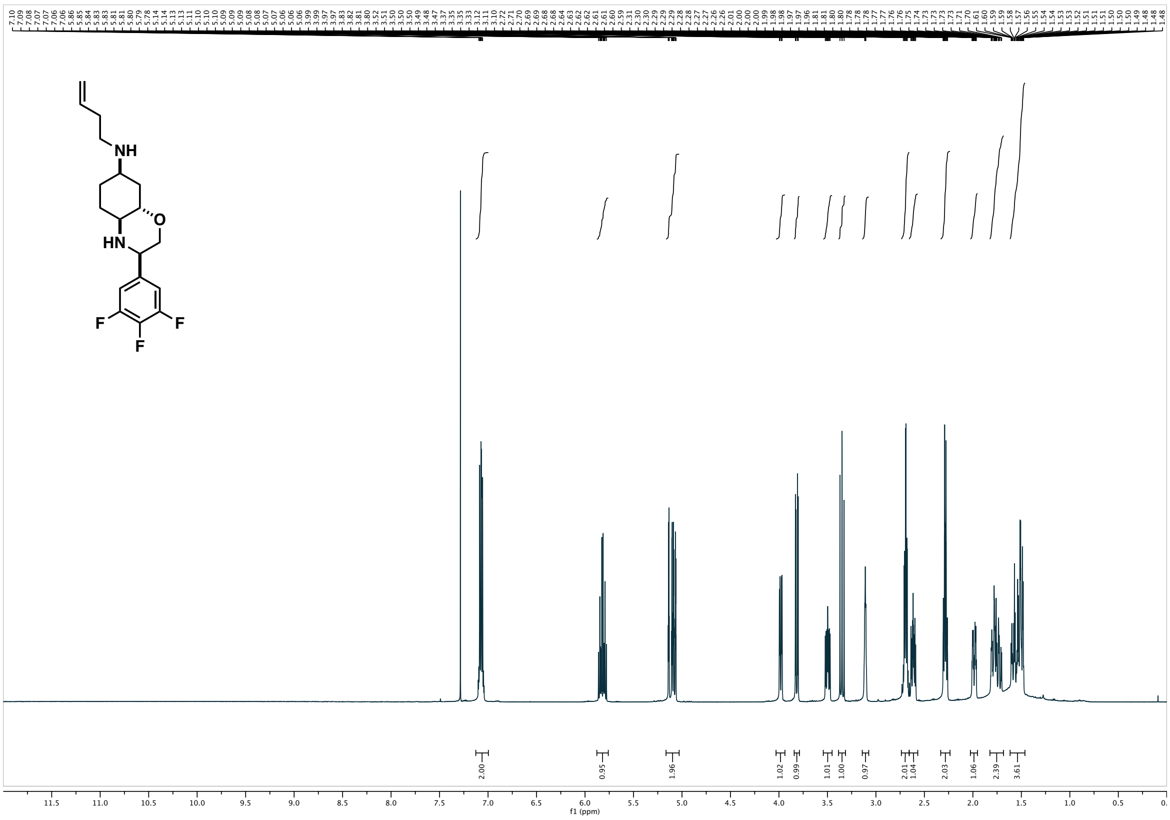

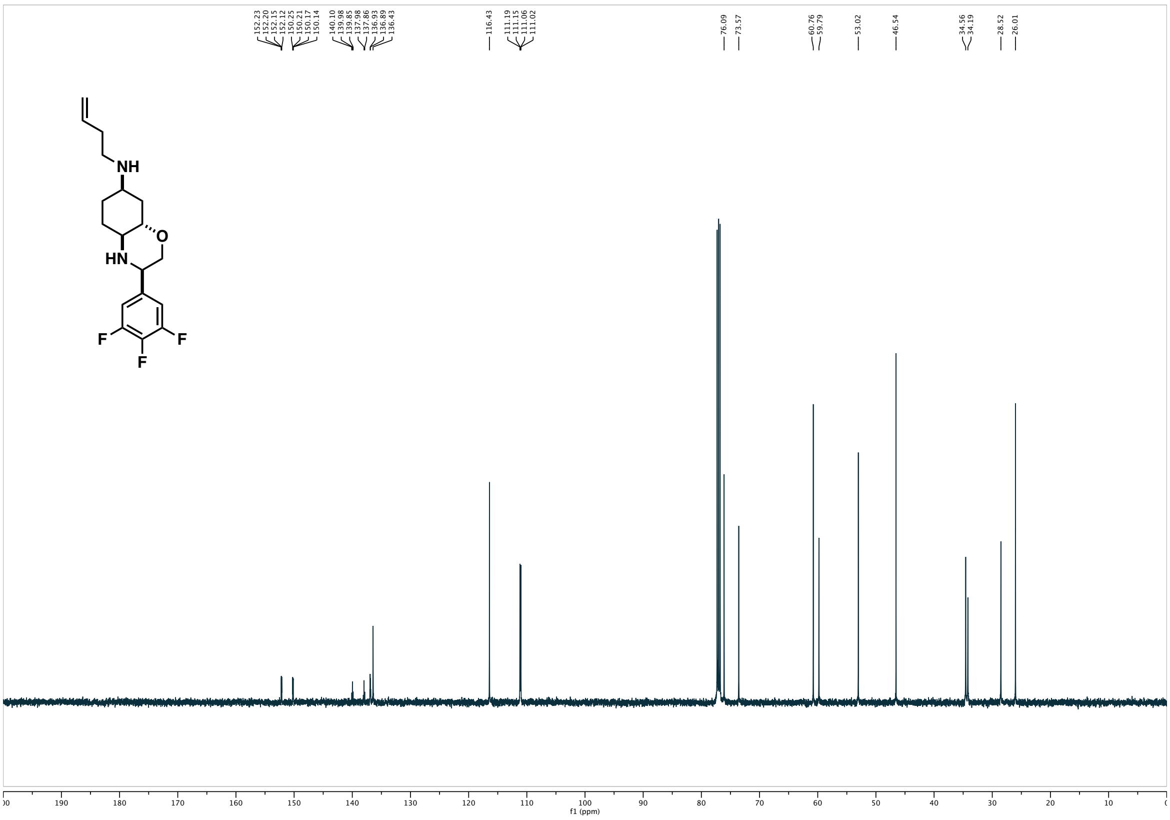

9

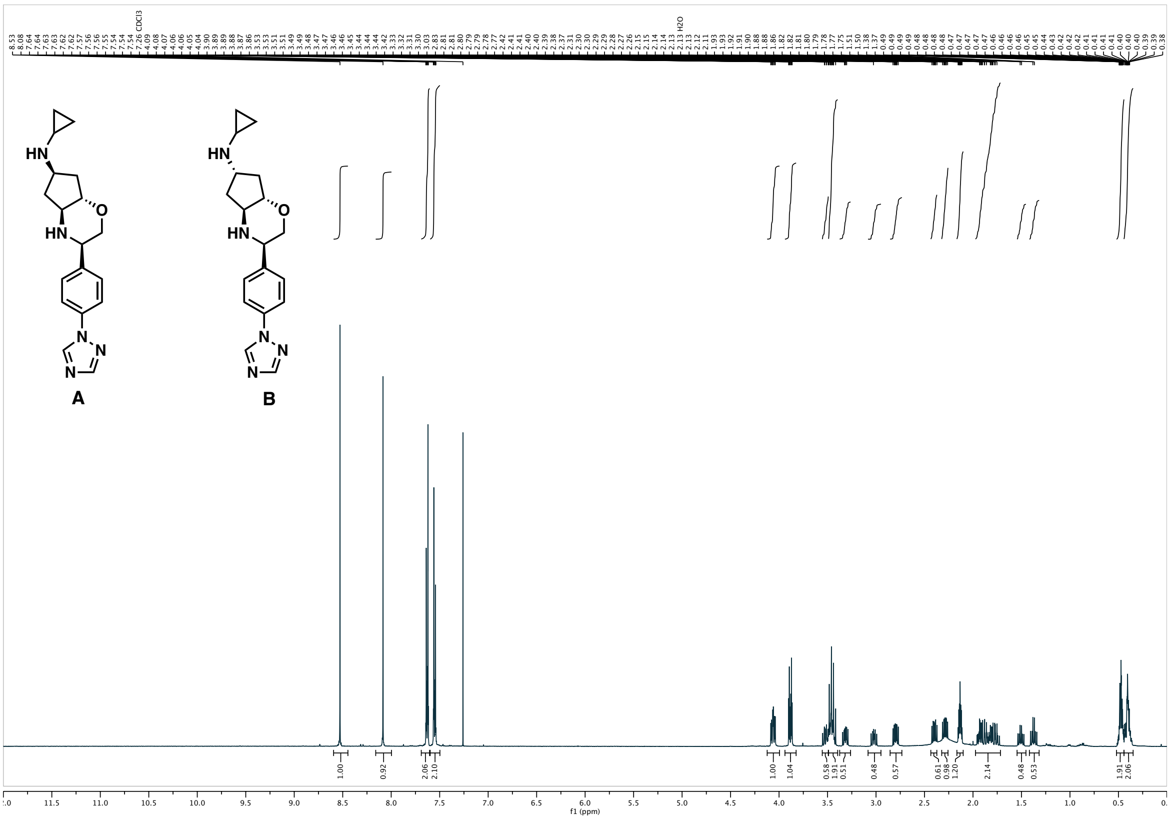

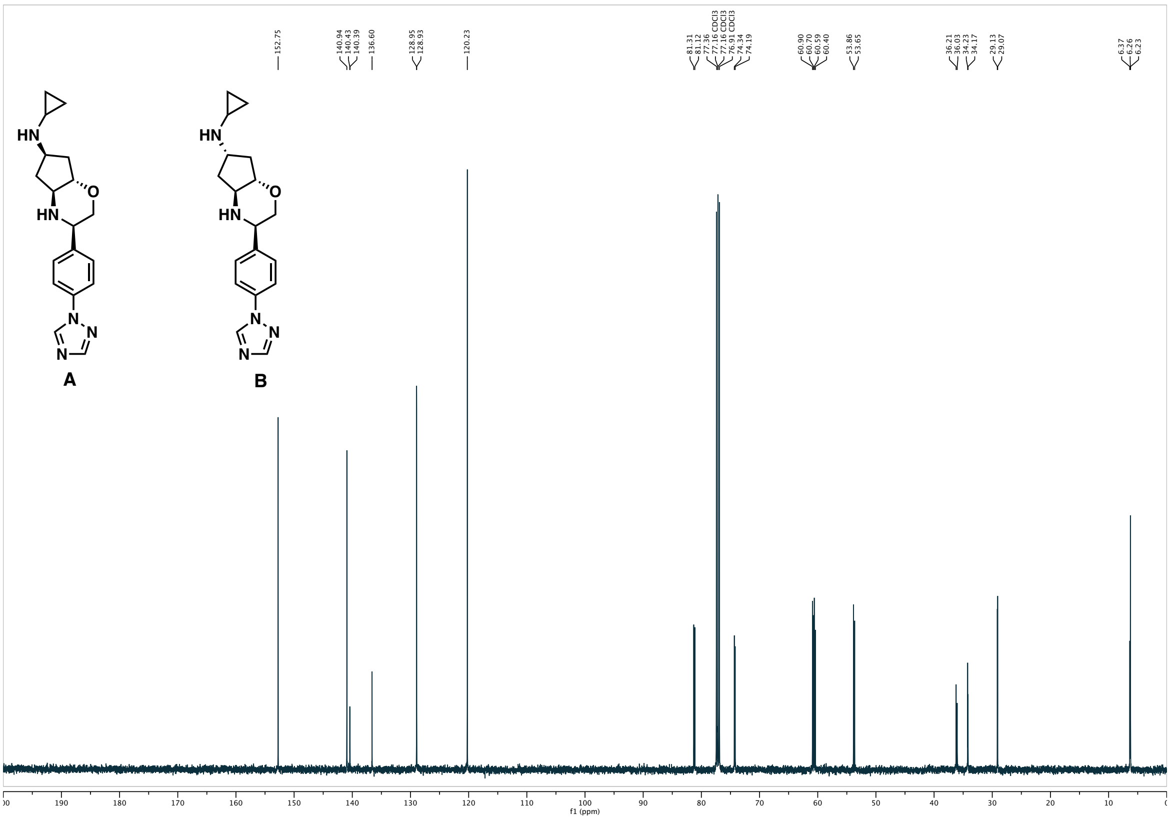

10

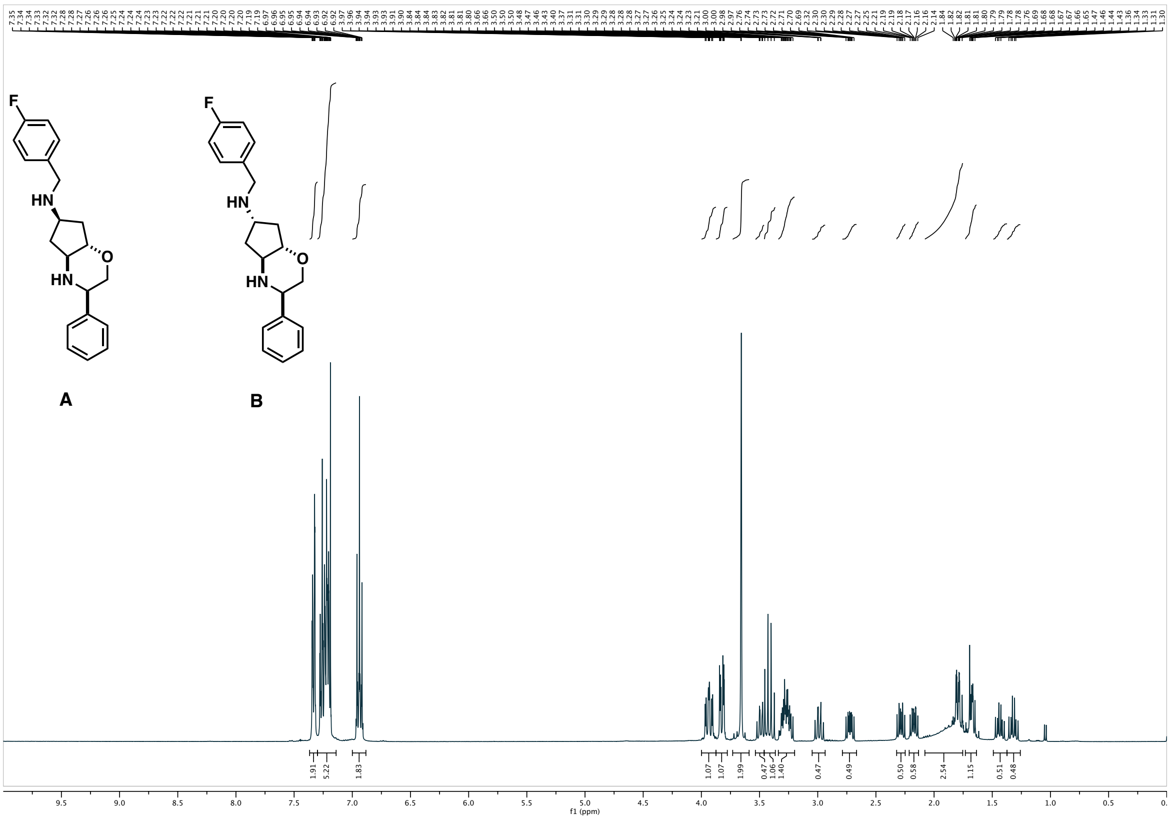

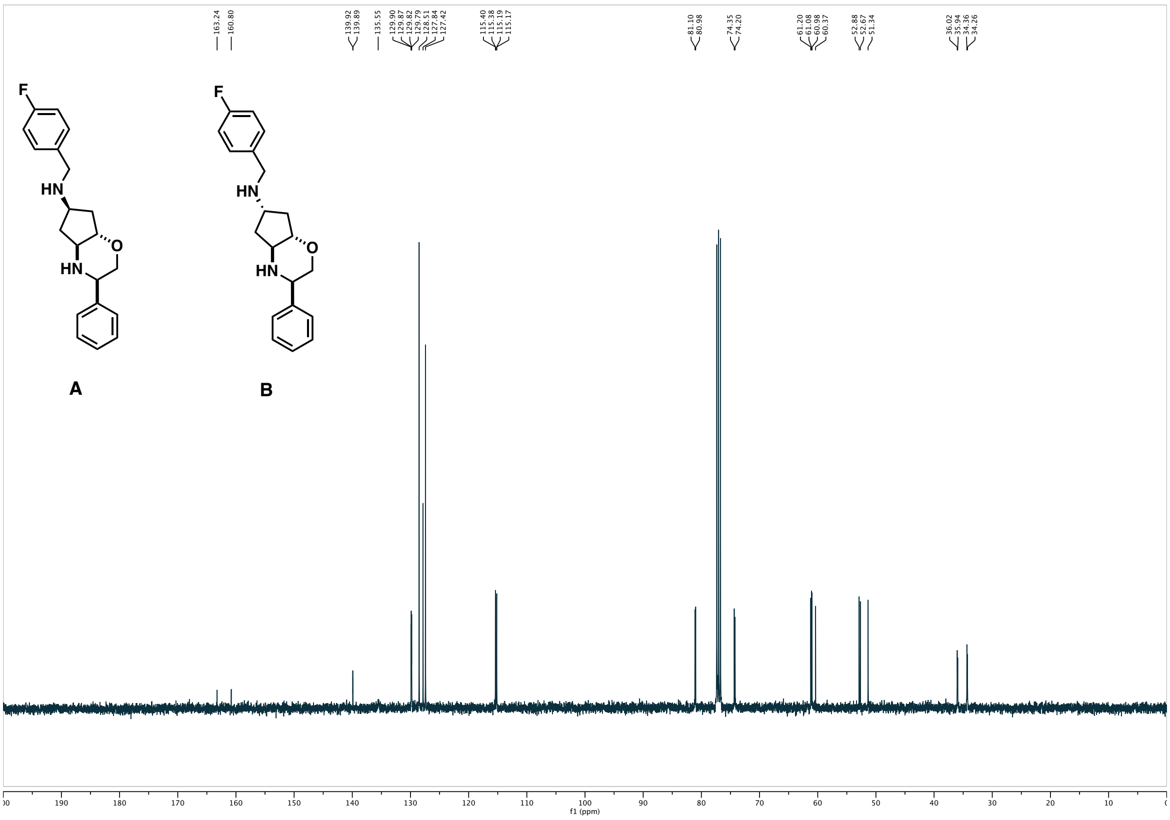

11

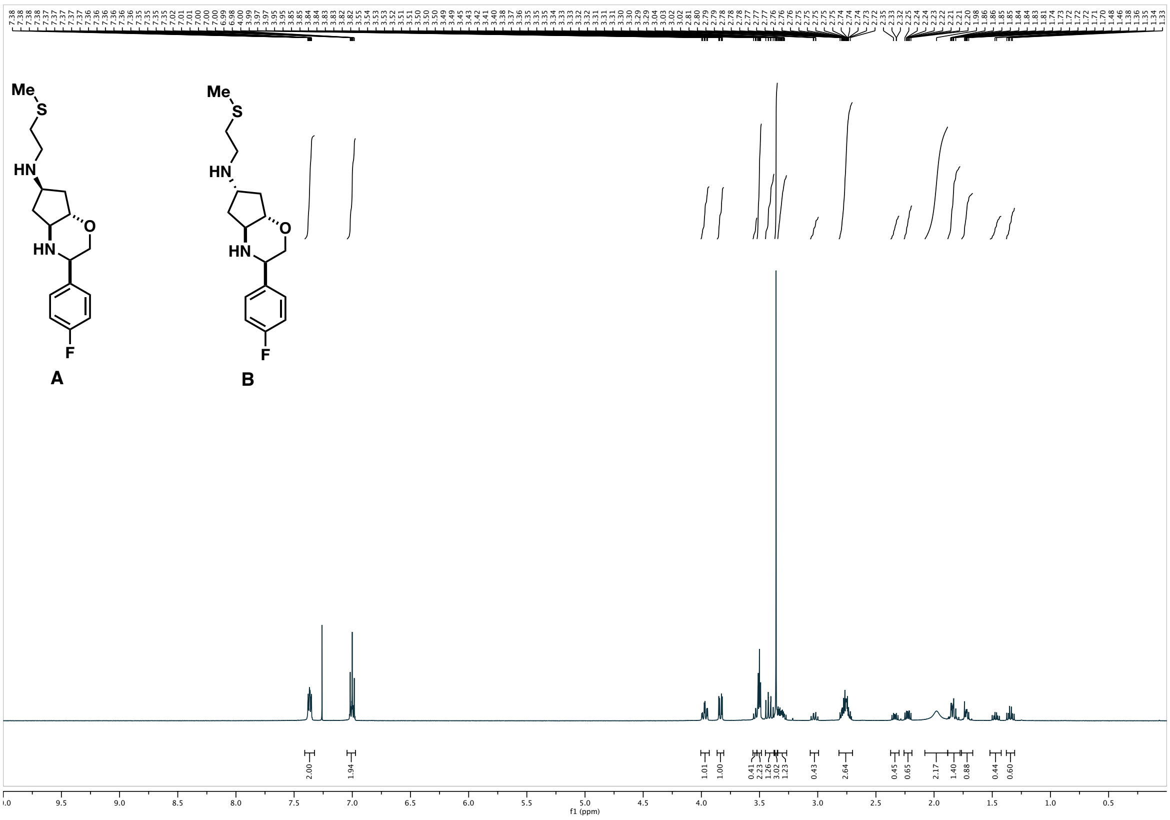
Me
S
HN
O
HN
F
A
B
2.00
1.94
1.01
1.00
0.41
2.23
1.26
3.02
1.23
0.43
2.64
0.45
0.65
2.17
1.40
0.88
0.44
0.60
f1 (ppm)

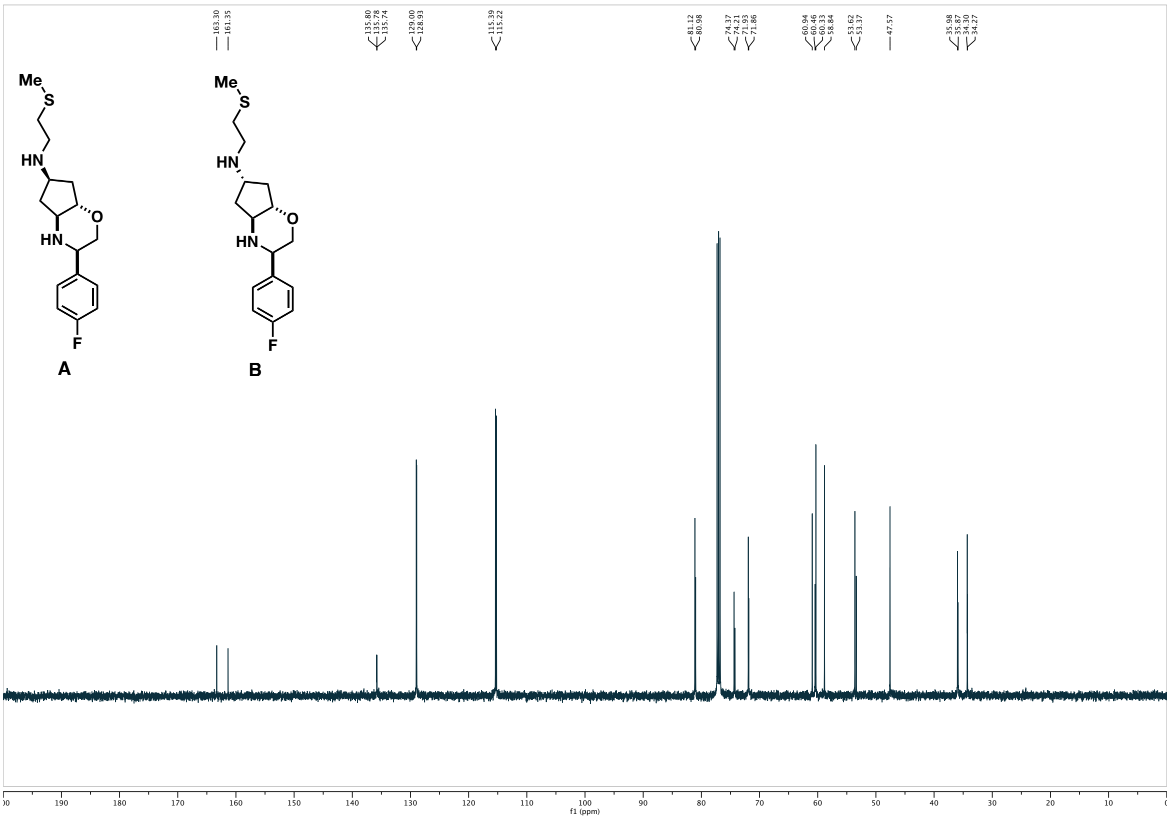
163.30
161.35
135.80
135.78
135.74
129.00
128.93
115.39
115.22
81.12
80.98
74.37
74.21
71.93
71.86
60.94
60.46
60.33
58.84
53.62
53.37
47.57
35.98
35.87
34.30
34.27
Me
S
HN
O
HN
F
A
B
f1 (ppm)

12

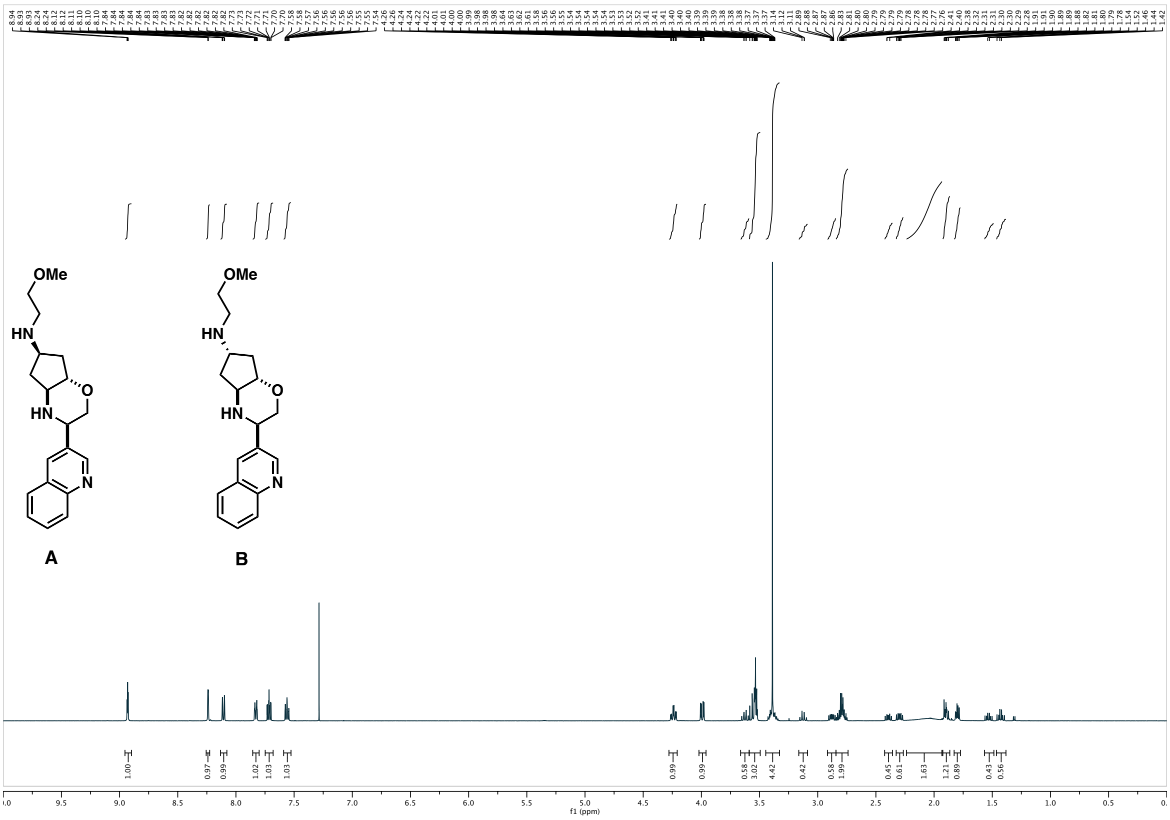

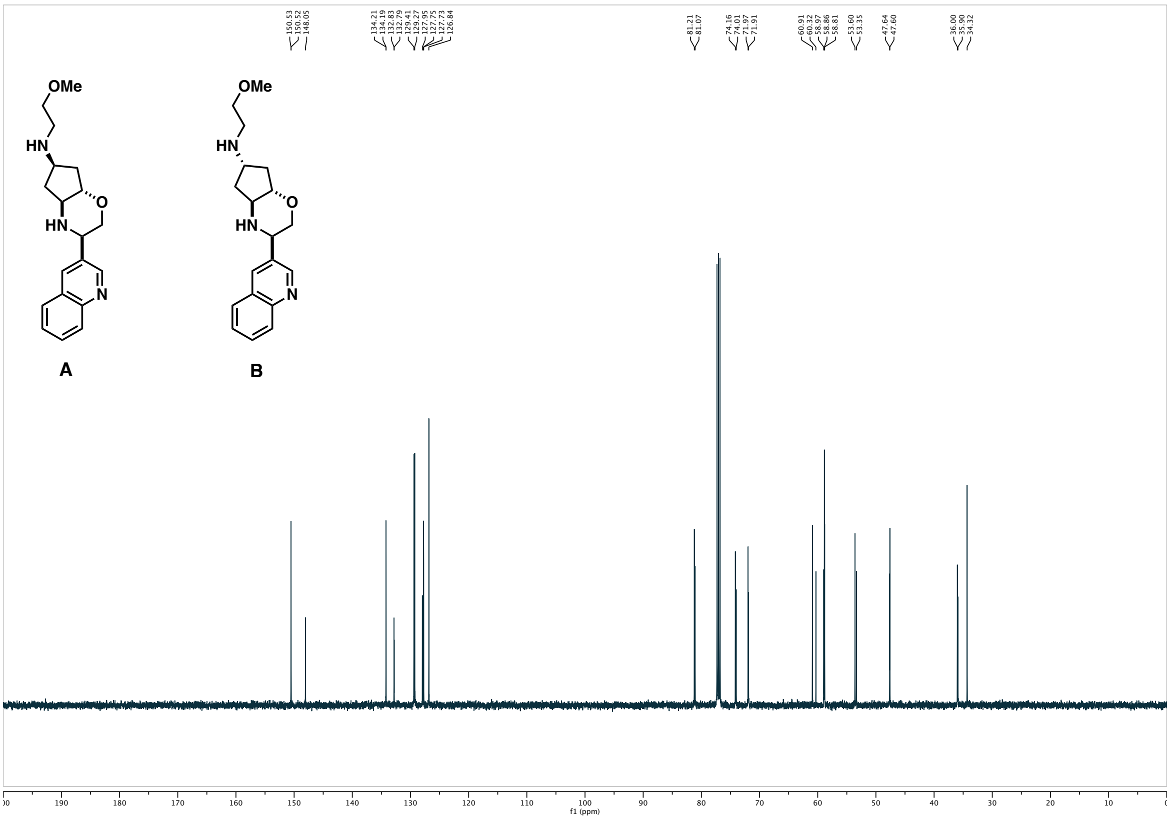

13-DiaA

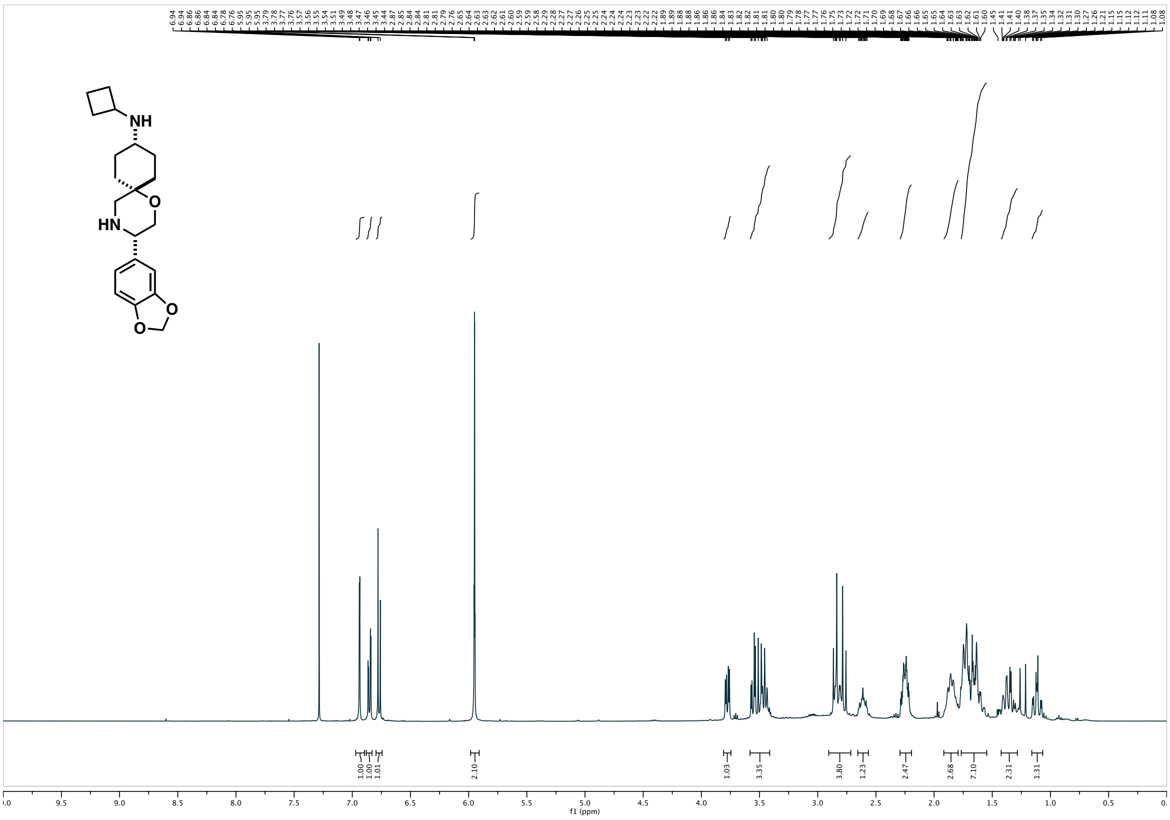
NH
O
HN
O
O
1.00
1.00
1.01
2.10
1.03
3.35
3.80
1.23
2.47
2.68
7.10
2.31
1.31
f1 (ppm)

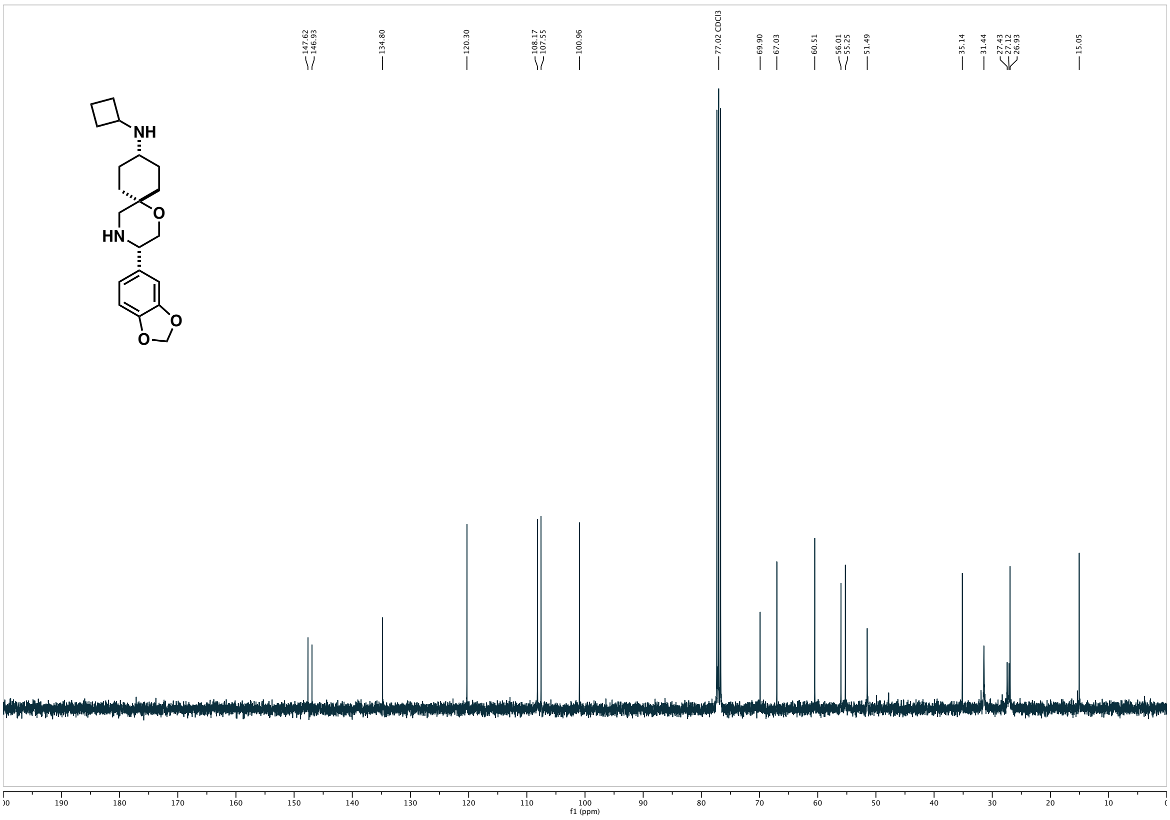
147.62
146.93
134.80
120.30
108.17
107.55
100.96
77.02 CDCl3
69.90
67.03
60.51
56.01
55.25
51.49
35.14
31.44
27.43
27.12
26.93
15.05
NH
O
HN
O
O
f1 (ppm)

13-DiaB

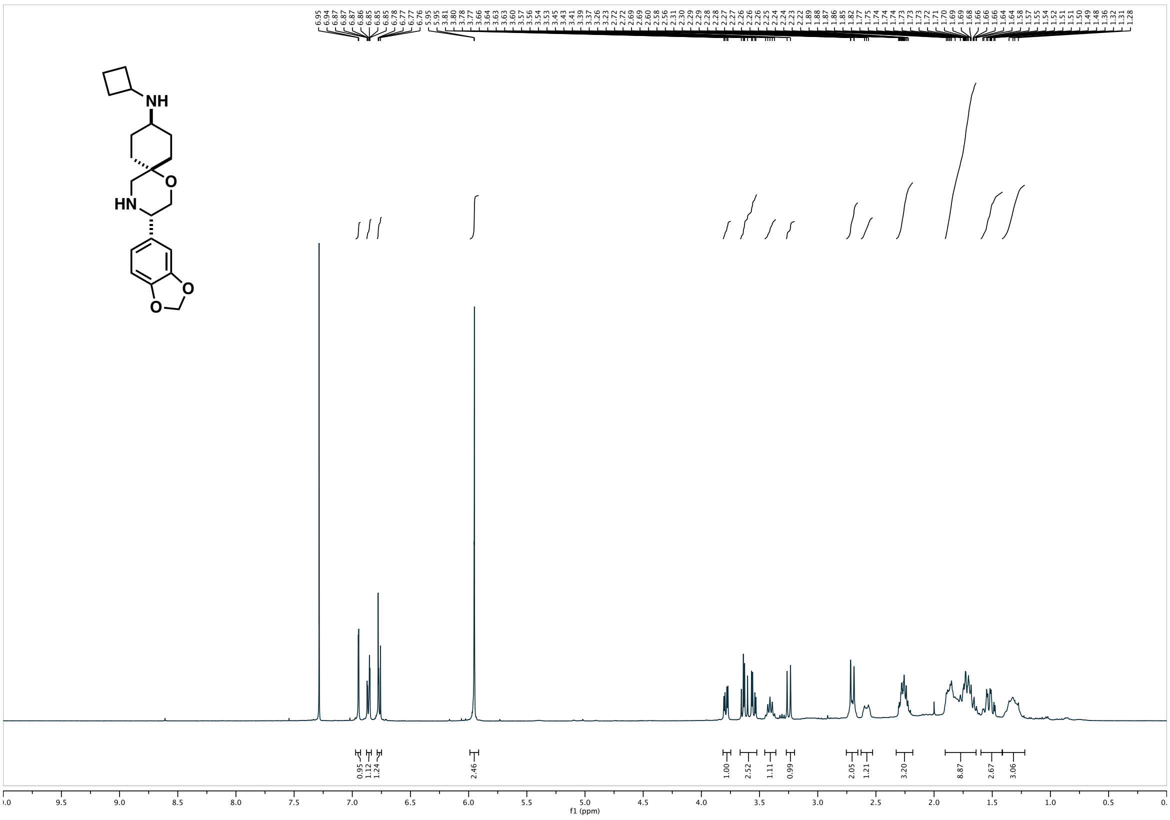

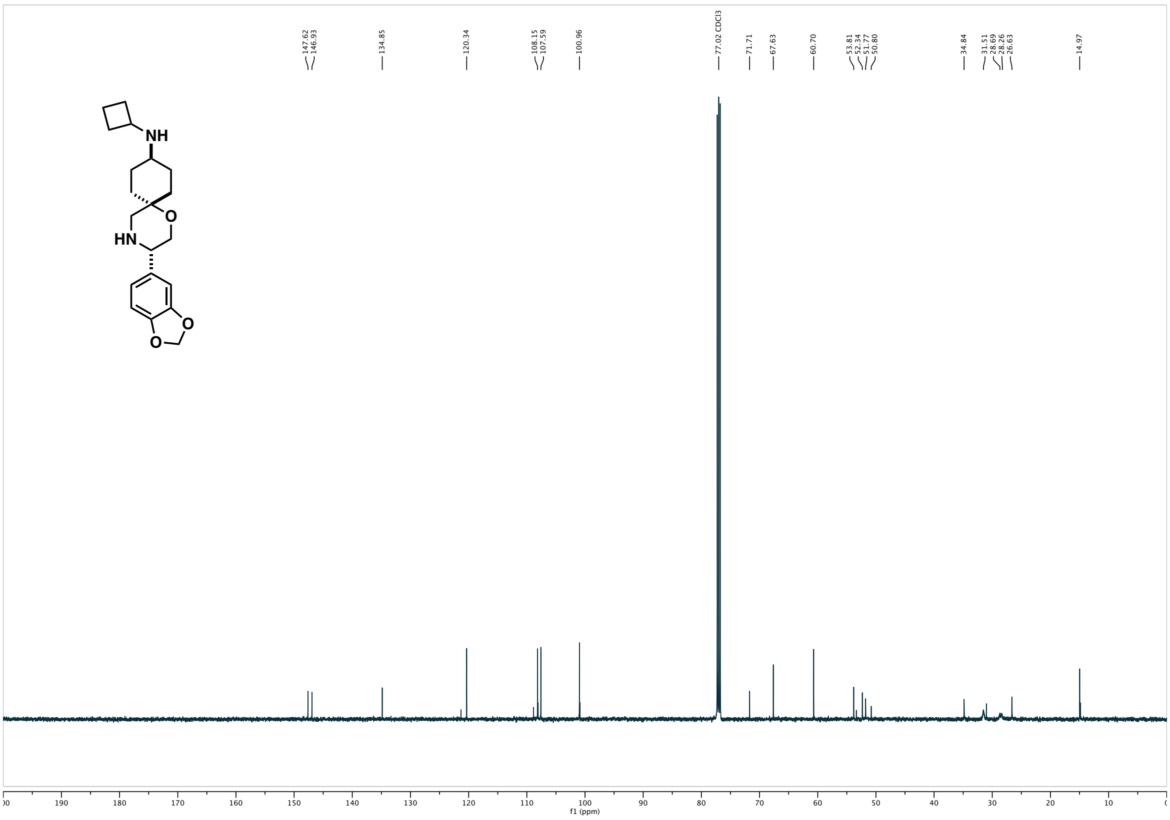

14-DiaA

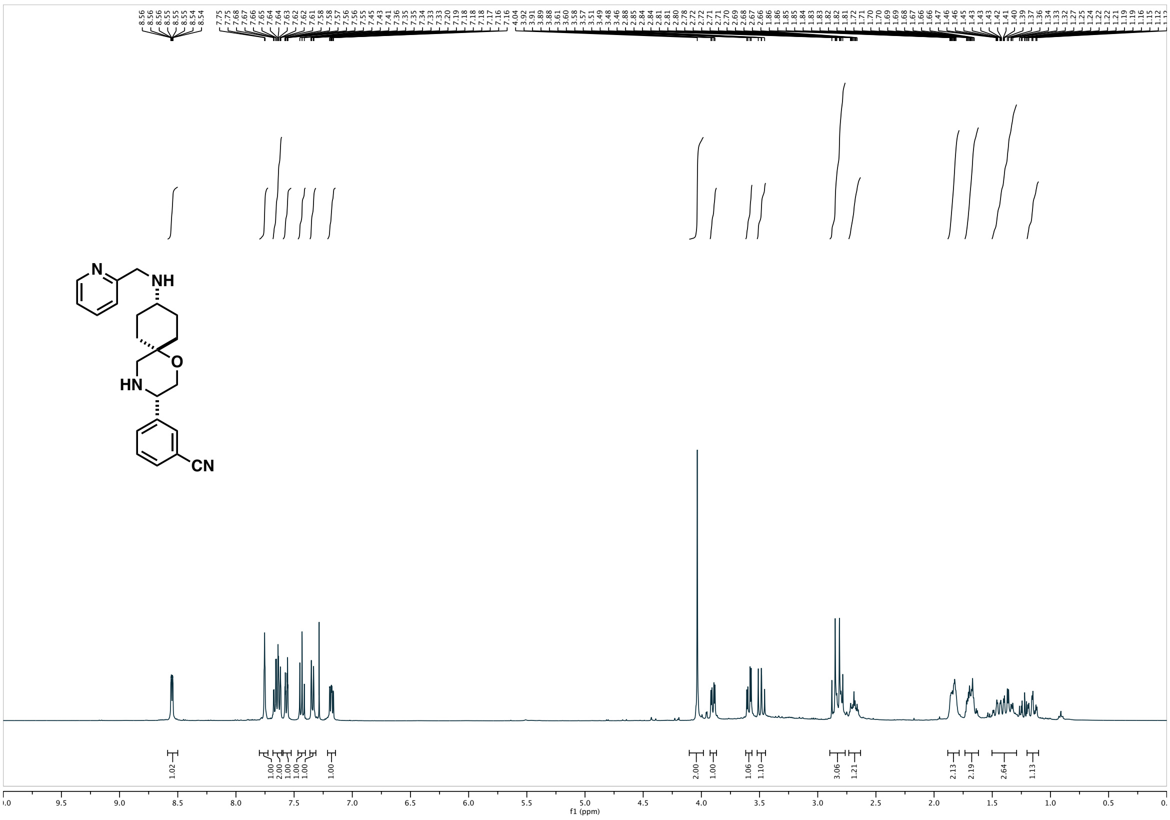
N
NH
O
HN
CN
f1 (ppm)

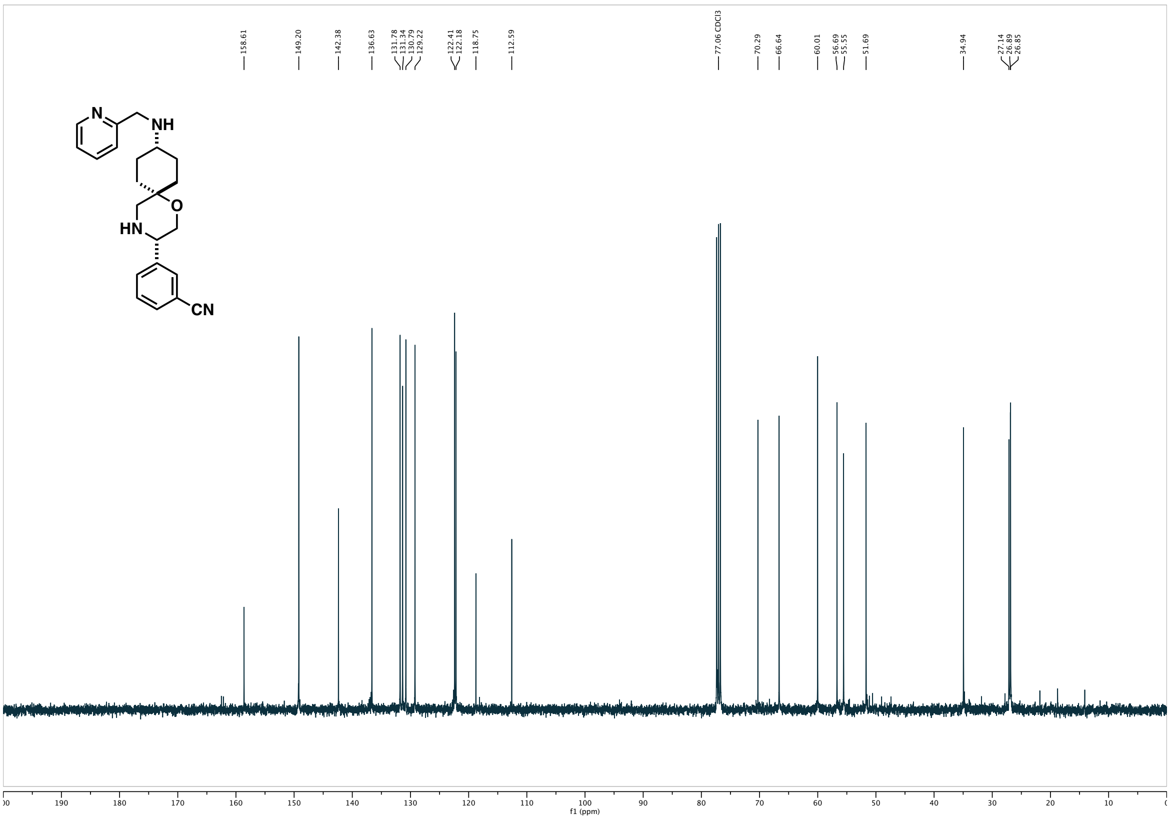
158.61
149.20
142.38
136.63
131.78
131.34
130.79
129.22
122.41
122.18
118.75
112.59
77.06 CDCl3
70.29
66.64
60.01
56.69
55.55
51.69
34.94
27.14
26.89
26.85
N
NH
O
HN
CN
f1 (ppm)

14-DiaB

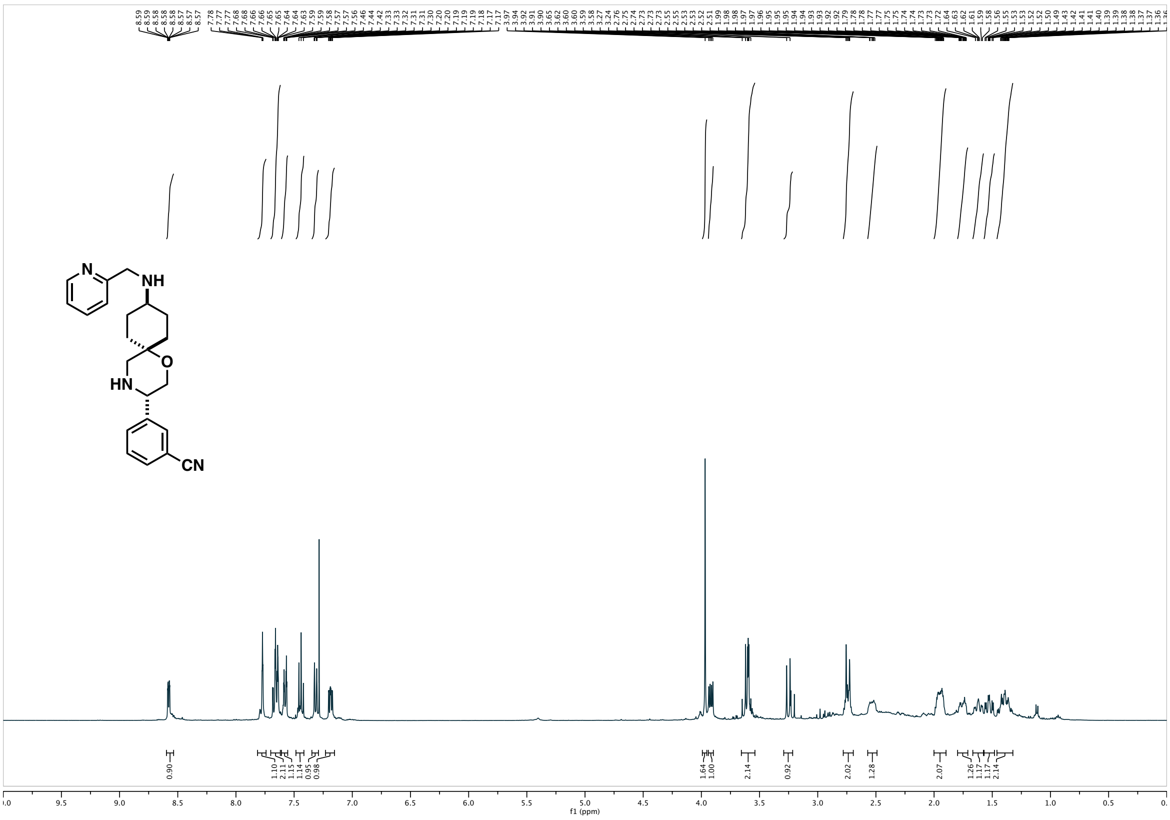
N
NH
O
HN
CN
f1 (ppm)

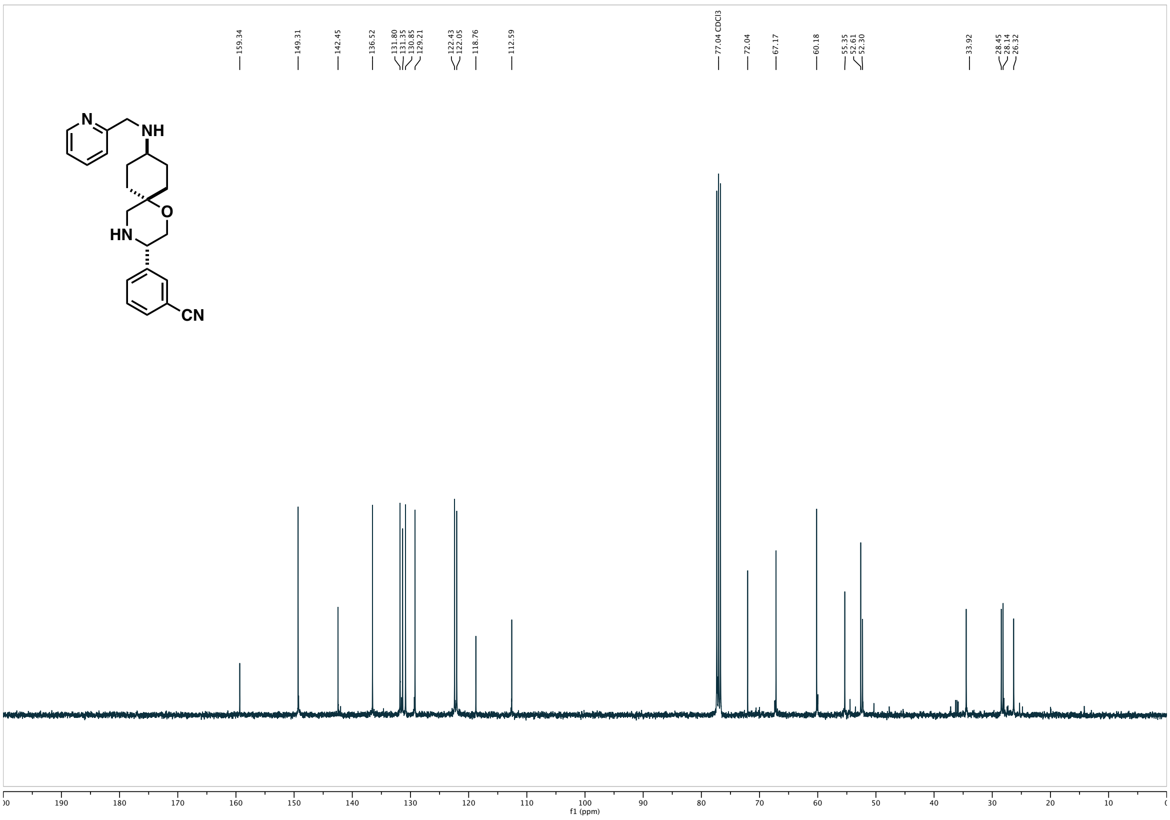
159.34
149.31
142.45
136.52
131.80
131.35
130.85
129.21
122.43
122.05
118.76
112.59
77.04 CDCl3
72.04
67.17
60.18
55.35
52.61
52.30
33.92
28.45
28.14
26.32
N
NH
O
HN
CN
f1 (ppm)

15-DiaA

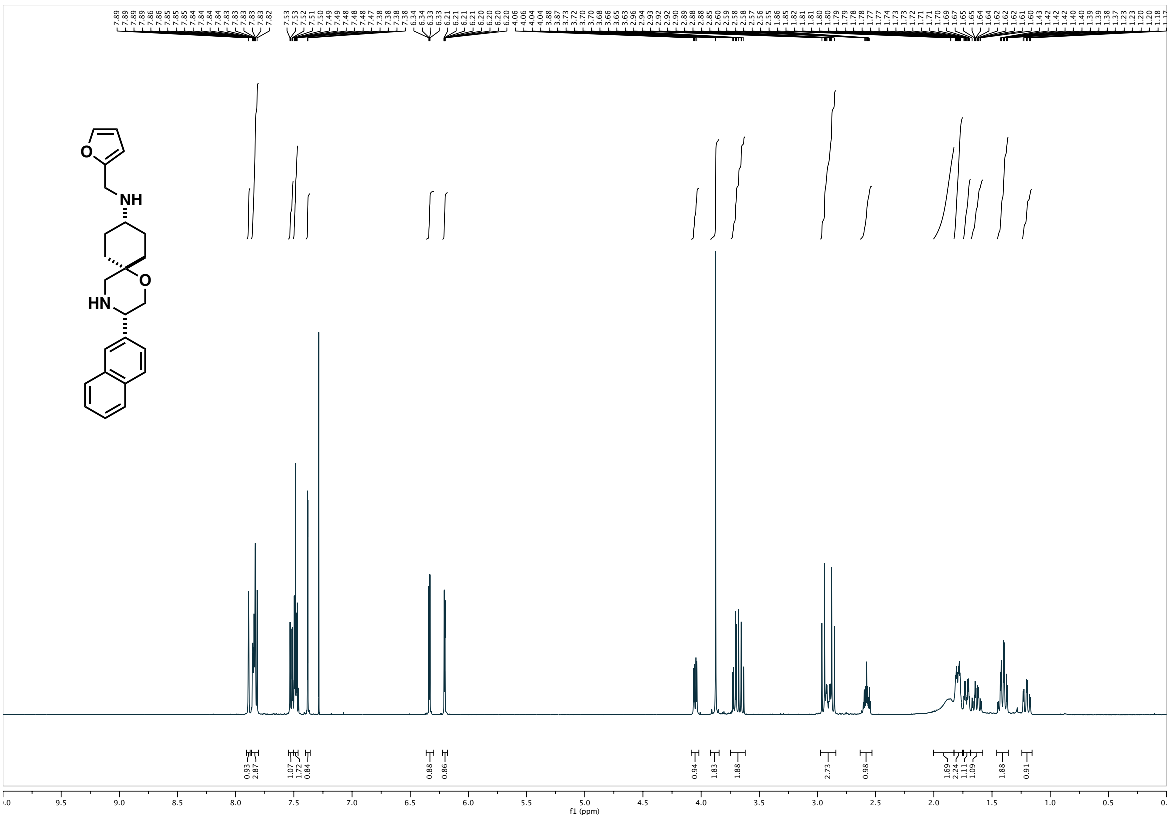

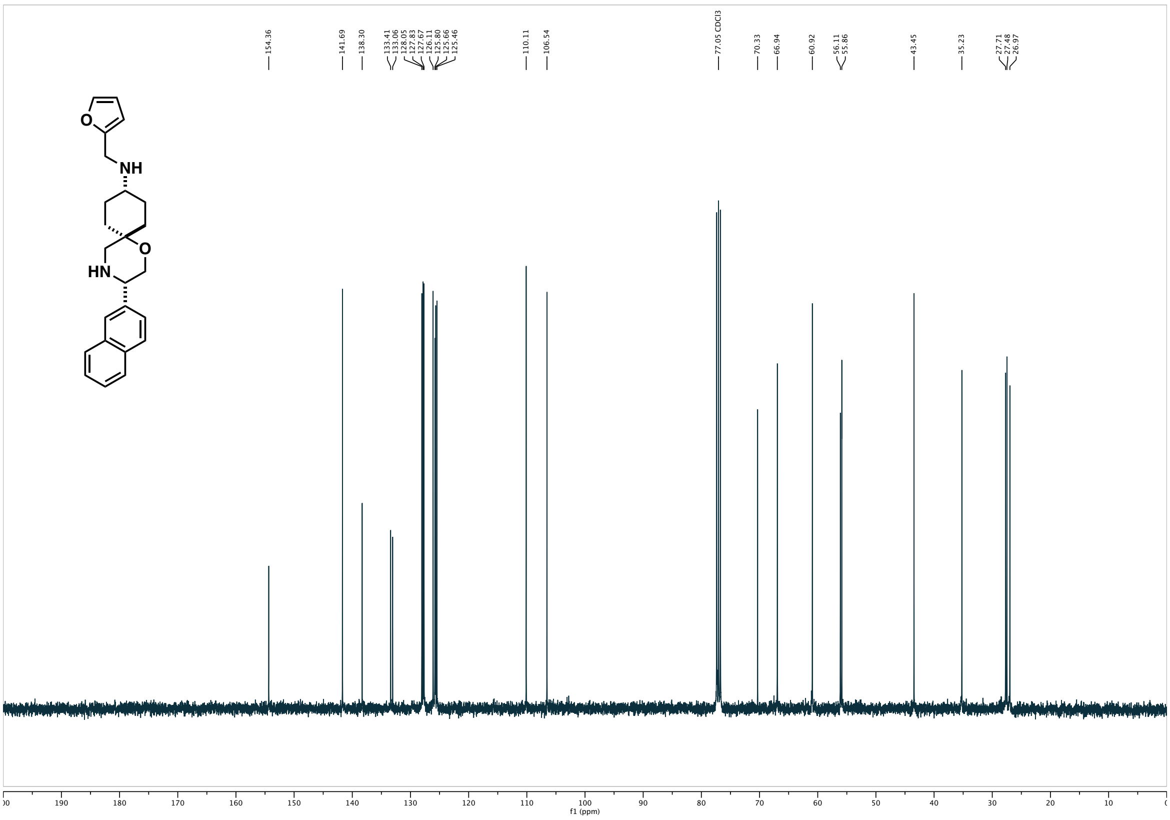

15-DiaB

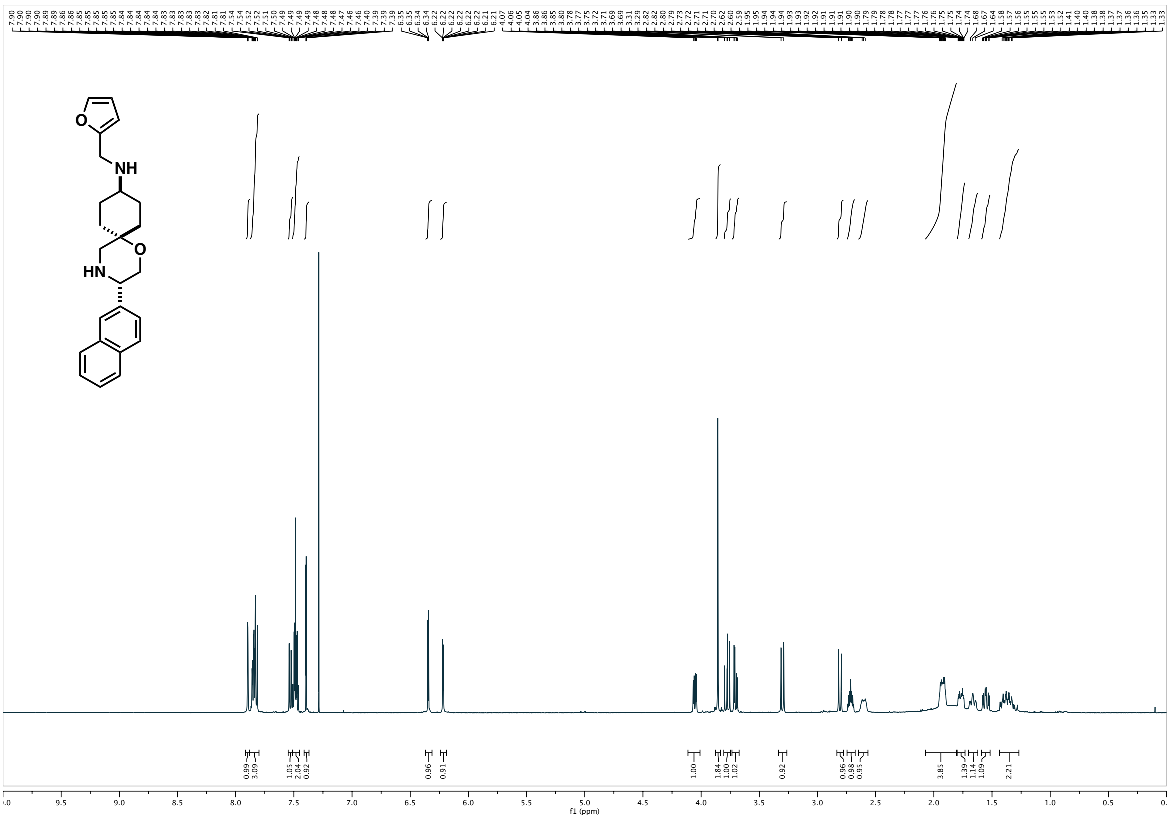
O
NH
O
HN
f1 (ppm)

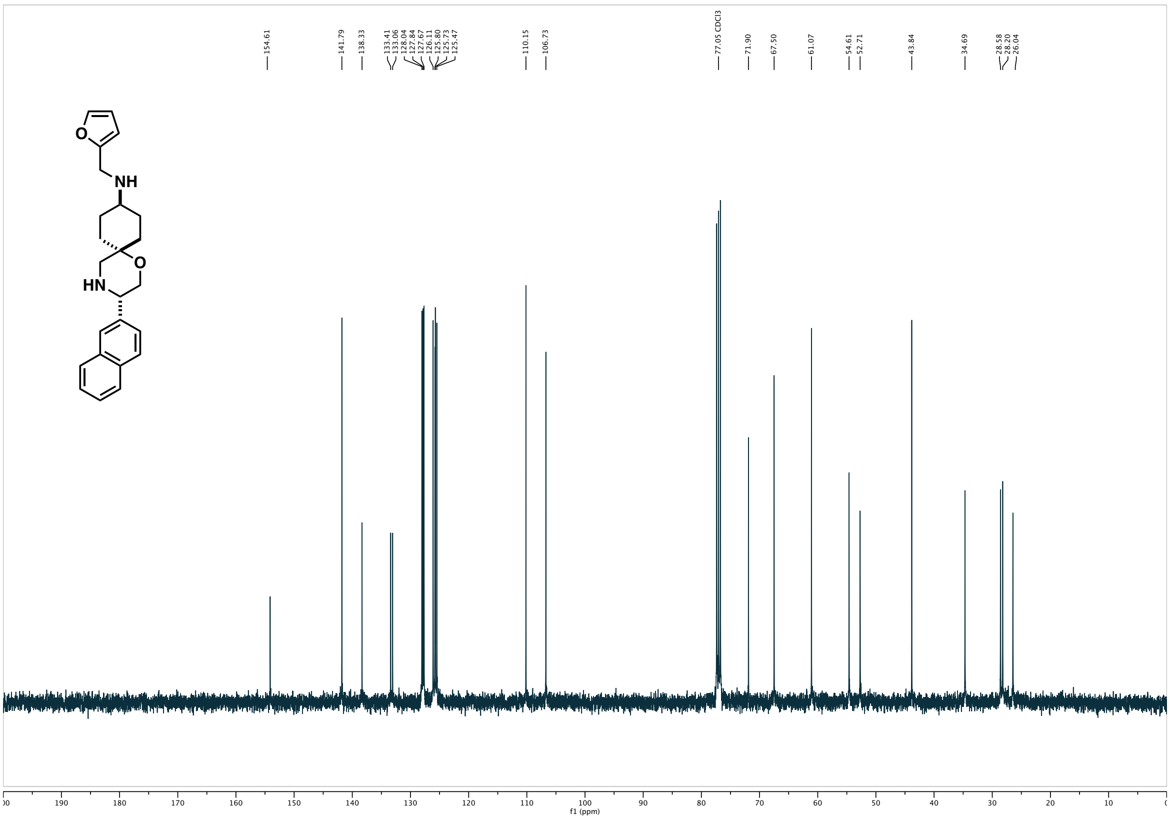
154.61
141.79
138.33
133.41
133.06
128.04
127.84
127.67
126.11
125.80
125.73
125.47
110.15
106.73
77.05 CDCl3
71.90
67.50
61.07
54.61
52.71
43.84
34.69
28.58
28.20
26.04
O
NH
O
HN
f1 (ppm)

16-DiaA

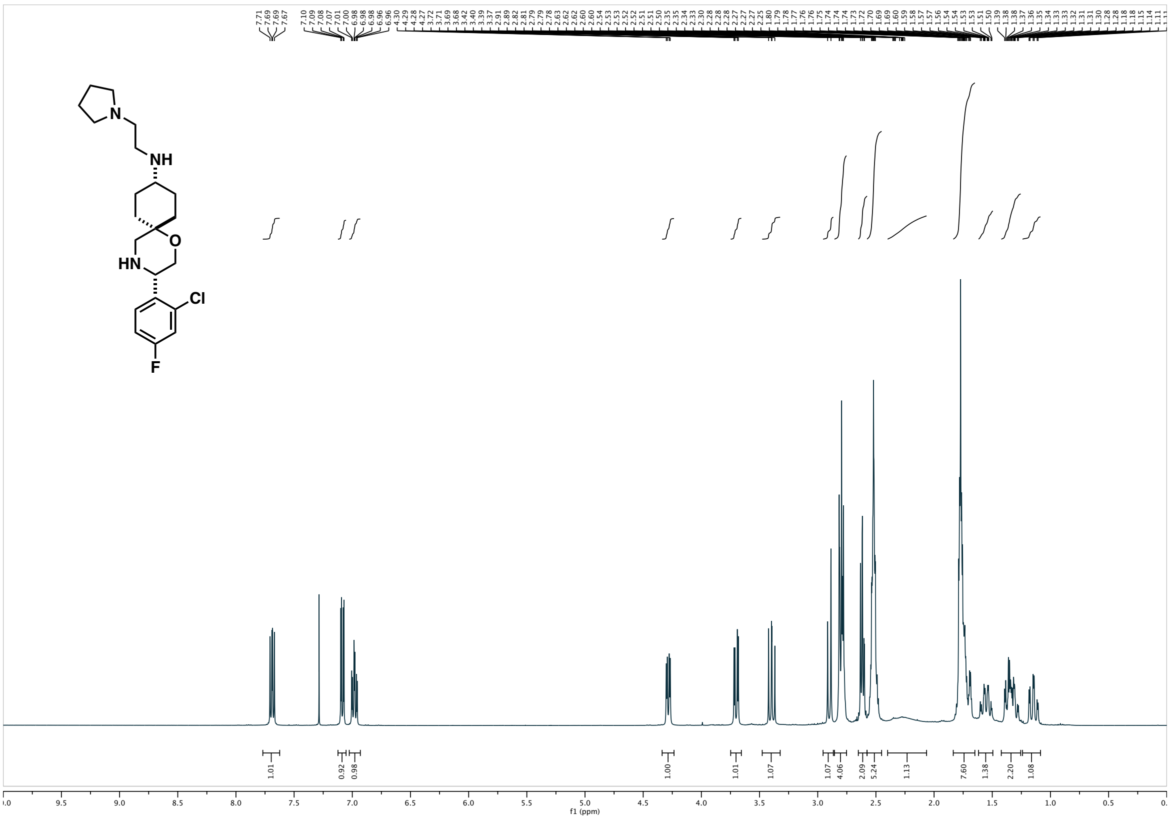

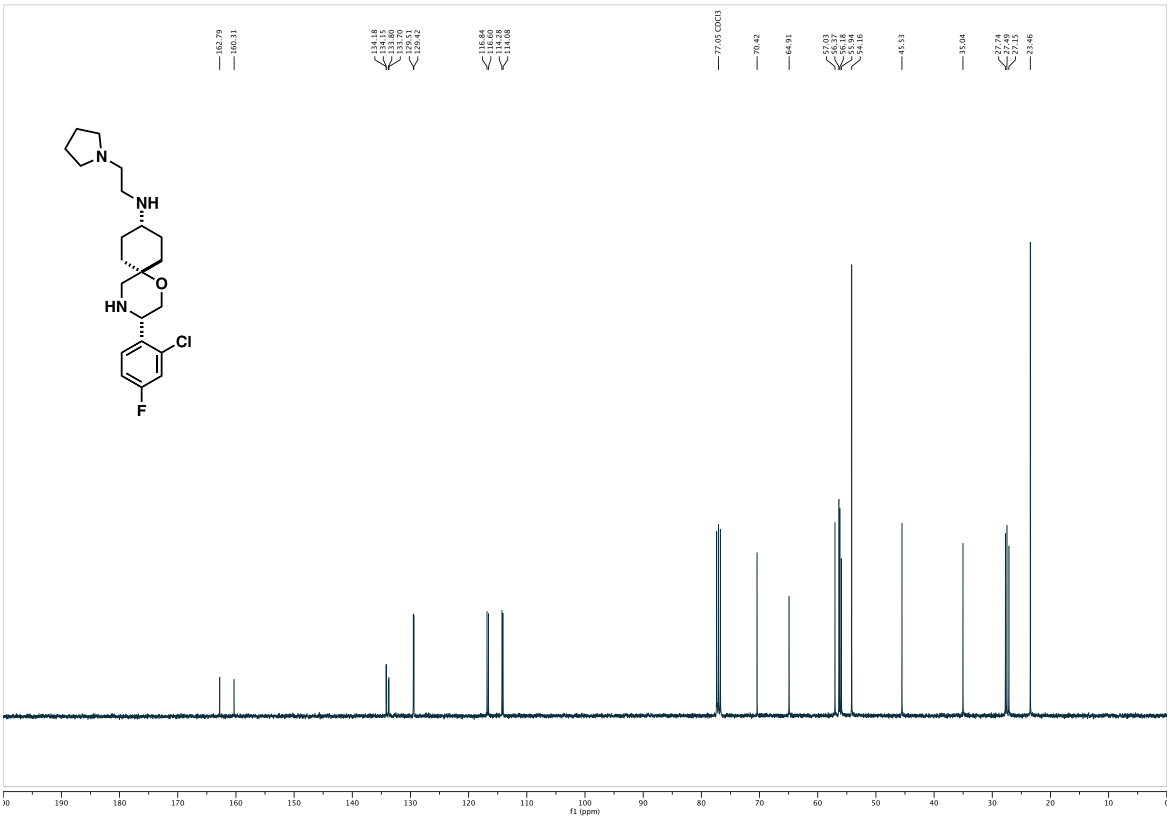

16-DiaB

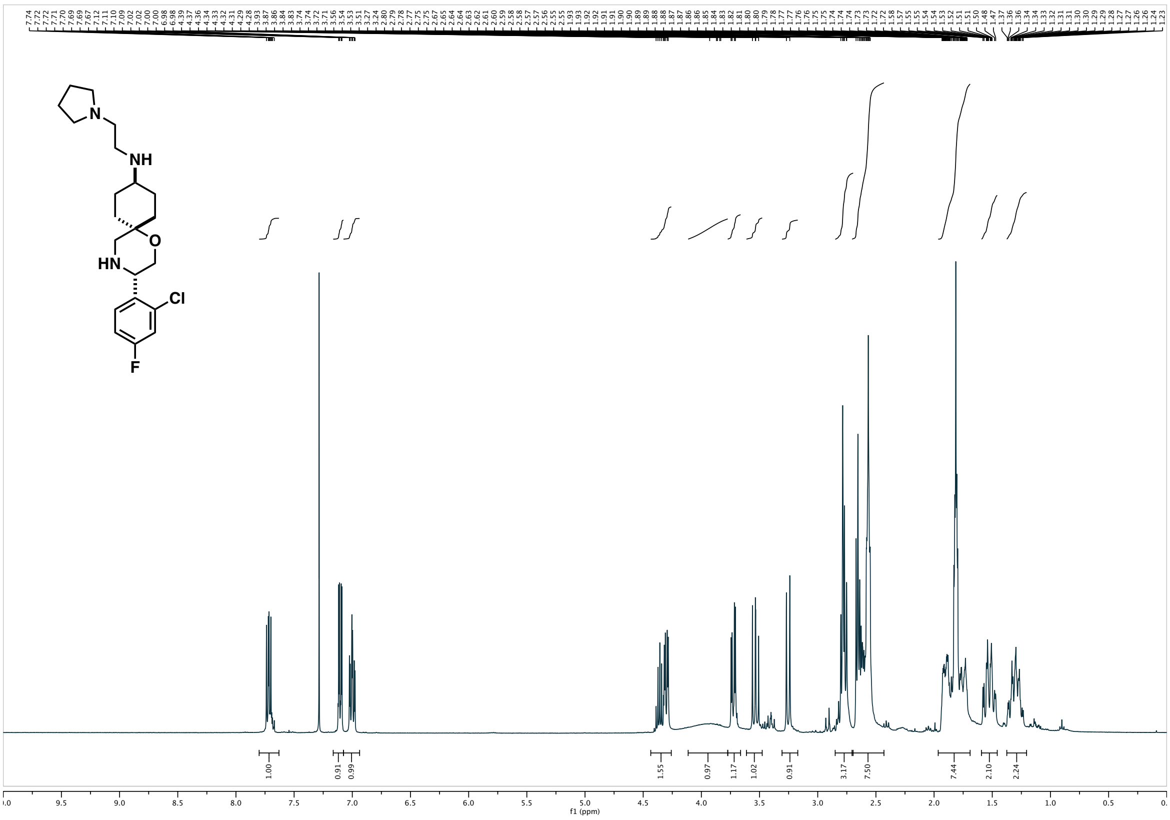
N
NH
O
HN
Cl
F
1.00
0.91
0.99
1.55
0.97
1.17
1.02
0.91
3.17
7.50
7.44
2.10
2.24
f1 (ppm)

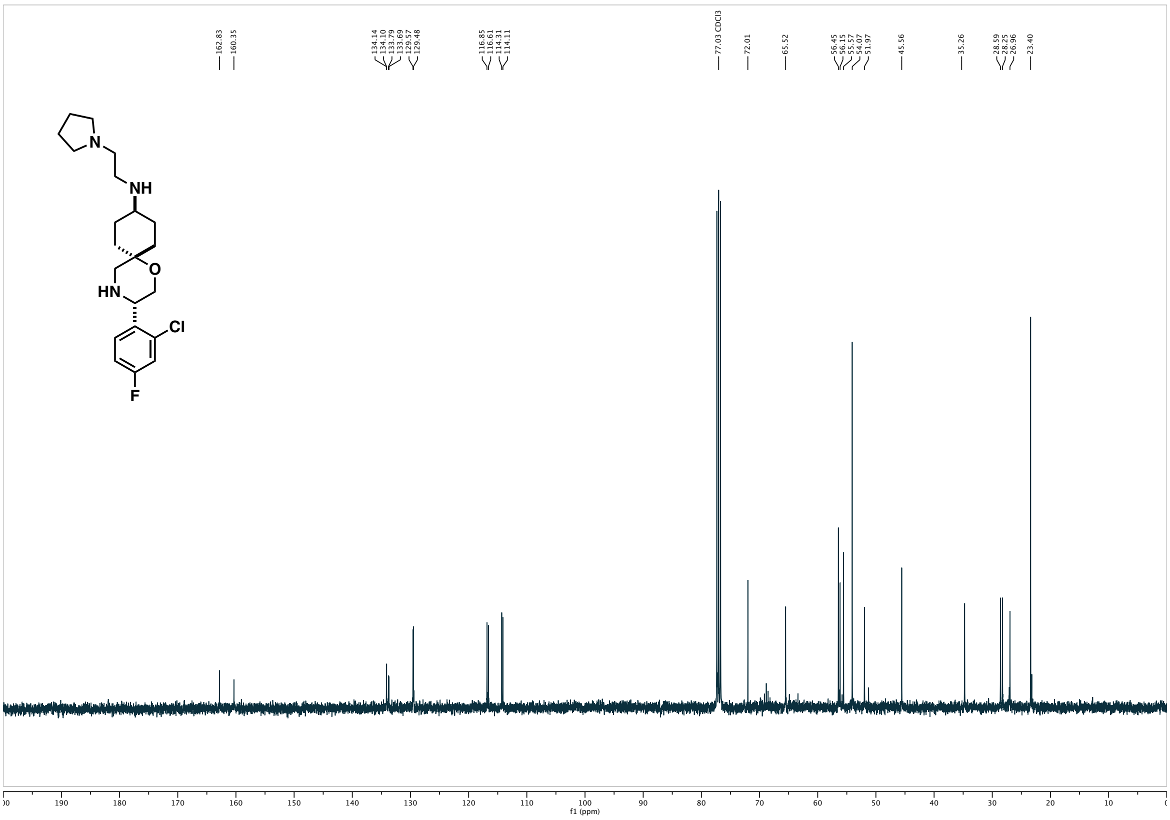
162.83
160.35
134.14
134.10
133.79
133.69
129.57
129.48
116.85
116.61
114.31
114.11
77.03 CDCl3
72.01
65.52
56.45
56.15
55.57
54.07
51.97
45.56
35.26
28.59
28.25
26.96
23.40
N
NH
O
HN
Cl
F
f1 (ppm)

17-DiaA

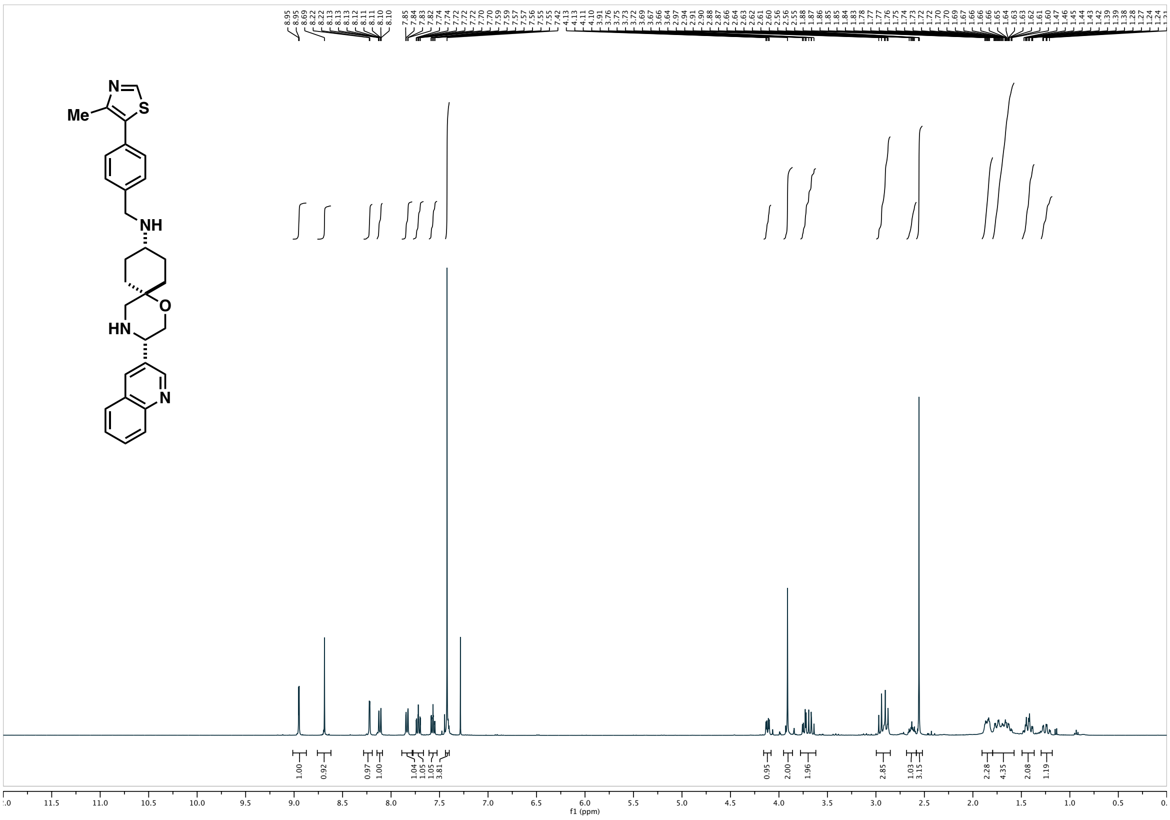

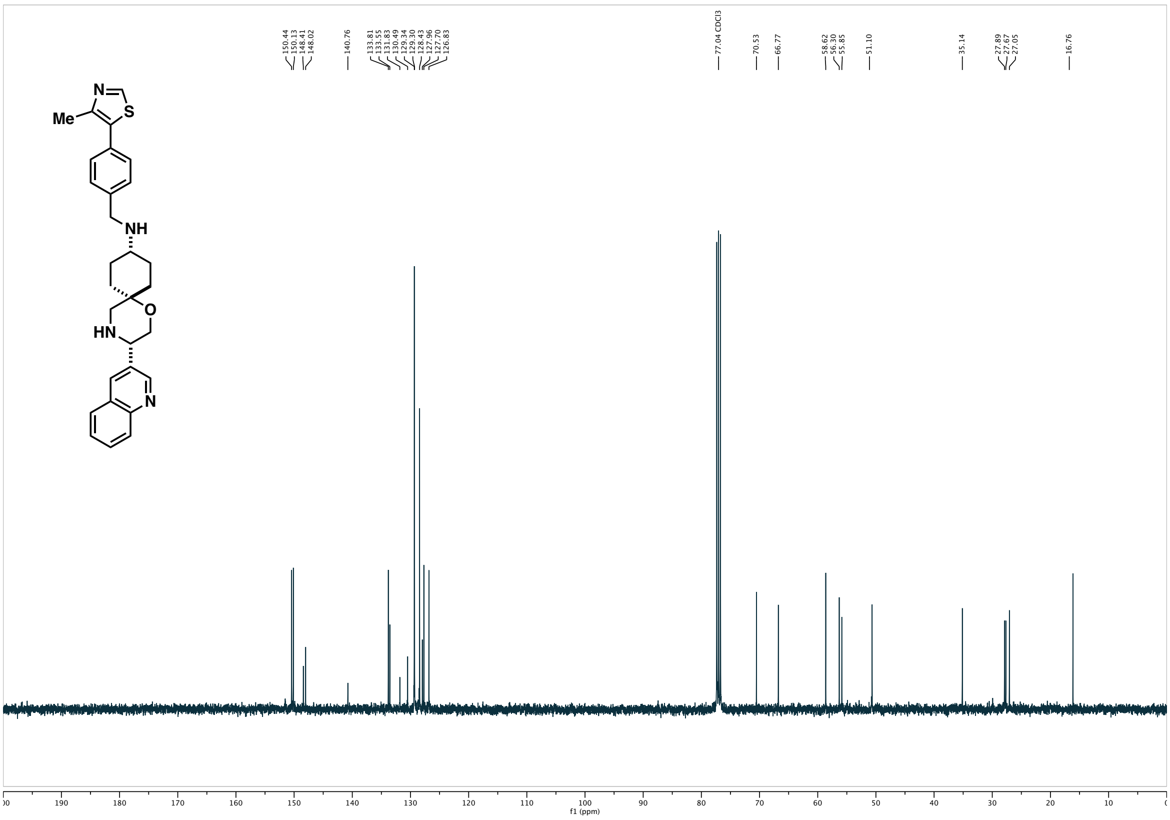

17-DiaB

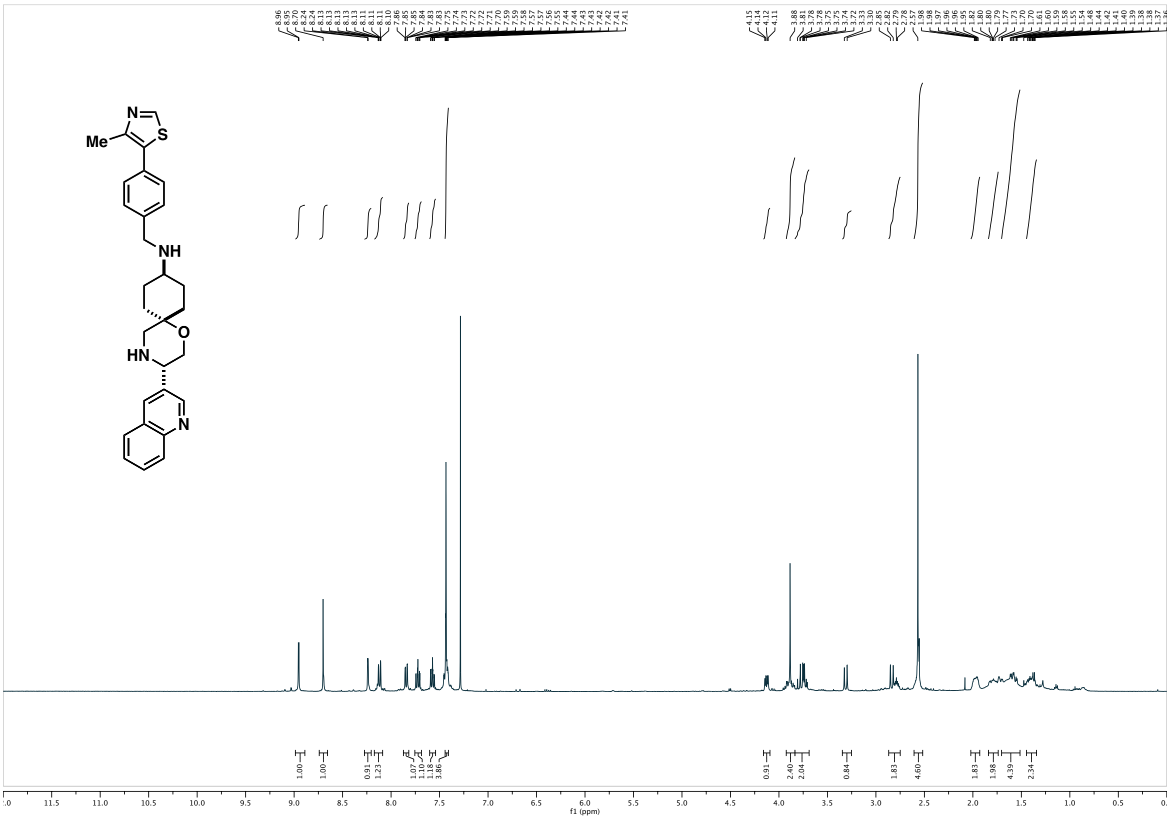

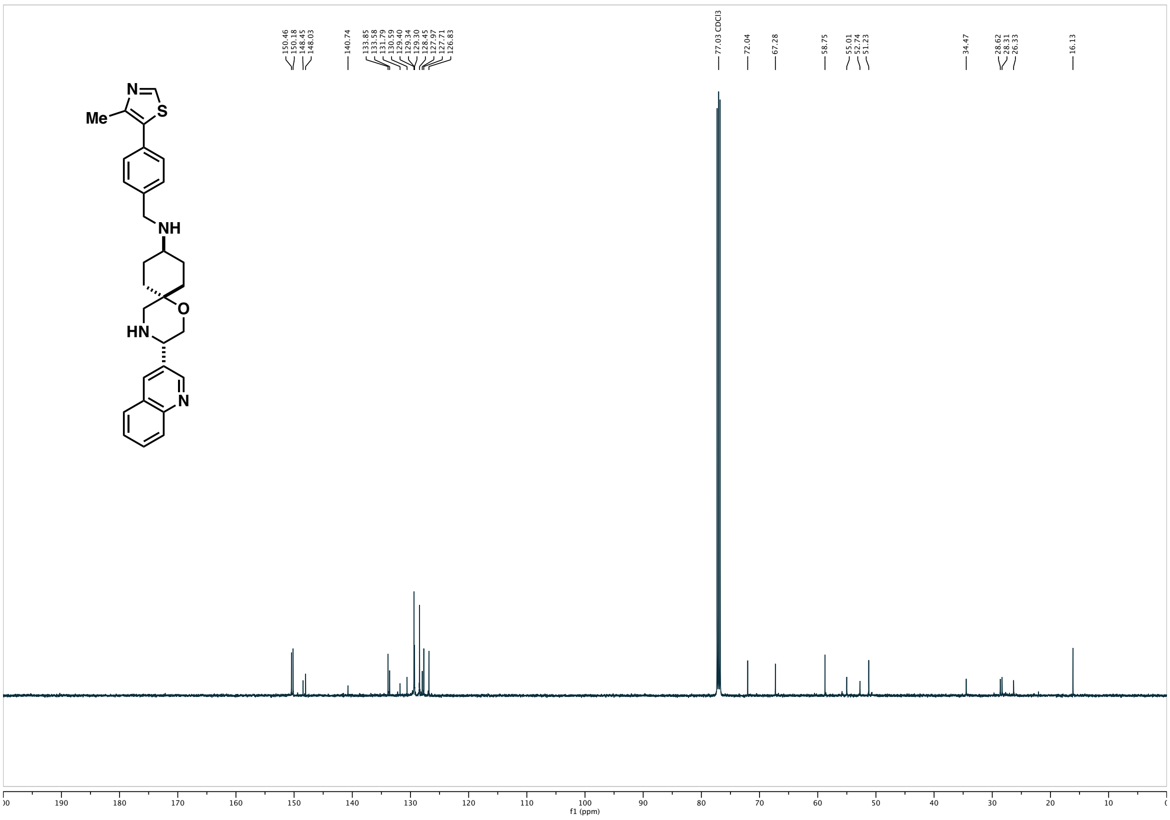

18-DiaA

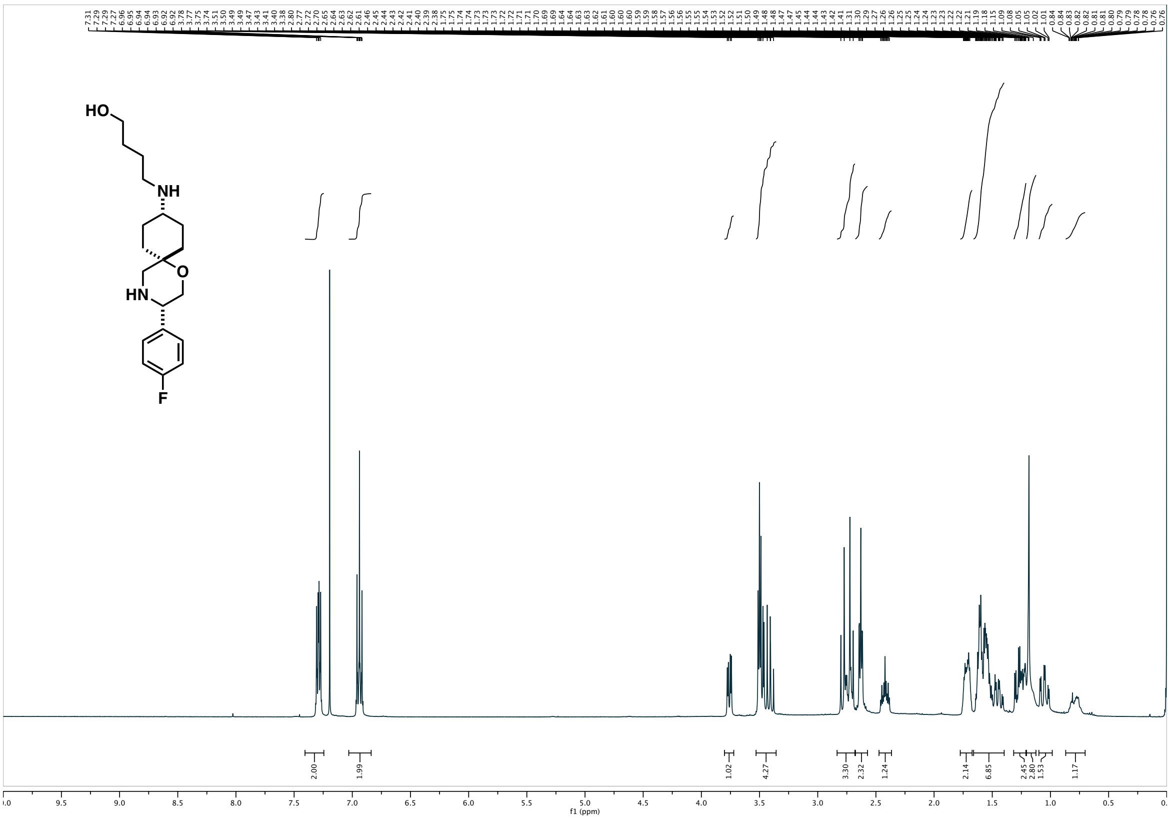

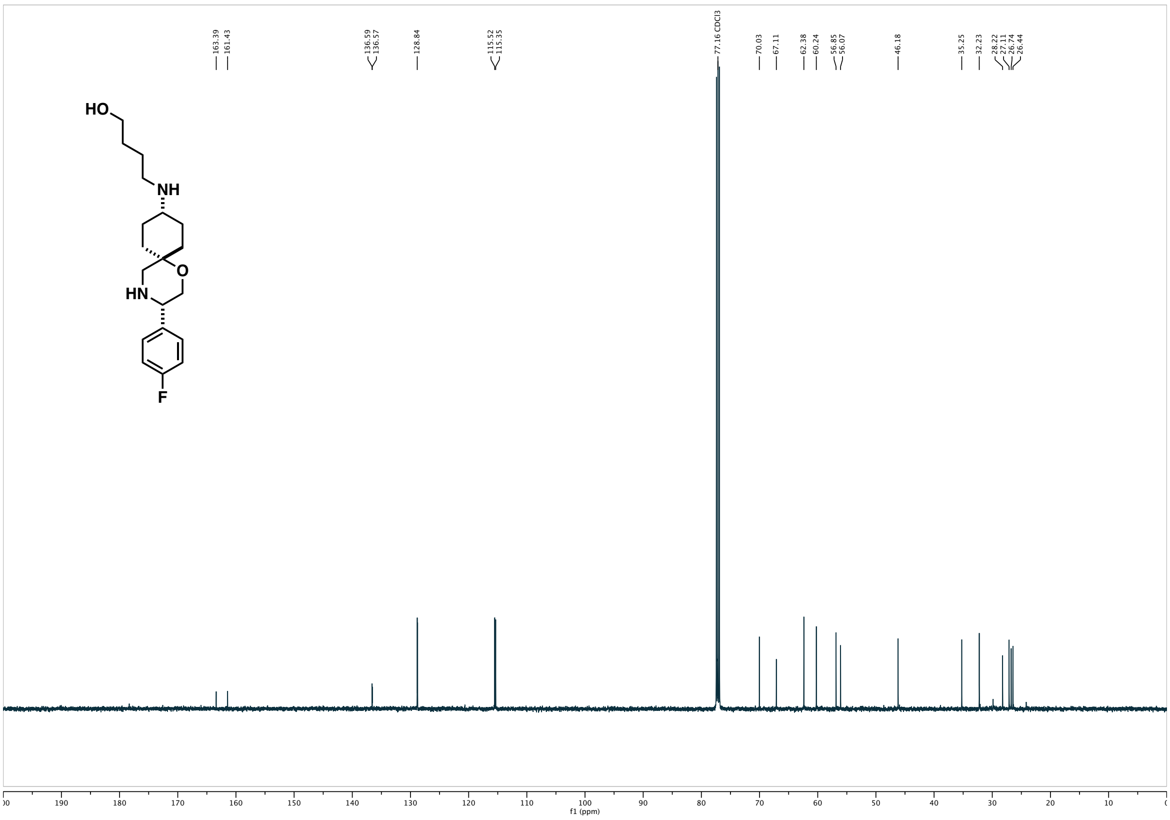

18-DiaB

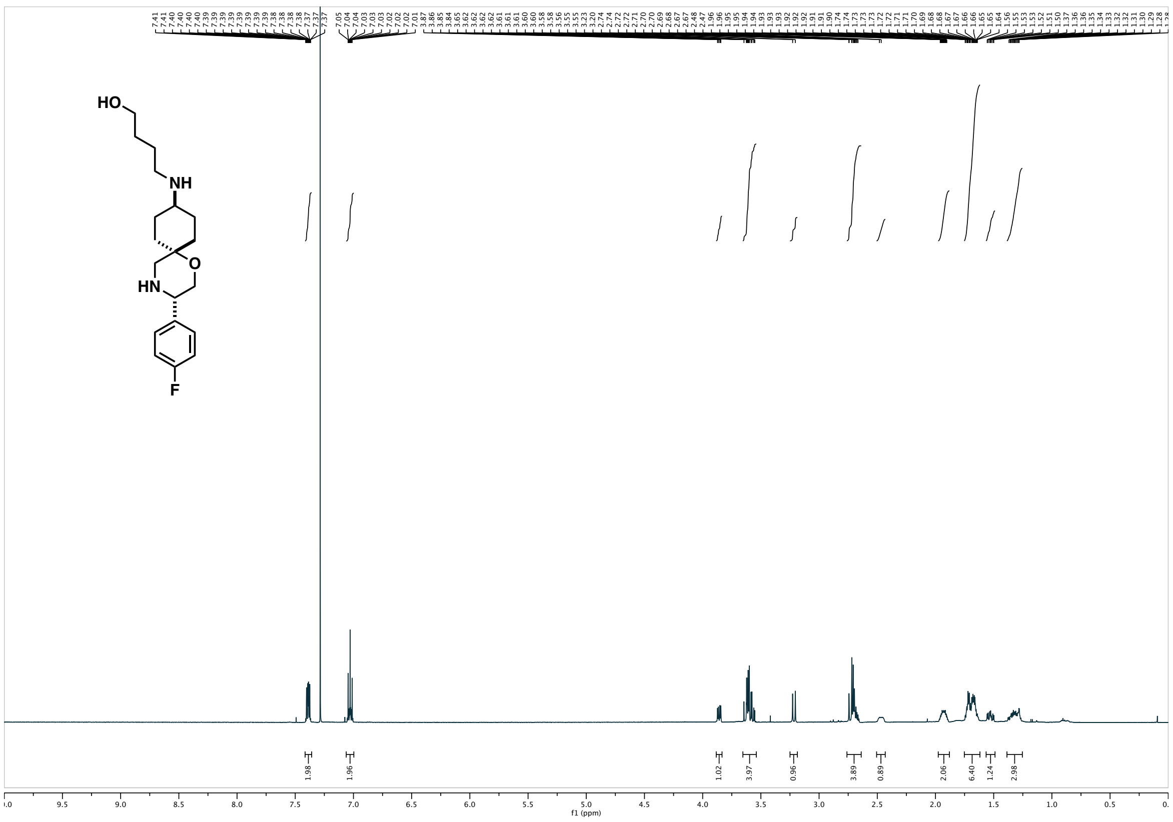

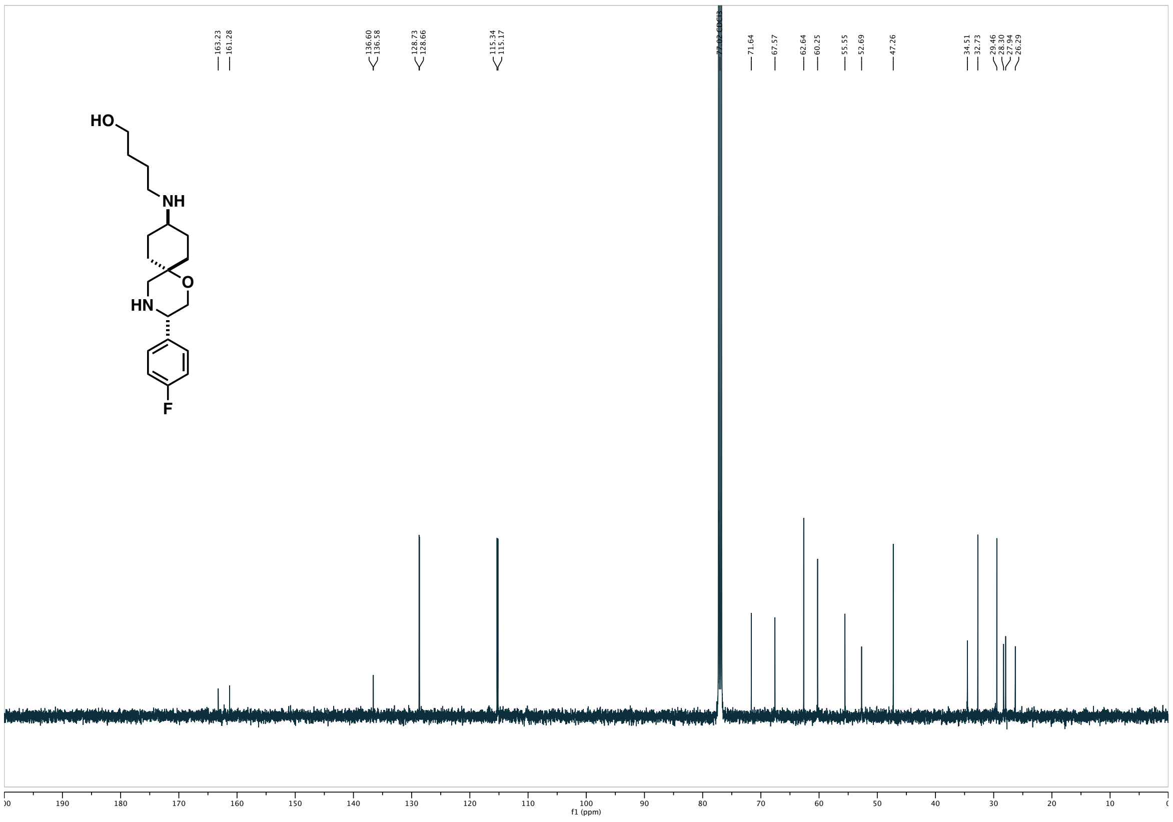

19-DiaA

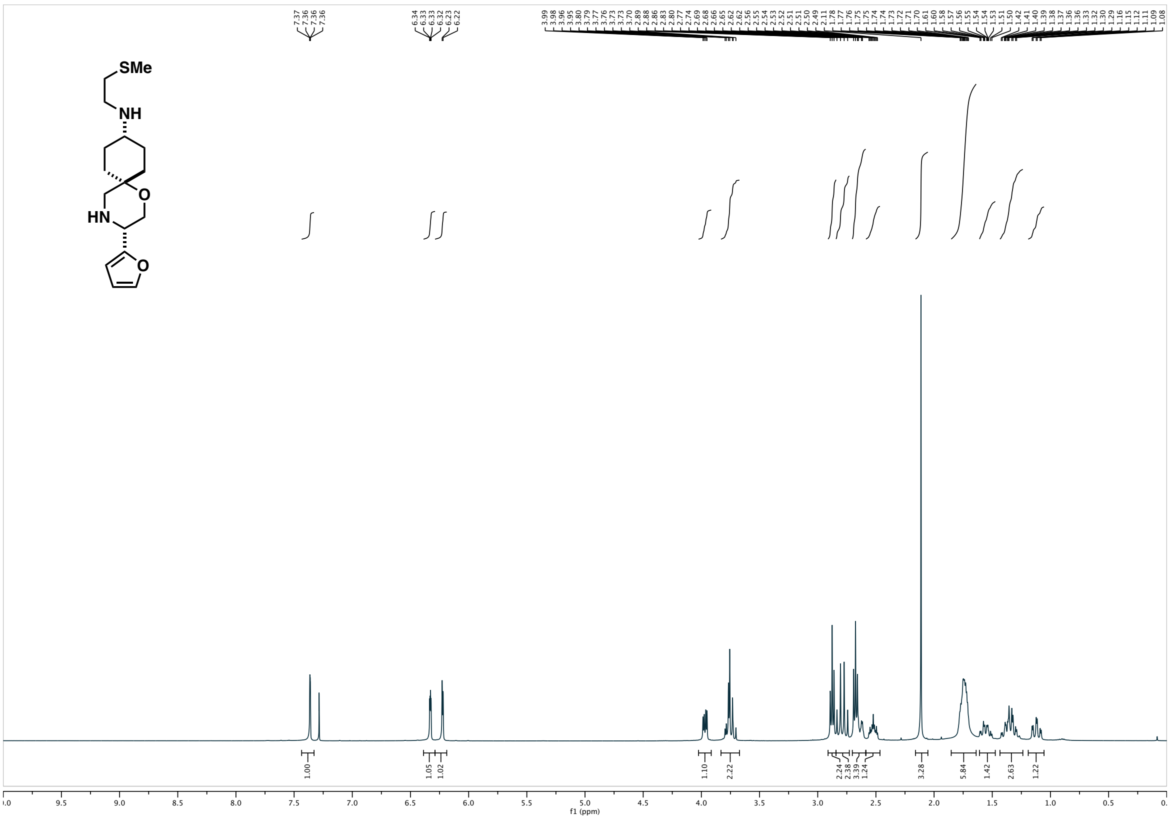

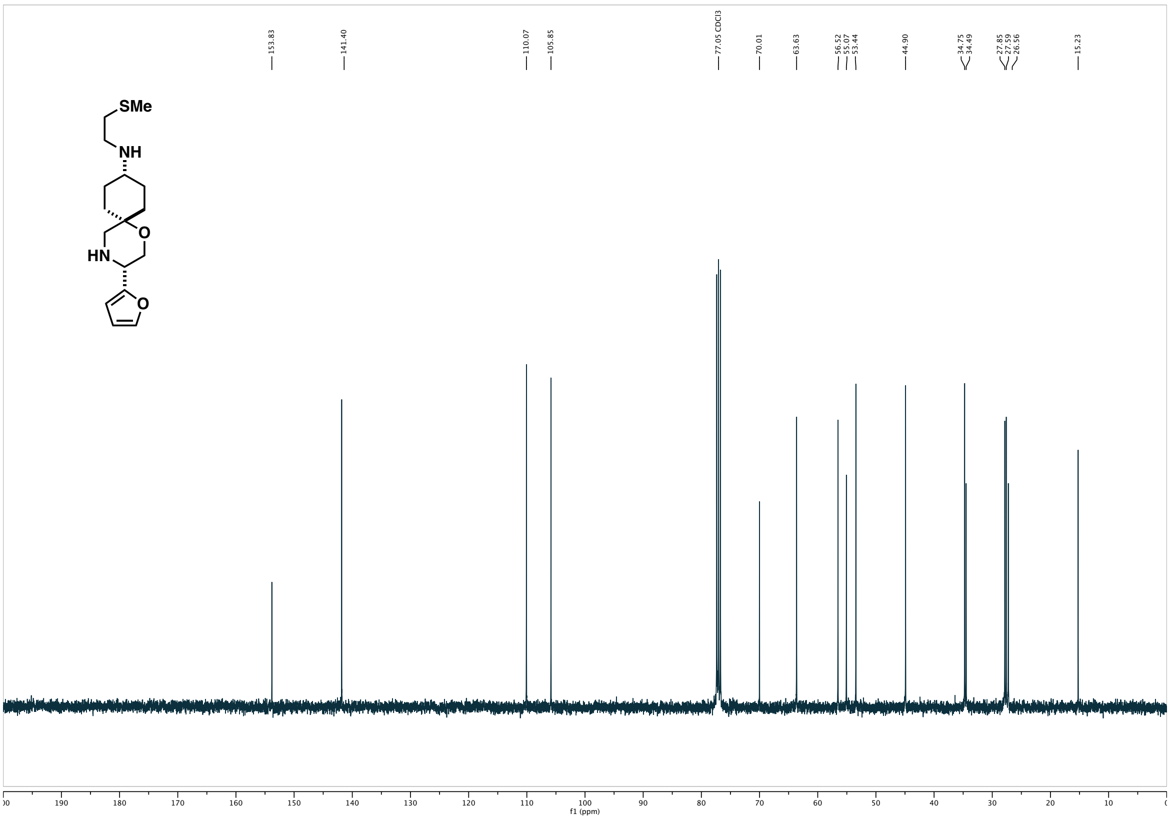

19-DiaB

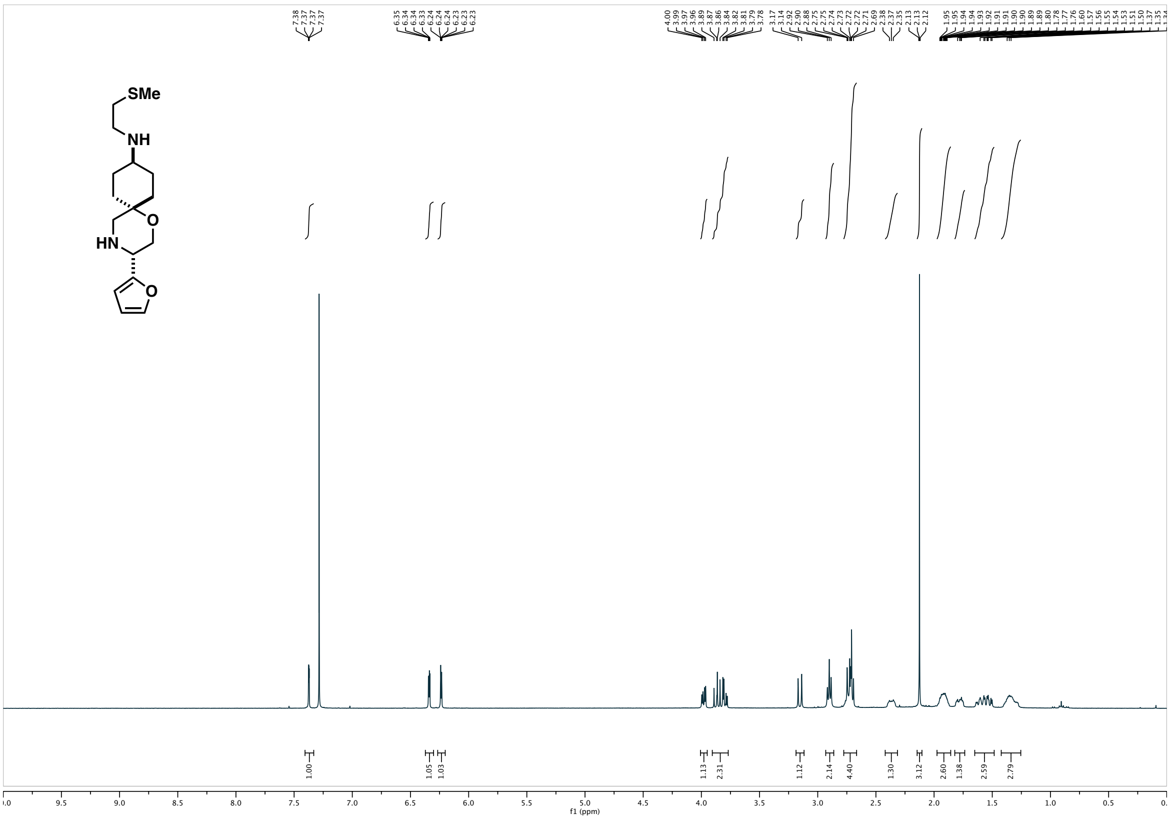

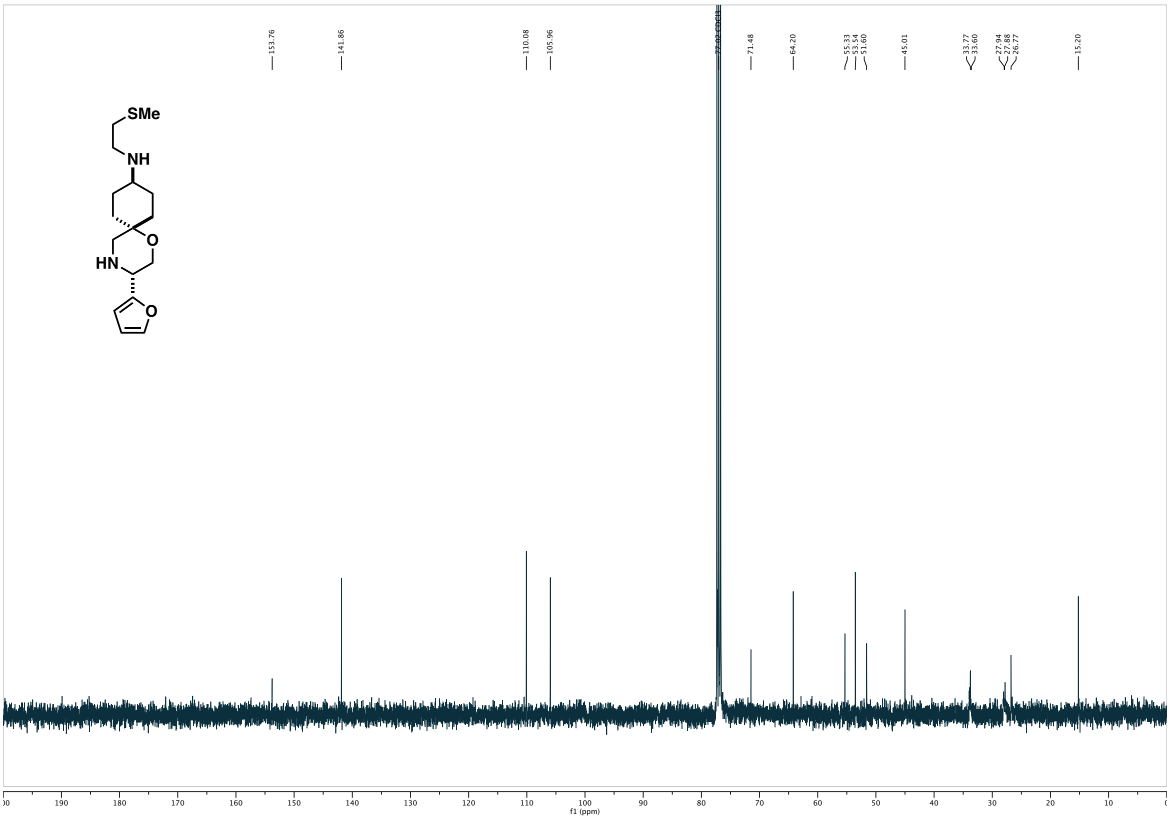

20-DiaA

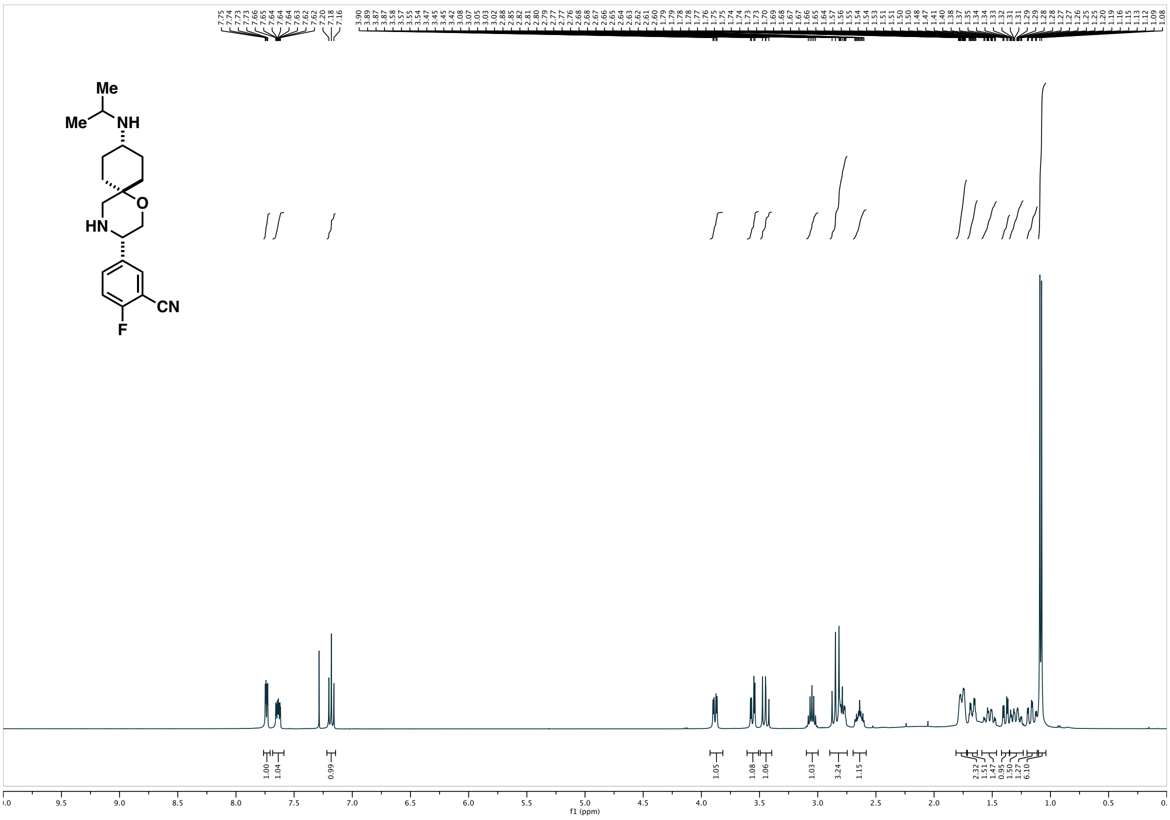
Me
Me
NH
O
HN
CN
F
f1 (ppm)

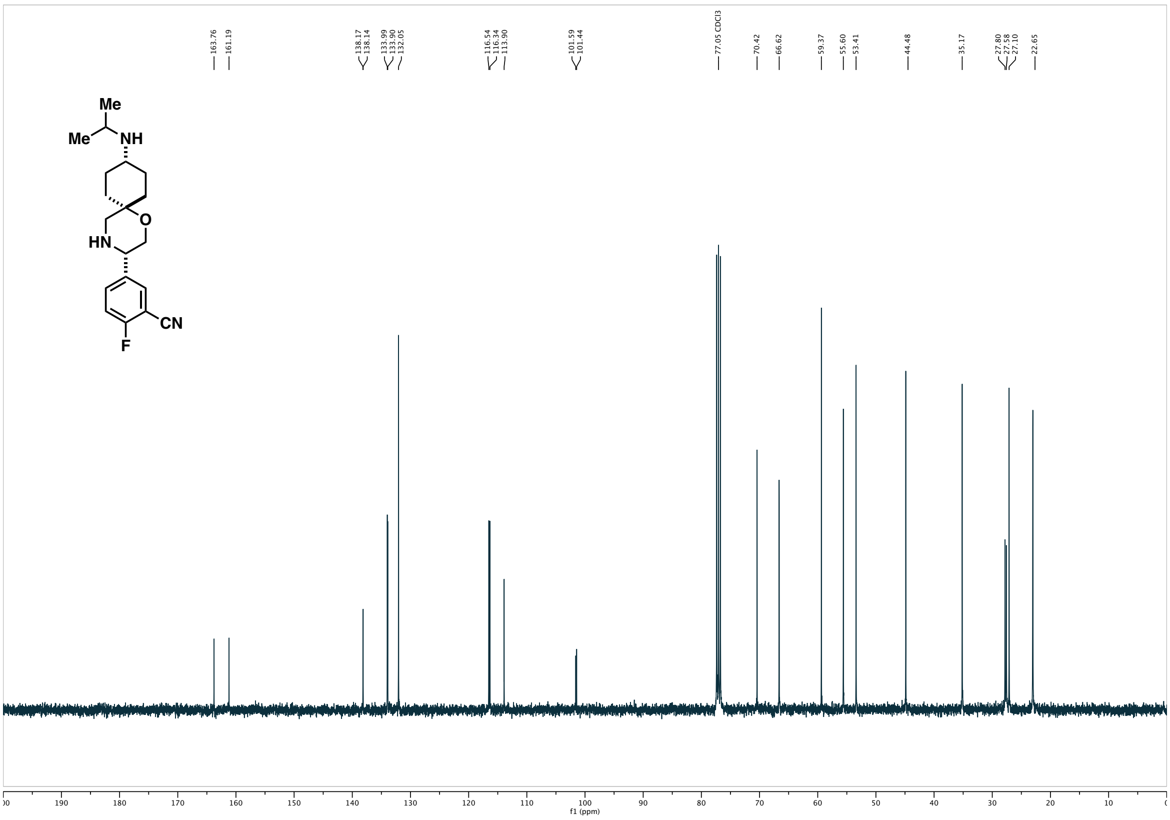
163.76
161.19
138.17
138.14
133.99
133.90
132.05
116.54
116.34
113.90
101.59
101.44
77.05 CDCl3
70.42
66.62
59.37
55.60
53.41
44.48
35.17
27.80
27.58
27.10
22.65
Me
Me
NH
O
HN
CN
F
f1 (ppm)

20-DiaB

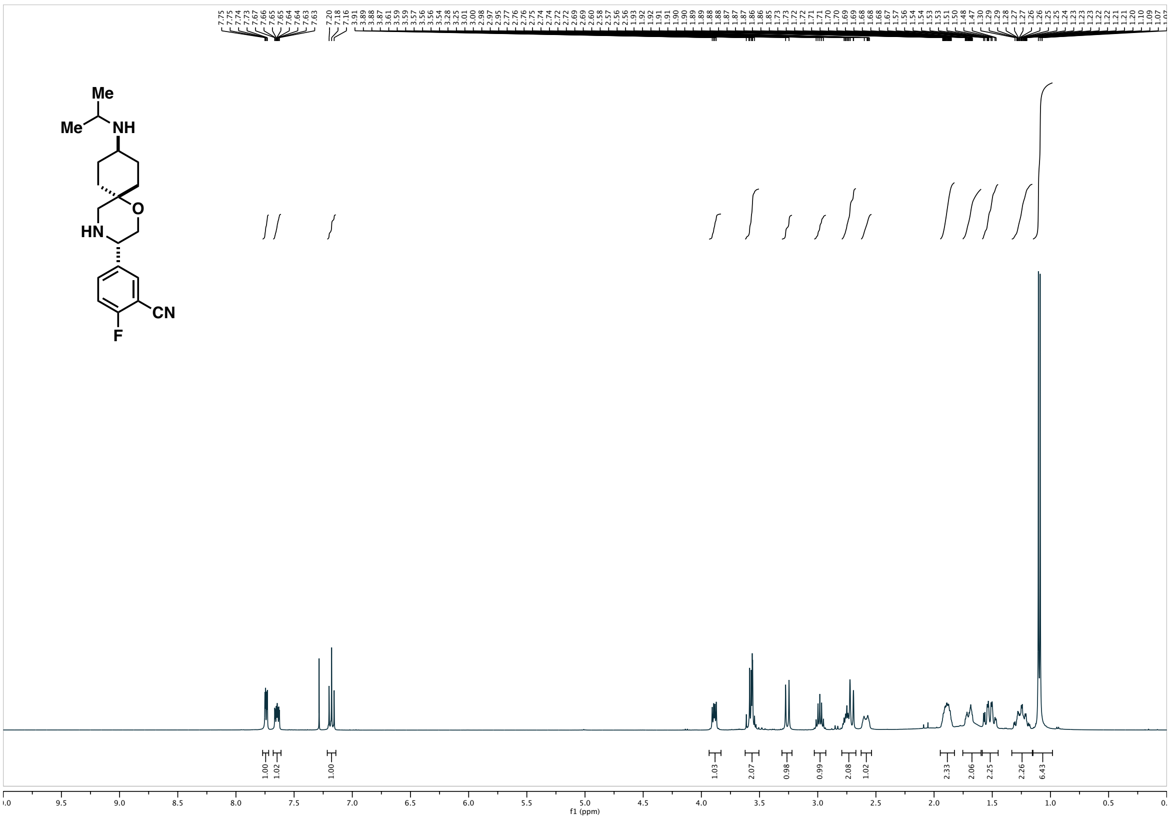

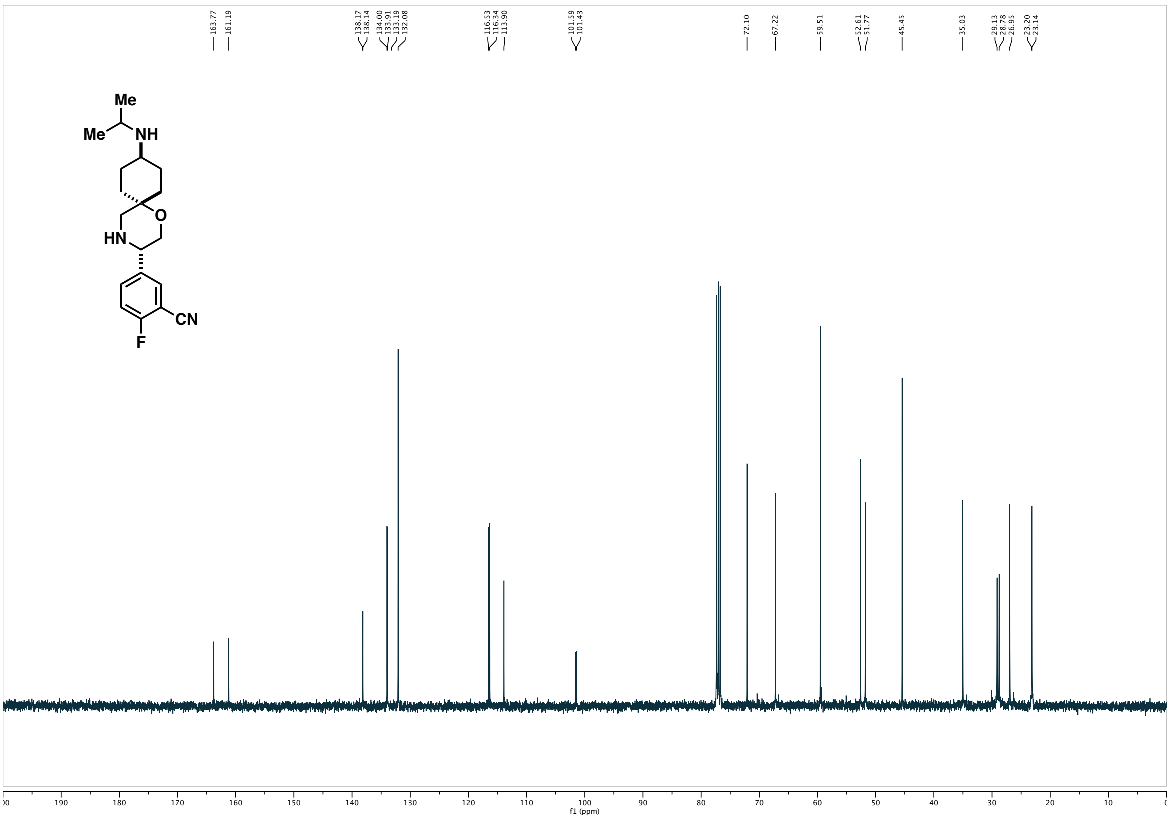

21-DiaA

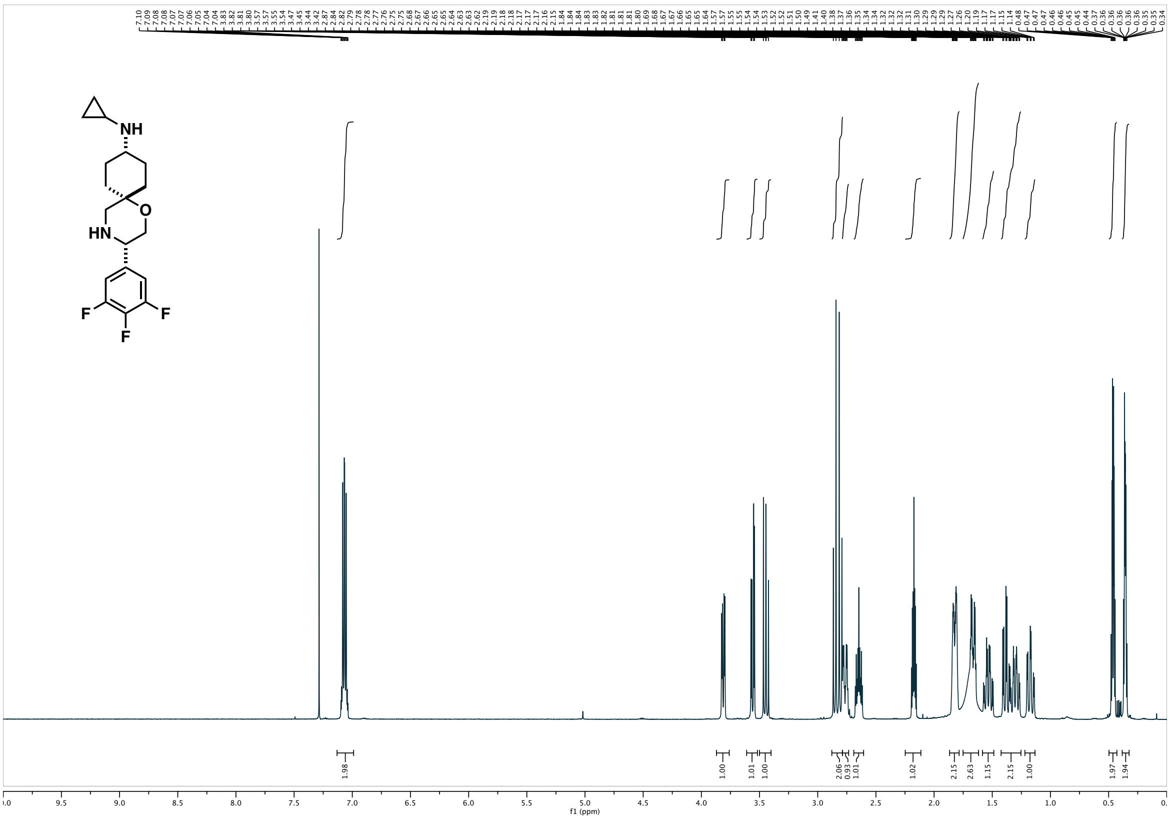

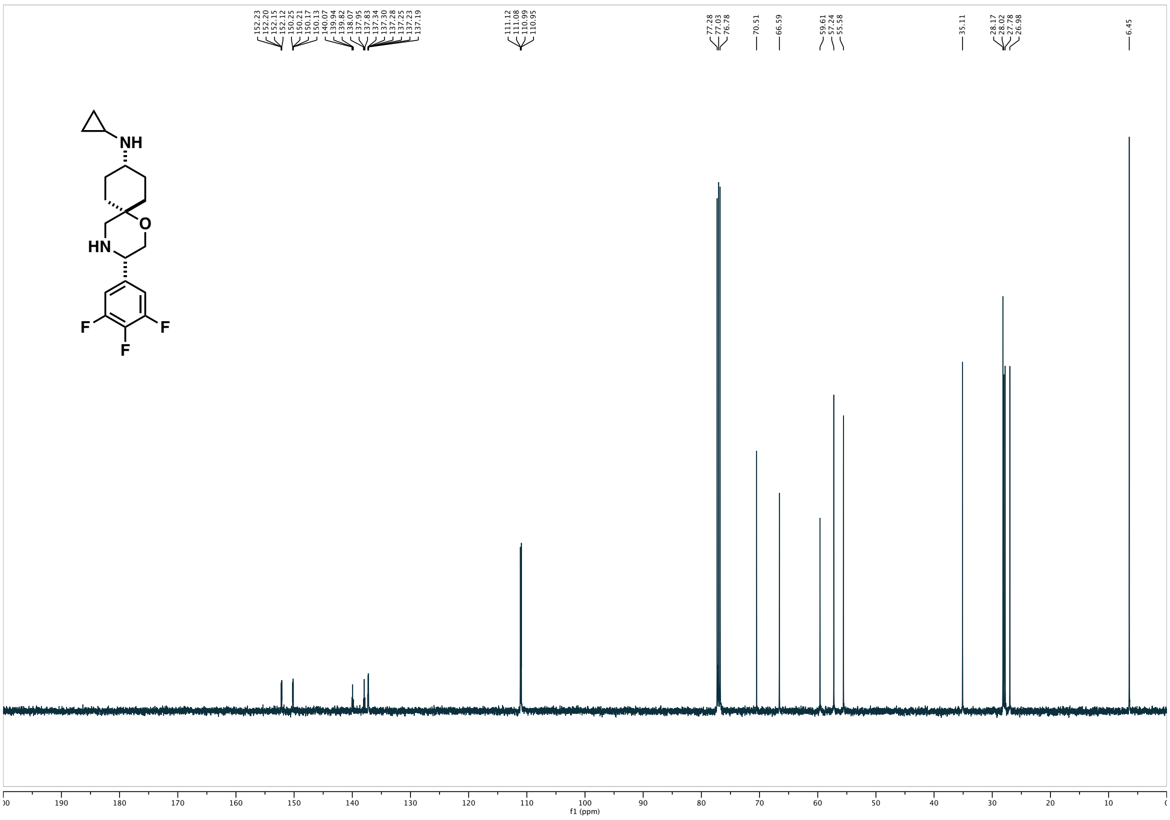

21-DiaB

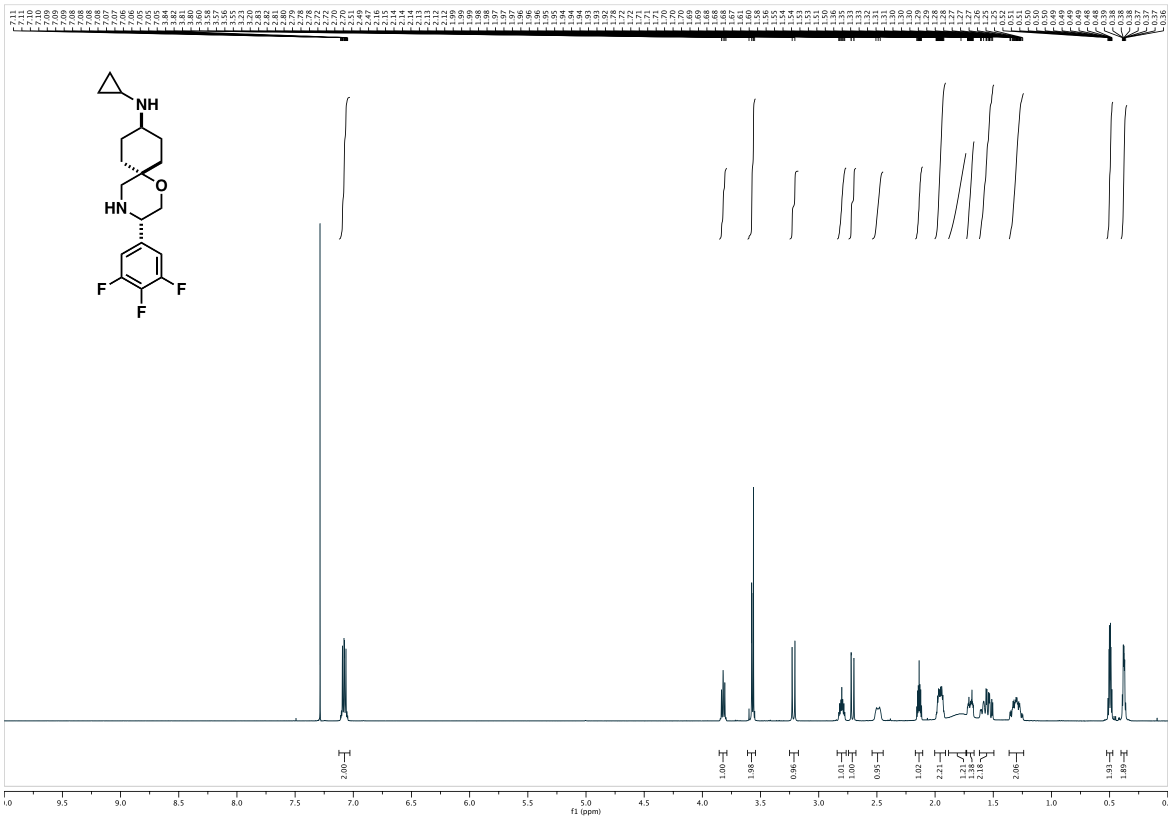
NH
O
HN
F
F
F
f1 (ppm)

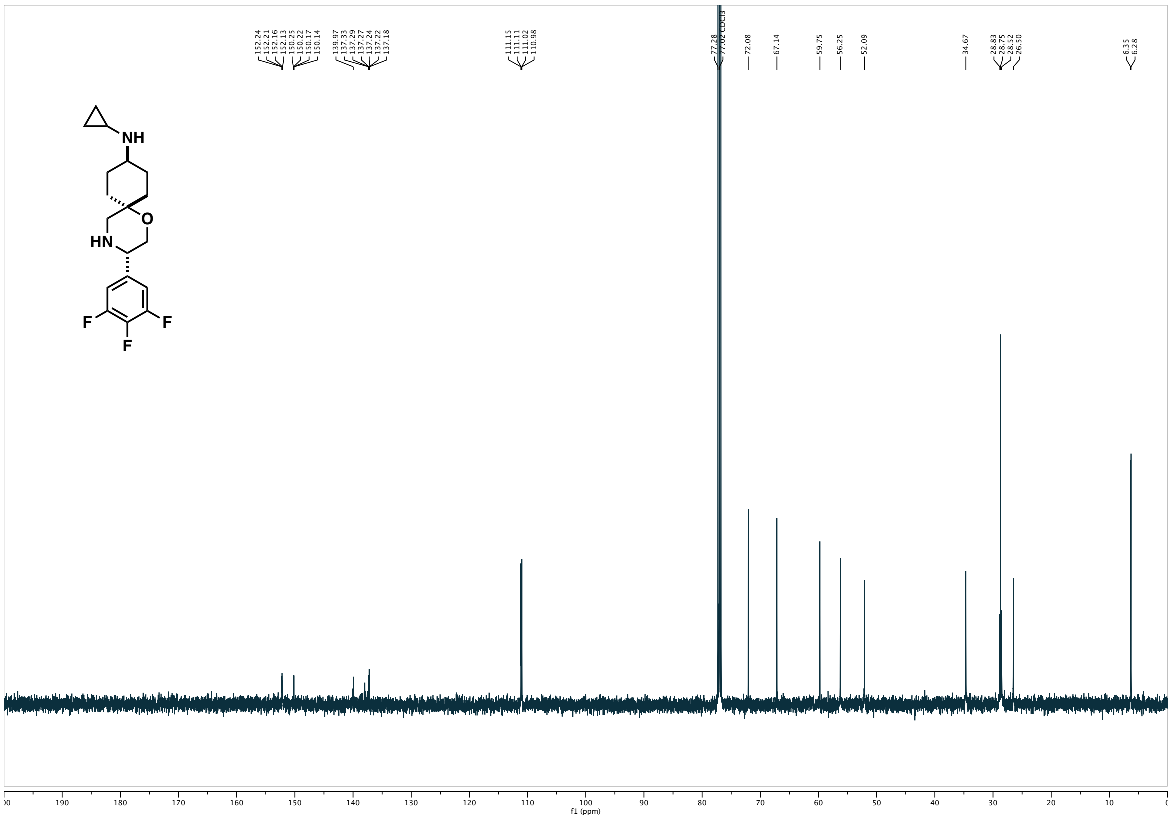
152.24
152.21
152.16
152.13
150.25
150.22
150.17
150.14
139.97
137.33
137.29
137.27
137.24
137.22
137.18
111.15
111.11
111.02
110.98
77.28
77.02 CDCl3
72.08
67.14
59.75
56.25
52.09
34.67
28.83
28.75
28.62
26.50
6.35
6.28
NH
O
HN
F
F
F
f1 (ppm)

22-DiaA

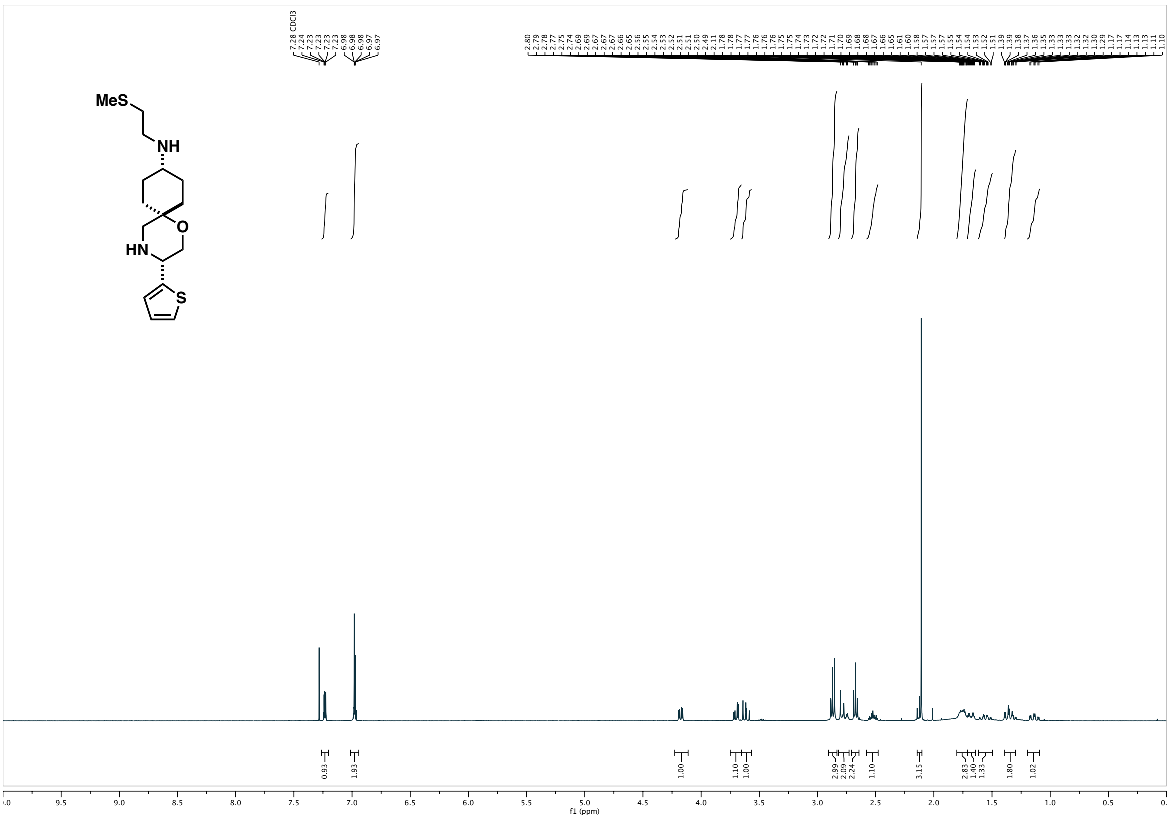
7.28 CDCl3
MeS
NH
O
HN
S
0.93
1.93
1.00
1.10
1.00
2.99
2.09
2.24
1.10
3.15
2.83
1.40
1.33
1.80
1.02
f1 (ppm)

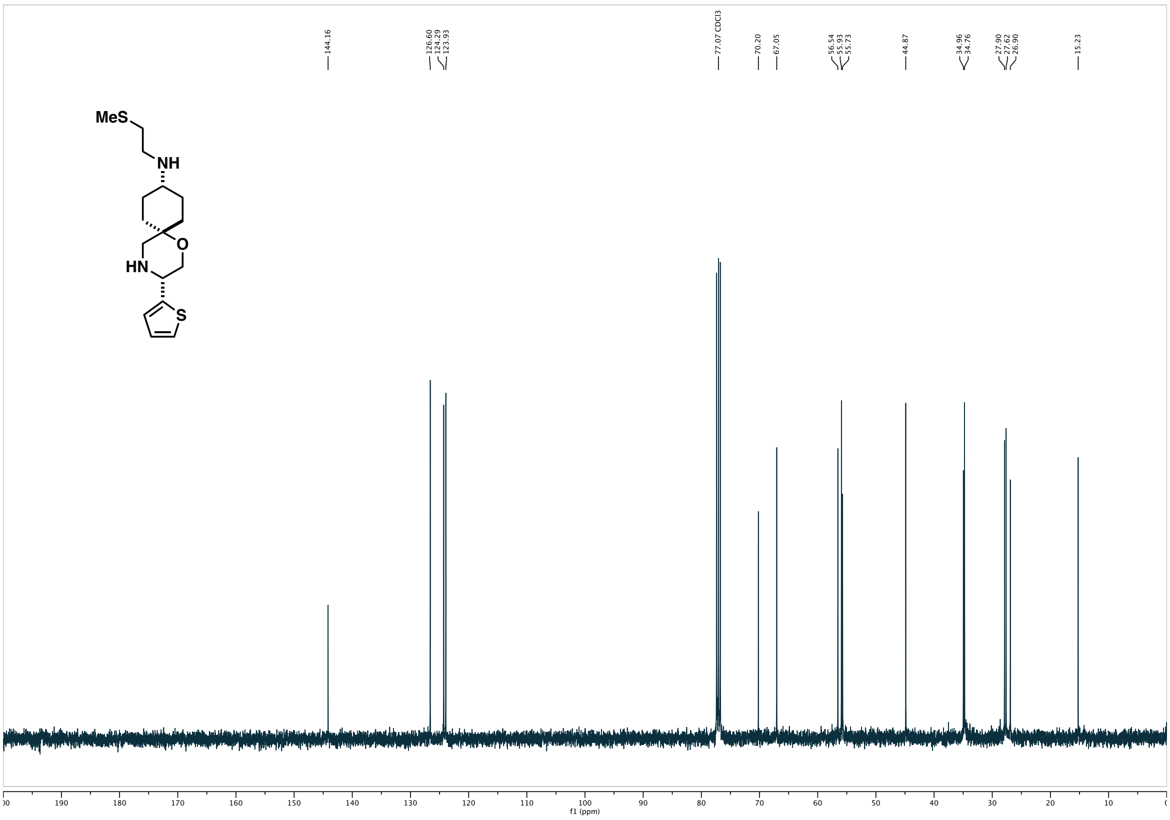
144.16
126.60
124.29
123.93
77.07 CDCl3
70.20
67.05
56.54
55.93
55.73
44.87
34.96
34.76
27.90
27.62
26.90
15.23
MeS
NH
O
HN
S
f1 (ppm)

22-DiaB

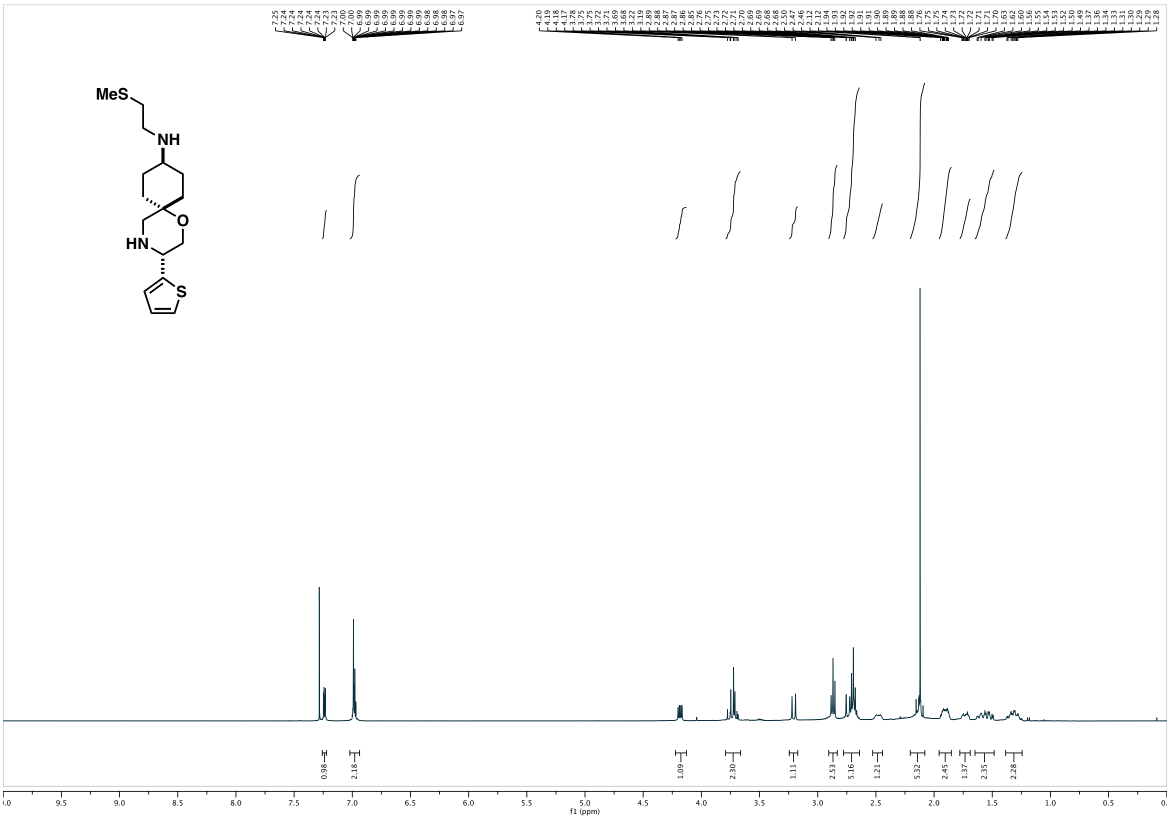

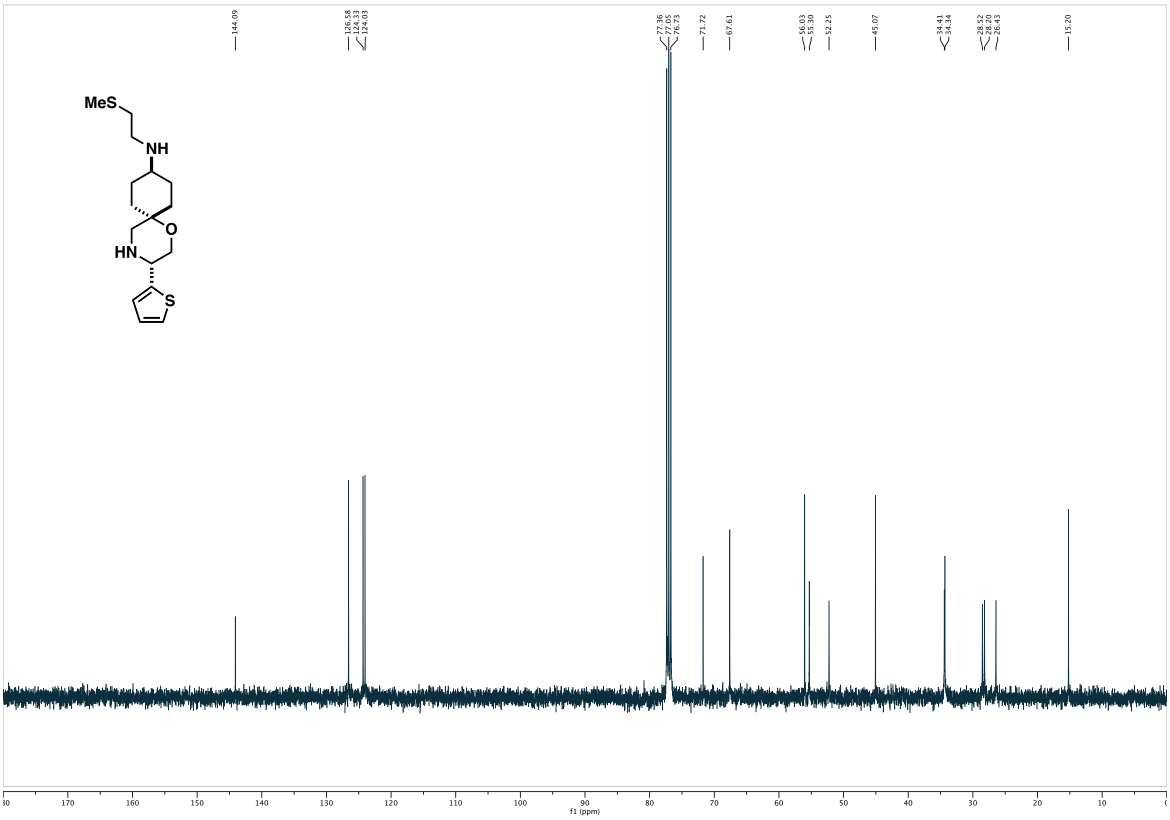

23-DiaA

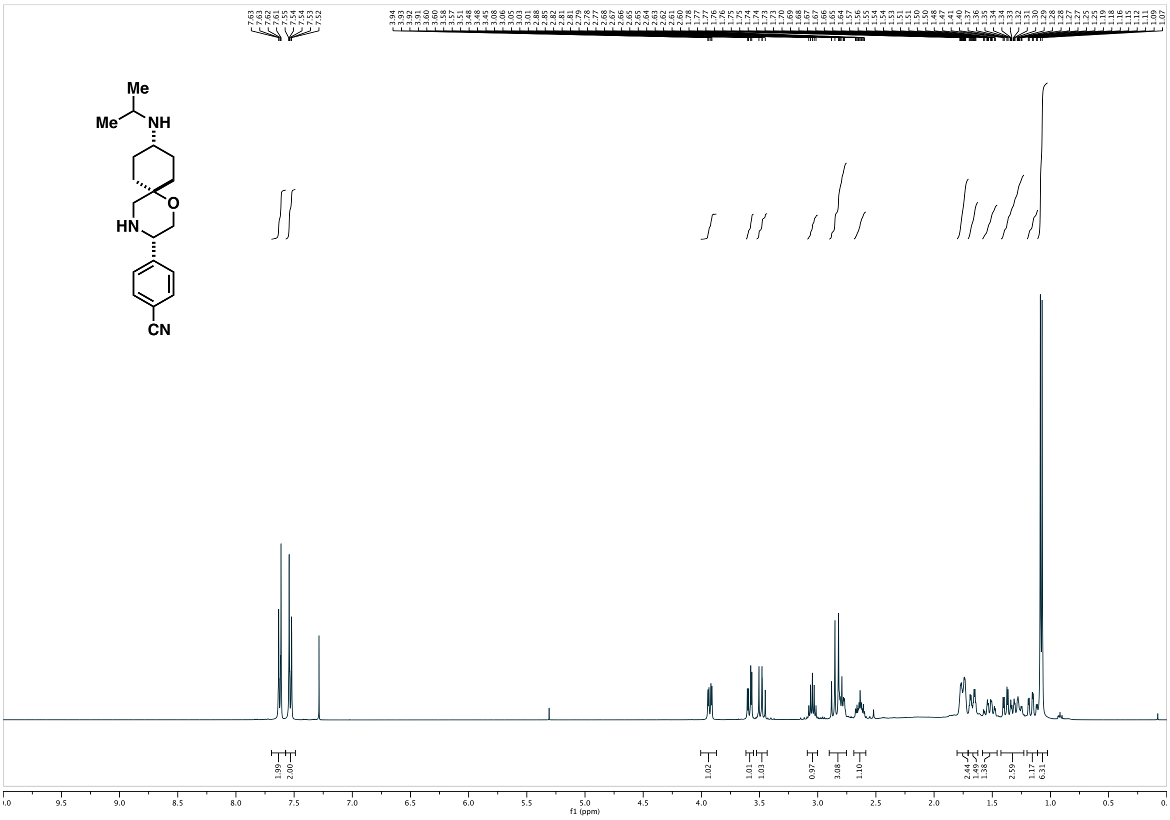

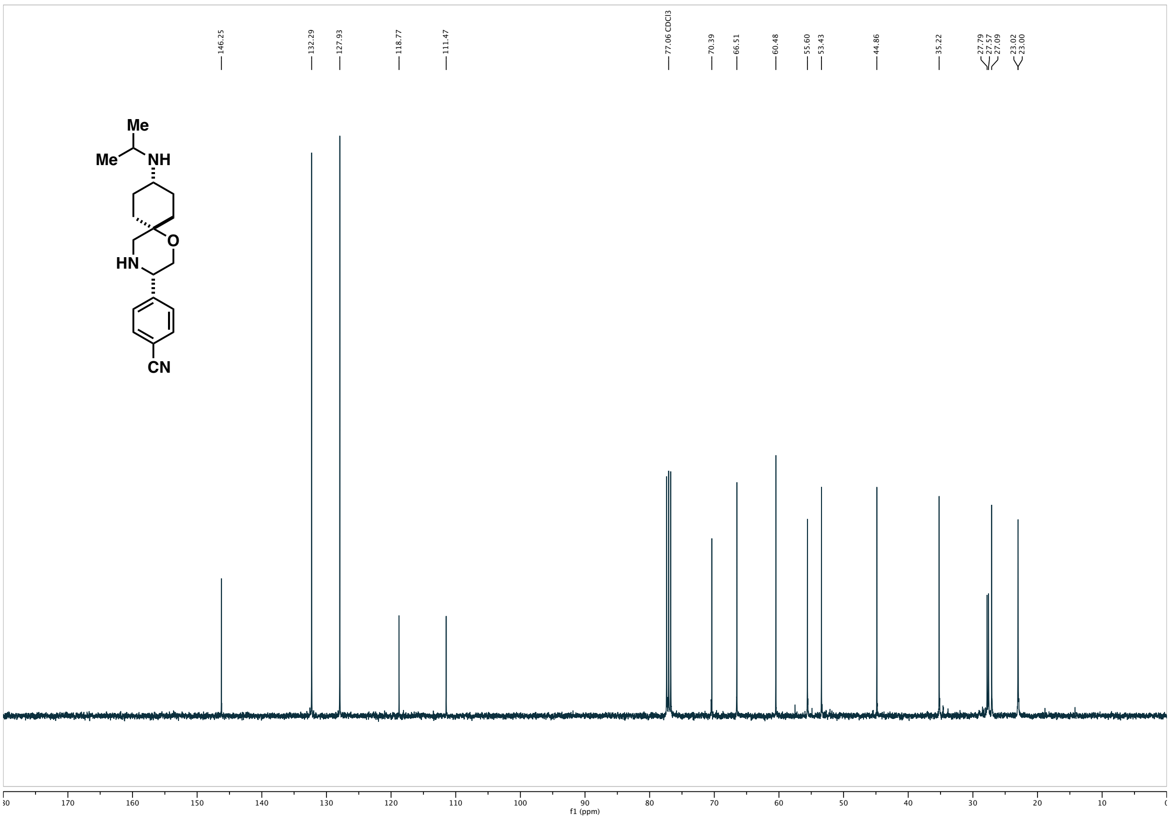

23-DiaB

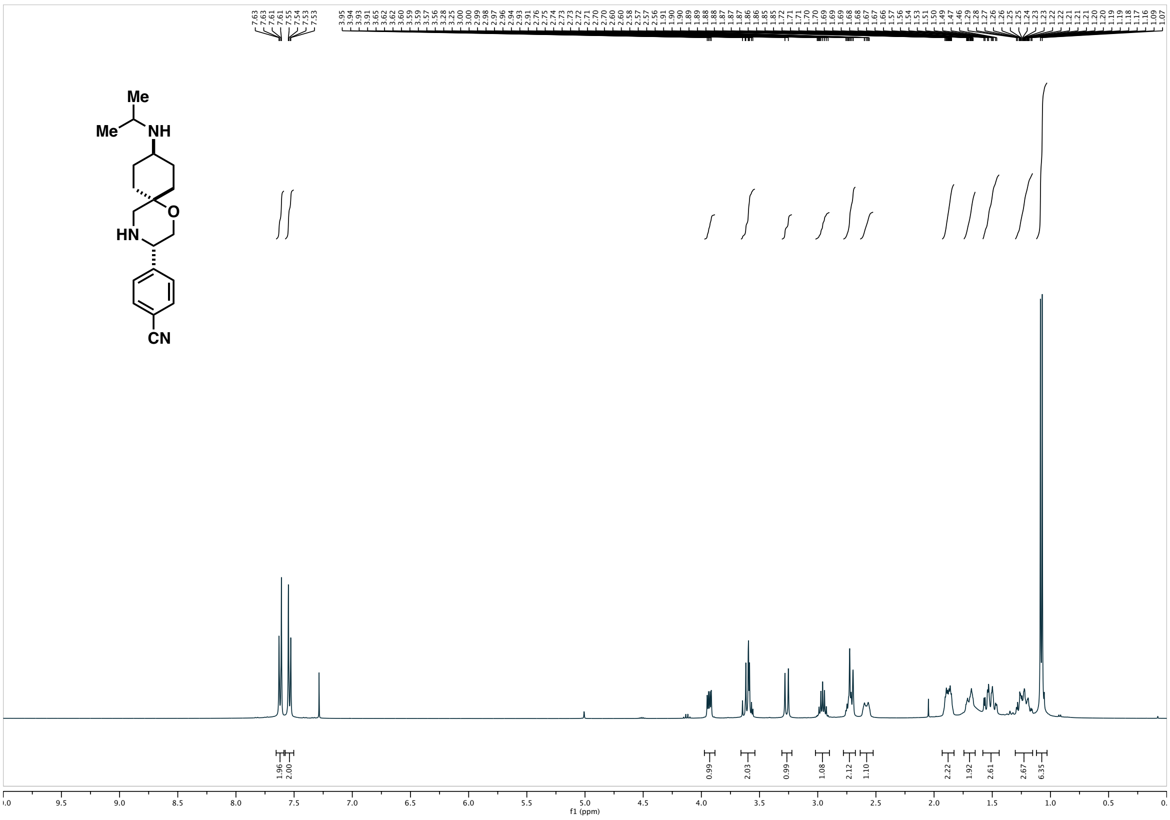

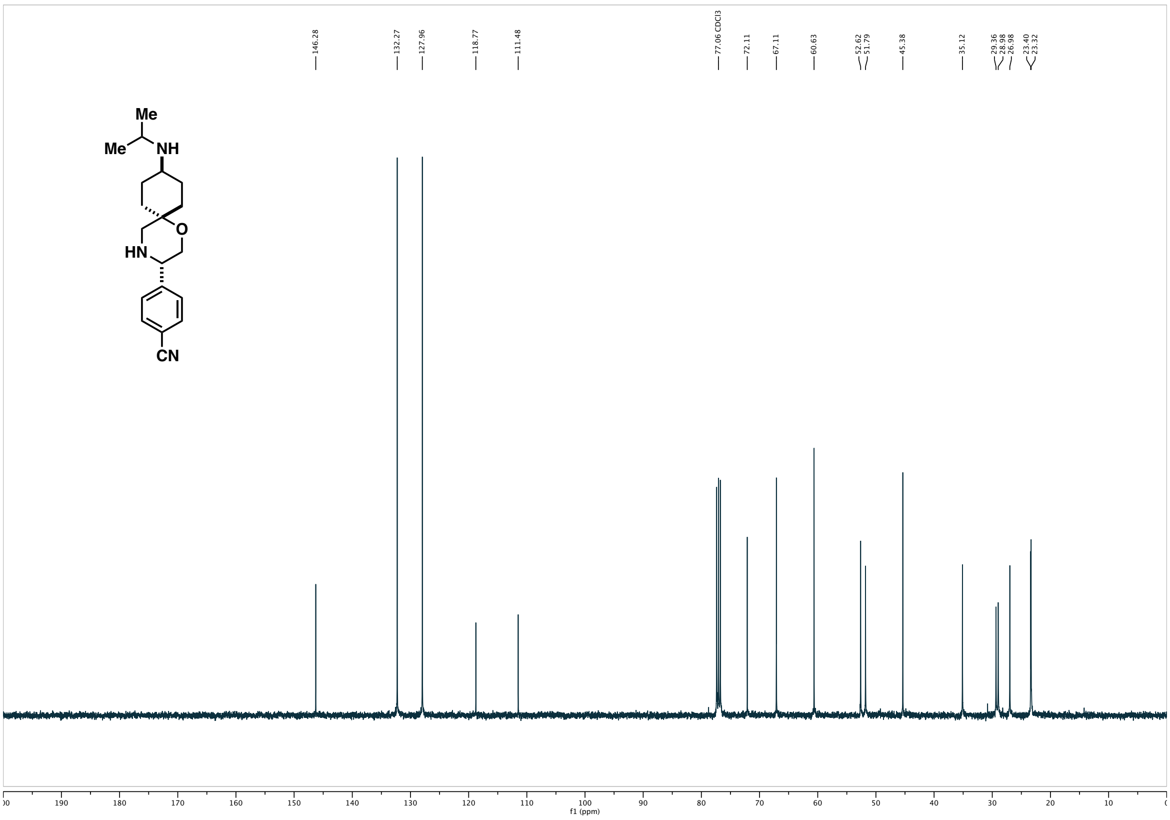

24-DiaA

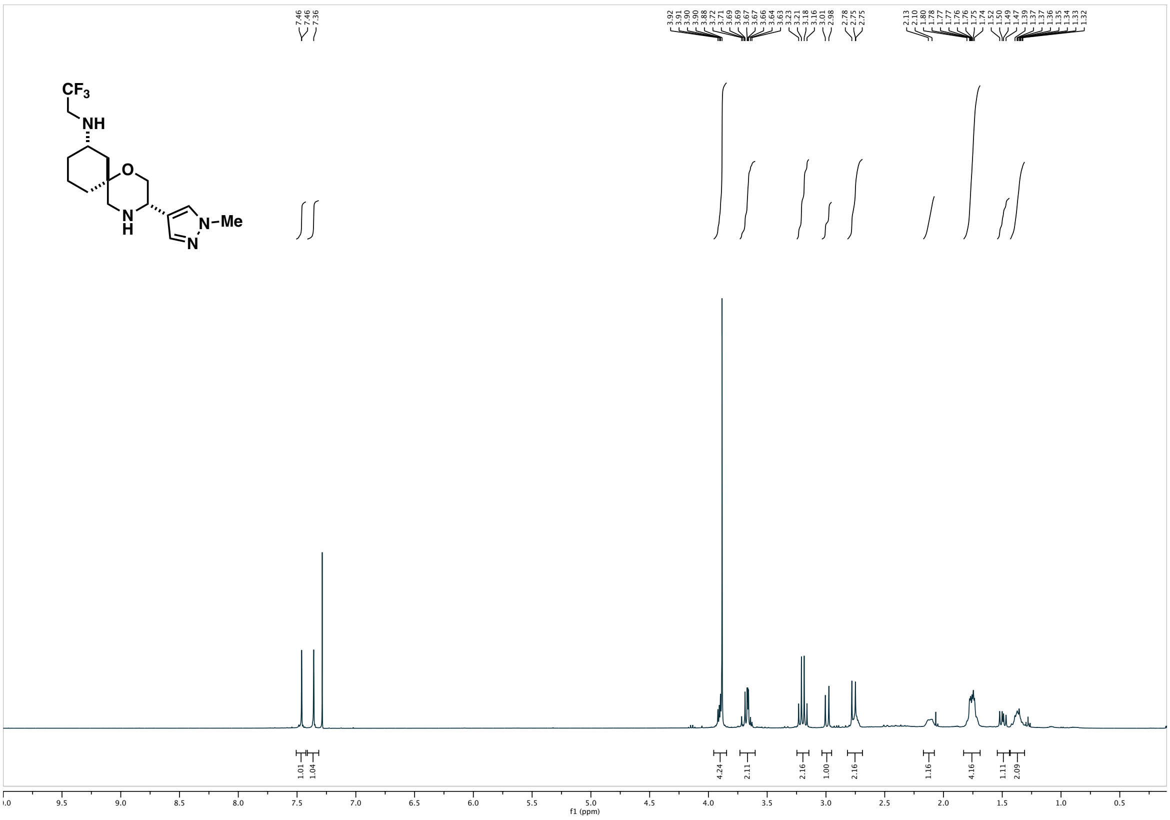

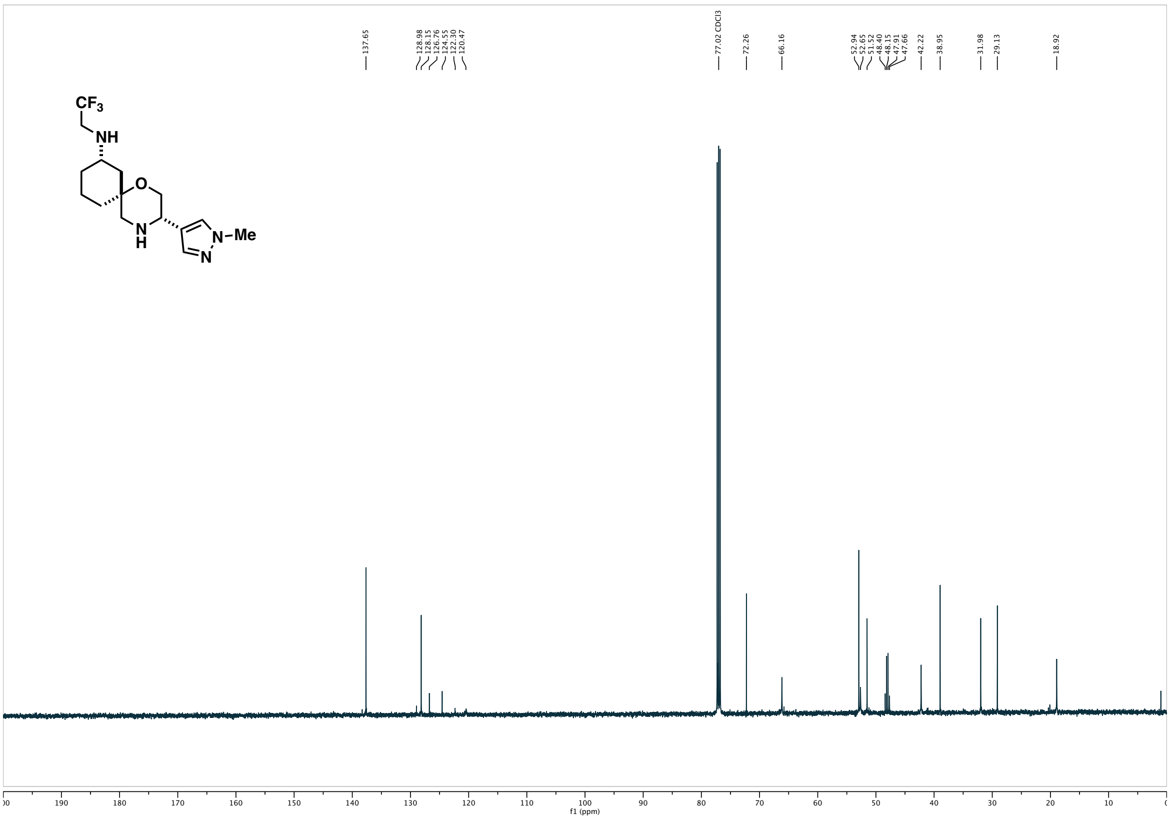

25-DiaA

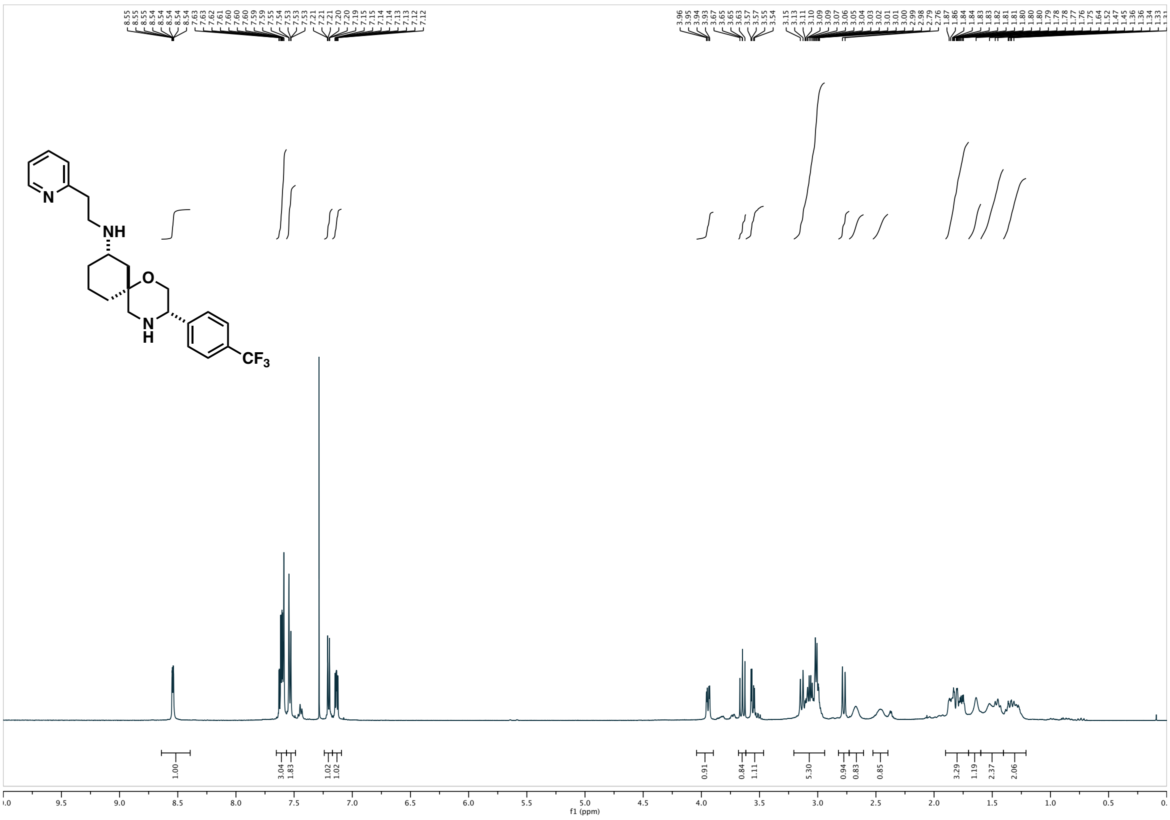
NH
O
N
H
CF3
f1 (ppm)

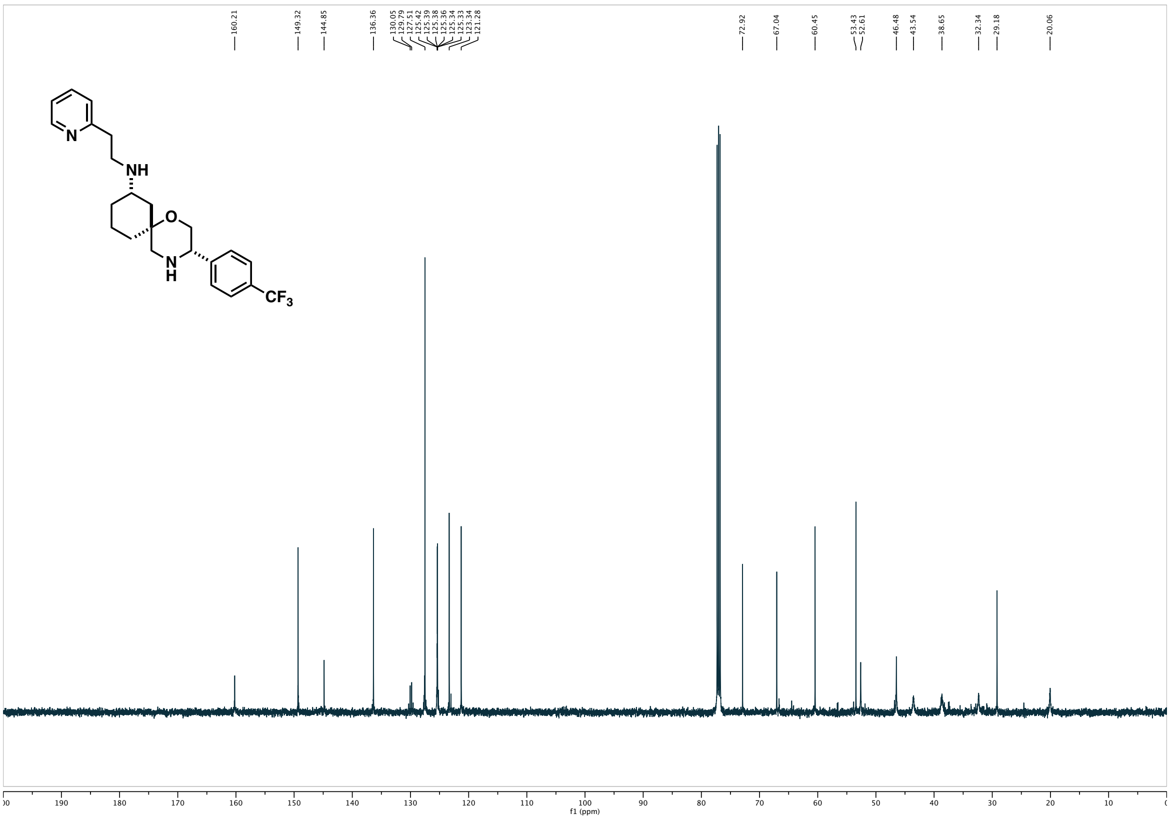
160.21
149.32
144.85
136.36
130.05
129.79
127.51
125.42
125.39
125.38
125.36
125.34
125.33
123.34
121.28
72.92
67.04
60.45
53.43
52.61
46.48
43.54
38.65
32.34
29.18
20.06
NH
O
N
H
CF3
f1 (ppm)

26-DiaD

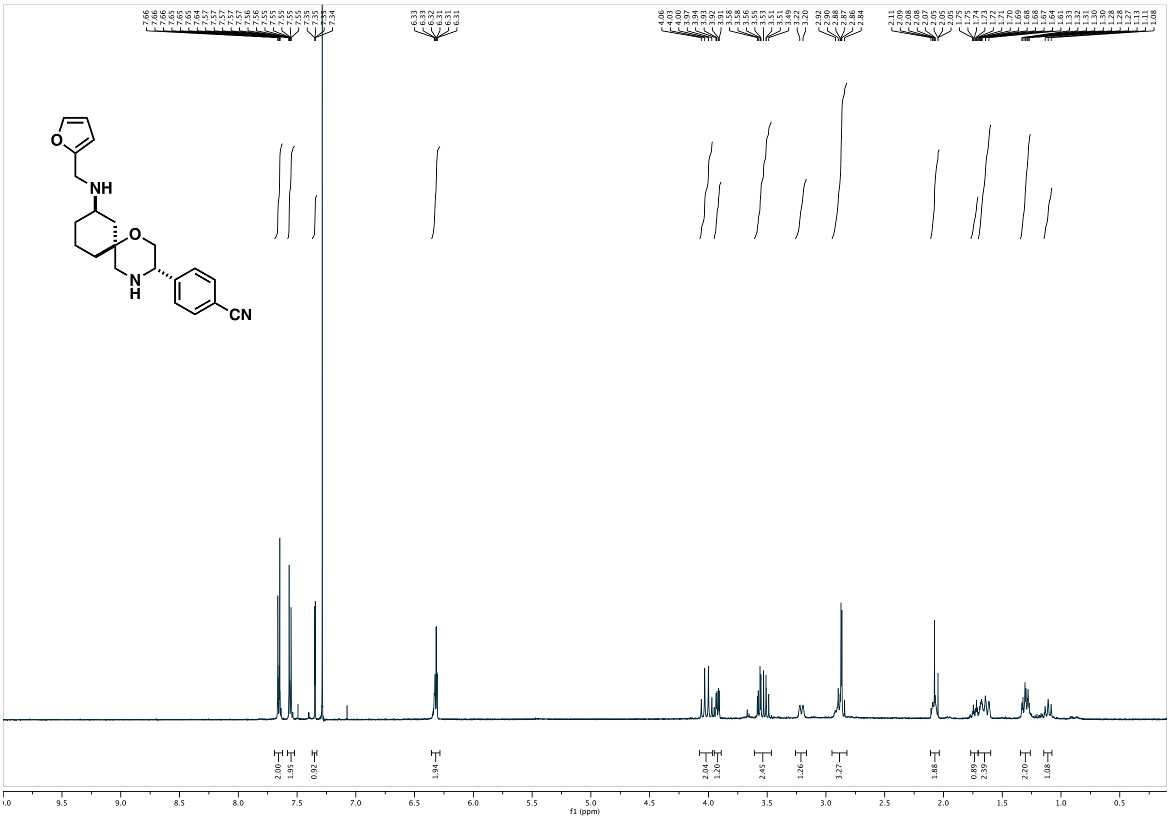
O
NH
O
N
H
CN
f1 (ppm)

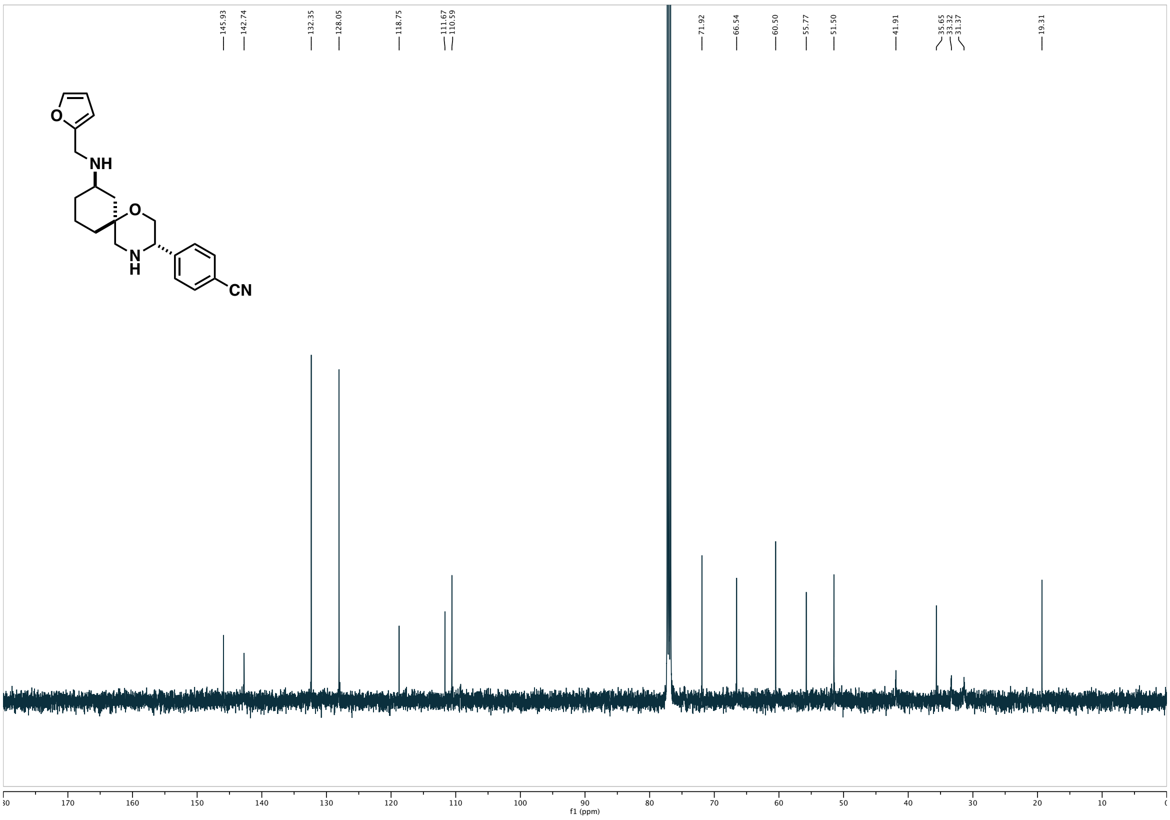
145.93
142.74
132.35
128.05
118.75
111.67
110.59
71.92
66.54
60.50
55.77
51.50
41.91
35.65
33.32
31.37
19.31
O
NH
O
N
H
CN
f1 (ppm)

27-DiaA

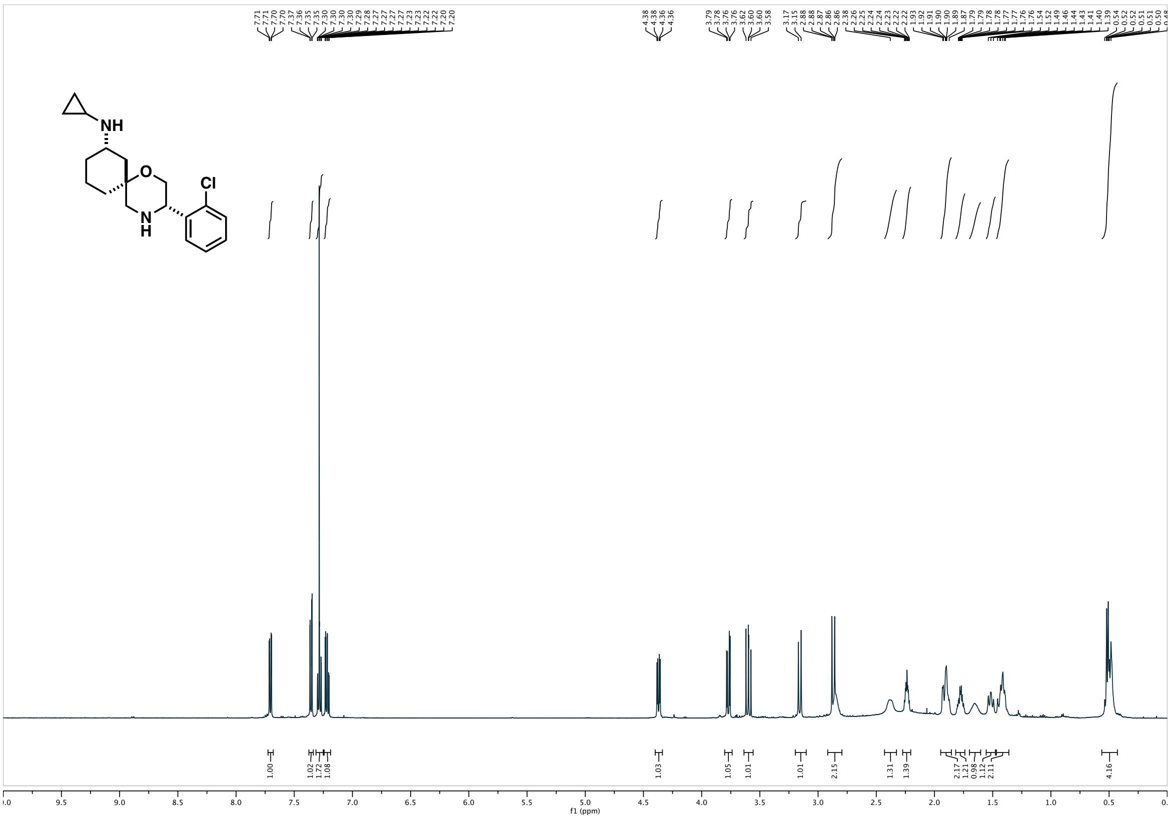
NH
O
Cl
N
H
f1 (ppm)

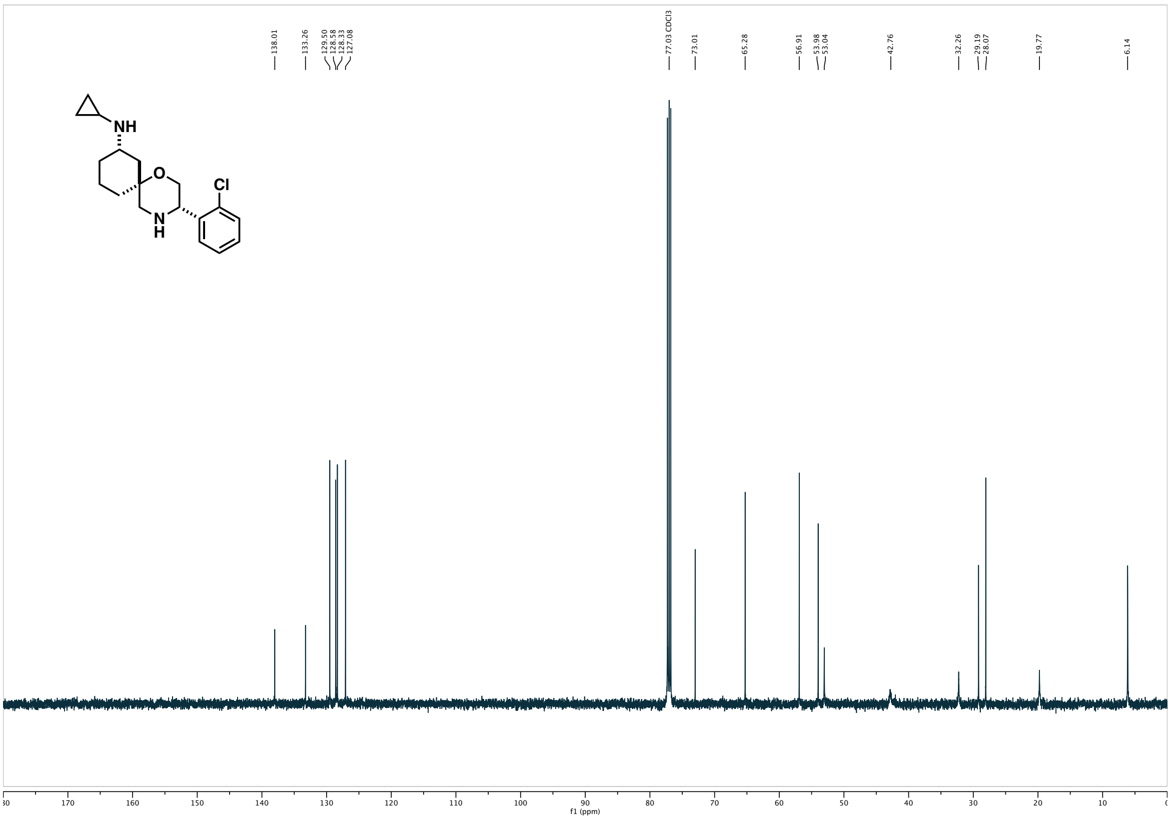
138.01
133.26
129.50
128.58
128.33
127.08
77.03 CDCl3
73.01
65.28
56.91
53.98
53.04
42.76
32.26
29.19
28.07
19.77
6.14
NH
O
Cl
N
H
f1 (ppm)

27-DiaB

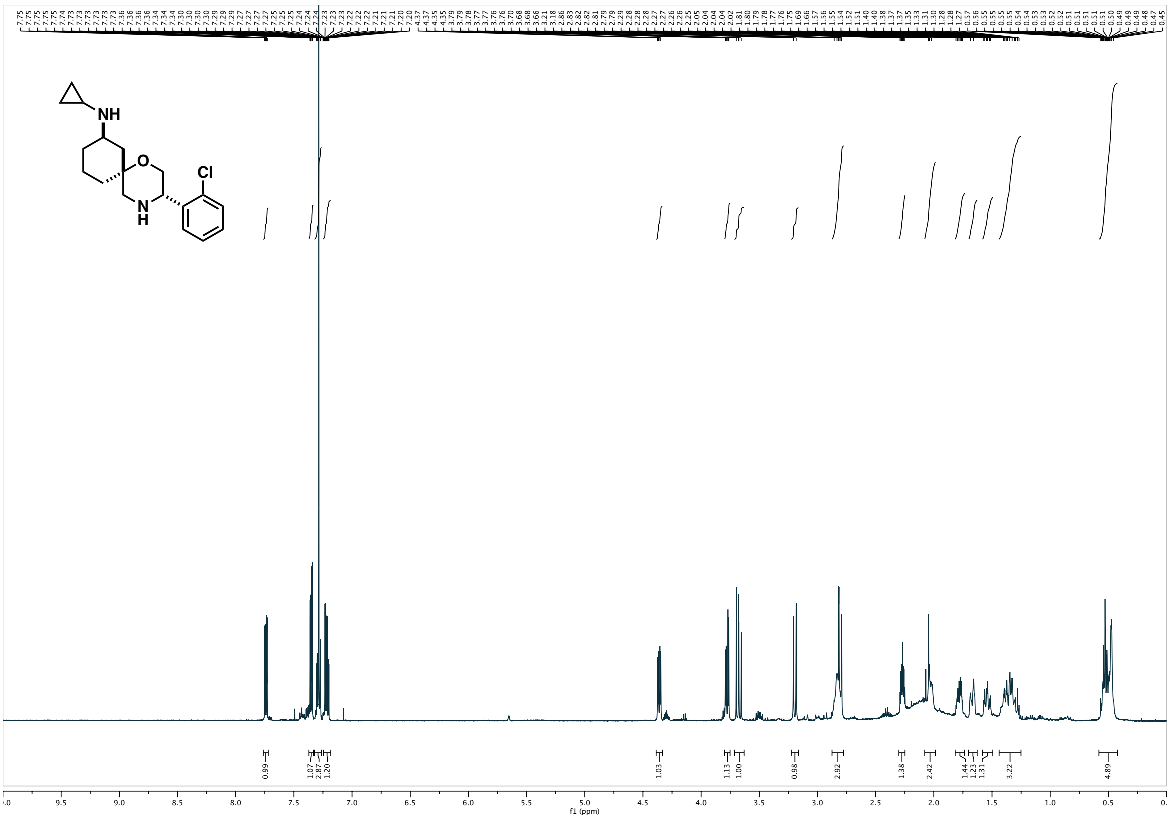
NH
O
Cl
N
H
0.99
1.07
2.87
1.20
1.03
1.13
1.00
0.98
2.92
1.38
2.42
1.44
1.23
1.31
3.22
4.89
f1 (ppm)

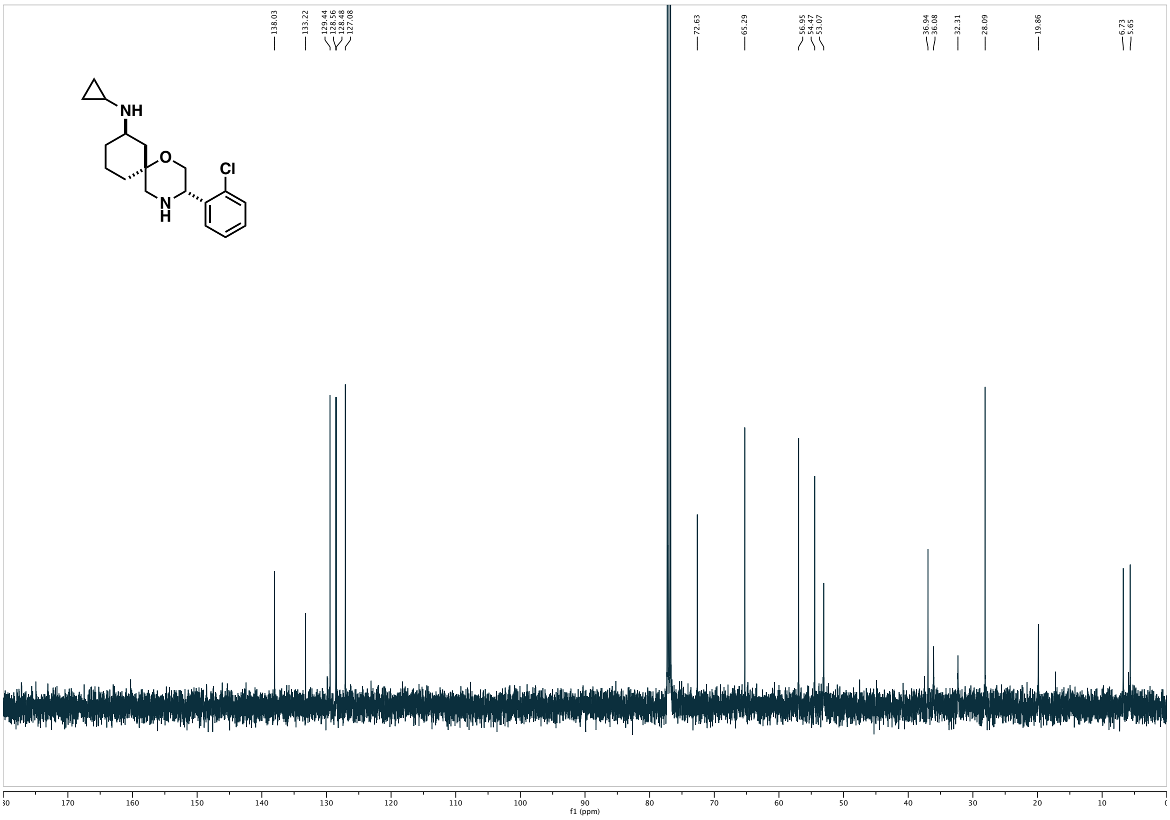
138.03
133.22
129.44
128.56
128.48
127.08
72.63
65.29
56.95
54.47
53.07
36.94
36.08
32.31
28.09
19.86
6.73
5.65
NH
O
Cl
N
H
f1 (ppm)

27-DiaC

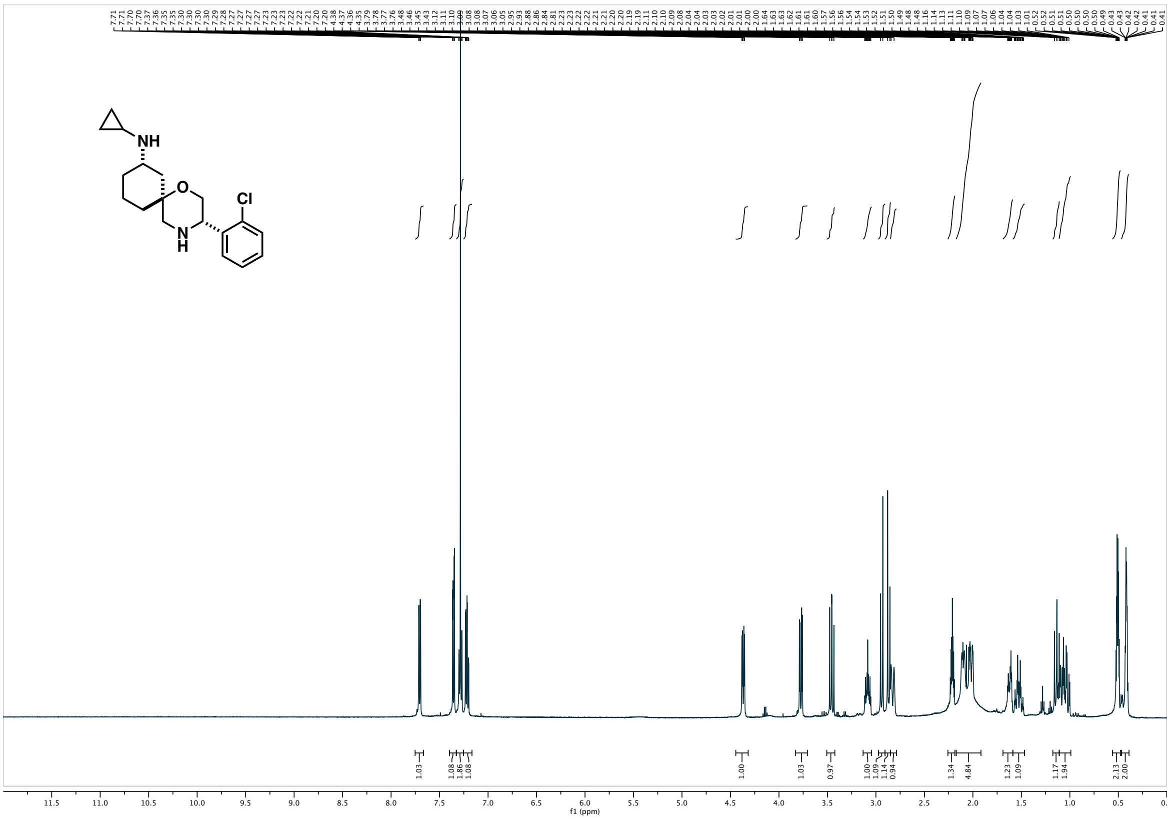

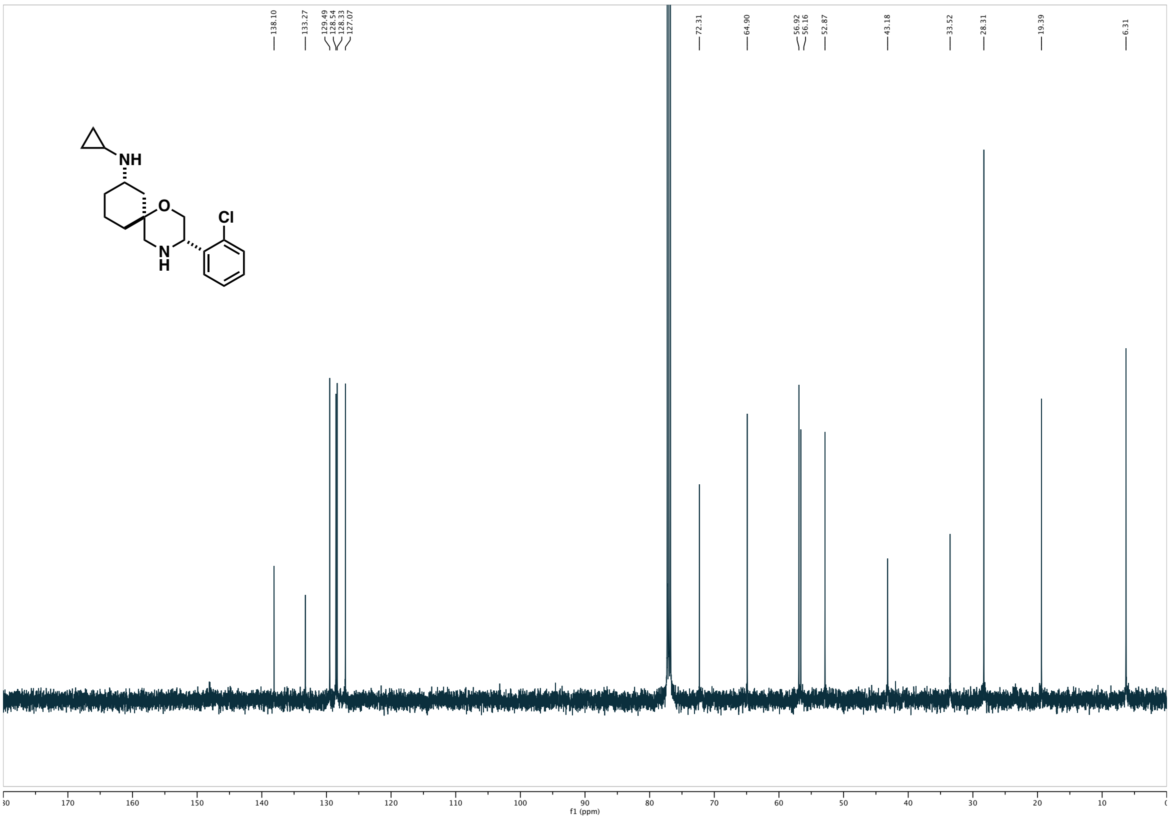

27-DiaD

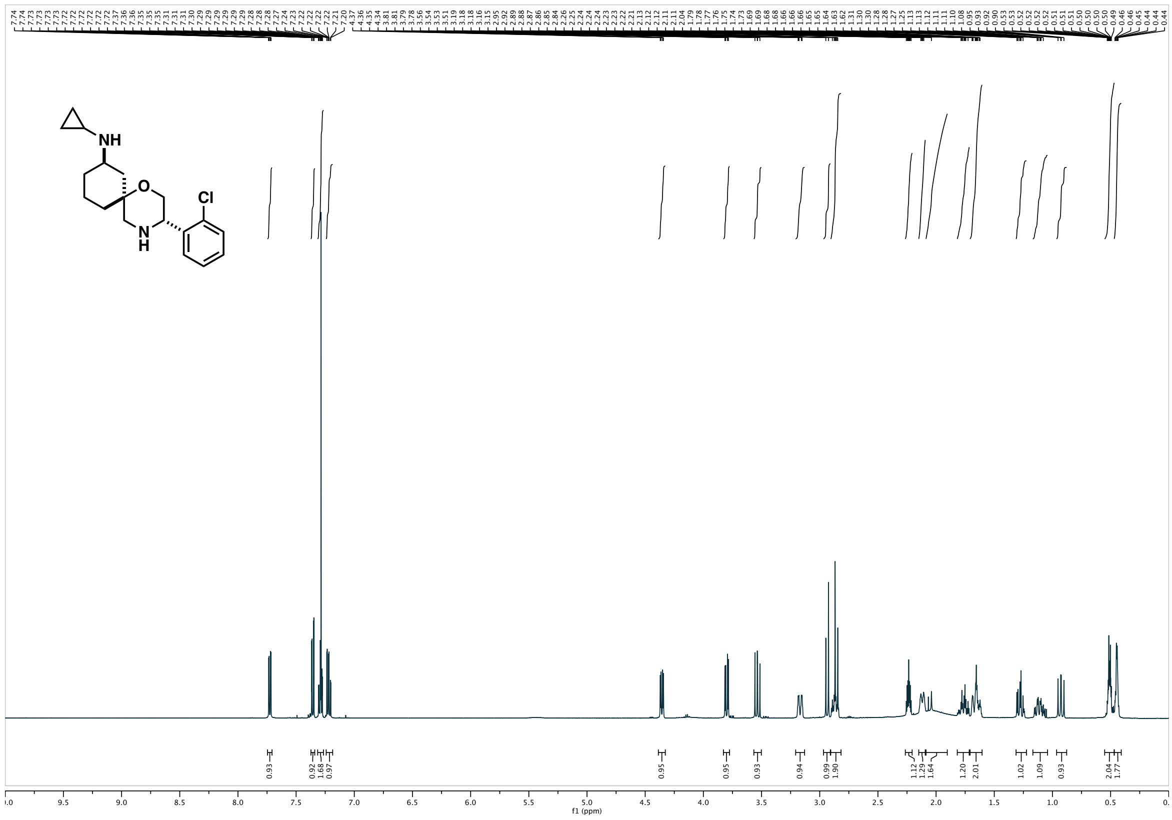
NH
O
Cl
N
H
f1 (ppm)

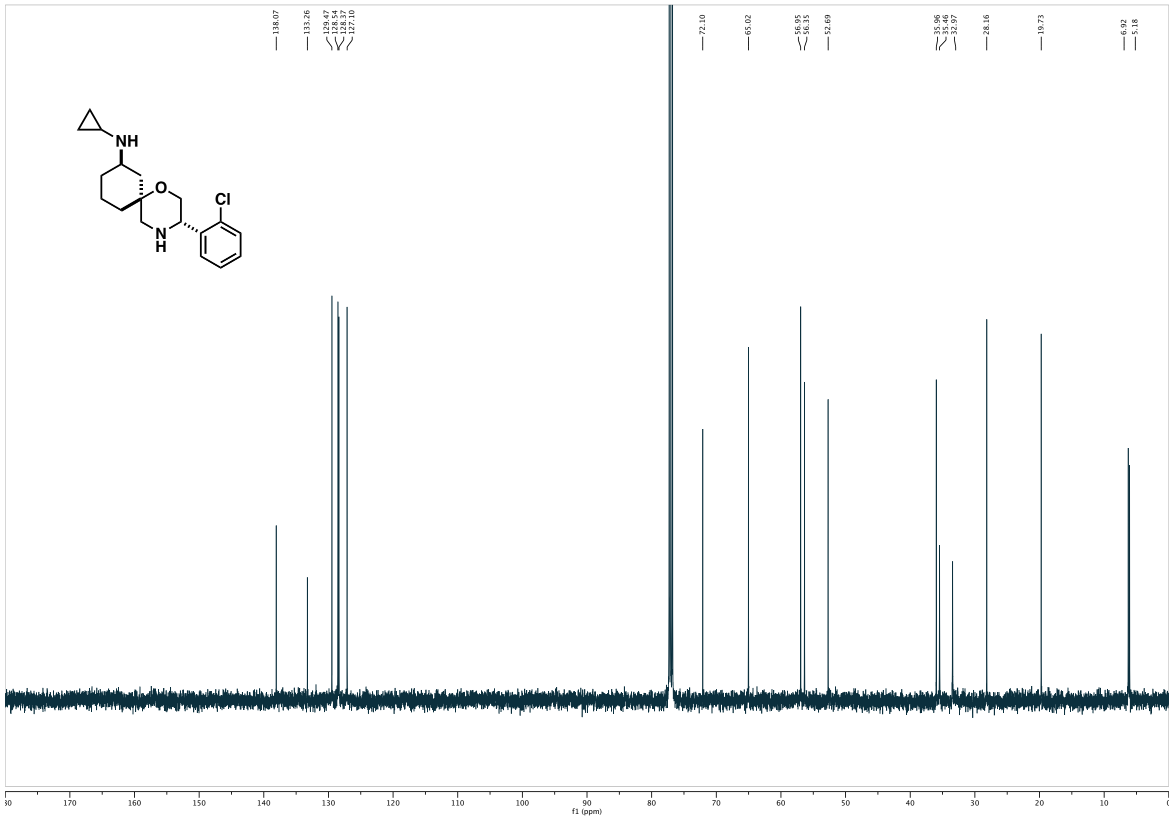
138.07
133.26
129.47
128.54
128.37
127.10
72.10
65.02
56.95
56.35
52.69
35.96
35.46
32.97
28.16
19.73
6.92
5.18
NH
O
Cl
N
H
f1 (ppm)

28

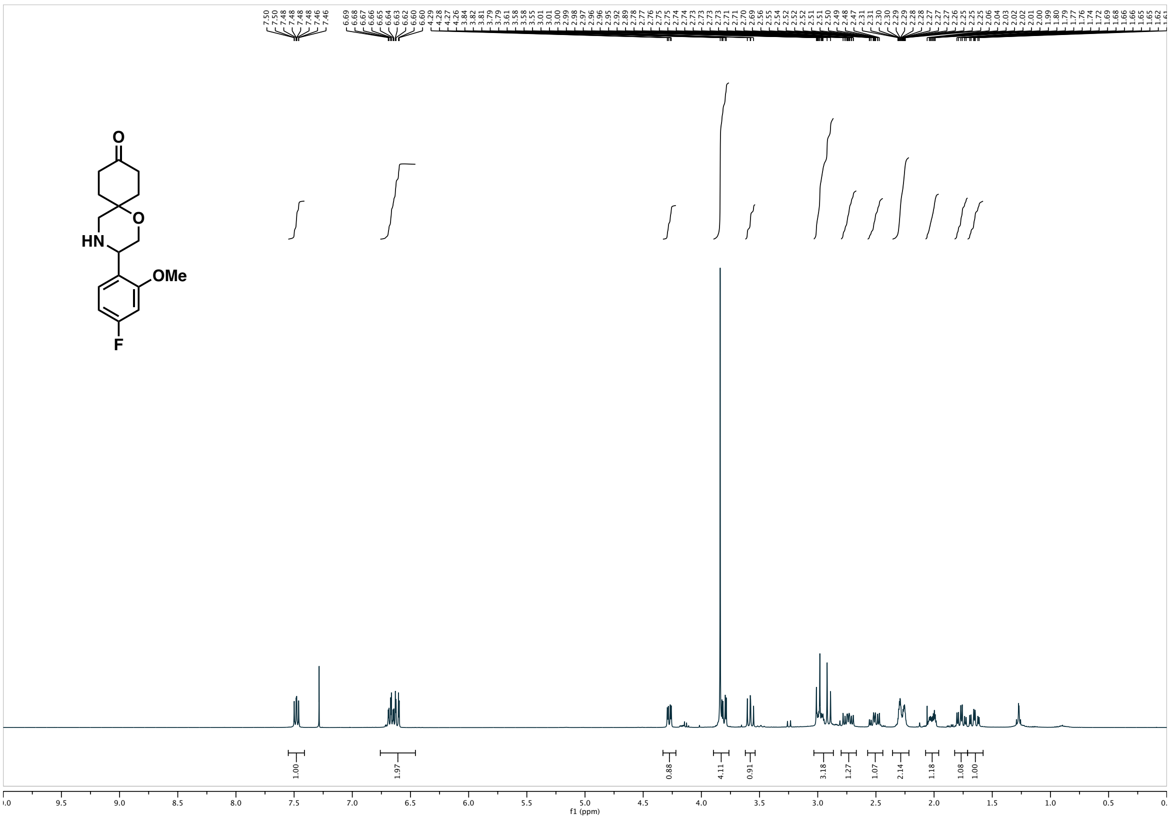

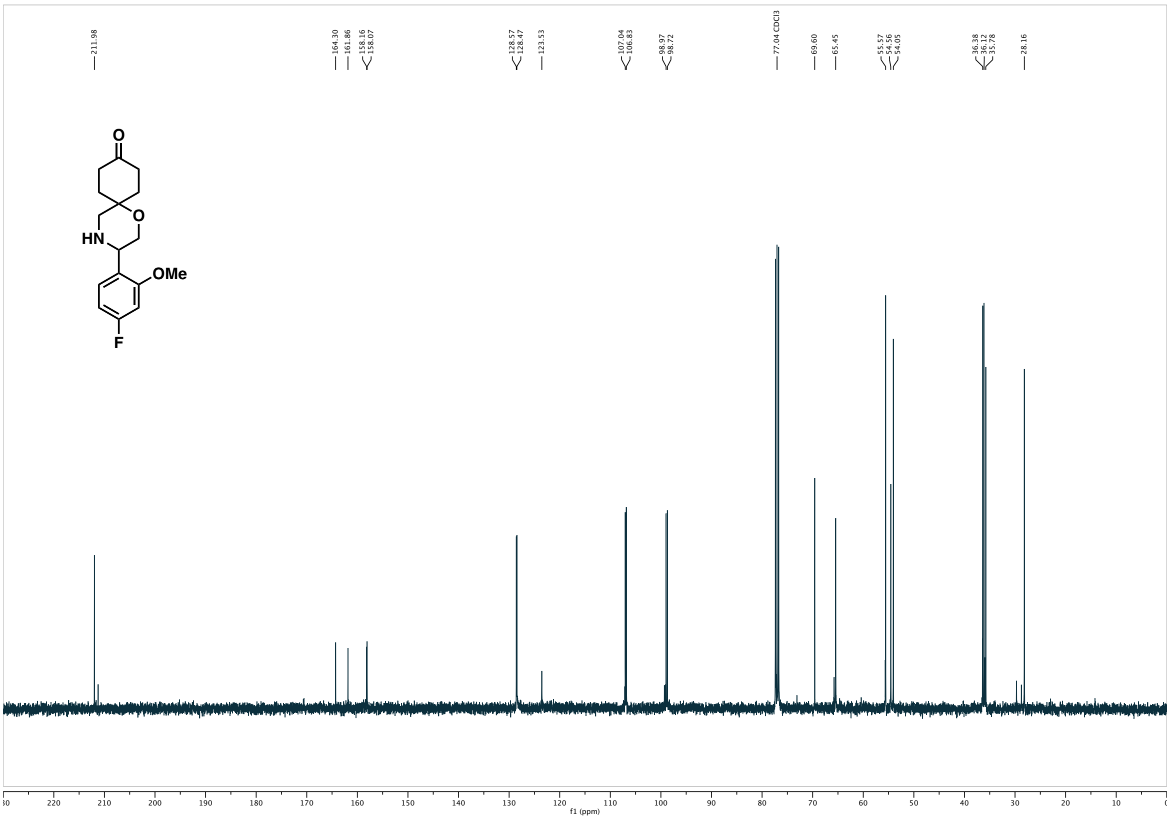

29

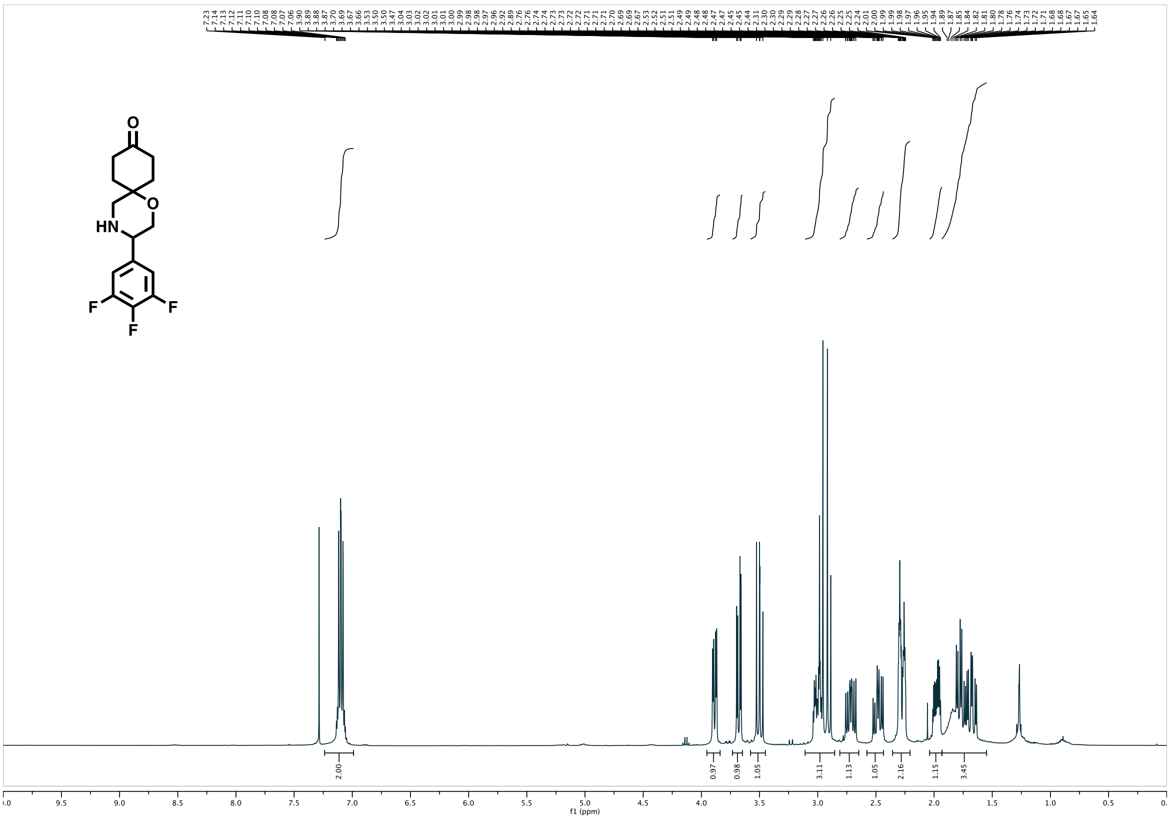
O
HN
O
F
F
F
f1 (ppm)

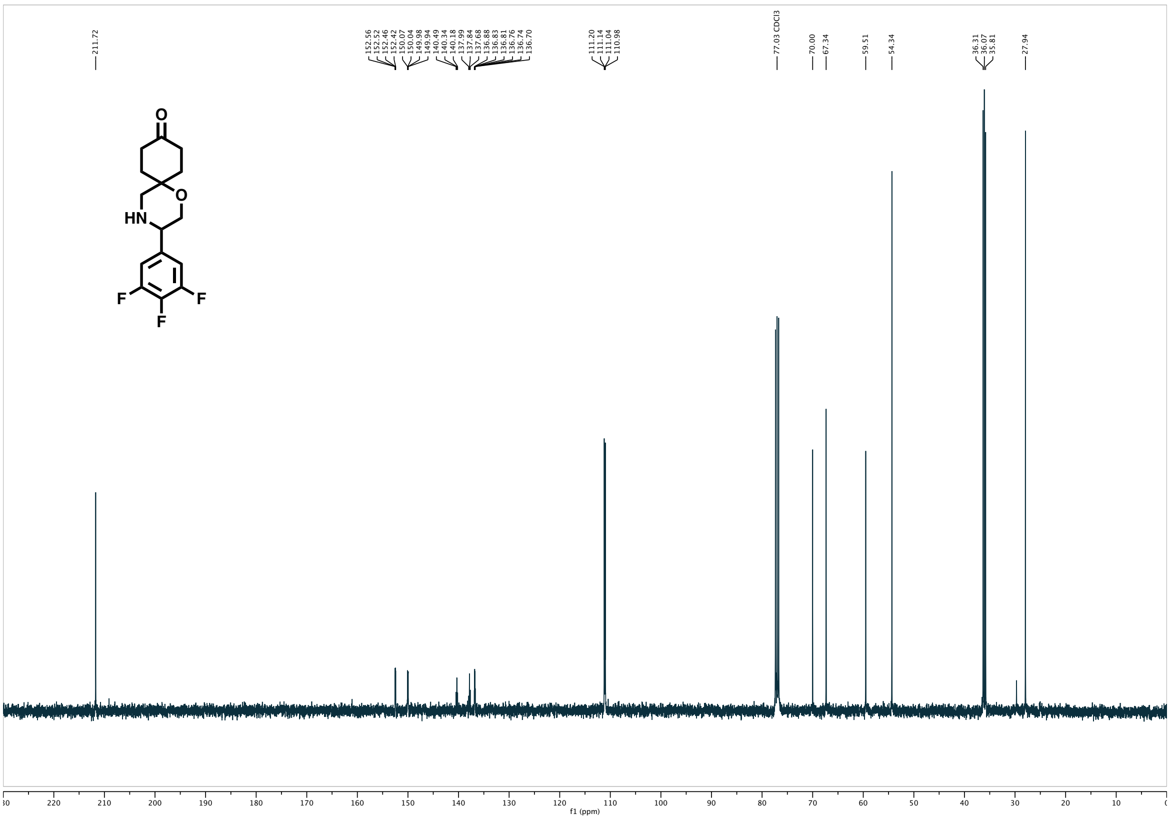
211.72
77.03 CDCl3
70.00
67.34
59.51
54.34
27.94
O
HN
O
F
F
F
f1 (ppm)

30

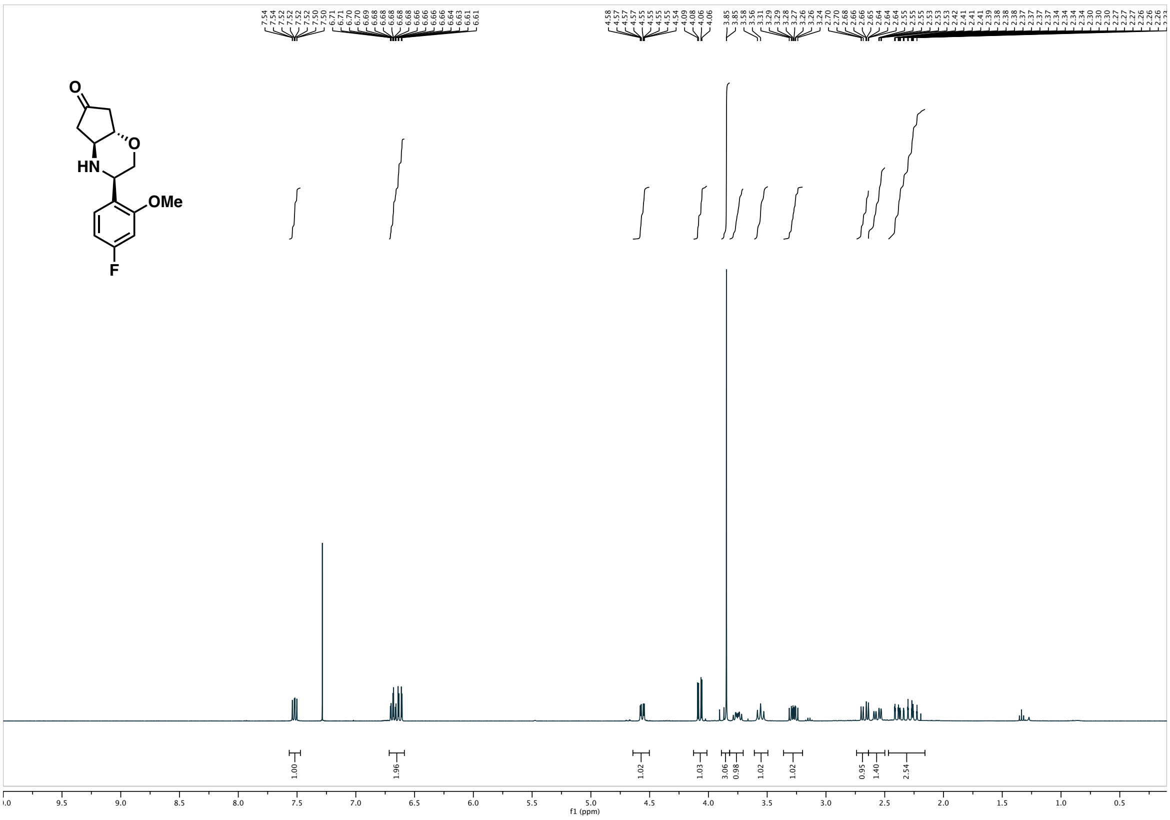
O
HN
O
OMe
F
1.00
1.96
1.02
1.03
3.06
0.98
1.02
1.02
0.95
1.40
2.54
f1 (ppm)

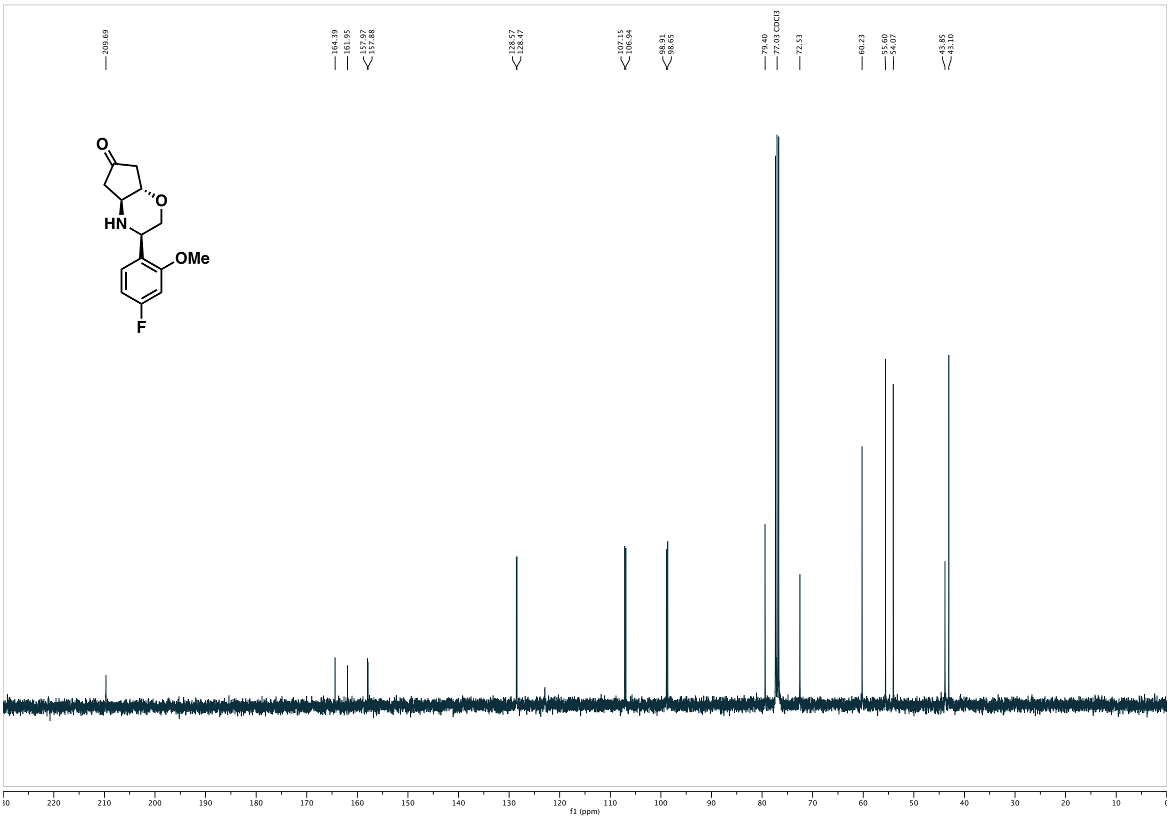
209.69
164.39
161.95
157.97
157.88
128.57
128.47
107.15
106.94
98.91
98.65
79.40
77.03 CDCl3
72.53
60.23
55.60
54.07
43.85
43.10
O
HN
O
OMe
F
f1 (ppm)

31

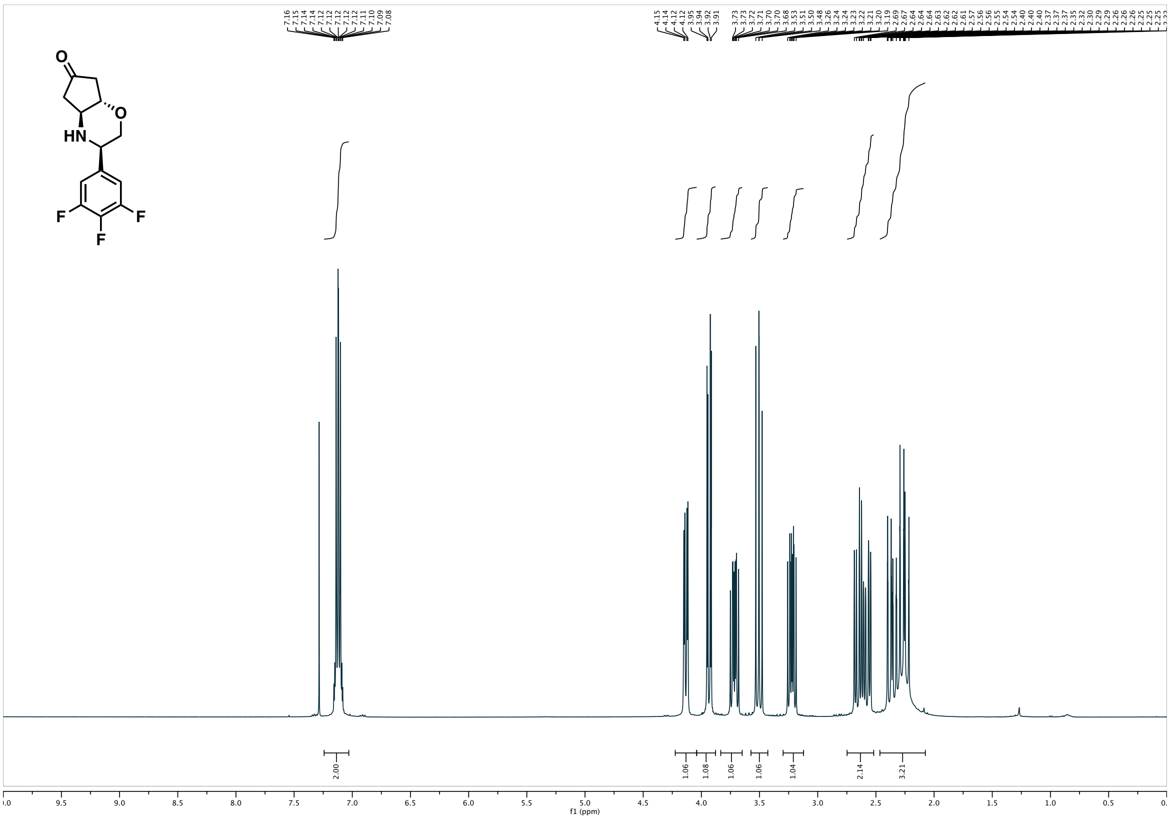

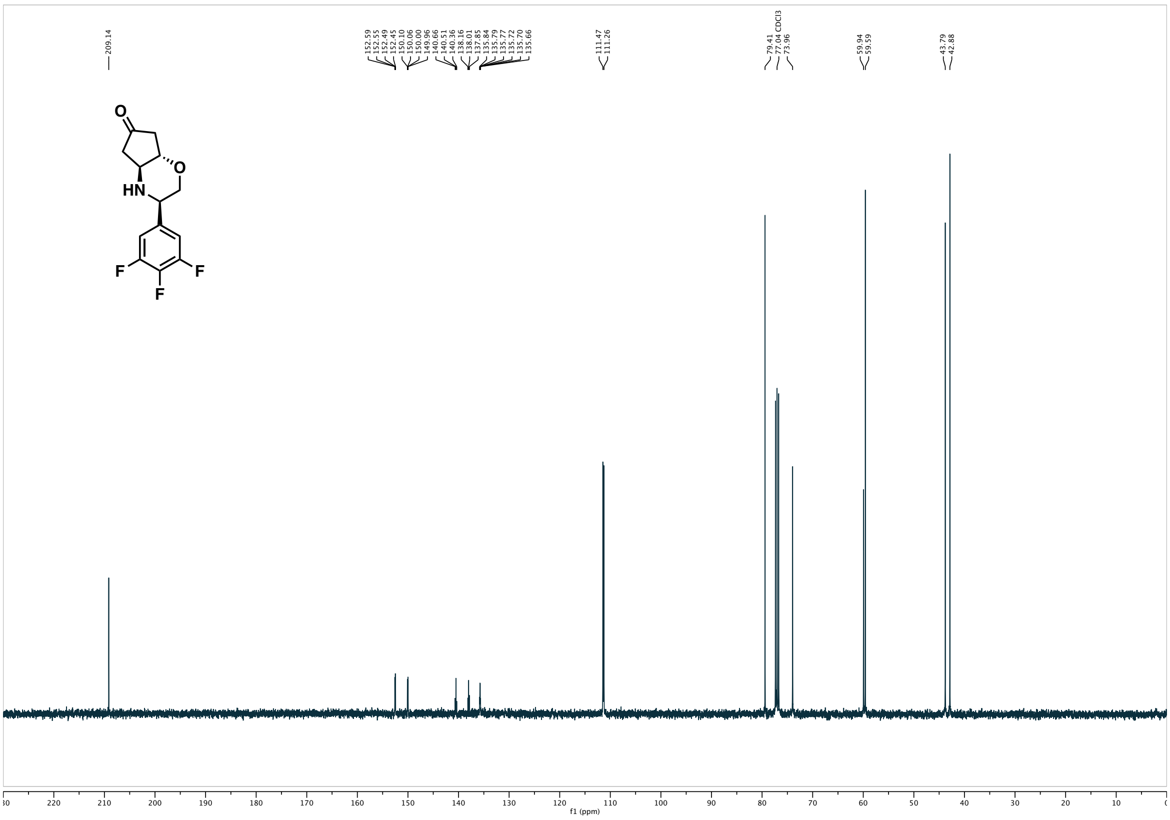

32

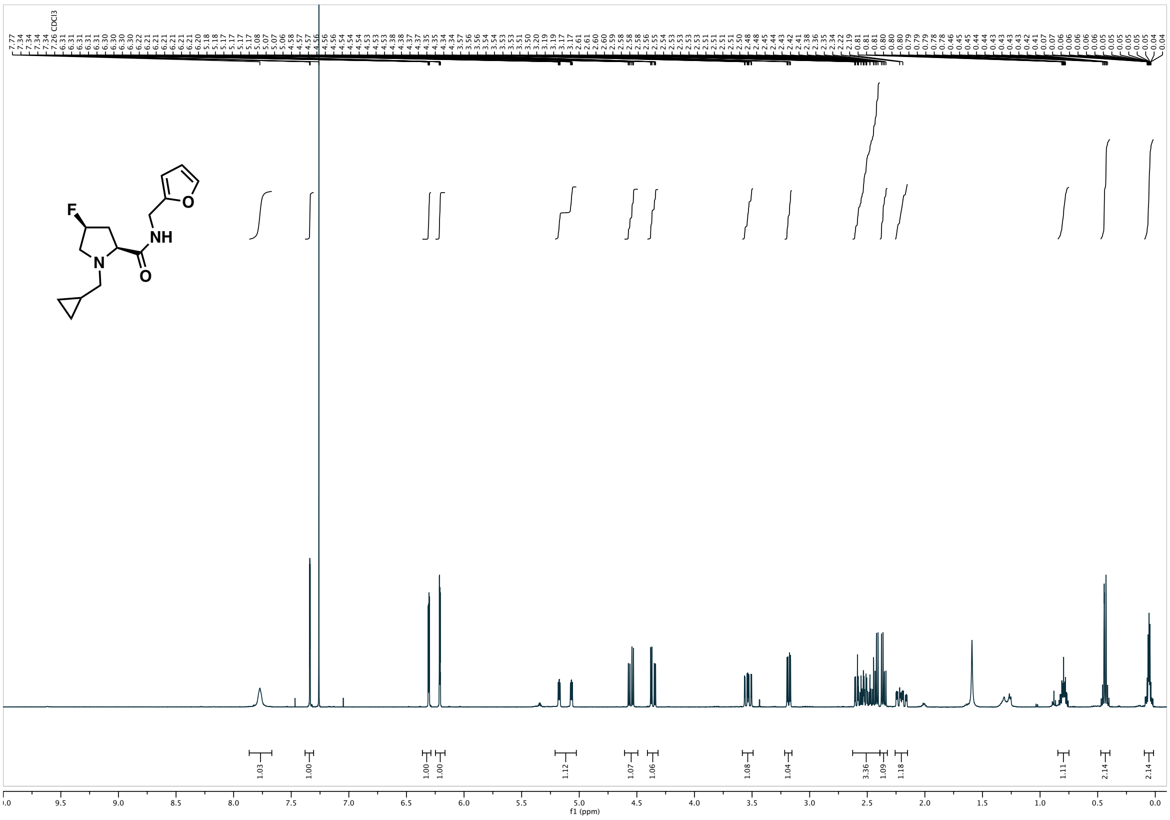
F
NH
N
O
O
1.03
1.00
1.00
1.00
1.12
1.07
1.06
1.08
1.04
3.36
1.09
1.18
1.11
2.14
2.14
f1 (ppm)

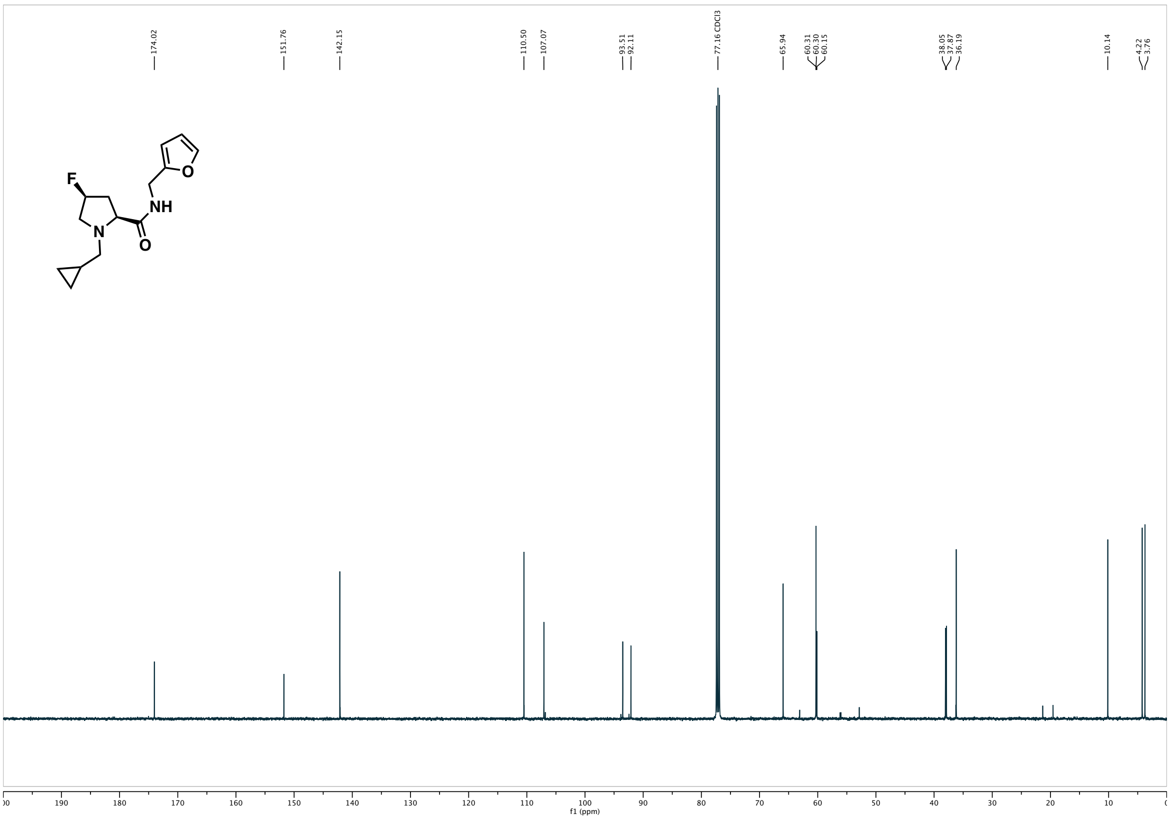
174.02
151.76
142.15
110.50
107.07
93.51
92.11
77.16 CDCl3
65.94
60.31
60.30
60.15
38.05
37.87
36.19
10.14
4.22
3.76
F
NH
N
O
O
f1 (ppm)

33

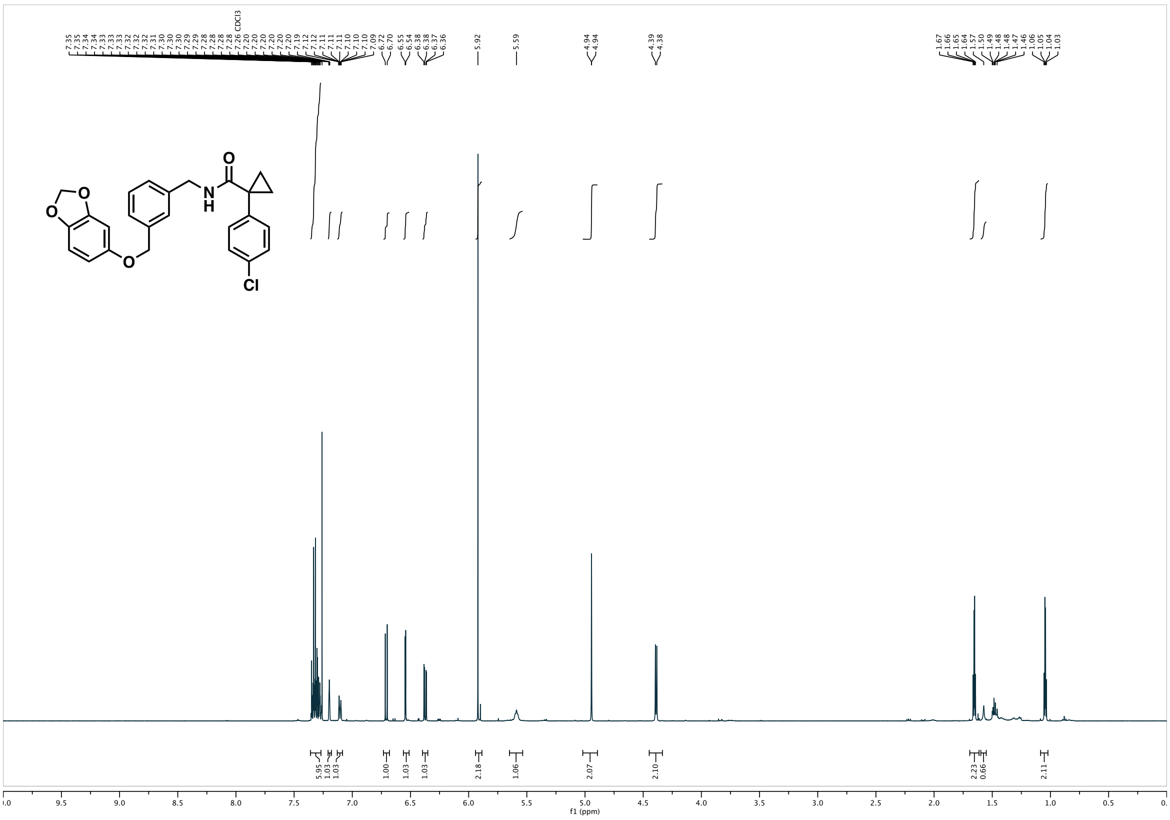

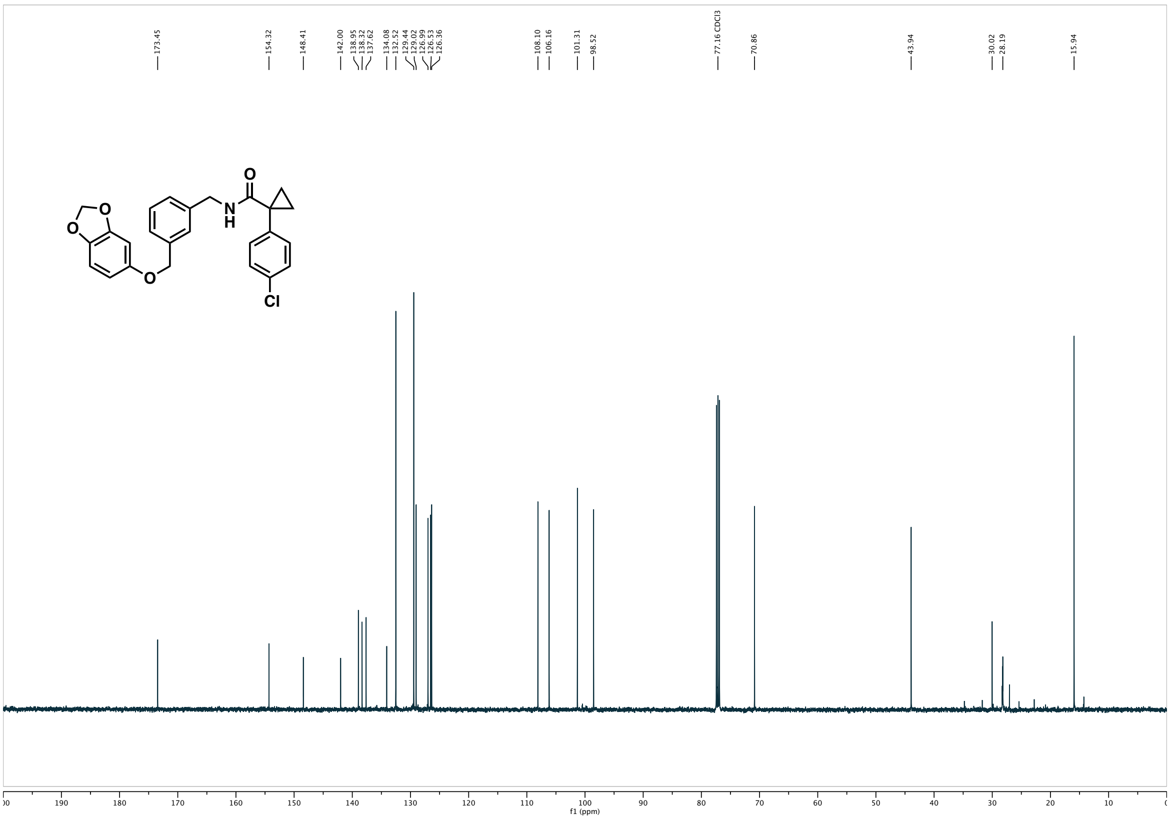

34

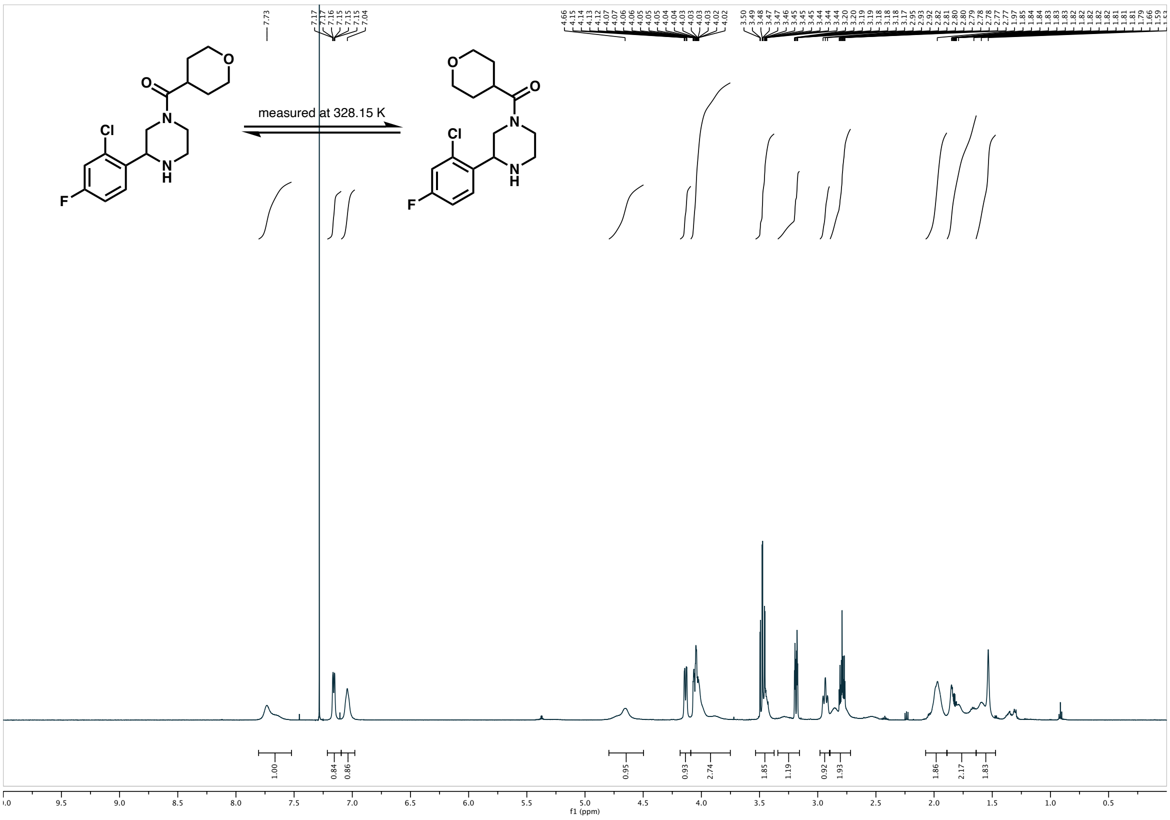
measured at 328.15 K
Cl
F
N
H
O
f1 (ppm)

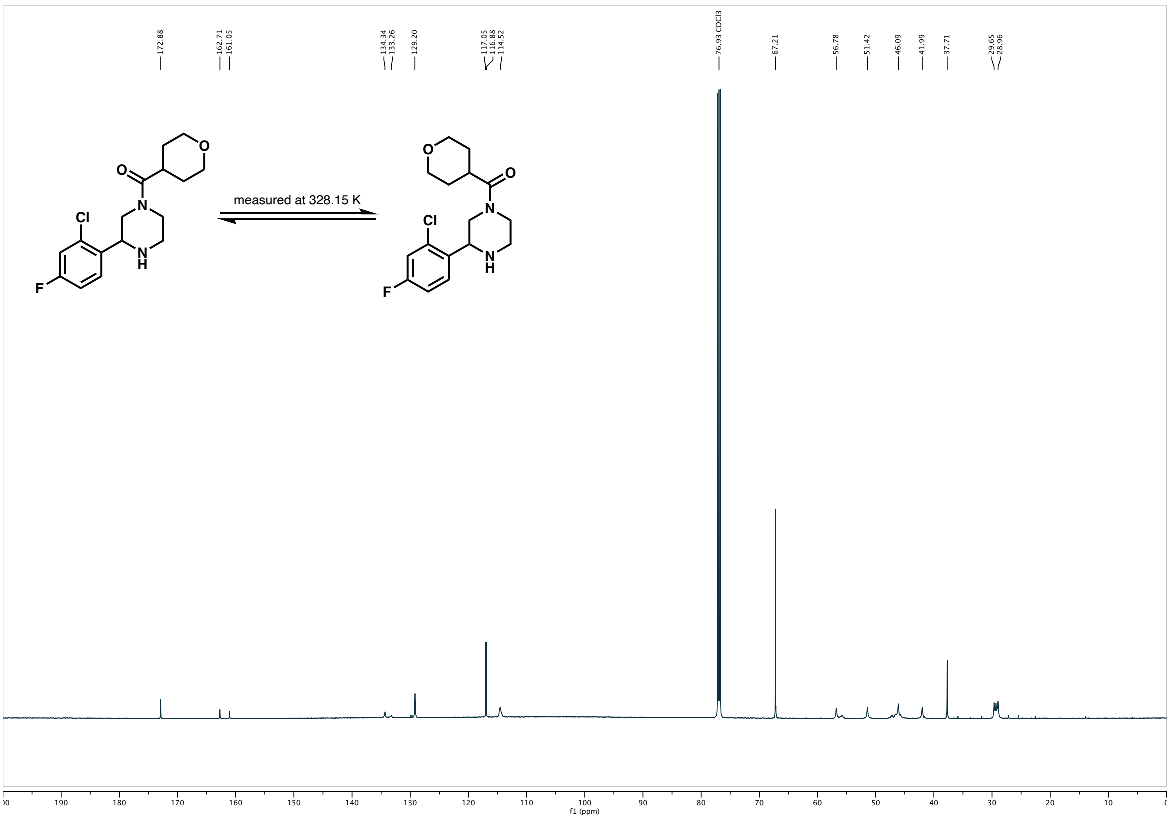
172.88
162.71
161.05
134.34
133.26
129.20
117.05
116.88
114.52
76.93 CDCl3
67.21
56.78
51.42
46.09
41.99
37.71
29.65
28.96
measured at 328.15 K
Cl
F
N
H
O
f1 (ppm)
